# Development of the Synthesis of Desepoxy-Tedanolide C

**DOI:** 10.1021/acs.joc.3c02437

**Published:** 2024-01-25

**Authors:** Daniel Lücke, Markus Kalesse

**Affiliations:** † Institute of Organic Chemistry, Gottfried Wilhelm Leibniz Universität Hannover, Schneiderberg 1B, 30167Hannover, Germany; ‡ Centre of Biomolecular Drug Research (BMWZ), Gottfried Wilhelm Leibniz Universität Hannover, Schneiderberg 38, 30167Hannover, Germany

## Abstract

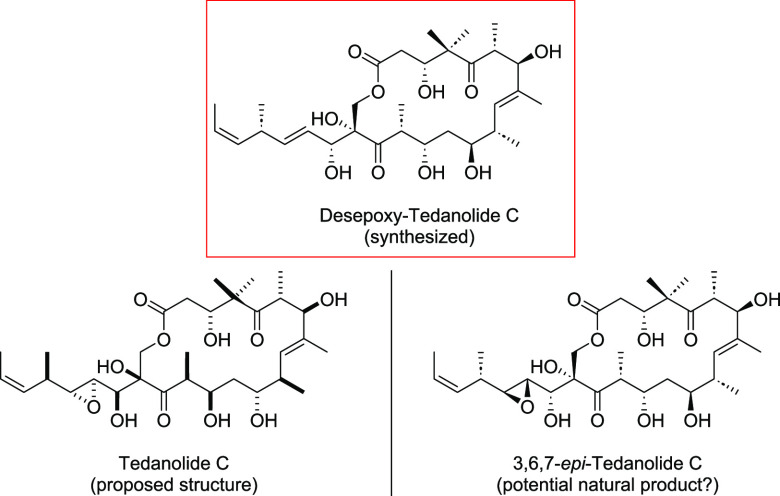

We are presenting the development of our route for the total synthesis of desepoxy-tedanolide C. Through the obtained analytical data, the proposed structure of tedanolide C is questioned and a different configuration for this natural product is proposed. Key steps of the synthesis are a Kiyooka aldol reaction that builds up the tertiary alcohol flanked by three oxygenated carbon atoms and two aldol reactions used for fragment couplings. A Julia–Kocienski olefination was used for installation of the side chain. Besides the successful synthesis, the development for the protecting group setup of the southwestern hemisphere is described in detail as well as another retrosynthetic attempt for building up the target molecule.

## Introduction

**Figure 1:**
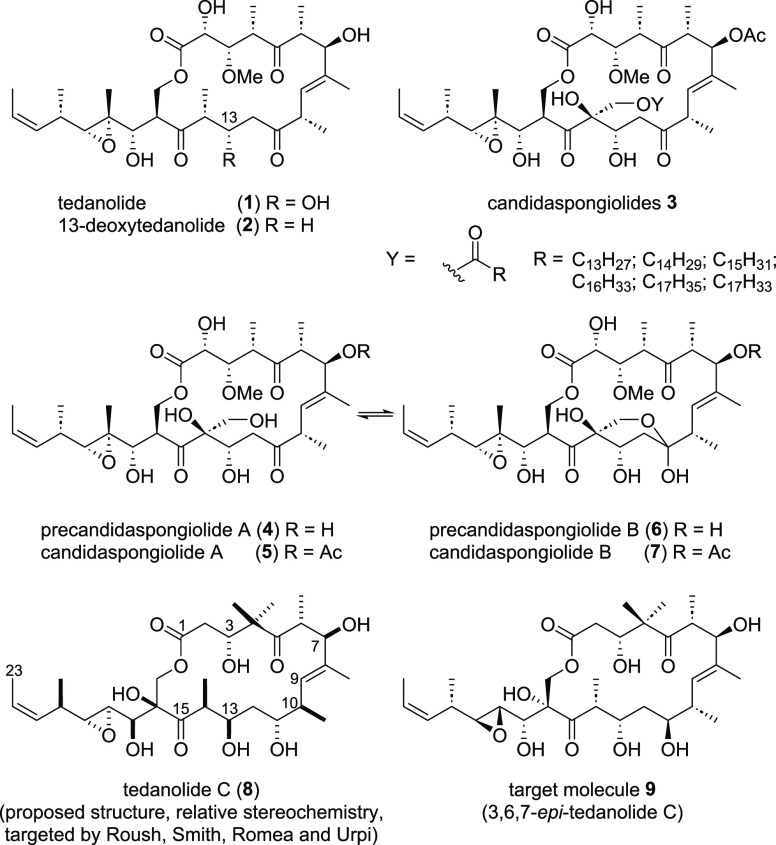
Family of tedanolides and structurally related natural products as well as the synthetic target of this work.

The tedanolides (Figure 1) are a family of natural products isolated from different marine sponges from different marine environments.^1^ The parent compound and name giver of this family is tedanolide (**1**) that was isolated in 1984 by Schmitz and co-workers.^2^ All family members show strong activities against different cancer cell lines (tedanolide (**1**): ED_50_ = 26.2 pM against lymphocytic leukemia cell lines;^2^ 13-deoxytedanolide (**2**): IC_50_ = 0.16 pM against P388 murine leukemia cell lines;^3^ tedanolide C (**8**): IC_50_ = 95.3 nM against HCT-116 cells^4^). Detailed studies about the biological profile showed the inhibition of translation as the primary biological target of the tedanolides and its non-natural congeners.^5^ Further structural related natural products were isolated by the group of McKee from the sponge genus *Candidaspongia* and named candidaspongiolides.^6^

Tedanolide C (**8**) was isolated by the group of Chris M. Ireland from the marine sponge *ircinia* sp. collected in Papua New Guinea. The methylation and oxygenation pattern of tedanolide C (**8**) differs significantly from tedanolide (**1**) and 13-deoxytedanolide (**2**). However, when comparing the proposed relative configuration of tedanolide C (**8**) with the other tedanolides, the divergence between northern (C1–C9) and southern (C10–C23) hemisphere is noteworthy. The relative configuration of tedanolide C (**8**) was determined by molecular modeling including coupling constants. There, the divergent configuration was rationalized by the coupling constant between H-9 and H-10, which is 9.2 Hz. It is argued that this coupling constant supports an eclipsed conformation of these protons and that only the proposed structure was in accordance with computationally generated isomers.^4^ Considering that the coupling constant of the same protons in tedanolide (**1**) are in a similar range (Schmitz: 10.8 Hz,^2^ Kalesse: 8.5 Hz,^7a^ Smith: 10.1 Hz,^7b^ Roush: 9.6 Hz^7c^) and that a common biosynthetic pathway of all tedanolides is quite likely, we decided to aim for **9** as the synthetic target of this work which is in better accordance with the other tedanolides. The absolute stereochemistry of **9** was chosen based on tedanolide (**1**) and 13-deoxytedanolide (**2**) as their structures were already confirmed by successful total syntheses.^7,8^ Previous synthetic attempts toward tedanolide C (**8**) were conducted by the groups of Roush,^9^ Smith,^10^ and Romea and Urpi^11^ targeting different enantiomers of the proposed structure. The syntheses of two fragments of tedanolide C (**8**) were reported by the Roush group so far. For their synthesis of the C1–C11 part of tedanolide C (**8**) the C7–C8 bond was build up by the addition of a vinyl zincate (C8–C11) to an aldehyde (C1–C7).^9b^ In contrast to this, we envisioned an aldol reaction for the linkage of northern and eastern part of the molecule. Their second publication describes the synthesis of the C15–C21 part of the natural product including the installation of the tertiary alcohol (C16) by stereoselective dihydroxylation.^9a^ We installed this key feature of tedanolide C by aldol chemistry, again. The Smith group reported the synthesis of two C1–C12 fragments differing only by the configuration at C10, which was established by an asymmetric hydroformylation.^10^ For the synthesis of their hydroformylation precursor, they started with an auxiliary controlled aldol reaction for building up the C6–C7 bond. Forming this bond by aldol chemistry is in line with our synthetic approach; however, we envisioned a late stage fragment coupling. The groups of Romea and Urpi reported the synthesis of the largest fragment of tedanolide C (**8**) so far. In their synthesis of the 13-*epi* C1–C15 fragment the key disconnection is again between C7 and C8, building up this bond by the addition of a vinyl nucleophile (13-*epi* C8–C15) to an aldehyde (C1–C7).^11^ As already mentioned, all these synthetic approaches aim for the synthesis of the proposed isomer of tedanolide C (**8**). However, our previously reported synthesis of desepoxy-tedanolide C supports our proposal of a configuration of tedanolide C that differs from the proposed structure.^12a^ In addition to the successful synthesis, we are now reporting our first retrosynthetic approach aiming for a different aldol disconnection between the southern and eastern part of the molecule. Furthermore, the development of the protecting group setup of the southern part is described in detail as this turned out to be crucial due to unexpected side reactions.^12b^

**Scheme 1:**
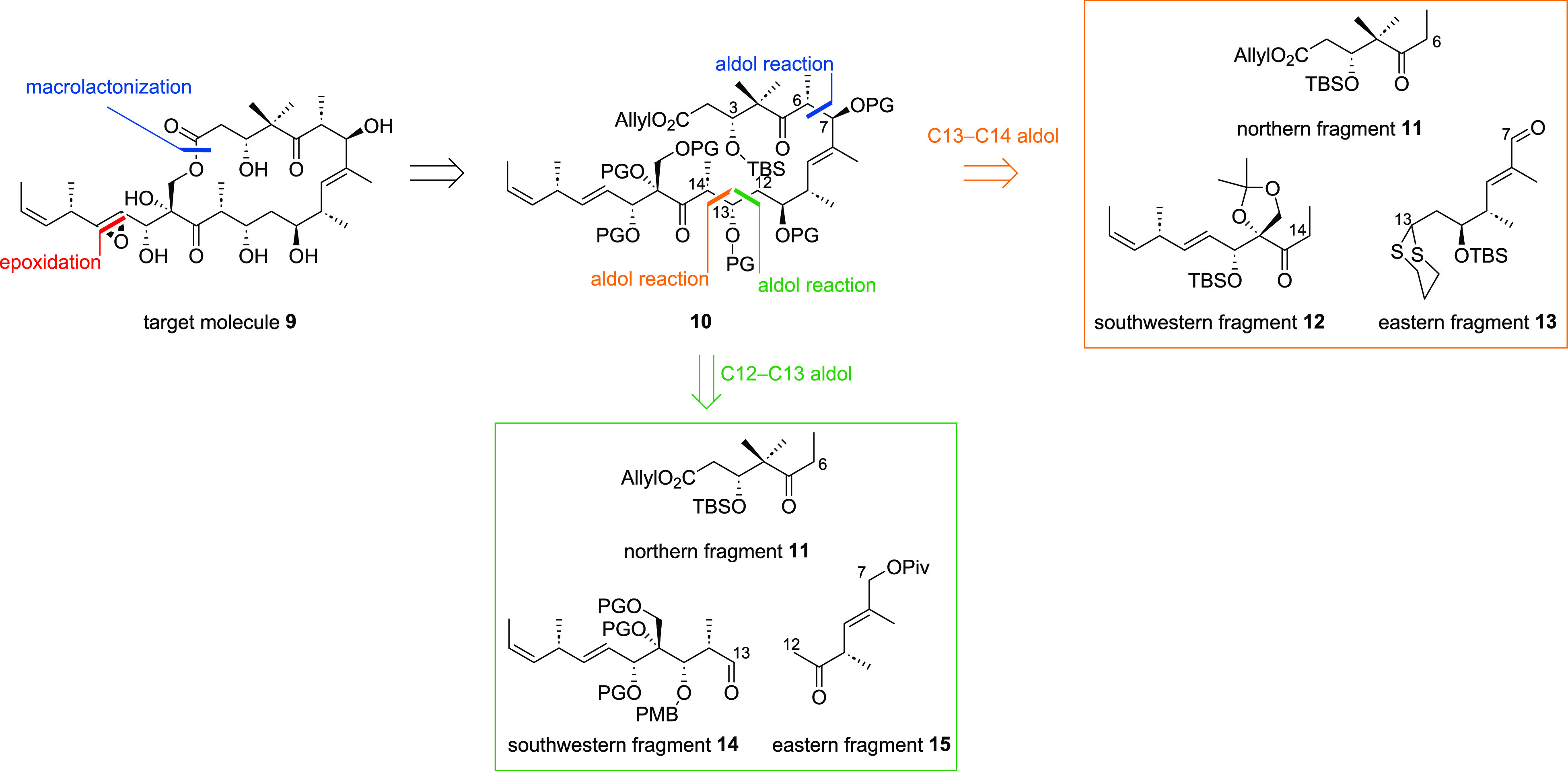
Retrosynthetic Analysis of Target Molecule **9**

Our retrosynthetic analysis of **9** (Scheme 1) took the state of knowledge of other tedanolide syntheses into account.^7,8^ We envisioned to install the epoxide in the side chain at the very end of the synthesis after a successful macrolactonization. Linear precursor **10** should be accessed from three fragments by applying two aldol reactions. For the first aldol disconnection, the C6–C7 bond was chosen dividing the molecule into northern fragment **11** and a remaining southern part. Through this disconnection, the stereogenic centers at C3, C6, and C7 could be installed at a late stage of the synthesis making it possible to access either target molecule **9** or the proposed tedanolide C (**8**) from an advanced intermediate. For the second aldol disconnection, the C12–C13 and the C13–14 bond were taken into consideration. The C13–C14 bond would benefit from no further need of redox manipulations after a successful bond forming event. However, due to the tertiary alcohol in the α-position of southwestern fragment **12**, the realization of this aldol reaction was expected to be highly challenging. The success of the C12–C13 aldol reaction was anticipated to be more likely not only because of the sterically less demanding substrates but due to the fact that this bond was successfully formed by aldol reactions in previous syntheses of tedanolide (**1**) and 13-deoxytedanolide (**2**).^7a,7c,8b^ However, the need of further redox manipulations would most likely increase the number of steps in this synthetic attempt. Due to the estimated higher efficiency of the C13–C14 aldol reaction, we aimed for this approach for the synthesis of **9** first.

## Results and Discussion

**Scheme 2:**
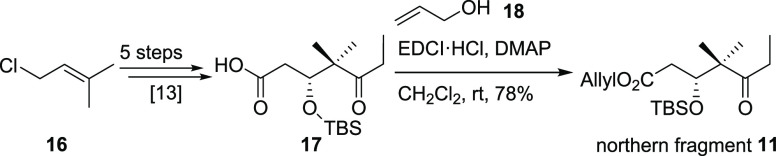
Synthesis of Northern Fragment **11**

For the synthesis of northern fragment **11** (Scheme 2), literature known carboxylic acid **17**(13) was first accessed from readily available **16** in a five step sequence. Subsequent esterification with allylic alcohol (**18**) provided **11** in 78% yield.

**Scheme 3:**
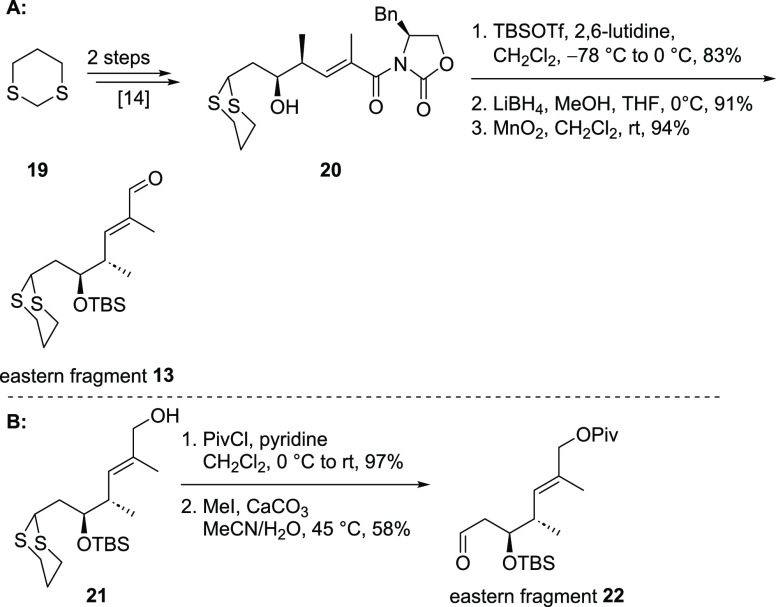
Synthesis of Eastern Fragments **11** and **22**

The synthesis of the eastern part (Scheme 3) commenced with 1,3-dithiane (**19**), which was transformed into alcohol **20** in a known sequence^14^ featuring a vinylogous Mukaiyama aldol reaction as the key step. Protection of the secondary alcohol followed by reductive removal of the auxiliary and oxidation of the obtained allylic alcohol gave access to eastern fragment **13** applicable to an aldol reaction with northern fragment **11**. As an alternative, the C13–C14 aldol-reaction between southwestern fragment **12** and eastern fragment **22** could be performed first. For this approach, allylic alcohol **21**, obtained after auxiliary removal, was protected as Piv ester and the dithiane was removed leading to **22** (Scheme 3B).

**Scheme 4:**
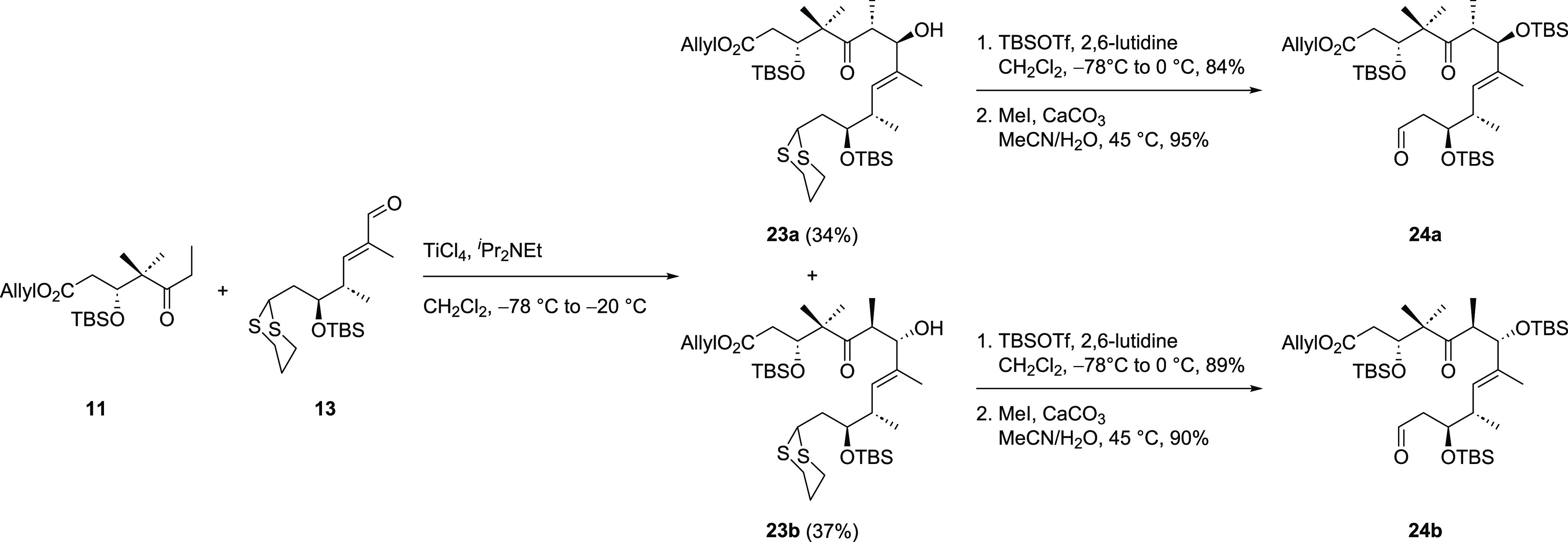
Aldol Reaction of Northern Fragment **11** and Eastern Fragment **13** and Syntheses of Aldehydes **24a** and **24b**

First investigations toward the C6–C7 aldol reaction were performed with northern fragment **11** and eastern fragment **13** (Scheme 4). The aldol reaction proceeded in a good overall yield of 71% when titanium tetrachloride and diisopropylethylamine were used for enolate formation. However, the desired aldol product **23a** was obtained in a nearly one-to-one mixture with its diastereomer **23b**, which could be separated at this stage. Unfortunately, other conditions screened for this aldol reaction did not lead to the desired product. The formation of the boron enolate of **11** was not possible in our hands applying standard conditions (^*n*^Bu_2_BOTf/^*i*^Pr_2_NEt), which is in accordance with literature reports for comparable ketones showing that neither boron nor tin enolates can be formed with these kind of compounds.^15^ When LDA was used, both starting materials could be recovered, which was rationalized by the dithianes acidic proton quenching the lithium enolate of **11**. The following reactions were carried out with both diastereomers of the aldol product and consisted of TBS protection of the secondary alcohol followed by liberation of the aldehyde through dithiane cleavage leading to **24a** and **24b**.

**Figure 2:**
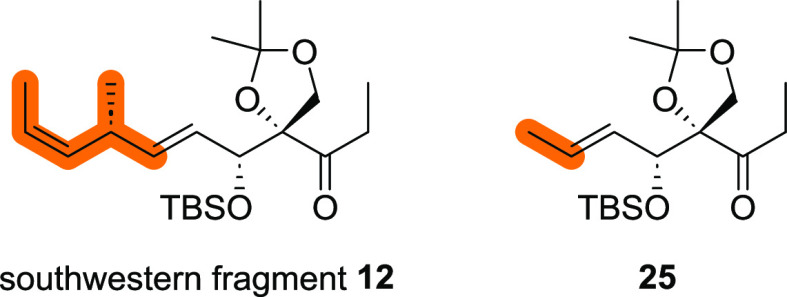
Southwestern fragment **12** and ketone **25**.

Since southwestern fragment **12** requires a synthetic sequence of 15 steps,^16^ we decided to begin our investigations on the C13–C14 aldol reaction with a simplified ketone such as **25**(Figure 2) , which could be accessed much faster and was expected to behave similarly to **12**.

**Scheme 5:**
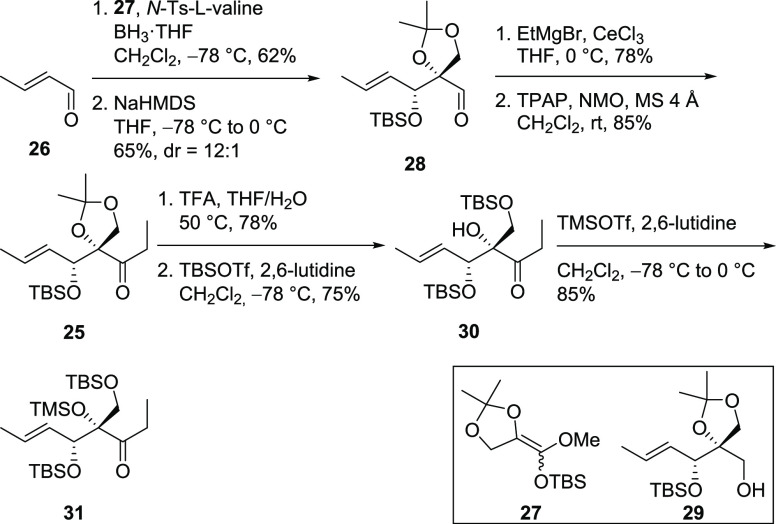
Syntheses of Ketones **25**, **30**, and **31**

The synthesis of **25** (Scheme 5) started with commercially available crotonaldehyde (**26**) that was first subjected to a Kiyooka aldol reaction^17^ with ketene acetal **27** installing the secondary and tertiary alcohol in a stereoselective way.^16,18^ Subsequent base-initiated silyl migration lead to aldehyde **28**. Prior to the addition of ethylmagnesium bromide, aldehyde **28** had to be premixed with anhydrous cerium trichloride to suppress the otherwise occurring reduction to alcohol **29** (obtained in 20–30% without the use of CeCl_3_).^19^ The obtained secondary alcohol (not shown) was oxidized employing the Ley–Griffith conditions^20^ giving access to ketone **25** in four steps. In addition to ketone **25** with its rigid acetonide protecting group for the primary and tertiary alcohol, we wanted to examine a potentially more flexible ketone bearing silyl protecting groups on the alcohols. For the acetonide removal, TFA in a mixture of THF and water at elevated temperatures turned out to be superior over other screened acids (CSA, PPTS, TfOH), which also showed TBS cleavage and thus gave the corresponding triol. The primary and secondary alcohol were both TBS protected using TBS triflate at low temperatures giving access to ketone **30**. Protection of the tertiary alcohol was achieved with TMS triflate leading to ketone **31**.

Before the first aldol reaction was conducted, we wanted to know whether it was possible to enolize the ketones. To investigate conditions for the enolization, ketones **25**, **30**, and **31** were treated with different bases and Lewis acids followed by quenching with deuterated water (Table 1). Ketone **25** could not be enolized with neither bases nor Lewis acids (Table 1, entries 1–4). For ketone **31**, it was not possible to form the lithium (^*n*^BuLi, entry 5) or boron (^*n*^Bu_2_BOTf, ^*i*^Pr_2_NEt, entry 6) enolate. On the other hand, Lewis acid-mediated enolization conditions led to cleavage of the primary TBS and the TMS ether. In cases where titanium tetrachloride was applied (entries 7 and 8), two outcomes were achieved. Only when the Lewis acid was added before the base, enolization could be observed in addition to silyl cleavage. With an inverted order of addition, no enolization occurred. Based on this observation we proposed that enolization of ketone **30** might also be possible due to the lack of the sterically demanding TMS ether on the tertiary alcohol in the α-position. We focused on base-mediated enolizations aiming for in situ deprotonation of the tertiary alcohol prior to enolate formation. Conditions previously applied to ketone **31** (entry 9) led to decomposition of the starting material exhibiting its higher reactivity. Usage of LDA at −78 °C (entry 10) showed partial enolization of **30**, which could be improved to full enolization at 0 °C (entry 11).

**Table 1:**
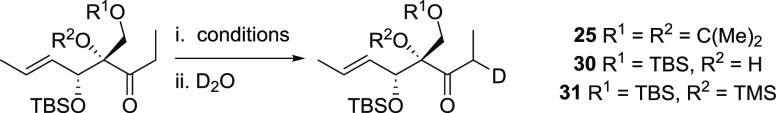
Selected Conditions Screened for the Enolization of Ketones **25**, **30**, and **31**

**entry**	**ketone**	**conditions**	**result**
1	**25**	LiHMDS, THF, – 78 °C	not enolized
2	**25**	KHMDS, 18-crown-6, THF, rt	not enolized
3	**25**	*^n^*BuLi, THF, rt	not enolized
4	**25**	*^n^*Bu_2_BOTf, ^*i*^Pr_2_NEt, CH_2_Cl_2_, 0 °C	not enolized
5	**31**	*^n^*BuLi, HMPA, THF, rt	not enolized
6	**31**	*^n^*Bu_2_BOTf, ^*i*^Pr_2_NEt, CH_2_Cl_2_, 0 °C	not enolized, cleavage of prim. TBS and TMS ether
7^a^	**31**	TiCl_4_, ^*i*^Pr_2_NEt, CH_2_Cl_2_, – 78 °C	enolized, cleavage of prim. TBS and TMS ether
8^b^	**31**	TiCl_4_, ^*i*^Pr_2_NEt, CH_2_Cl_2_, – 78 °C	not enolized
9	**30**	*^n^*BuLi, HMPA, THF, rt	decomposition
10	**30**	LDA, HMPA, THF, – 78 °C	partially enolized
11	**30**	LDA, HMPA, THF, 0 °C	enolized

a Added TiCl_4_ first.

b Added ^*i*^Pr_2_NEt first.

**Scheme 6:**
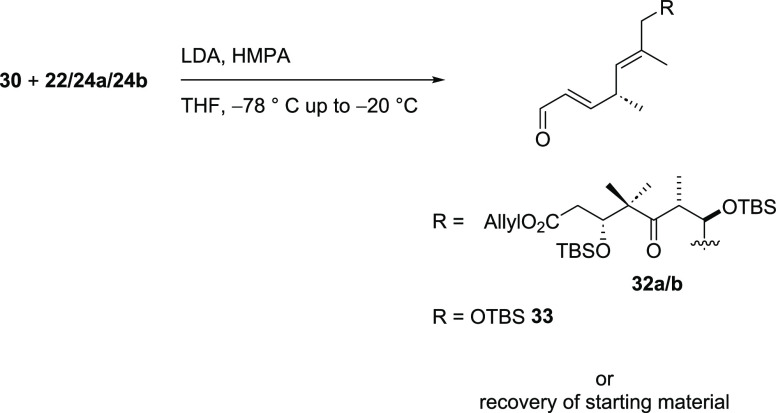
Attempts for the Aldol Reaction of Ketone **30**

Lastly, the C13–C14 aldol reaction of ketone **30** with eastern fragment **22** or the aldehydes **24a** and **b** (Scheme 6) did not lead to the desired products. Either no reaction occurred and the starting materials were recovered or the aldehydes were transferred into the corresponding enals (**32a**/**b** or **33**). The latter was most likely due to in situ enolization of the aldehydes followed by elimination of the TBS-protected alcohol. Due to the limitations regarding applicable conditions for the enolization, it was decided to further investigate on the C12–C13 aldol reaction.

**Scheme 7:**
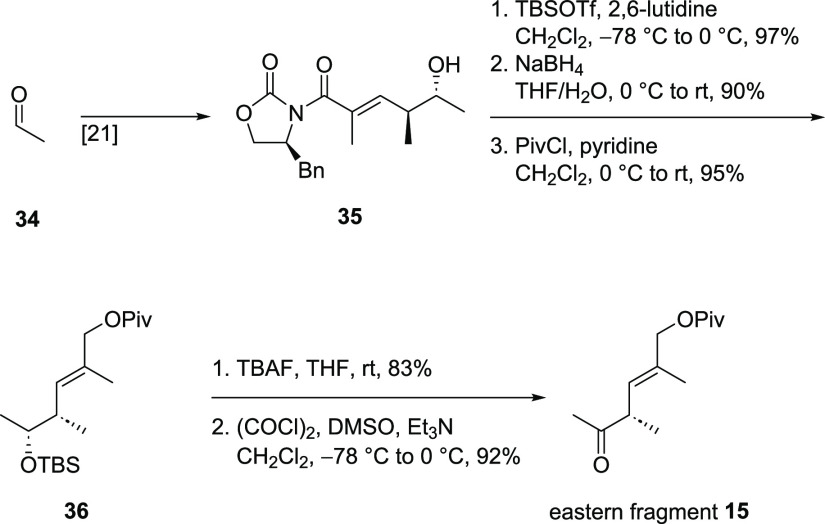
Synthesis of Eastern Fragment **15**

Acetic aldehyde (**34**) served as the starting material for the synthesis of eastern fragment **15** (Scheme 7). Applying a literature known protocol for a vinylogous Mukaiyama aldol reaction,^21^ it was transferred into alcohol **35**. A three step sequence consisting of protection of the alcohol, reductive removal of the auxiliary, and esterification of the primary alcohol led to Piv ester **36**. Cleavage of the TBS ether followed by Swern oxidation^22^ completed the synthesis of eastern fragment **15**.

**Scheme 8:**
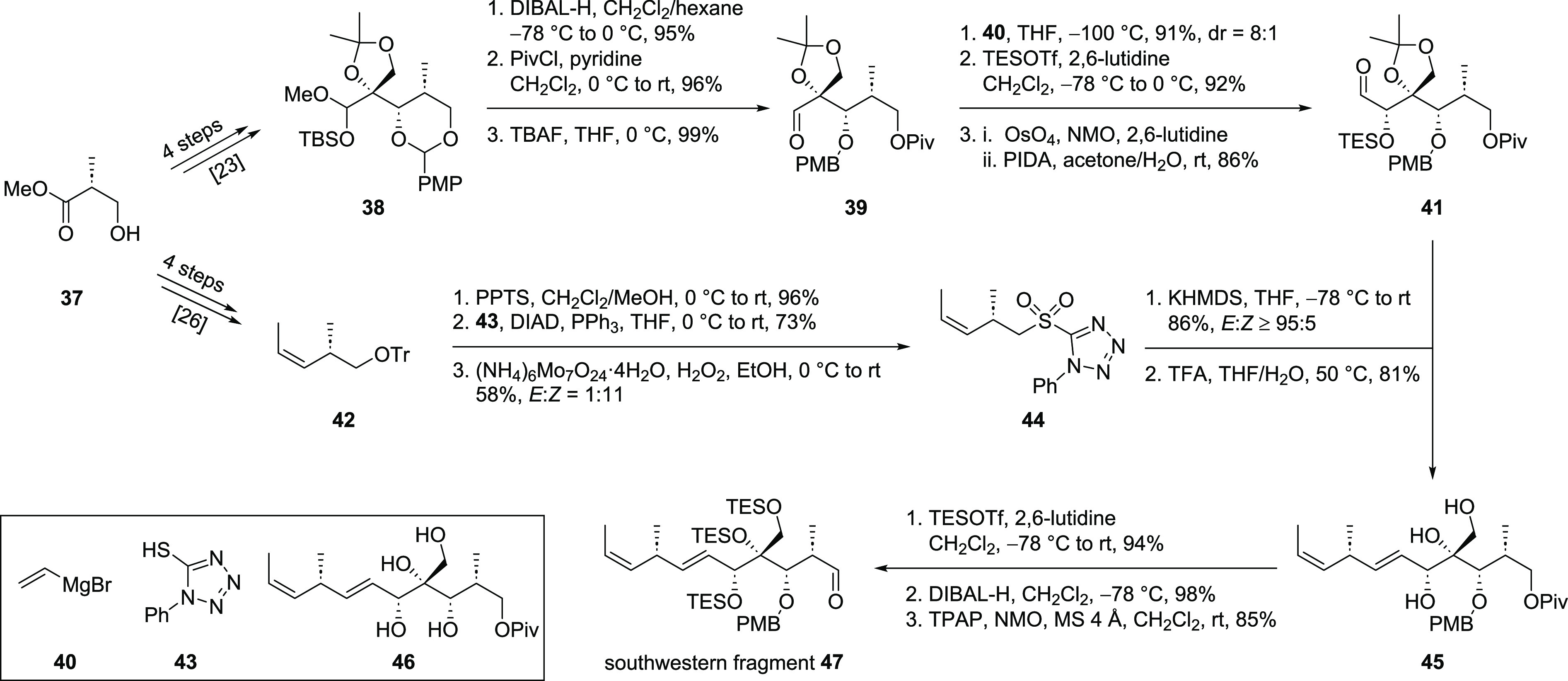
Synthesis of Southwestern Fragment **47**

A convergent approach dividing the southwestern fragment into a southern and a western part was envisioned. The synthesis of the southern part (Scheme 8) started from commercially available (*R*)-Roche ester (**37**), which was transferred into literature-known PMP-acetal^23^ first. In this sequence, stereoselective installation of the tertiary alcohol was again achieved by a Kiyooka aldol reaction. Acetal opening from the less hindered side with DIBAL-H followed by protection of the primary alcohol and TBAF-mediated cleavage of the mixed acetal led to aldehyde **39**. Addition of vinylmagnesium bromide (**40**) mainly gave the desired diastereomer of the obtained allylic alcohol (not shown), which was rationalized by Cram chelation control.^24^ The best result for this transformation was obtained at −100 °C whereas higher temperatures (0 °C or −78 °C), the addition of Lewis acids (CeCl_3_ or LaCl_3_·2LiCl) or a solvent switch to Et_2_O led to lower yields as well as diminished diastereoselectivities. TES protection of the secondary alcohol and subsequent oxidative cleavage of the olefin led to aldehyde **41**, ready for side chain installation through a Julia–Kocienski olefination.^25^ The required sulfone **44** was obtained in a seven steps sequence starting from (*R*)-Roche ester (**37**). First, the literature known alkene **42** was synthesized in four steps.^26^ Sulfone **44** was obtained after three additional transformations consisting of protecting group removal, installation of the sulfide and oxidation. Unfortunately, the oxidation conditions led to partial isomerization of the double bond, which could not be fully suppressed. The olefination proceeded in a good yield and an excellent *E*:*Z* ratio (≥95:5) building up the entire framework of the southwestern fragment. Due to the challenges we faced for the removal of the acetonide in our first approach, we decided to replace it by other protecting groups at this stage of the synthesis. Similar to the previous attempt, silyl ether cleavage occurred in addition to removal of the acetonide making triol **45** the product of this transformation. Furthermore, careful monitoring of this reaction was required as prolonged reaction times led to the formation of unusable tetraol **46** derived from PMB ether cleavage. All three alcohols of triol **45** were protected as TES ethers, expecting the selective liberation of the primary alcohol to be possible at a later stage of the synthesis. Removal of the Piv ester followed by Ley–Griffith oxidation^20^ finished the synthesis of southwestern fragment **47** that could now be subjected to the aldol reaction with eastern fragment **15**.

**Scheme 9:**
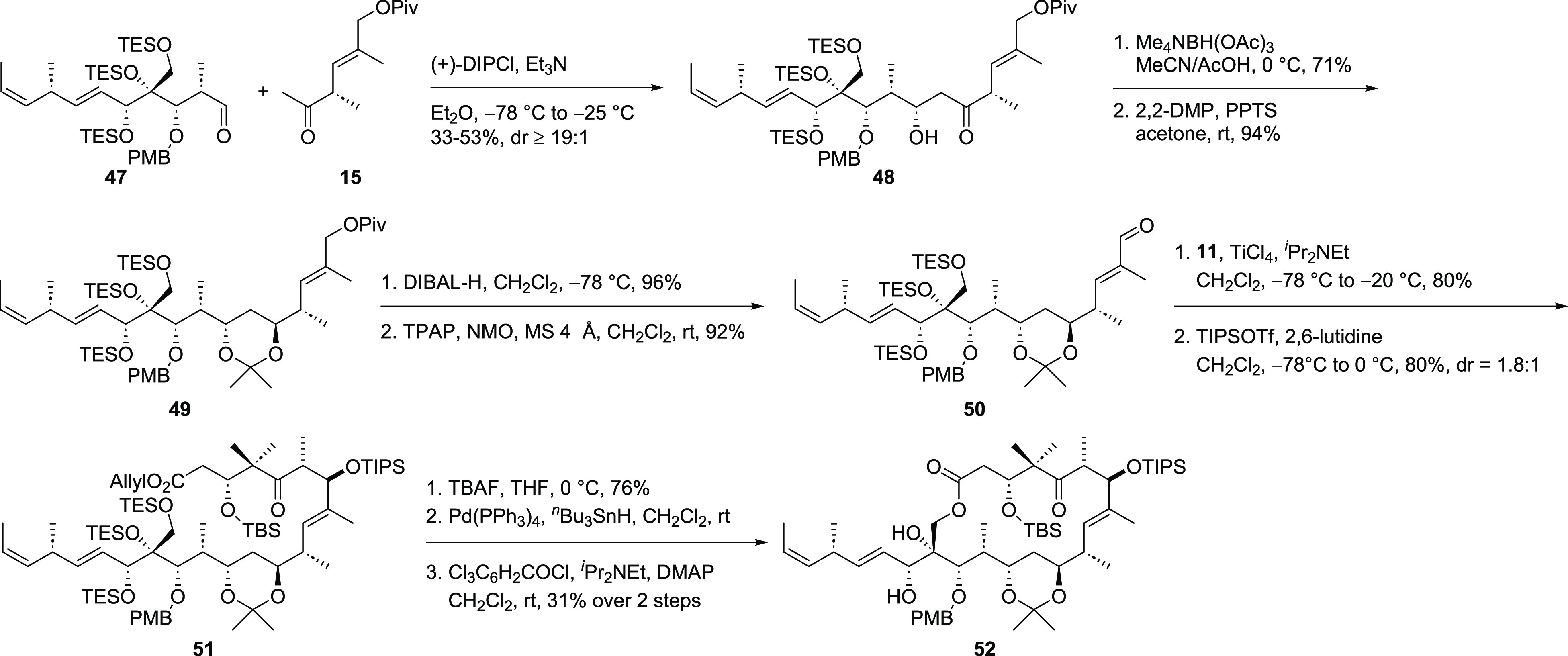
Synthesis of Macrolactone **52**

Alcohol **48** (Scheme 9) was obtained as a single diastereomer in the aldol reaction of eastern fragment **15** and southwestern fragment **47** when (+)-DIPCl was used for enolate formation. However, the yield of this reaction turned out to be highly dependent on its scale and dropped from 53% (300 μmol) to 33% (2.45 mmol) during up-scaling. An Evans–Saksena reduction^27^ established the desired *anti*-diol which was protected as its acetonide. Initially it was tried to protect both alcohols as TBS ethers which led to a mixture of different compounds most likely due to partial protecting group exchange of at least one TES ether. A similar observation was made by the Roush group during their work on tedanolide (**1**).^7c^ DIBAL-H mediated reduction of the Piv ester followed by oxidation with TPAP/NMO gave access to aldehyde **50** — the reaction partner for the aldol reaction with northern fragment **11**. In this reaction, the entire carbon framework of our target molecule was built up in a good yield of 80% with a low diastereomeric ratio of 1.8:1. Separation of the diastereomers was not possible until protection of the secondary alcohol, thus leaving the configuration of the major diastereomer unknown at this stage. Attempts for the selective cleavage of the primary TES ether turned out to be not fruitful. Under acidic conditions, either no reaction occurred or all TES ethers as well as the acetonide were removed at the same time. If fluoride sources were applied all TES ethers turned out to be nearly equal in reactivity so the obtained triol was used for the following macrolactonization. Liberation of the carboxylic acid followed by Yamaguchi lactonization^28^ led to macrolactone **52**. At this stage, it was not possible to reprotect the tertiary alcohol as its TES ether. Attempts to remove the PMB group in the presence of the tertiary alcohol were unsuccessful as a stable PMP-acetal was formed. As an alternative, it was tried to remove the PMB ether prior to the macrolactonization. However, treatment of TIPS ether **51** with DDQ led to a complex mixture of products still containing a *para*-methoxy arene moiety.

**Scheme 10:**
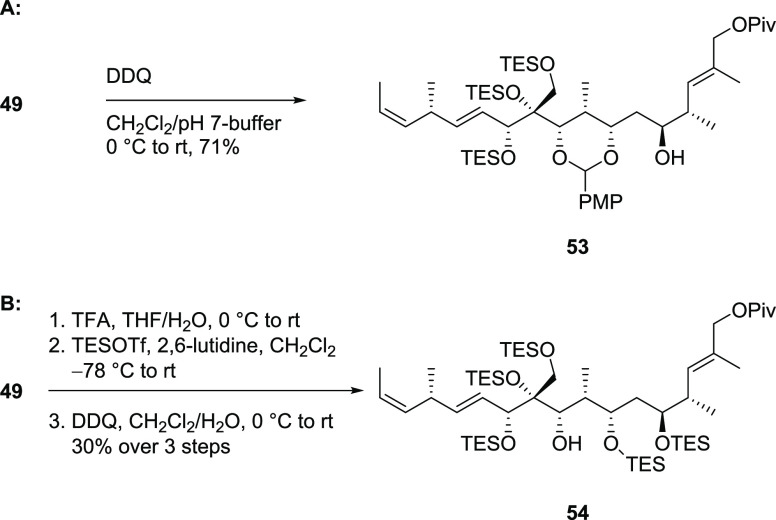
Attempts to PMB Ether Cleavage

Stepping back even further, acetonide **49** was chosen for the next attempt for removal of the PMB group. Treatment with DDQ gave a single product identified as PMP-acetal **53** (Scheme 10A). Formation of this most likely occurred by oxidation of the benzylic position followed by trapping of the oxonium ion through the neighboring oxygen and hydrolysis of the acetonide. This observation forced us to reconsider the protecting group for the 1,3-*anti*-diol. Estimating that silyl ethers would not cause any problems for the PMB ether cleavage, the acetonide was replaced by two TES ethers, which did not interfere with the subsequent removal of the PMB ether (Scheme 10B).

**Scheme 11:**
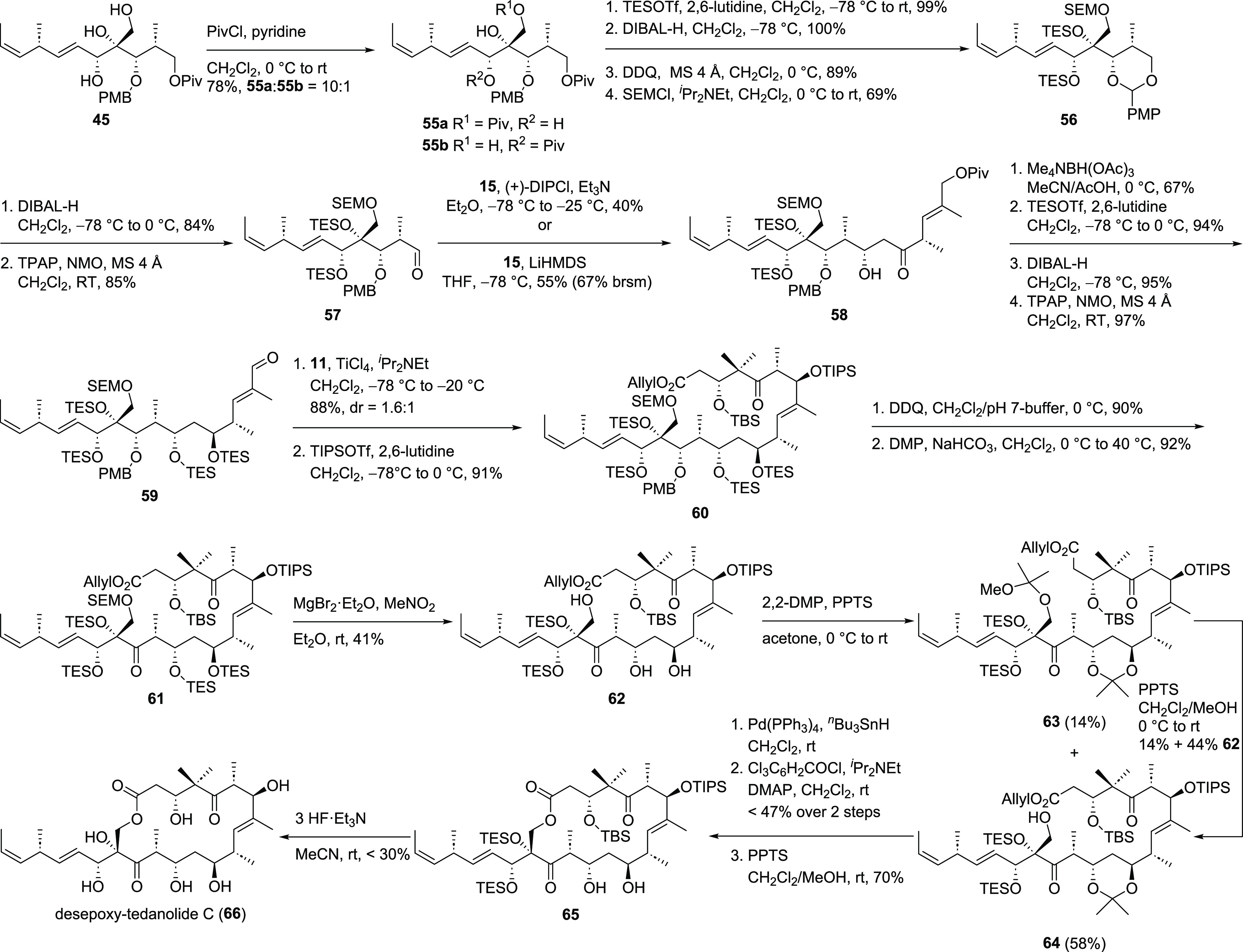
Synthesis of Desepoxy Target Molecule **66**

Given the fact that differentiation of the three TES ethers in **51** was highly challenging, we decided to replace the protecting group of the primary alcohol required for the macrolactonization before continuing our attempt toward our target molecule **9**. A SEM ether was deemed to be a suitable protecting group since there are protocols for its selective cleavage applying Lewis acids in the presence of other silyl ethers.^29^ Starting from triol **45** selective SEM protection of the primary alcohol turned out to be challenging (Scheme 11) as the secondary allylic alcohol was nearly equal in reactivity leading to a mixture of two mono SEM-protected products when 1 equiv of SEMCl was used. This problem was solved through a slightly prolonged reaction sequence. For this, a more bulky Piv ester was installed first, which proceeded in a better regioselectivity (10:1). TES protection of the secondary and tertiary alcohol followed by reductive removal of both Piv esters and regioselective PMP-acetal formation with the C13 alcohol left the desired hydroxyl group unprotected which was then SEM-protected to provide SEM ether **56**. Aldehyde **57** was obtained after opening of the PMP-acetal from the less hindered side followed by Ley–Griffith oxidation.^20^ For the aldol reaction with eastern fragment **15**, LiHMDS proved to be superior to (+)-DIPCl even though the reaction did not show full conversion. The Evans–Saksena protocol^27^ was again applied to obtain the *anti*-diol that was then protected as its bis-TES ether. Piv ester cleavage employing DIBAL-H followed by oxidation with TPAP^20^ led to aldehyde **59**, which was then used in the aldol reaction with northern fragment **11**. A very good yield of 88% along with a low diastereoselectivity (1.6:1) favoring the desired diastereomer was obtained for this fragment coupling. Separation of diastereomers was possible at this stage and the desired isomer was protected with TIPSOTf. Installation of the C15 ketone was achieved in two steps consisting of selective removal of the PMB ether followed by Dess–Martin oxidation^30^ at slightly elevated temperatures. Treatment of ketone **61** with MgBr_2_ and MeNO_2_^29^ removed the SEM ether as well as the TES ethers at C11 and C13 making triol **62** the main product of this reaction. Unfortunately, both alcohols of the *anti*-diol turned out to be superior over the primary alcohol in a macrolactonization leading to the undesired 12- and 14-membered macrolactones. Reprotection of the diol was achieved by conversion into its corresponding acetonide **64**. However, undesired methoxy ketal formation led to **63** as a side product. Attempts for the selective cleavage of the methoxy ketal of **63** provided **64** in low yields along with triol **62** as the main product, which was subjected again to the abovementioned sequence. Liberation of the carboxylic acid followed by Yamaguchi lactonization^28^ build up the macrocycle, which was globally deprotected in two steps (PPTS, HF·Et_3_N) leading to desepoxy target molecule **66** (Scheme 11).

**Figure 3:**
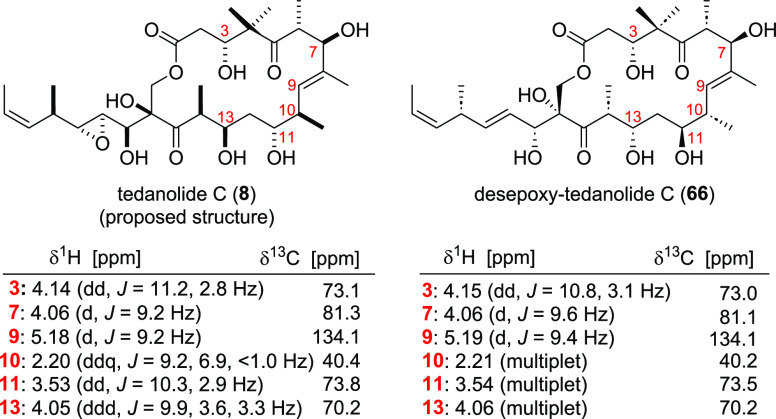
Comparison of indicative NMR shifts.

Unfortunately, **66** turned out to be unstable in MeOH and slowly decomposed during NMR measurements (retro-aldol products). However, it was possible to obtain a ^1^H NMR as well as a HSQC and HMBC, which were compared to the NMR data acquired for isolated tedanolide C (**8**)^4^ (Figure 3) showing a very good accordance of the chemical shift values for both macrolactones. Based on this, our proposal of a relative configuration between northern and southern hemisphere of tedanolide C that parallels the other tedanolides remains possible. However, more insights into the structure of tedanolide C could be gained either by the synthesis of its proposed structure **8** or our target molecule **9** and comparison with the data of the isolated compound.

## Conclusions

In summary, we have accomplished the synthesis of a desepoxidized diastereomer of the proposed structure of tedanolide C (**8**). To access the carbon framework of the target molecule two aldol reactions were applied. In addition, another unsuccessful aldol disconnection was investigated. Further challenges were the protecting group setup of the southern part of the molecule. In line with our proposal, the obtained NMR data indicate a different configuration for the natural product than initially proposed.

## Experimental Section

### General Information

Unless otherwise noted, all reactions were carried out under an argon atmosphere. The glassware used was dried under vacuum (∼0.6 mbar) using a heat gun. Air- and moisture-sensitive liquids and solutions were transferred via syringe flushed with argon prior to use. Unless otherwise noted, all reagents were obtained from commercial suppliers and used without further purification. All reactions were stirred with a magnetic stirrer. Temperatures refer to bath temperatures. **Dry solvents:***Tetrahydrofuran* was dried under an argon atmosphere by heating to reflux over sodium, using benzophenone as the indicator for air and moisture, and subsequent distillation. *Dichloromethane* and *triethylamine* were dried under a nitrogen atmosphere by heating to reflux over calcium hydride and subsequent distillation. *Acetone*, *acetonitrile*, *diethyl ether*, and *methanol* were bought from Acros Organics. **Flash column chromatography:** The silica gel used was obtained from Macharey-Nagel (40–63 μm, 240–400 mesh). Eluent is given in volume ratios (v/v). **Thin layer chromatography:** All reactions were monitored by TLC (0.2 mm, silica gel, F_254_, aluminum-backed, Macharey-Nagel) with detection by UV light (λ = 254 nm) and/or treateqmonium molybdate or acidic vanillin stain. ^**1**^**H NMR:** NMR data were recorded on a DPX 400 (Bruker), an AMX 400 (Bruker), an Ascend 400 Avance III HD (Bruker), a DRX 500 (Bruker), or an Ascend 600 (Bruker) instrument. Spectra were calibrated against the residual solvent signal: δ(CDCl_3_) = 7.26 ppm, δ(C_6_D_6_) = 7.16 ppm, δ(CD_3_OD) = 3.31 ppm. Data are recorded as follows: chemical shift, δ in parts per million (ppm), coupling constant, *J* in Hz, multiplicity (s, singlet; d, doublet; t, triplet; q, quartet; p, pentet; sext., sextet; m, multiplet; bs, broad singlet, or combination of these acronyms), and integration. NMR spectra were processed using the software TopSpin (Bruker). ^**13**^**C NMR:** NMR data were recorded on a DPX 400 (Bruker) and an AMX 400 (Bruker) instrument. Spectra were calibrated against the residual solvent signal: δ(CDCl_3_) = 77.16 ppm, δ(C_6_D_6_) = 128.06 ppm, δ(CD_3_OD) = 49.00 ppm. **2D-NMR:**^1^H–^1^H-COSY, ^1^H–^13^C-HSQC, and ^1^H–^13^C-HMBC data were recorded on an Ascend 600 (Bruker) instrument. NMR spectra were processed using the software TopSpin (Bruker). Structural assignments were made with additional information from gCOSY, gHSQC, and gHMBC experiments. **High resolution mass spectrometry (HRMS):** Electrospray ionization (ESI-HRMS) mass spectra were obtained using either a LCT Premier (Waters) or a Q-Tof Premier (Waters). **Optical rotation [α]**_**D**_^**T**^**:** Optical rotations were recorded on a Perkim-Elmer 341 polarimeter or a Krüss Optronic (P3000) using the following standard conditions: wavelength 589.3 nm (sodium D line), cell length 1 dm, solvent and sample concentration (in g/100 mL) are given with individual experiment. **Melting point:** Melting points were determined using an OptiMelt MPA 100 instrument (Stanford Research System).

#### Allyl (*R*)-3-((*tert*-Butyldimethylsilyl)oxy)-4,4-dimethyl-5-oxoheptanoate (**11**)

1-Ethyl-3-(3-(dimethylamino)propyl)carbodiimide hydrochloride (1.17 g, 6.12 mmol, 1.20 equiv) was added to a solution of carboxylic acid **15**(13) (1.54 g, 5.10 mmol, 1.00 equiv), allyl alcohol (**16**) (0.70 mL, 10.2 mmol, 2.00 equiv), and 4-dimethylaminopyridine (0.06 g, 0.51 mmol, 0.10 equiv) in CH_2_Cl_2_ (25 mL) at room temperature. The reaction mixture was stirred for 2.75 h before water was added. The phases were separated and the aqueous layer was extracted with CH_2_Cl_2_ (3×). The combined organic layers were washed with brine, dried over MgSO_4_, and concentrated *in vacuo*. The crude product was purified via column chromatography (petroleum ether:EtOAc 20:1) providing northern fragment **11** (1.36 g, 3.98 mmol, 78%) as a colorless oil. ^**1**^**H NMR (400 MHz, CDCl**_**3**_**)** δ = 5.96–5.86 (m, 1H), 5.32 (dq, *J* = 17.2, 1.4 Hz, 1H), 5.24 (dq, *J* = 10.4, 1.4 Hz, 1H), 4.56 (dq, *J* = 5.8, 1.4 Hz, 2H), 4.50 (dd, *J* = 6.9, 3.6 Hz, 1H), 2.62–2.43 (m, 3H), 2.33 (dd, *J* = 16.2, 7.0 Hz, 1H), 1.13 (s, 3H), 1.08 (s, 3H), 1.00 (t, *J* = 7.2 Hz, 3H), 0.84 (s, 9H), 0.06 (s, 3H), 0.01 (s, 3H) ppm; ^**13**^**C{1H}-NMR (100 MHz, CDCl**_**3**_**)** δ = 215.2, 171.8, 132.1, 118.7, 73.8, 65.5, 52.7, 39.6, 31.9, 26.0, 21.2, 20.8, 18.3, 7.9, −4.3, −4.8 ppm; **HRMS** (ESI) *m*/*z*: [M + Na]^+^ calcd for C_18_H_34_O_4_SiNa 365.2124; found 365.2128; **[α]**_**D**_^**22.3**^ = +21.6 (*c* = 1.00, CHCl_3_); **R**_*f*_ = 0.30 (petroleum ether:EtOAc 19:1).

#### (*S*)-4-Benzyl-3-((4*S*,5*S*,*E*)-5-((*tert*-butyldimethylsilyl)oxy)-6-(1,3-dithian-2-yl)-2,4-dimethylhex-2-enoyl)oxazolidin-2-one (**67**)

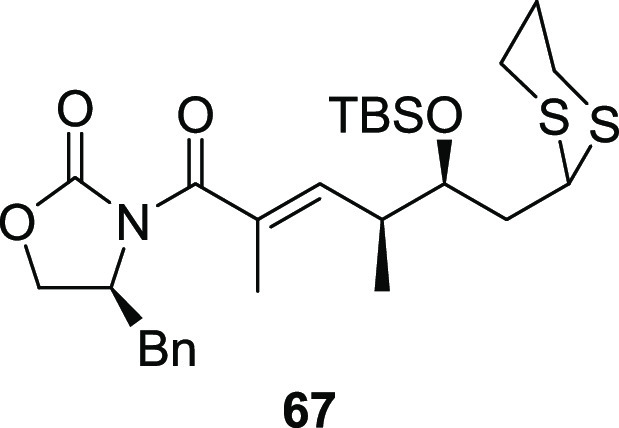



2,6-Lutidine (1.9 mL, 16.4 mmol, 2.65 equiv) and *tert*-butyldimethylsilyl trifluoromethanesulfonate (1.9 mL, 8.19 mmol, 1.32 equiv) were added successively to a solution of alcohol **20**(14) (2.69 g, 6.19 mmol, 1.00 equiv) in CH_2_Cl_2_ (31 mL) at −78 °C. The reaction mixture was stirred for 10 min at −78 °C and for 15 min at 0 °C before a saturated aqueous solution of NaHCO_3_ was added. The phases were separated, and the aqueous layer was extracted with CH_2_Cl_2_ (3×). The combined organic layers were successively washed with an aqueous solution of KHSO_4_ (1 M), a saturated aqueous solution of NaHCO_3_ and brine, dried over MgSO_4_, and concentrated *in vacuo*. The crude product was purified via column chromatography (petroleum ether:EtOAc 10:1 → 9:1 → 8:1) providing TBS-ether **67** (2.81 g, 5.11 mmol, 83%) as a colorless wax. ^**1**^**H NMR (400 MHz, CDCl**_**3**_**)** δ = 7.35–7.25 (m, 3H), 7.22–7.19 (m, 2H), 5.95–5.92 (m, 1H), 4.70–4.63 (m, 1H), 4.25–4.21 (m, 1H), 4.14 (dd, *J* = 8.9, 4.8 Hz, 1H), 4.07 (dd, *J* = 8.2, 6.4 Hz, 1H), 3.94–3.90 (m, 1H), 3.36 (dd, *J* = 13.5, 3.4 Hz, 1H), 2.92–2.79 (m, 5H), 2.71–2.62 (m, 1H), 2.14–2.07 (m, 1H), 1.98–1.83 (m, 3H), 1.93 (d, *J* = 1.4 Hz, 3H), 1.01 (d, *J* = 6.8 Hz, 3H), 0.90 (s, 9H), 0.12 (s, 3H), 0.08 (s, 3H) ppm; ^**13**^**C{1H}-NMR (100 MHz, CDCl**_**3**_**)** δ = 171.9, 153.0, 141.0, 135.4, 130.5, 129.6, 129.0, 127.4, 71.8, 66.5, 55.7, 43.8, 40.9, 38.6, 37.6, 30.4, 30.1, 26.1, 18.3 (2 different carbon atoms), 14.5, 14.0, −4.0, −4.3 ppm; **HRMS** (ESI) *m*/*z*: [M + Na]^+^ calcd for C_28_H_43_NO_4_S_2_SiNa 572.2301; found 572.2299; **[α]**_**D**_^**23.0**^ = +4.76 (*c* = 0.84, CHCl_3_); **R**_*f*_ = 0.46 (petroleum ether:EtOAc 3:1).

#### (4*S*,5*S*,*E*)-5-((*tert*-Butyldimethylsilyl)oxy)-6-(1,3-dithian-2-yl)-2,4-dimethylhex-2-en-1-ol (**21**)

Lithium borohydride (4 M in THF, 6.4 mL, 25.6 mml, 5.02 equiv) was added to a solution of TBS-ether **67** (2.80 g, 5.10 mmol, 1.00 equiv) and MeOH (1.00 mL, 24.7 mmol, 4.83 equiv) in THF (45 mL) at 0 °C. The reaction mixture was stirred for 3 h before a mixture of EtOAc, water, and brine (1:1:1) was added. The phases were separated and the aqueous layer was extracted with MTBE (3×). The combined organic layers were washed with a saturated aqueous solution of NaHCO_3_ and brine, dried over MgSO_4_, and concentrated *in vacuo*. The crude product was purified via column chromatography (petroleum ether:EtOAc 3:1) providing alcohol **21** (1.75 g, 4.65 mmol, 91%) as a colorless oil. ^**1**^**H NMR (400 MHz, CDCl**_**3**_**)** δ = 5.35–5.31 (m, 1H), 4.07 (dd, *J* = 8.4, 6.2 Hz, 1H), 4.00 (s, 2H), 3.85–3.81 (m, 1H), 2.92–2.78 (m, 4H), 2.56–2.47 (m, 1H), 2.15–2.08 (m, 1H), 1.92–1.77 (m, 3H), 1.69 (d, *J* = 1.4 Hz, 3H), 1.30 (bs, 1H), 0.94 (d, *J* = 6.8 Hz, 3H), 0.90 (s, 9H), 0.11 (s, 3H), 0.06 (s, 3H) ppm; ^**13**^**C{1H}-NMR (100 MHz, CDCl**_**3**_**)** δ = 134.8, 129.2, 72.7, 69.2, 44.3, 40.8, 38.0, 30.8, 30.4, 26.1, 18.4 (2 different carbon atoms), 16.0, 14.2, −4.0, −4.1 ppm; **HRMS** (ESI) *m*/*z*: [M + Na]^+^ calcd for C_18_H_36_O_2_S_2_SiNa 399.1824; found 399.1821; **[α]**_**D**_^**22.8**^ = —11.2 (*c* = 0.91, CHCl_3_); **R**_*f*_ = 0.45 (petroleum ether:EtOAc 3:1).

#### (4*S*,5*S*,*E*)-5-((*tert*-Butyldimethylsilyl)oxy)-6-(1,3-dithian-2-yl)-2,4-dimethylhex-2-enal (**13**)

The glassware was neither dried nor purged with inert gas prior to usage. Activated manganese dioxide (874 mg, 10.0 mmol, 20.0 equiv) was added to a solution of alcohol **21** (189 mg, 0.50 mmol, 1.00 equiv) in CH_2_Cl_2_ (10.0 mL) at room temperature. The reaction mixture was stirred for 31 h before the solids were filtered off by using CeliteⓇ (CH_2_Cl_2_), and the filtrate was concentrated *in vacuo*. Eastern fragment **13** (177 mg, 0.47 mmol, 94%) was obtained as a colorless oil and was used in the next reaction without detailed characterization.

#### (4*S*,5*S*,*E*)-5-((*tert*-Butyldimethylsilyl)oxy)-6-(1,3-dithian-2-yl)-2,4-dimethylhex-2-en-1-yl Pivalate (**68**)

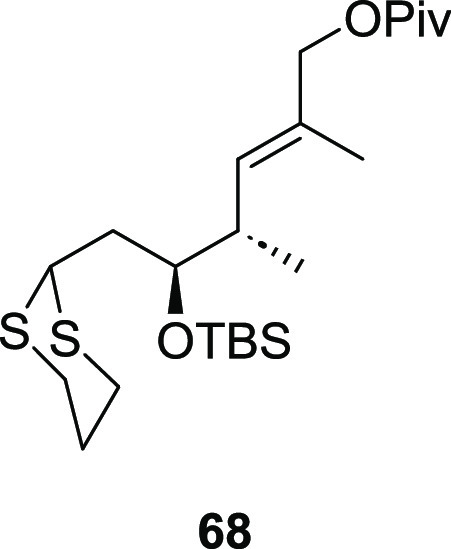



Pyridine (0.43 mL, 5.31 mmol, 20.0 equiv) and pivaloyl chloride (39 μL, 0.32 mmol, 1.14 equiv) were added successively to a solution of alcohol **21** (107 mg, 0.28 mmol, 1.00 equiv) in CH_2_Cl_2_ (2.7 mL) at 0 °C. The reaction mixture was stirred for 5 min at 0 °C and for 17 h at room temperature before water was added. The phases were separated and the aqueous layer was extracted with CH_2_Cl_2_ (3×). The combined organic layers were successively washed with an aqueous solution of KHSO_4_ (1 M), a saturated aqueous solution of NaHCO_3_ and brine, dried over MgSO_4_, and concentrated *in vacuo*. The crude product was purified via column chromatography (petroleum ether:EtOAc 10:1) providing pivalate **68** (125 mg, 0.27 mmol, 97%) as a colorless oil. ^**1**^**H NMR (400 MHz, CDCl**_**3**_**)** δ = 5.38–5.34 (m, 1H), 4.45 (dd, *J* = 12.2, 0.9 Hz, 1H), 4.41 (dd, *J* = 12.2, 0.9 Hz, 1H), 4.06 (dd, *J* = 8.2, 6.4 Hz, 1H), 3.88–3.84 (m, 1H), 2.92–2.77 (m, 4H), 2.57–2.48 (m, 1H), 2.15–2.08 (m, 1H), 1.93–1.78 (m, 3H), 1.66 (d, *J* = 1.3 Hz, 3H), 1.21 (s, 9H), 0.94 (d, *J* = 6.9 Hz, 3H), 0.89 (s, 9H), 0.10 (s, 3H), 0.05 (s, 3H) ppm; ^**13**^**C{1H}-NMR (100 MHz, CDCl**_**3**_**)** δ = 178.5, 132.2, 130.3, 72.4, 70.2, 44.2, 40.6, 39.0, 37.8, 30.7, 30.3, 27.4, 26.2, 26.1, 18.3, 15.5, 14.3, −4.1, −4.2 ppm; **HRMS** (ESI) *m*/*z*: [M + Na]^+^ calcd for C_23_H_44_O_3_S_2_SiNa 483.2399; found 483.2400; **[α]**_**D**_^**23.0**^ = —11.7 (*c* = 0.60, CHCl_3_); **R**_*f*_ = 0.65 (petroleum ether:EtOAc 9:1).

#### (4*S*,5*S*,*E*)-5-((*tert*-Butyldimethylsilyl)oxy)-2,4-dimethyl-7-oxohept-2-en-1-yl Pivalate (**22**)

The glassware was neither dried nor purged with inert gas prior to usage. Calcium carbonate (300 mg, 2.99 mmol, 20.0 equiv) and iodomethane (0.19 mL, 2.99 mmol, 20.0 equiv) were added successively to a solution of pivalate **68** (69.0 mg, 0.15 mmol, 1.00 equiv) in a mixture of acetonitrile (3.6 mL) and water (0.40 mL) at room temperature. The reaction mixture was stirred for 20.5 h at 45 °C before the solids were filtered by using Celite (Et_2_O). The filtrate was washed with water (2×) and brine, dried over MgSO_4_, and concentrated *in vacuo*. The crude product was purified via column chromatography (petroleum ether:EtOAc 10:1) providing eastern fragment **22** (32.0 mg, 86.3 μmol, 58%) as a colorless oil. Eastern fragment **22** was used in the next reaction without detailed characterization.

#### Allyl (3*R*,6*R*,7*R*,10*S*,11*S*,*E*)-3,11-bis((*tert*-butyldimethylsilyl)oxy)-12-(1,3-dithian-2-yl)-7-hydroxy-4,4,6,8,10-pentamethyl-5-oxododec-8-enoate (**23a**)

Titanium tetrachloride (1 M in CH_2_Cl_2_, 0.64 mL, 0.64 mmol, 1.40 equiv) and *N*,*N*-diisopropylethylamine (0.22 mL, 1.29 mmol, 2.80 equiv) were added successively to a solution of northern fragment **11** (236 mg, 0.67 mmol, 1.50 equiv) in CH_2_Cl_2_ (4.5 mL) at −78 °C. The reaction mixture was stirred for 70 min before a solution of eastern fragment **13** (296 mg, 264 μmol, 1.00 equiv) in CH_2_Cl_2_ (4.0 mL) was added dropwise over the course of 5 min. The reaction mixture was stirred for 10 min at −78 °C and for 3.25 h at −20 °C before an aqueous solution of phosphate buffer (pH = 7) was added. The phases were separated and the aqueous layer was extracted with CH_2_Cl_2_ (3×). The combined organic layers were washed with brine, dried over MgSO_4_, and concentrated *in vacuo*. The crude product was purified via column chromatography (pentane:Et_2_O 8:1 → 7:1 → 4:1) providing alcohol **23a** (111 mg, 0.15 mmol, 34%, mixture with eastern fragment **13**, 126 mg in total) and its diastereomer **23b** (122 mg, 0.17 mmol, 37%, mixture with eastern fragment **13**, 133 mg in total) as yellow oils. Both alcohols were used in the next reaction without detailed characterization.

#### Allyl (3*R*,6*R*,7*R*,10*S*,11*S*,*E*)-3,7,11-Tris((*tert*-butyldimethylsilyl)oxy)-12-(1,3-dithian-2-yl)-4,4,6,8,10-pentamethyl-5-oxododec-8-enoate (**69a**)

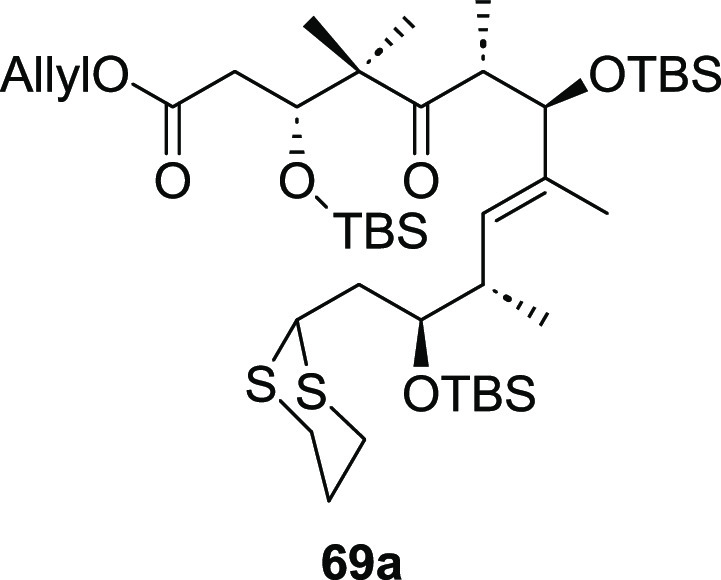



2,6-Lutidine (50 μL, 441 μmol, 3.00 equiv) and *tert*-butyldimethylsilyl trifluoromethanesulfonate (50 μL, 220 μmol, 1.50 equiv) were added successively to a solution of alcohol **23a** (105 mg, 147 μmol, 1.00 equiv) in CH_2_Cl_2_ (1.5 mL) at −78 °C. The reaction mixture was stirred for 10 min at −78 °C and for 40 min at 0 °C before a saturated aqueous solution of NaHCO_3_ was added. The phases were separated and the aqueous layer was extracted with CH_2_Cl_2_ (3×). The combined organic layers were successively washed with an aqueous solution of KHSO_4_ (1 M), a saturated aqueous solution of NaHCO_3_ and brine, dried over MgSO_4_, and concentrated *in vacuo*. The crude product was purified via column chromatography (petroleum ether:EtOAc 20:1) providing TBS-ether **69a** (103 mg, 124 μmol, 84%) as a colorless oil. ^**1**^**H NMR (400 MHz, CDCl**_**3**_**)** δ = 5.95–5.85 (m, 1H), 5.31 (dq, *J* = 17.2, 1.5 Hz, 1H), 5.25–5.20 (m, 2H), 4.55 (dt, *J* = 5.8, 1.5 Hz, 2H), 4.52 (dd, *J* = 6.9, 3.4 Hz, 1H), 4.15 (d, *J* = 8.9 Hz, 1H), 3.99 (dd, *J* = 8.8, 5.7 Hz, 1H), 3.76–3.72 (m, 1H), 3.18–3.11 (m, 1H), 2.88–2.77 (m, 4H), 2.41–2.29 (m, 3H), 2.12–2.05 (m, 1H), 1.93–1.70 (m, 3H), 1.58 (d, *J* = 1.1 Hz, 3H), 1.14 (s, 3H), 1.06 (d, *J* = 6.8 Hz, 3H), 0.92 (s, 3H), 0.90 (s, 9H), 0.89–0.87 (m, 3H), 0.88 (s, 9H), 0.84 (s, 9H), 0.13 (s, 3H), 0.12 (s, 3H), 0.06 (s, 3H), 0.04 (s, 3H), 0.02 (s, 3H), —0.03 (s, 3H) ppm; ^**13**^**C{1H}-NMR (100 MHz, CDCl**_**3**_**)** δ = 215.3, 171.9, 135.6, 132.2, 131.0, 118.6, 80.5, 72.5, 72.2, 65.4, 54.2, 46.1, 44.1, 40.7, 39.9, 37.9, 30.5, 30.1, 26.2, 26.2, 26.1, 26.0, 21.6, 18.5, 18.4, 18.4, 18.4, 16.1, 15.0, 12.0, −4.0, −4.1, −4.3, −4.3, −4.3, −4.9 ppm; **HRMS** (ESI) *m*/*z*: [M + Na]^+^ calcd for C_42_H_82_O_6_S_2_Si_3_Na 853.4758; found 853.4755; **[α]**_**D**_^**22.7**^ = −20.6 (*c* = 0.34, CHCl_3_); **R**_*f*_ = 0.24 (petroleum ether:EtOAc 19:1).

#### Allyl (3*R*,6*S*,7*S*,10*S*,11*S*,*E*)-3,7,11-Tris((*tert*-butyldimethylsilyl)oxy)-12-(1,3-dithian-2-yl)-4,4,6,8,10-pentamethyl-5-oxododec-8-enoate (**69b**)

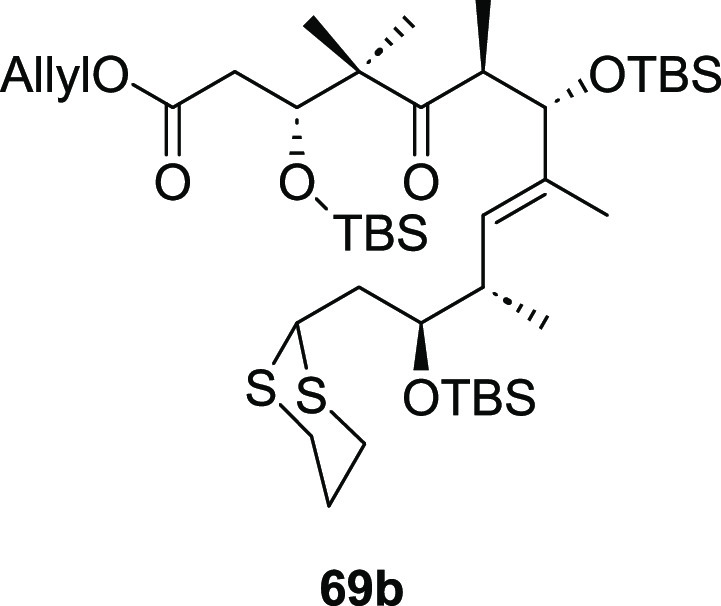



2,6-Lutidine (53 μL, 458 μmol, 3.00 equiv) and *tert*-butyldimethylsilyl trifluoromethanesulfonate (53 μL, 229 μmol, 1.50 equiv) were added successively to a solution of alcohol **23b** (109 mg, 153 μmol, 1.00 equiv) in CH_2_Cl_2_ (1.5 mL) at −78 °C. The reaction mixture was stirred for 10 min at −78 °C and for 40 min at 0 °C before a saturated aqueous solution of NaHCO_3_ was added. The phases were separated and the aqueous layer was extracted with CH_2_Cl_2_ (3×). The combined organic layers were successively washed with an aqueous solution of KHSO_4_ (1 M), a saturated aqueous solution of NaHCO_3_ and brine, dried over MgSO_4_, and concentrated *in vacuo*. The crude product was purified via column chromatography (petroleum ether:EtOAc 20:1) providing TBS-ether **69b** (113 mg, 136 μmol, 89%) as a colorless oil. ^**1**^**H NMR (400 MHz, CDCl**_**3**_**)** δ = 5.96–5.86 (m, 1H), 5.32 (dq, *J* = 17.2, 1.5 Hz, 1H), 5.24–5.21 (m, 1H), 5.15 (d, *J* = 9.6 Hz, 1H), 4.58–4.56 (m, 2H), 4.30 (dd, *J* = 6.8, 3.1 Hz, 1H), 4.16 (d, *J* = 8.8 Hz, 1H), 4.03 (dd, *J* = 9.6, 5.1 Hz, 1H), 3.81–3.77 (m, 1H), 3.18–3.10 (m, 1H), 2.90–2.73 (m, 4H), 2.48 (dd, *J* = 16.4, 3.1 Hz, 1H), 2.44–2.38 (m, 1H), 2.27 (dd, *J* = 16.4, 6.8 Hz, 1H), 2.13–2.06 (m, 1H), 1.92–1.71 (m, 3H), 1.59 (d, *J* = 1.2 Hz, 3H), 1.19 (s, 3H), 1.06 (d, *J* = 6.6 Hz, 3H), 0.93 (s, 3H), 0.90 (s, 9H), 0.88 (s, 9H), 0.87 (s, 9H), 0.83 (d, *J* = 6.8 Hz, 3H), 0.12 (s, 3H), 0.09 (s, 3H), 0.08 (s, 3H), 0.05 (s, 3H), 0.00 (s, 6H) ppm; ^**13**^**C{1H}-NMR (100 MHz, CDCl**_**3**_**)** δ = 216.7, 171.9, 136.0, 132.2, 130.5, 118.5, 79.6, 74.3, 73.1, 65.4, 53.3, 46.8, 44.3, 40.6, 40.4, 38.1, 30.8, 30.2, 26.2, 26.1, 26.1, 26.0, 23.2, 19.4, 18.4 (3 different carbon atoms), 16.2, 15.7, 12.2, −3.8, −4.0, −4.1, −4.2, −4.6, −4.8 ppm; **HRMS** (ESI) *m*/*z*: [M + Na]^+^ calcd for C_42_H_82_O_6_S_2_Si_3_Na 853.4758; found 853.4753; **[α]**_**D**_^**23.1**^ = +10.53 (*c* = 0.34, CHCl_3_); **R**_*f*_ = 0.39 (petroleum ether:EtOAc 19:1).

#### Allyl (3*R*,6*R*,7*R*,10*S*,11*S*,*E*)-3,7,11-Tris((*tert*-butyldimethylsilyl)oxy)-4,4,6,8,10-pentamethyl-5,13-dioxotridec-8-enoate (**24a**)

The glassware was neither dried nor purged with inert gas prior to usage. Calcium carbonate (48.0 mg, 481 μmol, 20.0 equiv) and iodomethane (30 μL, 481 μmol, 20.0 equiv) were added successively to a solution of TBS-ether **69a** (20.0 mg, 24.1 μmol, 1.00 equiv) in a mixture of acetonitrile (0.54 mL) and water (0.06 mL) at room temperature. The reaction mixture was stirred for 49.5 h at 45 °C before the solids were filtered by using Celite (Et_2_O). The filtrate was washed with water (2×) and brine, dried over Na_2_SO_4_, and concentrated *in vacuo*. Aldehyde **24a** (17.0 mg, 22.9 μmol, 95%) was obtained as a colorless oil and was used in the next reaction without detailed characterization.

#### Allyl (3*R*,6*S*,7*S*,10*S*,11*S*,*E*)-3,7,11-Tris((*tert*-butyldimethylsilyl)oxy)-4,4,6,8,10-pentamethyl-5,13-dioxotridec-8-enoate (**24b**)

The glassware was neither dried nor purged with inert gas prior to usage. Calcium carbonate (111 mg, 1.11 mmol, 40.0 equiv) and iodomethane (34 μL, 553 μmol, 20.0 equiv) were added successively to a solution of TBS-ether **69b** (23.0 mg, 27.6 μmol, 1.00 equiv) in a mixture of acetonitrile (0.66 mL) and water (0.07 mL) at room temperature. The reaction mixture was stirred for 24.5 h at 45 °C before the solids were filtered by using Celite (Et_2_O). The filtrate was washed with water (2×) and brine, dried over Na_2_SO_4_ and concentrated *in vacuo*. Aldehyde **24b** (18.4 mg, 24.4 μmol, 90%) was obtained as a colorless oil and was used in the next reaction without detailed characterization.

#### (1*R*,*E*)-1-((4*S*)-4-(((*tert*-Butyldimethylsilyl)oxy)(methoxy)methyl)-2,2-dimethyl-1,3-dioxolan-4-yl)but-2-en-1-ol (**70**)

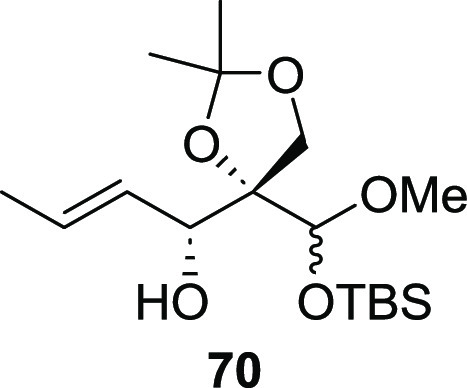



Borane-tetrahydrofuran (1 M in THF, 2.8 mL, 2.80 mmol, 1.00 equiv) was added dropwise to a suspension of *N*-tosyl-valine^16^ (0.84 g, 3.09 mmol, 1.10 equiv) in CH_2_Cl_2_ (18.0 mL) at 0 °C. The reaction mixture was stirred for 5 min at 0 °C and 1 h at room temperature before it was cooled to −78 °C. Crotonaldehyde (**26**) (0.23 mL, 2.81 mmol, 1.00 equiv) was added dropwise followed by the addition of a solution of ketene acetal **27** (1.00 g, 3.65 mmol, 1.30 equiv) in CH_2_Cl_2_ (7.0 mL). The reaction mixture was stirred for 2.5 h at −78 °C before an aqueous solution of phosphate buffer (pH = 7) was added. The phases were separated and the aqueous layer was extracted with MTBE (3×). The combined organic layers were washed with brine, dried over MgSO_4_, and concentrated *in vacuo*. The crude product was purified via column chromatography (petroleum ether:EtOAc 20:1 → 10:1) providing alcohol **70** (0.60 g, 1.74 mmol, 62%) as a colorless oil. Alcohol **70** was used in the next reaction without detailed characterization.

#### (*S*)-4-((*R*,*E*)-1-((*tert*-Butyldimethylsilyl)oxy)but-2-en-1-yl)-2,2-dimethyl-1,3-dioxolane-4-carbaldehyde (**28**)

Sodium bis(trimethylsilyl)amide (2 M in THF, 0.87 mL, 1.74 mmol, 1.00 equiv) was added dropwise to a solution of alcohol **70** (600 mg, 1.73 mmol, 1.00 equiv) in THF (57 mL) at −78 °C. The reaction mixture was stirred for 10 min at −78 °C and for 50 min at 0 °C before a saturated aqueous solution of NH_4_Cl was added. The phases were separated and the aqueous layer was extracted with MTBE (3×). The combined organic layers were washed with a saturated aqueous solution of NaHCO_3_ and brine, dried over MgSO_4_, and concentrated *in vacuo*. The crude product was purified via column chromatography (petroleum ether:EtOAc 40:1) providing aldehyde **28** (353 mg, 1.12 mmol, 65%, dr = 12:1) as a colorless oil. Analytical data are given for an enriched sample of aldehyde (dr ≥95:5, determined by NMR) **28**. ^**1**^**H NMR (400 MHz, CDCl**_**3**_**)** δ = 9.77 (d, *J* = 0.6 Hz, 1H), 5.72–5.63 (m, 1H), 5.51 (ddq, *J* = 15.4, 7.6, 1.5 Hz, 1H), 4.23 (d, *J* = 7.6 Hz, 1H), 4.22 (d, *J* = 8.8 Hz, 1H), 3.83 (dd, *J* = 8.8, 0.6 Hz, 1H), 1.72 (dd, *J* = 6.3, 1.3 Hz, 3H), 1.42 (s, 3H), 1.40 (s, 3H), 0.86 (s, 9H), 0.04 (s, 3H), 0.01 (s, 3H) ppm; ^**13**^**C{1H}-NMR (100 MHz, CDCl**_**3**_**)** δ = 203.4, 130.2, 128.9, 111.7, 89.4, 76.2, 67.8, 26.7, 26.2, 25.9, 18.2, 17.9, – 3.8, – 4.8 ppm; **HRMS** (ESI) *m*/*z*: [M + Na]^+^ calcd for C_16_H_30_O_4_SiNa 337.1811; found 337.1814; **[α]**_**D**_^**21.7**^ = −8.33 (*c* = 0.60, CHCl_3_); **R**_*f*_ = 0.31 (petroleum ether:EtOAc 19:1).

#### 1-((*R*)-4-((*R*,*E*)-1-((*tert*-Butyldimethylsilyl)oxy)but-2-en-1-yl)-2,2-dimethyl-1,3-dioxolan-4-yl)propan-1-ol (**71**)

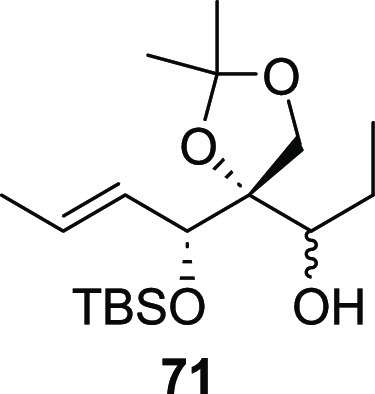



A suspension of aldehyde **28** (185 mg, 0.59 mmol, 1.00 equiv) and anhydrous cerium(III)-chloride (435 mg, 1.77 mmol, 3.00 equiv) in THF (2.1 mL) was stirred for 1 h before ethylmagnesium bromide (1 M in THF, 0.82 mL, 0.82 mmol, 1.40 equiv) was added. The reaction mixture was stirred for 1 h at 0 °C before a saturated aqueous solution of NH_4_Cl was added. The phases were separated and the aqueous layer was extracted with MTBE (3×). The combined organic layers were washed with a saturated aqueous solution of NaHCO_3_ and brine, dried over MgSO_4_, and concentrated *in vacuo*. The crude product was purified via column chromatography (petroleum ether:EtOAc 10:1) providing alcohol **71** (158 mg, 0.46 mmol, 78%) as a colorless oil. Alcohol **71** was used in the next reaction without detailed characterization.

#### 1-((*S*)-4-((*R*,*E*)-1-((*tert*-Butyldimethylsilyl)oxy)but-2-en-1-yl)-2,2-dimethyl-1,3-dioxolan-4-yl)propan-1-one (**25**)

A suspension of alcohol **71** (152 mg, 0.44 mmol, 1.00 equiv), *N*-methylmorpholine *N*-oxide (155 mg, 1.32 mmol, 3.00 equiv) and activated molecular sieves (4 Å, powdered, 390 mg) in CH_2_Cl_2_ (4.4 mL) was stirred for 30 min at room temperature before tetrapropylammonium perruthenate (8.0 mg, 22.8 μmol, 0.05 equiv) was added. The reaction mixture was stirred for 4.5 h before the solvent was removed *in vacuo*. The crude product was purified via column chromatography (petroleum ether:EtOAc 20:1) providing ketone **25** (128 mg, 0.37 mmol, 85%) as a colorless oil. ^**1**^**H NMR (400 MHz, CDCl**_**3**_**)** δ = 5.66–5.57 (m, 1H), 5.51–5.44 (m, 1H), 4.21 (d, *J* = 8.1 Hz, 1H), 4.15 (d, *J* = 8.8 Hz, 1H), 3.78 (d, *J* = 8.8 Hz, 1H), 2.83–2.64 (m, 2H), 1.72 (dd, *J* = 6.3, 1.4 Hz, 3H), 1.41 (s, 3H), 1.40 (s, 3H), 1.01 (t, *J* = 7.2 Hz, 3H), 0.84 (s, 9H), −0.02 (s, 3H), −0.02 (s, 3H) ppm; ^**13**^**C{1H}-NMR (100 MHz, CDCl**_**3**_**)** δ = 215.4, 129.8, 129.7, 111.4, 92.1, 77.3, 69.4, 34.0, 26.4, 26.1, 26.0, 18.3, 17.9, 7.1, −3.9, −4.7 ppm; **HRMS** (ESI) *m*/*z*: [M + Na]^+^ calcd for C_18_H_34_O_4_SiNa 365.2124; found 365.2126; **[α]**_**D**_^**20.0**^ = −15.3 (*c* = 1.00, CHCl_3_); **R**_*f*_ = 0.29 (petroleum ether:EtOAc 19:1).

#### (4*S*,5*R*,*E*)-4,5-Dihydroxy-4-(hydroxymethyl)oct-6-en-3-one (**72**)

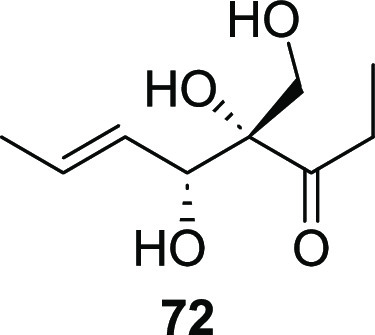



The glassware was neither dried nor purged with inert gas prior to usage. Trifluoroacetic acid (3.3 mL, 42.6 mmol, 76.0 equiv) was added to a solution of ketone **25** (190 mg, 0.56 mmol, 1.00 equiv) in THF (17.0 mL) and water (8.5 mL) at room temperature. The reaction mixture was stirred for 14 h at 50 °C before it was cooled to 0 °C. Triethylamine (8.0 mL, 57.7 mmol, 100 equiv) was added and stirring was continued for 10 min before EtOAc and a saturated aqueous solution of NaHCO_3_ were added. The phases were separated and the aqueous layer was extracted with EtOAc (4×). The combined organic layers were washed with brine, dried over MgSO_4_, and concentrated *in vacuo*. The crude product was purified via column chromatography (EtOAc) providing triol **72** (82.0 mg, 0.44 mmol, 78%) as a colorless oil. Triol **72** was used in the next reaction without detailed characterization.

#### (4*S*,5*R*,*E*)-5-((*tert*-Butyldimethylsilyl)oxy)-4-(((*tert*-butyldimethylsilyl)oxy)methyl)-4-hydroxyoct-6-en-3-one (**30**)

2,6-Lutidine (0.20 mL, 1.70 mmol, 4.00 equiv) and *tert*-butyldimethylsilyl trifluoromethanesulfonate (0.20 mL, 0.85 mmol, 1.50 equiv) were added successively to a solution of triol **72** (80.0 mg, 0.43 mmol, 1.00 equiv) in CH_2_Cl_2_ (2.1 mL) at −78 °C. The reaction mixture was stirred for 30 min at −78 °C before a saturated aqueous solution of NaHCO_3_ was added. The phases were separated and the aqueous layer was extracted with CH_2_Cl_2_ (3×). The combined organic layers were successively washed with an aqueous solution of KHSO_4_ (1 M), a saturated aqueous solution of NaHCO_3_ and brine, dried over MgSO_4_, and concentrated *in vacuo*. The crude product was purified via column chromatography (petroleum ether:EtOAc 20:1) providing TBS-ether **30** (135 mg, 0.32 mmol, 75%) as a colorless oil. ^**1**^**H NMR (400 MHz, CDCl**_**3**_**)** δ = 5.68–5.60 (m, 1H), 5.41 (ddq, *J* = 15.4, 8.1, 1.6 Hz, 1H), 4.30 (d, *J* = 8.1 Hz, 1H), 3.86 (dd, *J* = 10.3, 0.6 Hz, 1H), 3.79 (d, *J* = 0.6 Hz, 1H), 3.44 (d, *J* = 10.3 Hz, 1H), 2.74–2.58 (m, 2H), 1.69 (dd, *J* = 6.3, 1.6 Hz, 3H), 1.04 (t, *J* = 7.2 Hz, 3H), 0.84 (s, 9H), 0.83 (s, 9H), 0.01 (s, 3H), 0.00 (s, 6H), – 0.02 (s, 3H)ppm; ^**13**^**C{1H}-NMR (100 MHz, CDCl**_**3**_**)** δ = 213.5, 129.7, 129.2, 84.7, 76.2, 66.6, 32.6, 25.9, 25.9, 18.3, 18.1, 17.8, 7.3, −3.6, −4.9, −5.3, −5.6 ppm; **HRMS** (ESI) *m*/*z*: [M + Na]^+^ calcd for C_21_H_44_O_4_Si_2_Na 439.2676; found: 439.2679; **[α]**_**D**_^**22.3**^ = −11.2 (*c* = 1.00, CHCl_3_); **R**_*f*_ = 0.42 (petroleum ether:EtOAc 19:1).

#### (4*S*,5*R*,*E*)-5-((*tert*-Butyldimethylsilyl)oxy)-4-(((*tert*-butyldimethylsilyl)oxy)methyl)-4-((trimethylsilyl)oxy)oct-6-en-3-one (**31**)

2,6-Lutidine (58 μL, 500 μmol, 2.80 equiv) and trimethylsilyl trifluoromethanesulfonate (45 μL, 249 μmol, 1.40 equiv) were added successively to a solution of TBS-ether **30** (74.0 mg, 178 μmol, 1.00 equiv) in CH_2_Cl_2_ (1.8 mL) at −78 °C. The reaction mixture was stirred for 30 min at −78 °C and for 2 h at 0 °C before additional 2,6-lutidine (30 μL, 258 μmol, 1.45 equiv) and trimethylsilyl trifluoromethanesulfonate (23 μL, 127 μmol, 0.72 equiv) were added. The reaction mixture was stirred for 1 h at 0 °C before a saturated aqueous solution of NaHCO_3_ was added. The phases were separated and the aqueous layer was extracted with CH_2_Cl_2_ (3×). The combined organic layers were successively washed with an aqueous solution of KHSO_4_ (1 M), a saturated aqueous solution of NaHCO_3_ and brine, dried over MgSO_4_, and concentrated *in vacuo*. The crude product was purified via column chromatography (petroleum ether:EtOAc 40:1) providing TMS-ether **31** (74.0 mg, 152 μmol, 85%) as a colorless oil. ^**1**^**H NMR (400 MHz, CDCl**_**3**_**)** δ = 5.60–5.51 (m, 1H), 5.37 (ddq, *J* = 15.5, 8.0, 1.6 Hz, 1H), 4.17 (d, *J* = 8.0 Hz, 1H), 3.85 (d, *J* = 10.2 Hz, 1H), 3.36 (d, *J* = 10.2 Hz, 1H), 2.73–2.50 (m, 2H), 1.70 (dd, *J* = 6.3, 1.6 Hz, 3H), 0.97 (t, *J* = 7.2 Hz, 3H), 0.85 (s, 9H), 0.82 (s, 9H), 0.16 (s, 9H), 0.01 (s, 3H), −0.01 (s, 3H), −0.06 (s, 6H) ppm; ^**13**^**C{1H}-NMR (100 MHz, CDCl**_**3**_**)** δ = 215.0, 130.7, 128.7, 89.4, 77.3, 67.2, 33.9, 26.0, 25.9, 18.5, 18.1, 17.9, 7.2, 2.7, −3.7, −4.9, −5.3, −5.5 ppm; **HRMS** (ESI) *m*/*z*: [M + Na]^+^ calcd for C_24_H_52_O_4_Si_3_Na 511.3071; found 511.3078; **[α]**_**D**_^**22.5**^ = +12.0 (*c* = 1.00, CHCl_3_); **R**_*f*_ = 0.72 (petroleum ether:EtOAc 19:1).

#### (*S*)-4-Benzyl-3-((4*S*,5*R*,*E*)-5-((*tert*-butyldimethylsilyl)oxy)-2,4-dimethylhex-2-enoyl)oxazolidin-2-one (**73**)

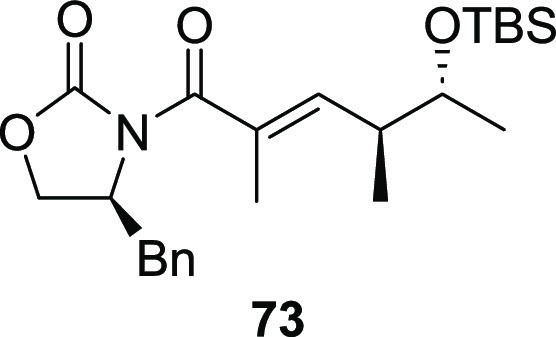



2,6-Lutidine (5.4 mL, 45.9 mmol, 2.60 equiv) and *tert*-butyldimethylsilyl trifluoromethanesulfonate (5.3 mL, 22.9 mmol, 1.30 equiv) were added successively to a solution of alcohol **35**(21) (5.60 g, 17.6 mmol, 1.00 equiv) in CH_2_Cl_2_ (60 mL) at −78 °C. The reaction mixture was stirred for 15 min at −78 °C and for 20 min at 0 °C before a saturated aqueous solution of NaHCO_3_ was added. The phases were separated and the aqueous layer was extracted with CH_2_Cl_2_ (3×). The combined organic layers were successively washed with an aqueous solution of KHSO_4_ (1 M), a saturated aqueous solution of NaHCO_3_ and brine, dried over MgSO_4_, and concentrated *in vacuo*. The crude product was purified via column chromatography (petroleum ether:EtOAc 4:1) providing TBS-ether **73** (7.37 g, 17.1 mmol, 97%) as a colorless oil. ^**1**^**H NMR (400 MHz, CDCl**_**3**_**)** δ = 7.34–7.31 (m, 2H), 7.28–7.26 (m, 1H), 7.21–7.19 (m, 2H), 6.00 (dq, *J* = 10.0, 1.3 Hz, 1H), 4.71–4.67 (m, 1H), 4.23 (dd, *J* = 8.9, 8.2 Hz, 1H), 4.14 (dd, *J* = 8.9, 5.3 Hz, 1H), 3.82–3.78 (m, 1H), 3.35 (dd, *J* = 13.6, 3.4 Hz, 1H), 2.83 (dd, *J* = 13.6, 9.4 Hz, 1H), 2.57–2.51 (m, 1H), 1.91 (d, *J* = 1.5 Hz, 3H), 1.11 (d, *J* = 6.5 Hz, 3H), 1.02 (d, *J* = 7.1 Hz, 3H), 0.89 (s, 9H), 0.05 (s, 6H) ppm; ^**13**^**C{1H}-NMR (100 MHz, CDCl**_**3**_**)** δ = 172.2, 153.2, 141.5, 135.4, 130.6, 129.6, 129.1, 127.5, 71.4, 66.5, 55.7, 40.5, 37.7, 26.0, 21.3, 18.2, 15.8, 13.8, – 4.2, – 4.7 ppm; **HRMS** (ESI) *m*/*z*: [M + Na]^+^ calcd for C_24_H_37_NO_4_SiNa 454.2390; found 454.2388; **[α]**_**D**_^**25.4**^ = +33.8 (*c* = 1.00, CHCl_3_); **R**_*f*_ = 0.65 (petroleum ether:EtOAc 4:1).

#### (4*S*,5*R*,*E*)-5-((*tert*-Butyldimethylsilyl)oxy)-2,4-dimethylhex-2-en-1-ol (**74**)

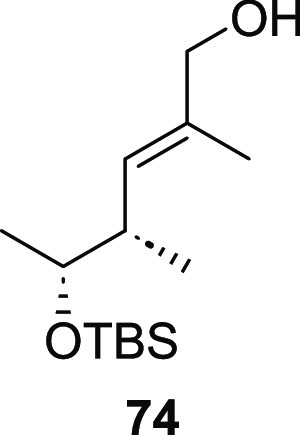



Sodium borohydride (4.11 g, 109 mmol, 5.00 equiv) was dissolved in water (44 mL) and the resulting solution was added dropwise to a solution of TBS-ether **73** (9.39 g, 21.8 mmol, 1.00 equiv) in THF (88 mL) at 0 °C. The reaction mixture was stirred for 10 min at 0 °C and for 15 h at room temperature before a saturated aqueous solution of NH_4_Cl was added. The phases were separated and the aqueous layer was extracted with MTBE (3×). The combined organic layers were successively washed with a saturated aqueous solution of NaHCO_3_ and brine, dried over MgSO_4_, and concentrated *in vacuo*. The crude product was purified via column chromatography (petroleum ether:EtOAc 4:1) providing alcohol **74** (5.09 g, 19.7 mmol, 90%) as a colorless oil. ^**1**^**H NMR (400 MHz, CDCl**_**3**_**)** δ = 5.32–5.29 (m, 1H), 4.01 (s, 2H), 3.71–3.65 (m, 1H), 2.45–2.36 (m, 1H), 1.67 (d, *J* = 1.1 Hz, 3H), 1.26 (bs, 1H), 1.04 (d, *J* = 6.3 Hz, 3H), 0.93 (d, *J* = 7.0 Hz, 3H), 0.88 (s, 9H), 0.03 (s, 3H), 0.03 (s, 3H) ppm; ^**13**^**C{1H}-NMR (100 MHz, CDCl**_**3**_**)** δ = 134.6, 129.3, 71.9, 69.4, 39.7, 26.0, 21.0, 18.2, 16.6, 14.1, −4.2, −4.7 ppm; **HRMS** (ESI) *m*/*z*: [M + Na]^+^ calcd for C_14_H_30_O_2_SiNa 281.1913; found 281.1915; **[α]**_**D**_^**25.8**^ = −7.6 (*c* = 1.00, CHCl_3_); **R**_*f*_ = 0.62 (petroleum ether:EtOAc 4:1). The anayltical data are in accordance with the literature.^31^

#### (4*S*,5*R*,*E*)-5-((*tert*-Butyldimethylsilyl)oxy)-2,4-dimethylhex-2-en-1-yl Pivalate (**36**)

Pyridine (8.0 mL, 98.3 mmol, 5.00 equiv) and pivaloyl chloride (2.9 mL, 23.6 mmol, 1.20 equiv) were added successively to a solution of alcohol **74** (5.08 g, 19.7 mmol, 1.00 equiv) in CH_2_Cl_2_ (66 mL) at 0 °C. The reaction mixture was stirred for 15 min at 0 °C and for 2 h at room temperature before water was added. The phases were separated and the aqueous layer was extracted with CH_2_Cl_2_ (3×). The combined organic layers were successively washed with an aqueous solution of KHSO_4_ (1 M), a saturated aqueous solution of NaHCO_3_ and brine, dried over MgSO_4_, and concentrated *in vacuo*. The crude product was purified via column chromatography (petroleum ether:EtOAc 20:1) providing pivalate **36** (6.40 g, 18.7 mmol, 95%) as a colorless oil. ^**1**^**H NMR (400 MHz, CDCl**_**3**_**)** δ = 5.35–5.32 (m, 1H), 4.48–4.41 (m, 2H), 3.69–3.64 (m, 1H), 2.44–2.35 (m, 1H), 1.63 (d, *J* = 1.3 Hz, 3H), 1.21 (s, 9H), 1.04 (d, *J* = 6.2 Hz, 3H), 0.93 (d, *J* = 6.9 Hz, 3H), 0.87 (s, 9H), 0.03 (s, 3H), 0.02 (s, 3H) ppm; ^**13**^**C{1H}-NMR (100 MHz, CDCl**_**3**_**)** δ = 178.5, 131.3, 130.0, 71.9, 69.9, 39.8, 39.0, 27.4, 26.0, 21.2, 18.2, 16.8, 14.2, – 4.2, – 4.6 ppm; **HRMS** (ESI) *m*/*z*: [M + Na]^+^ calcd for C_19_H_38_O_3_SiNa 365.2488; found 365.2488; **[α]**_**D**_^**23.2**^ = −3.0 (*c* = 1.00, CHCl_3_); **R**_*f*_ = 0.5 (petroleum ether:EtOAc 19:1).

#### (4*S*,5*R*,*E*)-5-Hydroxy-2,4-dimethylhex-2-en-1-yl Pivalate (**75**)

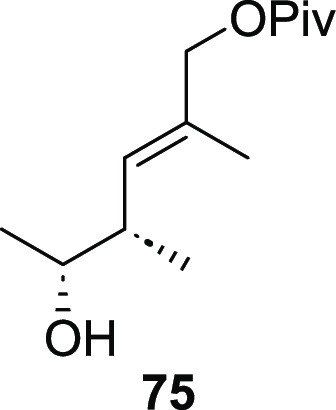



Tetrabutylammonium fluoride (1 m in THF, 75 mL, 75.0 mmol, 4.00 equiv) was added to a solution of pivalate **36** (6.40 g, 18.7 mmol, 1.00 equiv) in THF (93 mL) at room temperature. The reaction mixture was stirred for 29 h before the subsequent addition of water (100 mL) and MTBE (100 mL). The phases were separated and the aqueous layer was extracted with MTBE (3×). The combined organic layers were washed with brine, dried over MgSO_4_ and concentrated *in vacuo*. The crude product was purified via column chromatography (petroleum ether:EtOAc 5:1) providing alcohol **75** (3.53 g, 15.4 mmol, 83%) as a colorless oil. ^**1**^**H NMR (400 MHz, CDCl**_**3**_**)** δ = 5.31–5.29 (m, 1H), 4.49 (d, *J* = 12.4 Hz, 1H), 4.46 (d, *J* = 12.4 Hz, 1H), 3.54 (p, *J* = 6.4 Hz, 1H), 2.42–2.36 (m, 1H), 1.68 (d, *J* = 1.1 Hz, 3H), 1.65 (bs, 1H), 1.21 (s, 9H), 1.17 (d, *J* = 6.4 Hz, 3H), 0.96 (d, *J* = 6.8 Hz, 3H) ppm; ^**13**^**C{1H}-NMR (100 MHz, CDCl**_**3**_**)** δ = 178.5, 132.8, 130.9, 71.8, 69.8, 40.4, 39.0, 27.4, 20.3, 16.8, 14.5 ppm; **HRMS** (ESI) *m*/*z*: [M + Na]^+^: calcd for C_13_H_24_O_3_Na 251.1623; found 251.1623; **[α]**_**D**_^**25.8**^ = −36.0 (*c* = 1.00, CHCl_3_); **R**_*f*_ = 0.37 (petroleum ether:EtOAc 5:1).

#### (*S*,*E*)-2,4-Dimethyl-5-oxohex-2-en-1-yl pivalate (**15**)

Dimethyl sulfoxide (5.5 mL, 77.3 mmol, 5.00 equiv) was added to a solution of oxalyl chloride (4.0 mL, 46.4 mmol, 3.00 equiv) in CH_2_Cl_2_ (115 mL) at −78 °C. The reaction mixture was stirred for 15 min before a solution of alcohol **75** (3.53 g, 15.4 mmol, 1.00 equiv) in CH_2_Cl_2_ (40 mL) was added dropwise over the course of 10 min. The reaction mixture was stirred for 1 h before triethylamine (15.0 mL, 108 mmol, 7.00 equiv) was added. The reaction mixture was stirred for 1 h at −78 °C and for 1.5 h at 0 °C before a saturated aqueous solution of NH_4_Cl was added. The phases were separated and the aqueous layer was extracted with CH_2_Cl_2_ (3×). The combined organic layers were successively washed with a saturated aqueous solution of NaHCO_3_ and brine, dried over MgSO_4_, and concentrated *in vacuo*. The crude product was purified via column chromatography (petroleum ether:EtOAc 10:1) providing eastern fragment **15** (3.24 g, 14.3 mmol, 92%) as a colorless oil. ^**1**^**H NMR (400 MHz, CDCl**_**3**_**)** δ = 5.39–5.37 (m, 1H), 4.51 (d, *J* = 12.5 Hz, 1H), 4.47 (d, *J* = 12.5 Hz, 1H), 3.45–3.38 (m, 1H), 2.13 (s, 3H), 1.75 (d, *J* = 1.1 Hz, 3H), 1.23 (s, 9H), 1.17 (d, *J* = 7.0 Hz, 3H) ppm; ^**13**^**C{1H}-NMR (100 MHz, CDCl**_**3**_**)** δ = 209.5, 178.3, 133.4, 127.3, 69.0, 46.8, 39.0, 28.0, 27.3, 16.4, 14.4 ppm; **HRMS** (ESI) *m*/*z*: [M + Na]^+^ calcd for C_13_H_22_O_3_Na 249.1467, found: 249.1467; **[α]**_**D**_^**24.8**^ = +199 (*c* = 1.00, CHCl_3_); **R**_*f*_ = 0.21 (petroleum ether:EtOAc 19:1), 0.42 (petroleum ether:EtOAc 9:1).

#### (2*R*,3*S*)-3-((4*R*)-4-(((*tert*-Butyldimethylsilyl)oxy)(methoxy)methyl)-2,2-dimethyl-1,3-dioxolan-4-yl)-3-((4-methoxybenzyl)oxy)-2-methylpropan-1-ol (**76**)

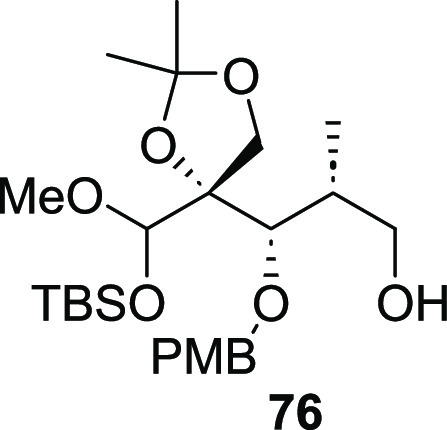



Diisobutylaluminum hydride (1 M in hexane, 72 mL, 72.0 mmol, 3.02 equiv) was added dropwise to a solution of PMP-acetal **38**(23) (11.5 g, 23.8 mmol, 1.00 equiv) in CH_2_Cl_2_ (87 mL) at −78 °C. The reaction mixture was stirred for 30 min at −78 °C and for 1 h at 0 °C before MeOH (20.0 mL) was added. The resulting slurry was diluted with MTBE (250 mL) and poured into a vigorously stirred solution of Rochelle salt (250 mL) at room temperature. Stirring was continued until two phases were formed (1 h). The phases were separated and the aqueous layer was extracted with MTBE (3×). The combined organic layers were washed with brine, dried over MgSO_4_, and concentrated *in vacuo*. The crude product was purified via column chromatography (petroleum ether:EtOAc 4:1) providing alcohol **76** (11.0 g, 22.7 mmol, 95%) as a colorless oil. ^**1**^**H NMR (400 MHz, CDCl**_**3**_**)** δ = 7.29–7.25 (m, 2H), 6.88–6.84 (m, 2H), 4.90 (d, *J* = 11.2 Hz, 1H), 4.70 (s, 1H), 4.57 (d, *J* = 11.2 Hz, 1H), 4.24 (d, *J* = 1.8 Hz, 1H), 4.12 (d, *J* = 8.8 Hz, 1H), 4.08 (d, *J* = 8.8 Hz, 1H), 3.80 (s, 3H), 3.52 (s, 3H), 3.50–3.43 (m, 2H), 2.35–2.26 (m, 1H), 1.50 (s, 3H), 1.44 (bs, 1H), 1.36 (s, 3H), 0.91 (s, 9H), 0.77 (d, *J* = 6.8 Hz, 3H), 0.16 (s, 3H), 0.16 (s, 3H) ppm; ^**13**^**C{1H}-NMR (100 MHz, CDCl**_**3**_**)** δ = 159.0, 131.6, 129.0, 113.8, 108.7, 102.4, 90.2, 77.1, 75.7, 67.0, 66.0, 58.1, 55.4, 36.8, 28.3, 26.0, 25.9, 18.2, 11.2, −4.0, −4.6 ppm; **HRMS** (ESI) *m*/*z*: [M + Na]^+^ calcd for C_25_H_44_O_7_SiNa 507.2754; found 507.2755; **[α]**_**D**_^**32.3**^ = −1.0 (*c* = 1.00, CHCl_3_); **R**_*f*_ = 0.35 (petroleum ether:EtOAc 4:1).

#### *(2R*,3*S*)-3-((4*R*)-4-(((*tert*-Butyldimethylsilyl)oxy)(methoxy)methyl)-2,2-dimethyl-1,3-dioxolan-4-yl)-3-((4-methoxybenzyl)oxy)-2-methylpropyl Pivalate (**77**)

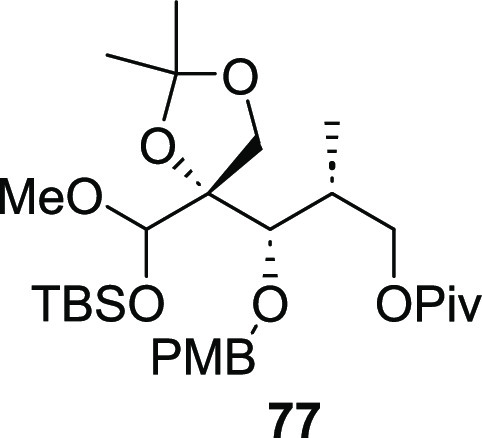



Pyridine (9.5 mL, 113 mmol, 5.00 equiv) and pivaloyl chloride (3.4 mL, 27.2 mmol, 1.20 equiv) were added successively to a solution of alcohol **76** (11.0 g, 22.7 mmol, 1.00 equiv) in CH_2_Cl_2_ (110 mL) at 0 °C. The reaction mixture was stirred for 15 min at 0 °C and for 2.25 h at room temperature before water was added. The phases were separated and the aqueous layer was extracted with CH_2_Cl_2_ (3 x). The combined organic layers were successively washed with an aqueous solution of KHSO_4_ (1 M), a saturated aqueous solution of NaHCO_3_ and brine, dried over MgSO_4_ and concentrated *in vacuo*. The crude product was purified via column chromatography (petroleum ether:EtOAc 10:1) providing pivalate **77** (12.5 g, 21.9 mmol, 96%) as a colorless oil. ^**1**^**H NMR (400 MHz, CDCl**_**3**_**)** δ = 7.27–7.24 (m, 2H), 6.88–6.84 (m, 2H), 4.90 (d, *J* = 10.8 Hz, 1H), 4.68 (s, 1H), 4.47 (d, *J* = 10.8 Hz, 1H), 4.18 (d, *J* = 1.5 Hz, 1H), 4.10 (d, *J* = 8.8 Hz, 1H), 4.07 (d, *J* = 8.8 Hz, 1H), 4.01 (dd, *J* = 10.6, 6.2 Hz, 1H), 3.87 (dd, *J* = 10.6, 9.1 Hz, 1H), 3.80 (s, 3H), 3.49 (s, 3H), 2.58–2.49 (m, 1H), 1.50 (s, 3H), 1.34 (s, 3H), 1.21 (s, 9H), 0.91 (s, 9H), 0.77 (d, *J* = 6.8 Hz, 3H), 0.14 (s, 6H) ppm; ^**13**^**C{1H}-NMR (100 MHz, CDCl**_**3**_**)** δ = 178.4, 159.0, 131.5, 128.9, 113.8, 108.6, 102.1, 90.2, 76.5, 75.9, 67.0, 66.0, 57.9, 55.4, 38.9, 34.0, 28.4, 27.4, 25.9, 25.9, 18.2, 11.1, −4.1, −4.6 ppm; **HRMS** (ESI) *m*/*z*: [M + Na]^+^ calcd for C_30_H_52_O_8_SiNa 591.3329; found 591.3330; **[α]**_**D**_^**32.4**^ = −12.0 (*c* = 1.00, CHCl_3_); **R**_*f*_ = 0.49 (petroleum ether:EtOAc 9:1).

#### (2*R*,3*S*)-3-((*R*)-4-Formyl-2,2-dimethyl-1,3-dioxolan-4-yl)-3-((4-methoxybenzyl)oxy)-2-methylpropyl Pivalate (**39**)

Tetrabutylammonium fluoride (1 M in THF, 39 mL, 39.0 mmol, 1.82 equiv) was added to a solution of pivalate **77** (12.2 g, 21.4 mmol, 1.00 equiv) in THF (68 mL) at 0 °C. The reaction mixture was stirred for 1.5 h before water was added. The mixture was extracted with MTBE (3×). The combined organic layers were washed with brine, dried over MgSO_4_, and concentrated *in vacuo*. The crude product was purified via column chromatography (petroleum ether:EtOAc 9:1) providing aldehyde **39** (8.97 g, 21.1 mmol, 99%) as a colorless oil. Aldehyde **39** was used in the next reaction without detailed characterization.

#### (2*R*,3*S*)-3-((*S*)-4-((*R*)-1-Hydroxyallyl)-2,2-dimethyl-1,3-dioxolan-4-yl)-3-((4-methoxybenzyl)oxy)-2-methylpropyl Pivalate (**78**)

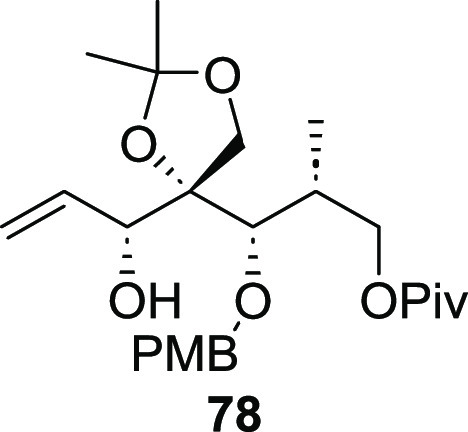



Vinylmagnesium bromide (**40**) (1 M in THF, 64 mL, 64.0 mmol, 3.02 equiv) was added dropwise over the course of 10 min to a solution of aldehyde **39** (8.97 g, 21.2 mmol, 1.00 equiv) in THF (144 mL) at −100 °C. The reaction mixture was stirred for 20 min before a saturated aqueous solution of NH_4_Cl was added. The obtained suspension was diluted with water, the phases were separated, and the aqueous layer was extracted with MTBE (3×). The combined organic layers were successively washed with a saturated aqueous solution of NaHCO_3_ and brine, dried over MgSO_4_ and concentrated *in vacuo*. The crude product was purified via column chromatography (petroleum ether:EtOAc 5:1) providing alcohol **78** (8.73 g, 19.4 mmol, 91%, dr = 8:1) as a colorless oil. Analytical data are given for a pure sample of the major diastereomer. ^**1**^**H NMR (400 MHz, CDCl**_**3**_**)** δ = 7.25–7.22 (m, 2H), 6.89–6.85 (m, 2H), 6.10 (ddd, *J* = 17.3, 10.6, 5.0 Hz, 1H), 5.38 (dt, *J* = 17.3, 1.7 Hz, 1H), 5.26 (dt, *J* = 10.6, 1.6 Hz, 1H), 4.76 (d, *J* = 10.8 Hz, 1H), 4.50 (d, *J* = 10.8 Hz, 1H), 4.23–4.19 (m, 1H), 4.12 (d, *J* = 8.9 Hz, 1H), 4.05–3.93 (m, 4H), 3.80 (s, 3H), 2.41–2.31 (m, 1H), 2.08 (d, *J* = 6.2 Hz, 1H), 1.47 (s, 3H), 1.39 (s, 3H), 1.22 (s, 9H), 0.87 (d, *J* = 7.0 Hz, 3H) ppm; ^**13**^**C{1H}-NMR (100 MHz, CDCl**_**3**_**)** δ = 178.5, 159.2, 136.5, 130.9, 128.9, 116.3, 113.9, 109.5, 88.4, 78.6, 75.7, 73.7, 67.1, 66.6, 55.4, 39.0, 34.1, 27.4, 27.2, 26.4, 11.3 ppm; **HRMS** (ESI) *m*/*z*: [M + Na]^+^ calcd for C_25_H_38_O_7_Na 473.2515; found 473.2517; **[α]**_**D**_^**32.5**^ = +20.0 (*c* = 1.00, CHCl_3_); **R**_*f*_ = 0.29 (petroleum ether:EtOAc 5:1).

#### (2*R*,3*S*)-3-((*R*)-2,2-Dimethyl-4-((*R*)-1-((triethylsilyl)oxy)allyl)-1,3-dioxolan-4-yl)-3-((4-methoxybenzyl)oxy)-2-methylpropyl Pivalate (**79**)

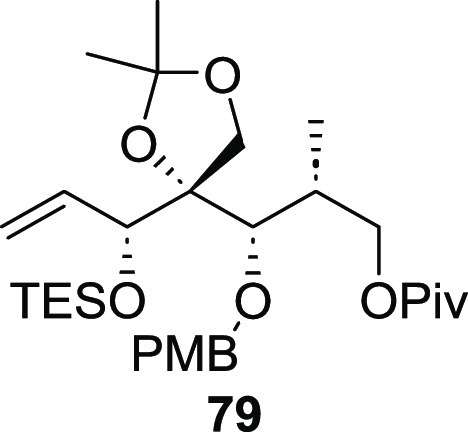



2,6-Lutidine (6.9 mL, 58.9 mmol, 3.00 equiv) and triethylsilyl trifluoromethanesulfonate (6.7 mL, 29.4 mmol, 1.50 equiv) were added successively to a solution of alcohol **78** (8.84 g, 19.6 mmol, 1.00 equiv) in CH_2_Cl_2_ (96 mL) at −78 °C. The reaction mixture was stirred for 10 min at −78 °C and for 20 min at 0 °C before a saturated aqueous solution of NaHCO_3_ was added. The phases were separated and the aqueous layer was extracted with CH_2_Cl_2_ (3×). The combined organic layers were successively washed with an aqueous solution of KHSO_4_ (1 M), a saturated aqueous solution of NaHCO_3_ and brine, dried over MgSO_4_, and concentrated *in vacuo*. The crude product was purified via column chromatography (petroleum ether:EtOAc 20:1) providing TES-ether **79** (10.2 g, 18.1 mmol, 92%, 9.33 g, 16.5 mmol, undesired diastereomer could be fully separated at this stage leading to 84% of the desired diastereomer) as a colorless oil. ^**1**^**H NMR (400 MHz, CDCl**_**3**_**)** δ = 7.24–7.21 (m, 2H), 6.87–6.83 (m, 2H), 6.08–6.00 (m, 1H), 5.33 (dt, *J* = 17.3, 1.6 Hz, 1H), 5.26 (dt, *J* = 10.6, 1.6 Hz, 1H), 4.86 (d, *J* = 10.7 Hz, 1H), 4.37 (d, *J* = 10.7 Hz, 1H), 4.24–4.22 (m, 1H), 4.17 (d, *J* = 8.6 Hz, 1H), 4.11 (d, *J* = 8.6 Hz, 1H), 3.97–3.87 (m, 3H), 3.79 (s, 3H), 2.55–2.46 (m, 1H), 1.49 (s, 3H), 1.35 (s, 3H), 1.21 (s, 9H), 0.96 (t, *J* = 7.9 Hz, 9H), 0.76 (d, *J* = 6.8 Hz, 3H), 0.66–0.60 (m, 6H) ppm; ^**13**^**C{1H}-NMR (100 MHz, CDCl**_**3**_**)** δ = 178.3, 159.0, 136.4, 131.4, 128.8, 117.2, 113.8, 108.5, 89.1, 77.4, 75.8, 75.5, 67.0, 65.7, 55.4, 38.9, 33.6, 28.6, 27.4, 26.0, 10.9, 6.9, 5.0 ppm; **HRMS** (ESI) *m*/*z*: [M + Na]^+^ calcd for C_31_H_52_O_7_SiNa 587.3380; found 587.3381; **[α]**_**D**_^**32.5**^ = +10.0 (*c* = 1.00, CHCl_3_); **R**_*f*_ = 0.23 (petroleum ether:EtOAc 19:1).

#### (2*R*,3*S*)-3-((*R*)-2,2-Dimethyl-4-((*S*)-2-oxo-1-((triethylsilyl)oxy)ethyl)-1,3-dioxolan-4-yl)-3-((4-methoxybenzyl)oxy)-2-methylpropyl Pivalate (**41**)

The glassware was neither dried nor purged with inert gas prior to usage. Osmium tetroxide (2.5% (w/w) in ^*t*^BuOH, 2.7 mL, 0.21 mmol, 0.04 equiv) was added to a solution of TES-ether **79** (3.00 g, 5.31 mmol, 1.00 equiv), 2,6-lutidine (2.5 mL, 21.2 mmol, 4.00 equiv), and *N*-methylmorpholine *N*-oxide (1.87 g, 15.9 mmol, 3.00 equiv) in acetone (22.0 mL) and water (4.4 mL) at room temperature. The reaction mixture was stirred for 20.5 h before (diacetoxyiodo)benzene (2.57 g, 7.97 mmol, 1.50 equiv) was added. The reaction mixture was stirred for 1.75 h before a saturated aqueous solution of Na_2_S_2_O_3_ (30 mL) was added and stirring was continued for 3 h before the reaction mixture was diluted with MTBE. The phases were separated and the aqueous layer was extracted with MTBE (3×). The combined organic layers were successively washed with an aqueous solution of CuSO_4_ (1 M) and brine, dried over MgSO_4_, and concentrated *in vacuo*. The crude product was purified via column chromatography (petroleum ether:EtOAc 20:1) providing aldehyde **41** (2.59 g, 4.57 mmol, 86%) as a colorless oil. Aldehyde **41** was used in the next reaction without detailed characterization.

#### (*S*,*Z*)-2-Methylpent-3-en-1-ol (**80**)

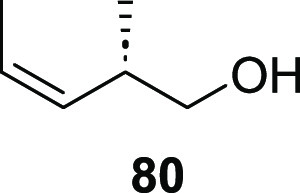



Pyridinium *p*-toluenesulfonate (10.4 g, 41.4 mmol, 0.41 equiv) was added to a solution of alkene **42**(26) (35.0 g, 102 mmol, 1.00 equiv) in CH_2_Cl_2_ (120 mL) and MeOH (60 mL) at 0 °C. The reaction mixture was stirred for 23 h at room temperature before a saturated aqueous solution of NaHCO_3_ was added. The phases were separated and the aqueous layer was extracted with Et_2_O (3×). The combined organic layers were washed with brine, dried over MgSO_4_, and concentrated under reduced pressure (*p*_min_ = 900 mbar). The crude product was purified via column chromatography (pentane:Et_2_O 4:1 → 2:1) providing alcohol **80** (9.76 g, 97.4 mmol, 96%, mixture with Et_2_O, 16.5 g in total) as a colorless liquid. Due to the volatility of alcohol **80**, the Et_2_O was not fully removed and the product therefore not characterized in detail.

#### (*S*,*Z*)-5-((2-Methylpent-3-en-1-yl)thio)-1-phenyl-1*H*-tetrazole (**81**)

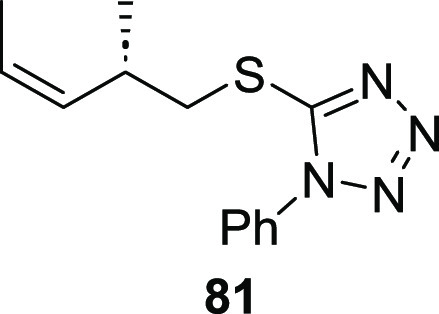



Diisopropyl azodicarboxylate (23.0 mL, 117 mmol, 1.20 equiv) was added over the course of 10 min to a solution of alcohol **80** (9.75 g, 97.3 mmol, 96%, mixture with Et_2_O, 16.5 g in total), 1-phenyl-1*H*-tetrazole-5-thiol (**27**) (20.8 g, 117 mmol, 1.20 equiv), and triphenylphosphine (30.6 g, 117 mmol, 1.20 equiv) in THF (320 mL) at 0 °C. The reaction mixture was stirred for 10 min at 0 °C and for 100 min at room temperature before a saturated aqueous solution of NH_4_Cl was added. The phases were separated and the aqueous layer was extracted with MTBE (3×). The combined organic layers were successively washed with a saturated aqueous solution of NaHCO_3_ and brine, dried over MgSO_4_, and concentrated *in vacuo*. The crude product was loaded onto silica and purified via column chromatography (petroleum ether:EtOAc 10:1), thus providing sulfide **81** (18.6 g, 71.4 mmol, 73%) as a colorless oil. ^**1**^**H NMR (400 MHz, CDCl**_**3**_**)** δ = 7.57–7.51 (m, 5H), 5.55–5.47 (m, 1H), 5.23–5.16 (m, 1H), 3.42 (dd, *J* = 12.5, 6.3 Hz, 1H), 3.29 (dd, *J* = 12.5, 8.1 Hz, 1H), 3.08–2.97 (m, 1H), 1.59 (dd, *J* = 7.0, 1.8 Hz, 3H), 1.12 (d, *J* = 6.6 Hz, 3H) ppm; ^**13**^**C{1H}-NMR (100 MHz, CDCl**_**3**_**)** δ = 154.8, 133.9, 133.5, 130.2, 129.9, 125.8, 124.0, 40.3, 31.4, 20.4, 13.3 ppm; **HRMS** (ESI) *m*/*z*: [M + Na]^+^ calcd for C_13_H_16_N_4_SNa 283.0993; found 283.0994; **[α]**_**D**_^**27.5**^ = +3.67 (*c* = 1.00, CHCl_3_); **R**_*f*_ = 0.33 (petroleum ether:EtOAc 9:1).

#### (*S*,*Z*)-5-((2-Methylpent-3-en-1-yl)sulfonyl)-1-phenyl-1*H*-tetrazole (**44**)

The glassware was neither dried nor purged with inert gas prior to usage. Ammonium heptamolybdate tetrahydrate (6.46 g, 5.22 mmol, 0.10 equiv) was dissolved in an aqueous solution of H_2_O_2_ (30% (w/w), 53 mL, 515 mmol, 10.0 equiv) and added over the course of 5 min to a solution of sulfide **81** (13.4 g, 51.5 mmol, 1.00 equiv) in EtOH (340 mL) at 0 °C. The reaction mixture was stirred for 5 min at 0 °C and for 17 h at room temperature before it was diluted with water (200 mL) and CH_2_Cl_2_ (300 mL). The phases were separated and the aqueous layer was extracted with CH_2_Cl_2_ (3×). The combined organic layers were washed with brine, dried over MgSO_4_, and concentrated *in vacuo*. The crude product was purified via column chromatography (petroleum ether:EtOAc 10:1) providing sulfone **44** (8.75 g, 29.9 mmol, 58%, *E*:*Z* = 1:11, partial double bond isomerization could not be fully suppressed) as a colorless solid. ^**1**^**H NMR (400 MHz, CDCl**_**3**_**)** δ = 7.67–7.57 (m, 5H), 5.48–5.40 (m, 1H), 5.19–5.13 (m, 1H), 3.81 (dd, *J* = 14.4, 7.8 Hz, 1H), 3.68 (dd, *J* = 14.4, 5.9 Hz, 1H), 3.42–3.31 (m, 1H), 1.58 (dd, *J* = 6.8, 1.9 Hz, 3H), 1.18 (d, *J* = 6.7 Hz, 3H) ppm; ^**13**^**C{1H}-NMR (100 MHz, CDCl**_**3**_**)** δ = 154.1, 133.2, 131.8, 131.6, 129.8, 125.9, 125.3, 61.8, 27.0, 21.0, 13.1 ppm; **HRMS** (ESI) *m*/*z*: [M + Na] calcd for C_13_H_16_N_4_O_2_SNa 315.0892; found 315.0893; **[α]**_**D**_^**25.9**^ = +1.0 (*c* = 1.00, CHCl_3_); **R**_*f*_ = 0.30 (petroleum ether:EtOAc 9:1); **Melting point:** 60.3–62.3 °C.

#### (2*R*,3*S*)-3-((*R*)-2,2-Dimethyl-4-((1*R*,2*E*,4*S*,5*Z*)-4-methyl-1-((triethylsilyl)oxy)hepta-2,5-dien-1-yl)-1,3-dioxolan-4-yl)-3-((4-methoxybenzyl)oxy)-2-methylpropyl Pivalate (**82**)

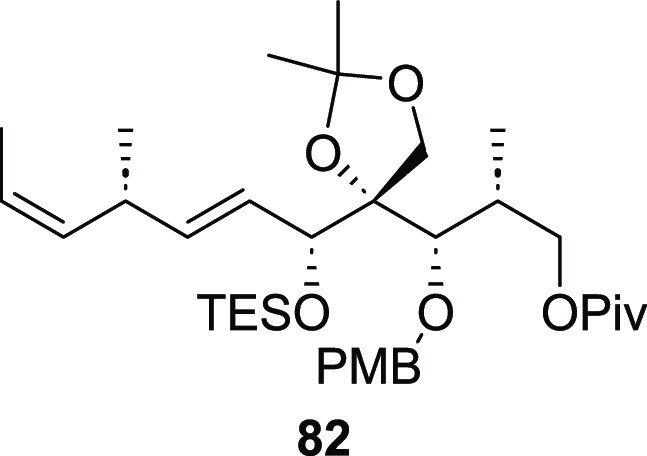



Potassium bis(trimethylsilyl)amide (1 M in THF, 15.0 mL, 15.0 mmol, 2.55 equiv) was added dropwise to a solution of sulfone **44** (4.39 g, 15.0 mmol, 2.55 equiv) in THF (24.0 mL) at −78 °C. The reaction mixture was stirred for 1 h before a solution of aldehyde **41** (3.34 g, 5.89 mmol, 1.00 equiv) in THF (20.0 mL) was added. The reaction mixture was stirred 2 h at room temperature before a saturated aqueous solution of NH_4_Cl was added. The phases were separated, and the aqueous layer was extracted with MTBE (3×). The combined organic layers were successively washed with a saturated aqueous solution of NaHCO_3_ and brine, dried over MgSO_4_, and concentrated *in vacuo*. The crude product was purified via column chromatography (petroleum ether:EtOAc 20:1) providing alkene **82** (3.20 g, 5.06 mmol, 86%) as a yellow oil. ^**1**^**H NMR (400 MHz, CDCl**_**3**_**)** δ = 7.24–7.21 (m, 2H), 6.87–6.83 (m, 2H), 5.67–5.61 (m, 1H), 5.54 (ddd, *J* = 15.6, 6.8, 1.2 Hz, 1H), 5.48–5.40 (m, 1H), 5.28–5.22 (m, 1H), 4.82 (d, *J* = 10.8 Hz, 1H), 4.36 (d, *J* = 10.8 Hz, 1H), 4.17 (d, *J* = 6.7 Hz, 1H), 4.12 (d, *J* = 8.4 Hz, 1H), 4.08 (d, *J* = 8.4 Hz, 1H), 3.97 (dd, *J* = 10.6, 8.1 Hz, 1H), 3.88 (dd, *J* = 10.6, 7.1 Hz, 1H), 3.84 (d, *J* = 1.7 Hz, 1H), 3.79 (s, 3H), 3.26 (sext, *J* = 7.2 Hz, 1H), 2.54–2.45 (m, 1H), 1.65 (dd, *J* = 6.8, 1.8 Hz, 3H), 1.47 (s, 3H), 1.33 (s, 3H), 1.21 (s, 9H), 1.10 (d, *J* = 6.8 Hz, 3H), 0.95 (t, *J* = 8.0 Hz, 9H), 0.80 (d, *J* = 6.8 Hz, 3H), 0.60 (q, *J* = 8.1 Hz, 6H) ppm; ^**13**^**C{1H}-NMR (100 MHz, CDCl**_**3**_**)** δ = 178.4, 159.0, 138.3, 134.4, 131.3, 128.9, 125.6, 123.3, 113.8, 108.5, 89.2, 78.1, 75.7, 75.5, 67.7, 65.9, 55.4, 38.9, 34.1, 34.0, 28.4, 27.4, 26.1, 20.5, 13.1, 11.2, 6.9, 5.1 ppm; **HRMS** (ESI) *m*/*z*: [M + Na]^+^ calcd for C_36_H_60_O_7_SiNa 655.4006; found 655.4008; **[α]**_**D**_^**22.2**^ = +46.0 (*c* = 1.00, CHCl_3_); **R**_*f*_ = 0.25 (petroleum ether:EtOAc 19:1).

#### (2*R*,3*S*,4*S*,5*R*,6*E*,8*S*,9*Z*)-4,5-Dihydroxy-4-(hydroxymethyl)-3-((4-methoxybenzyl)oxy)-2,8-dimethylundeca-6,9-dien-1-yl Pivalate (**45**)

The glassware was neither dried nor purged with inert gas prior to usage. Trifluoroacetic acid (23.0 mL, 298 mmol, 76.0 equiv) was added to a solution of alkene **82** (2.48 g, 3.92 mmol, 1.00 equiv) in THF (115 mL) and water (57 mL) at room temperature. The reaction mixture was stirred for 13 h at 50 °C before it was cooled to 0 °C. Triethylamine (54 mL, 392 mmol, 100 equiv) was added and stirring was continued for 20 min before EtOAc (200 mL) and a saturated aqueous solution of NaHCO_3_ (100 mL) were added. The phases were separated and the aqueous layer was extracted with EtOAc (4×). The combined organic layers were washed with brine, dried over MgSO_4_, and concentrated *in vacuo*. The crude product was purified via column chromatography (petroleum ether:EtOAc 4:1 → 3:1 → 2:1) providing triol **45** (1.52 g, 3.18 mmol, 81%) as a colorless oil. ^**1**^**H NMR (400 MHz, CDCl**_**3**_**)** δ = 7.27–7.23 (m, 2H), 6.90–6.86 (m, 2H), 5.73 (ddd, *J* = 15.6, 6.1, 0.8 Hz, 1H), 5.59 (ddd, *J* = 15.6, 7.1, 1.3 Hz, 1H), 5.47–5.39 (m, 1H), 5.23–5.16 (m, 1H), 4.60 (d, *J* = 10.8 Hz, 1H), 4.55 (d, *J* = 10.8 Hz, 1H), 4.17 (dd, *J* = 7.0, 4.6 Hz, 1H), 4.03 (dd, *J* = 10.9, 6.7 Hz, 1H), 3.98 (dd, *J* = 10.9, 8.3 Hz, 1H), 3.81 (s, 4H), 3.67 (dd, *J* = 11.7, 5.6 Hz, 1H), 3.59 (dd, *J* = 11.7, 6.7 Hz, 1H), 3.21 (sext, *J* = 7.4 Hz, 1H), 3.12 (s, 1H), 2.73–2.70 (m, 1H), 2.41–2.30 (m, 2H), 1.61 (dd, *J* = 6.8, 1.8 Hz, 3H), 1.23 (s, 9H), 1.05 (d, *J* = 7.0 Hz, 3H), 1.05 (d, *J* = 6.9 Hz, 3H) ppm; ^**13**^**C{1H}-NMR (100 MHz, CDCl**_**3**_**)** δ = 178.5, 159.6, 139.1, 134.0, 130.0, 129.6, 125.5, 123.7, 114.1, 80.6, 77.9, 75.8, 74.9, 67.9, 64.1, 55.4, 39.0, 34.2, 33.9, 27.4, 20.7, 13.1, 11.9 ppm; **HRMS** (ESI) *m*/*z*: [M + Na]^+^ calcd for C_27_H_42_O_7_Na 501.2828; found 501.2826; **[α]**_**D**_^**21.9**^ = +69.0 (*c* = 1.00, CHCl_3_); **R**_*f*_ = 0.22 (petroleum ether:EtOAc 3:1), 0.62 (petroleum ether:EtOAc 1:1).

#### (2*R*,3*S*,4*R*,5*R*,6*E*,8*S*,9*Z*)-3-((4-Methoxybenzyl)oxy)-2,8-dimethyl-4,5-bis((triethylsilyl)oxy)-4-(((triethylsilyl)oxy)methyl)undeca-6,9-dien-1-yl Pivalate (**83**)

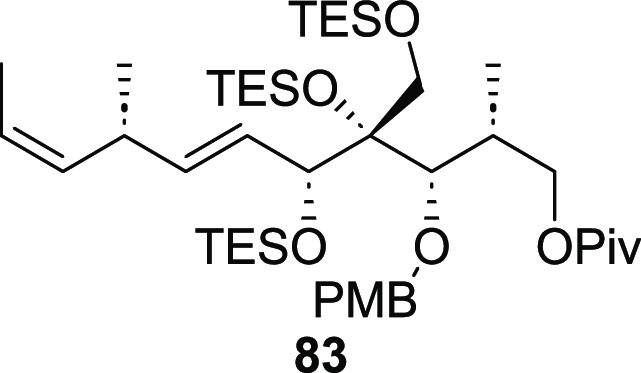



2,6-Lutidine (6.8 mL, 58.6 mmol, 12.0 equiv) and triethylsilyl trifluoromethanesulfonate (6.6 mL, 29.3 mmol, 6.00 equiv) were added successively to a solution of triol **45** (2.34 g, 4.88 mmol, 1.00 equiv) in CH_2_Cl_2_ (24.0 mL) at −78 °C. The reaction mixture was stirred for 30 min at −78 °C and for 1.5 h at room temperature before a saturated aqueous solution of NaHCO_3_ was added. The phases were separated and the aqueous layer was extracted with CH_2_Cl_2_ (3×). The combined organic layers were successively washed with an aqueous solution of KHSO_4_ (1 M), a saturated aqueous solution of NaHCO_3_ and brine, dried over MgSO_4_, and concentrated *in vacuo*. The crude product was purified via column chromatography (petroleum ether:EtOAc 50:1) providing TES-ether **83** (3.77 g, 4.59 mmol, 94%) as a colorless oil. ^**1**^**H NMR (400 MHz, CDCl**_**3**_**)** δ = 7.25–7.22 (m, 2H), 6.87–6.84 (m, 2H), 5.64 (ddd, *J* = 15.6, 9.1, 1.2 Hz, 1H), 5.46–5.36 (m, 2H), 5.21–5.15 (m, 1H), 4.46 (s, 2H), 4.44 (d, *J* = 9.2 Hz, 1H), 4.01–3.92 (m, 2H), 3.82 (d, *J* = 9.8 Hz, 1H), 3.81 (s, 3H), 3.61 (d, *J* = 9.9 Hz, 1H), 3.57 (d, *J* = 2.3 Hz, 1H), 3.21–3.12 (m, 1H), 2.62–2.53 (m, 1H), 1.61 (dd, *J* = 6.8, 1.7 Hz, 3H), 1.21 (s, 9H), 1.02 (d, *J* = 6.5 Hz, 3H), 1.00 (d, *J* = 6.6 Hz, 3H), 0.99–0.90 (m, 27H), 0.66–0.56 (m, 18H) ppm; ^**13**^**C{1H}-NMR (100 MHz, CDCl**_**3**_**)** δ = 178.6, 158.7, 138.2, 134.3, 131.8, 128.2, 128.1, 122.9, 113.6, 83.9, 80.8, 77.4, 73.6, 70.0, 63.7, 55.4, 38.9, 34.3, 33.7, 27.4, 20.4, 13.0, 12.7, 7.5, 7.2 (2 different carbon atoms), 7.0, 5.9, 4.5 ppm; **HRMS** (ESI) *m*/*z*: [M + Na]^+^ calcd for C_45_H_84_O_7_Si_3_Na 843.5423; found 843.5421; **[α]**_**D**_^**23.4**^ = +31.8 (*c* = 1.00, CHCl_3_); **R**_*f*_ = 0.24 (petroleum ether:EtOAc 19:1).

#### (2*R*,3*S*,4*R*,5*R*,6*E*,8*S*,9*Z*)-3-((4-Methoxybenzyl)oxy)-2,8-dimethyl-4,5-bis((triethylsilyl)oxy)-4-(((triethylsilyl)oxy)methyl)undeca-6,9-dien-1-ol (**84**)

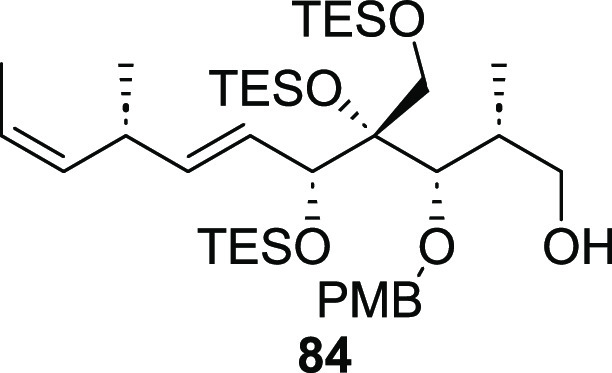



Diisobutylaluminum hydride (1 M in hexane, 7.5 mL, 7.50 mmol, 2.50 equiv) was added dropwise to a solution of TES-ether **83** (2.40 g, 2.93 mmol, 1.00 equiv) in CH_2_Cl_2_ (21.0 mL) at −78 °C. The reaction mixture was stirred for 1 h before MeOH (4.0 mL) was added. The resulting slurry was diluted with MTBE (100 mL) and poured into a vigorously stirred solution of Rochelle salt (100 mL) at room temperature. Stirring was continued until two phases were formed (1 h). The phases were separated and the aqueous layer was extracted with MTBE (3×). The combined organic layers were washed with brine, dried over MgSO_4_, and concentrated *in vacuo*. The crude product was purified via column chromatography (petroleum ether:EtOAc 10:1) providing alcohol **84** (2.11 g, 2.87 mmol, 98%) as a colorless oil. ^**1**^**H NMR (400 MHz, CDCl**_**3**_**)** δ = 7.26–7.22 (m, 2H), 6.87–6.84 (m, 2H), 5.64 (ddd, *J* = 15.6, 9.1, 1.4 Hz, 1H), 5.46–5.38 (m, 2H), 5.22–5.15 (m, 1H), 4.54–4.46 (m, 3H), 3.89 (d, *J* = 9.9 Hz, 1H), 3.81 (s, 3H), 3.69 (d, *J* = 1.9 Hz, 1H), 3.64 (d, *J* = 9.9 Hz, 1H), 3.49–3.46 (m, 2H), 3.21–3.12 (m, 1H), 2.38–2.29 (m, 1H), 1.83 (t, *J* = 5.4 Hz, 1H), 1.61 (dd, *J* = 6.7, 1.8 Hz 3H), 1.03 (d, *J* = 7.0 Hz, 3H), 1.01–0.92 (m, 30H), 0.69–0.57 (m, 18H) ppm; ^**13**^**C{1H}-NMR (100 MHz, CDCl**_**3**_**)** δ = 158.7, 137.9, 134.3, 132.0, 128.2, 128.1, 123.0, 113.6, 84.1, 80.6, 77.4, 73.5, 69.0, 63.5, 55.4, 36.7, 34.2, 20.3, 13.0, 12.4, 7.5, 7.2, 7.2, 7.0, 5.9, 4.5 ppm; **HRMS** (ESI) *m*/*z*: [M + Na]^+^ calcd for C_40_H_76_O_6_Si_3_Na 759.4847; found 759.4850; **[α]**_**D**_^**23.0**^ = +39.4 (*c* = 0.33, CHCl_3_); **R**_*f*_ = 0.41 (petroleum ether:EtOAc 9:1).

#### (2S,3S,4R,5R,6E,8S,9Z)-3-((4-Methoxybenzyl)oxy)-2,8-dimethyl-4,5-bis((triethylsilyl)oxy)-4-(((triethylsilyl)oxy)methyl)undeca-6,9-dienal (**47**)

A suspension of alcohol **84** (2.14 g, 2.90 mmol, 1.00 equiv), *N*-methylmorpholine *N*-oxide (1.02 g, 8.71 μmol, 3.00 equiv), and activated molecular sieves (4 Å, powdered, 2.55 g) in CH_2_Cl_2_ (14.5 mL) was stirred for 45 min at room temperature before tetrapropylammonium perruthenate (51.0 mg, 0.15 mmol, 0.05 equiv) was added. The reaction mixture was stirred for 45 min before it was diluted with petroleum ether (20.0 mL) and put onto a chromatography column. Purification via column chromatography (petroleum ether:EtOAc 50:1) provided southwestern fragment **47** (1.81 g, 2.46 μmol, 85%) as a colorless oil. Southwestern fragment **47** was used in the next reaction without detailed characterization.

#### (2*E*,4*S*,7*S*,8*R*,9*S*,10*R*,11*R*,12*E*,14*S*,15*Z*)-7-Hydroxy-9-((4-methoxybenzyl)oxy)-2,4,8,14-tetramethyl-5-oxo-10,11-bis((triethylsilyl)oxy)-10-(((triethylsilyl)oxy)methyl)heptadeca-2,12,15-trien-1-yl Pivalate (**48**)

A solution of eastern fragment **15** (124 mg, 548 μmol, 1.86 equiv in Et_2_O (1.00 mL) was added dropwise to a solution of (+)-B-chlordiisopinocampheylboran (156 mg, 486 μmol, 1.65 equiv) and triethylamine (0.08 mL, 588 μmol, 2.00 equiv) in Et_2_O (0.50 mL) at 0 °C. The reaction mixture was stirred for 1 h at 0 °C before it was cooled to −78 °C and a solution of southwestern fragment **47** (216 mg, 294 μmol, 1.00 equiv) in Et_2_O (1.4 mL) was added. The reaction mixture was stirred for 1 h at −78 °C and for 14.5 h at −25 °C before an aqueous solution of phosphate buffer (pH = 7, 2.0 mL), an aqueous solution of H_2_O_2_ (30% (w/w), 2.0 mL) and MeOH (2.0 mL) were added. The temperature was raised to 0 °C and stirring was continued for 1 h. The phases were separated and the aqueous layer was extracted with MTBE (3×). The combined organic layers were washed with brine, dried over MgSO_4_, and concentrated *in vacuo*. The crude product was loaded onto silica and purified via column chromatography (petroleum ether:EtOAc 10:1), thus providing alcohol **48** (150 mg, 156 μmol, 53%) as a colorless oil. ^**1**^**H NMR (400 MHz, CDCl**_**3**_**)** δ = 7.26–7.23 (m, 2H), 6.85–6.82 (m, 2H), 5.63 (ddd, *J* = 15.7, 8.9, 1.3 Hz, 1H), 5.45–5.37 (m, 3H), 5.22–5.16 (m, 1H), 4.69 (d, *J* = 11.8 Hz, 1H), 4.46 (s, 2H), 4.43 (d, *J* = 11.8 Hz, 1H), 4.42 (d, *J* = 8.9 Hz, 1H), 4.14–4.10 (m, 1H), 3.79 (s, 3H), 3.77 (d, *J* = 10.0 Hz, 1H), 3.75 (d, *J* = 3.0 Hz, 1H), 3.54 (d, *J* = 9.9 Hz, 1H), 3.43–3.36 (m, 1H), 3.22–3.13 (m, 1H), 2.93 (d, *J* = 2.8 Hz, 1H), 2.66 (dd, *J* = 17.8, 9.8 Hz, 1H), 2.44 (dd, *J* = 17.8, 2.3 Hz, 1H), 2.34–2.27 (m, 1H), 1.70 (d, *J* = 1.3 Hz, 3H), 1.61 (dd, *J* = 6.8, 1.7 Hz, 3H), 1.21 (s, 9H), 1.15 (d, *J* = 6.9 Hz, 3H), 1.03 (d, *J* = 6.9 Hz, 3H), 0.99–0.90 (m, 30H), 0.65–0.55 (m, 18H) ppm; ^**13**^**C{1H}-NMR (100 MHz, CDCl**_**3**_**)** δ = 212.7, 178.3, 158.5, 137.6, 134.3, 133.3, 132.5, 128.3, 128.1, 127.2, 123.0, 113.5, 83.8, 82.5, 72.7, 70.9, 69.1, 63.8, 55.4, 46.5, 45.9, 39.0, 38.1, 34.2, 27.3, 20.3, 16.6, 14.3, 13.0, 8.5, 7.6, 7.2, 7.2, 7.1, 5.9, 4.6 ppm (1 carbon atom is overlapped by CDCl_3_); **HRMS** (ESI) *m*/*z*: [M + Na]^+^ calcd for C_53_H_96_O_9_Si_3_Na 983.6260; found 983.6261; **[α]**_**D**_^**23.2**^ = +73.3 (*c* = 0.44, CHCl_3_); **R**_*f*_ = 0.41 (petroleum ether:EtOAc 9:1).

#### (2*E*,4*S*,5*S*,7*S*,8*R*,9*S*,10*R*,11*R*,12*E*,14*S*,15*Z*)-5,7-Dihydroxy-9-((4-methoxybenzyl)oxy)-2,4,8,14-tetramethyl-10,11-bis((triethylsilyl)oxy)-10-(((triethylsilyl)oxy)methyl)heptadeca-2,12,15-trien-1-yl Pivalate (**85**)

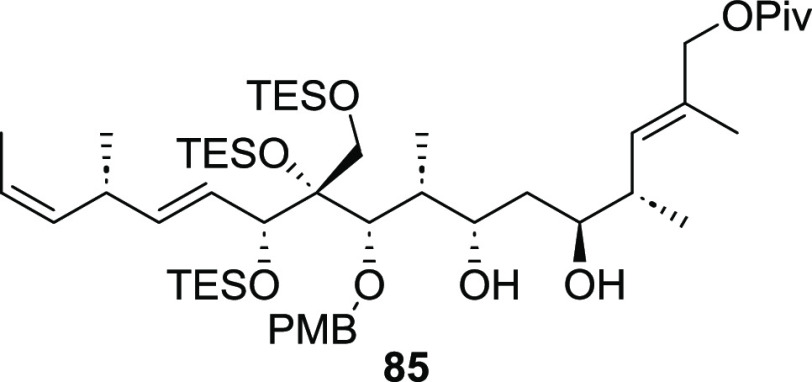



Acetic acid (21.5 mL) was added to a suspension of tetramethylammonium triacetoxyborohydride (920 mg, 3.50 mmol, 8.20 equiv) in MeCN (21.5 mL) at 0 °C. The reaction mixture was stirred for 45 min before a solution of alcohol **48** (410 mg, 0.43 mmol, 1.00 equiv) in MeCN (9.0 mL) was added. The reaction mixture was stirred for 20.25 h before a solution of Rochelle salt was added. The reaction mixture was warmed to room temperature and diluted with CH_2_Cl_2_, and a saturated aqueous solution of NaHCO_3_ (400 mL) was added. The phases were separated and the aqueous layer was extracted with CH_2_Cl_2_ (3×). The combined organic layers were washed with brine, dried over MgSO_4_, and concentrated *in vacuo*. The crude product was purified via column chromatography (petroleum ether:EtOAc 5:1) providing diol **85** (296 mg, 307 μmol, 71%) as a colorless oil. ^**1**^**H NMR (400 MHz, CDCl**_**3**_**)** δ = 7.25–7.21 (m, 2H), 6.87–6.83 (m, 2H), 5.60 (ddd, *J* = 15.7, 8.2, 1.0 Hz, 1H), 5.52–5.39 (m, 2H), 5.27 (dd, *J* = 9.9, 1.0 Hz, 1H), 5.23–5.17 (m, 1H), 4.71 (d, *J* = 11.3 Hz, 1H), 4.52 (d, *J* = 11.3 Hz, 1H), 4.45–4.37 (m, 2H), 4.39 (d, *J* = 8.3 Hz, 1H), 3.96 (d, *J* = 10.5 Hz, 1H), 3.82–3.78 (m, 2H), 3.80 (s, 3H), 3.76 (d, *J* = 10.0 Hz, 1H), 3.67–3.61 (m, 1H), 3.23–3.14 (m, 1H), 2.62 (bs, 1H), 2.55–2.45 (m, 1H), 2.32–2.27 (m, 1H), 2.13 (bs, 1H), 1.70–1.62 (m, 1H), 1.66 (d, *J* = 1.2 Hz, 3H), 1.63 (dd, *J* = 6.8, 1.8 Hz, 3H), 1.35–1.27 (m, 1H), 1.19 (s, 9H), 1.05 (d, *J* = 6.9 Hz, 3H), 1.02 (d, *J* = 6.8 Hz, 3H), 1.01–0.89 (m, 30H), 0.69–0.63 (m, 12H), 0.59 (q, *J* = 8.0 Hz, 6H) ppm; ^**13**^**C{1H}-NMR (100 MHz, CDCl**_**3**_**)** δ = 178.4, 158.9, 138.1, 134.2, 131.7 (2 different carbon atoms), 130.7, 128.4, 127.7, 123.1, 113.8, 85.6, 84.4, 76.4, 73.7, 73.3, 73.0, 70.1, 63.5, 55.4, 39.0, 38.9, 38.8, 38.8, 34.2, 27.3, 20.4, 16.6, 14.4, 13.0, 8.9, 7.7, 7.3, 7.2 (2 different carbon atoms), 5.8, 4.6 ppm; **HRMS** (ESI) *m*/*z*: [M + Na]^+^ calcd for C_53_H_98_O_9_Si_3_Na 985.6416; found 985.6417; **[α]**_**D**_^**22.2**^ = +30.0 (*c* = 0.10, CHCl_3_); **R**_*f*_ = 0.39 (petroleum ether:EtOAc 5:1).

#### (*S*,*E*)-4-((4*S*,6*S*)-6-((2*R*,3*S*,4*R*,5*R*,6*E*,8*S*,9*Z*)-3-((4-Methoxybenzyl)oxy)-8-methyl-4,5-bis((triethylsilyl)oxy)-4-(((triethylsilyl)oxy)methyl)undeca-6,9-dien-2-yl)-2,2-dimethyl-1,3-dioxan-4-yl)-2-methylpent-2-en-1-yl Pivalate (**49**)

The glassware was not dried prior to usage. Pyridinium *p*-toluenesulfonate (4.1 mg, 16.3 μmol, 0.06 equiv) was added to a solution of diol **85** (284 mg, 295 μmol, 1.00 equiv) and 2,2-dimethoxypropan (2.2 mL, 17.7 mmol, 60.0 equiv) in acetone (2.2 mL) at room temperature. The reaction mixture was stirred for 13.5 h before the subsequent addition of a saturated aqueous solution of NaHCO_3_ and MTBE. The phases were separated and the aqueous layer was extracted with MTBE (3×). The combined organic layers were washed with brine, dried over MgSO_4_, and concentrated *in vacuo*. The crude product was purified via column chromatography (petroleum ether:EtOAc 20:1) providing acetonide **49** (277 mg, 276 μmol, 94%) as a colorless oil. ^**1**^**H NMR (400 MHz, CDCl**_**3**_**)** δ = 7.26–7.23 (m, 2H), 6.87–6.83 (m, 2H), 5.62 (dd, *J* = 15.5, 9.2 Hz, 1H), 5.42–5.34 (m, 2H), 5.24 (d, *J* = 9.6 Hz, 1H), 5.18–5.13 (m, 1H), 4.73 (d, *J* = 11.4 Hz, 1H), 4.47–4.40 (m, 3H), 4.18 (d, *J* = 11.4 Hz, 1H), 3.82 (d, *J* = 12.5 Hz, 1H), 3.81 (s, 3H), 3.75–3.70 (m, 1H), 3.70–3.52 (m, 3H), 3.18–3.09 (m, 1H), 2.47–2.37 (m, 1H), 2.23–2.18 (m, 1H), 1.73–1.62 (m, 1H), 1.66 (d, *J* = 0.7 Hz, 3H), 1.59 (dd, *J* = 6.8, 1.7 Hz, 3H), 1.44–1.36 (m, 1H), 1.33 (s, 3H), 1.30 (s, 3H), 1.21 (s, 9H), 1.00–0.90 (m, 36H), 0.65–0.55 (m, 18H) ppm; ^**13**^**C{1H}-NMR (100 MHz, CDCl**_**3**_**)** δ = 178.4, 158.4, 137.3, 134.4, 132.9, 131.0, 130.7, 128.9, 127.9, 122.6, 113.4, 100.1, 83.7, 82.2, 77.6, 72.1, 71.1, 70.7, 70.0, 63.7, 55.4, 39.0, 37.9, 37.1, 34.4, 34.2, 27.3, 24.7, 24.6, 20.4, 17.0, 14.4, 12.9, 8.6, 7.5, 7.3, 7.2, 6.9, 5.9, 4.6 ppm; **HRMS** (ESI) *m*/*z*: [M + Na]^+^ calcd for C_56_H_102_O_9_Si_3_Na 1025.6729; found 1025.6729; **[α]**_**D**_^**22.3**^ = +28.8 (*c* = 1.00, CHCl_3_); **R**_*f*_ = 0.35 (petroleum ether:EtOAc 50:1).

#### (*S*,*E*)-4-((4*S*,6*S*)-6-((2*R*,3*S*,4*R*,5*R*,6*E*,8*S*,9*Z*)-3-((4-Methoxybenzyl)oxy)-8-methyl-4,5-bis((triethylsilyl)oxy)-4-(((triethylsilyl)oxy)methyl)undeca-6,9-dien-2-yl)-2,2-dimethyl-1,3-dioxan-4-yl)-2-methylpent-2-en-1-ol (**86**)

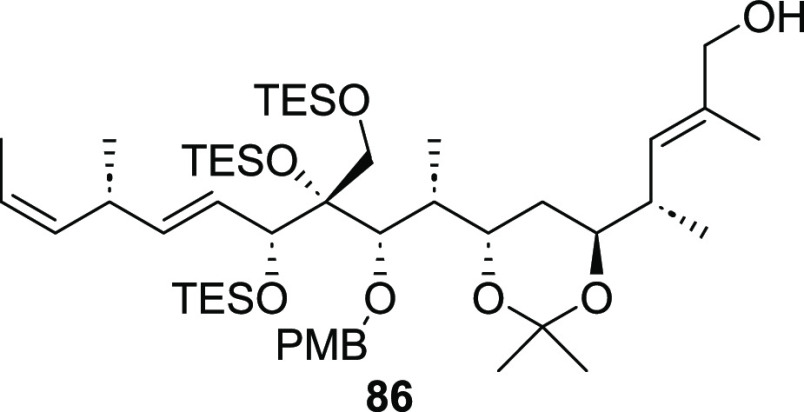



Diisobutylaluminum hydride (1 M in hexane, 0.83 mL, 830 μmol, 2.70 equiv) was added dropwise to a solution of acetonide **49** (309 mg, 308 μmol, 1.00 equiv) in CH_2_Cl_2_ (2.3 mL) at −78 °C. The reaction mixture was stirred for 1 h before MeOH (0.50 mL) was added. The resulting slurry was diluted with MTBE (10 mL) and poured into a vigorously stirred solution of Rochelle salt (10 mL) at room temperature. Stirring was continued until two phases were formed (30 min). The phases were separated and the aqueous layer was extracted with MTBE (3×). The combined organic layers were washed with brine, dried over MgSO_4_, and concentrated *in vacuo*. The crude product was purified via column chromatography (petroleum ether:EtOAc 10:1) providing alcohol **86** (273 mg, 297 μmol, 96%) as a colorless oil. ^**1**^**H NMR (400 MHz, CDCl**_**3**_**)** δ = 7.27–7.23 (m, 2H), 6.87–6.83 (m, 2H), 5.63 (ddd, *J* = 15.6, 9.2, 1.3 Hz, 1H), 5.42–5.34 (m, 2H), 5.21–5.13 (m, 2H), 4.71 (d, *J* = 11.8 Hz, 1H), 4.44 (d, *J* = 9.1 Hz, 1H), 4.21 (d, *J* = 11.8 Hz, 1H), 3.98 (s, 2H), 3.82 (d, *J* = 10.4 Hz, 1H), 3.81 (s, 3H), 3.75–3.70 (m, 1H), 3.52 (d, *J* = 2.0 Hz, 1H), 3.49–3.44 (m, 2H), 3.18–3.09 (m, 1H), 2.48–2.38 (m, 1H), 2.26–2.20 (m, 1H), 1.74–1.67 (m, 1H), 1.68 (d, *J* = 1.0 Hz, 3H), 1.59 (dd, *J* = 6.8, 1.7 Hz, 3H), 1.45–1.38 (m, 1H), 1.33 (s, 3H), 1.30 (s, 3H), 1.00–0.90 (m, 36H), 0.66–0.55 (m, 18H) ppm; ^**13**^**C{1H}-NMR (100 MHz, CDCl**_**3**_**)** δ = 158.4, 137.4, 135.2, 134.4, 132.9, 128.8, 128.0, 127.9, 122.7, 113.4, 100.1, 83.8, 82.1, 77.6, 72.1, 71.2, 70.7, 69.0, 63.7, 55.4, 37.9, 37.1, 34.4, 34.2, 24.7, 24.7, 20.4, 17.2, 14.1, 13.0, 8.8, 7.5, 7.3, 7.2, 7.0, 6.0, 4.6 ppm; **HRMS** (ESI) *m*/*z*: [M + Na]^+^ calcd for C_51_H_94_O_8_Si_3_Na 941.6154; found 941.6153; **[α]**_**D**_^**22.8**^ = +30.0 (*c* = 0.26, CHCl_3_); **R**_*f*_ = 0.19 (petroleum ether:EtOAc 9:1).

#### (*S*,*E*)-4-((4*S*,6*S*)-6-((2*R*,3*S*,4*R*,5*R*,6*E*,8*S*,9*Z*)-3-((4-Methoxybenzyl)oxy)-8-methyl-4,5-bis((triethylsilyl)oxy)-4-(((triethylsilyl)oxy)methyl)undeca-6,9-dien-2-yl)-2,2-dimethyl-1,3-dioxan-4-yl)-2-methylpent-2-enal (**50**)

A suspension of alcohol **86** (235 mg, 256 μmol, 1.00 equiv), *N*-methylmorpholine *N*-oxide (90.0 mg, 767 μmol, 3.00 equiv), and activated molecular sieves (4 Å, powdered, 225 mg) in CH_2_Cl_2_ (2.5 mL) was stirred for 1 h at room temperature before tetrapropylammonium perruthenate (9.9 mg, 28.2 μmol, 0.11 equiv) was added. The reaction mixture was stirred for 30 min before it was diluted with petroleum ether (4.0 mL) and put onto a chromatography column. Purification via column chromatography (petroleum ether:EtOAc 20:1) provided aldehyde **50** (215 mg, 234 μmol, 92%) as a colorless oil. Aldehyde **50** was used in the next reaction without detailed characterization.

#### Allyl (3*R*,10*S*,*E*)-3-((*tert*-Butyldimethylsilyl)oxy)-7-hydroxy-10-((4*S*,6*S*)-6-((2*R*,3*S*,4*R*,5*R*,6*E*,8*S*,9*Z*)-3-((4-methoxybenzyl)oxy)-8-methyl-4,5-bis((triethylsilyl)oxy)-4-(((triethylsilyl)oxy)methyl)undeca-6,9-dien-2-yl)-2,2-dimethyl-1,3-dioxan-4-yl)-4,4,6,8-tetramethyl-5-oxoundec-8-enoate (**87**)

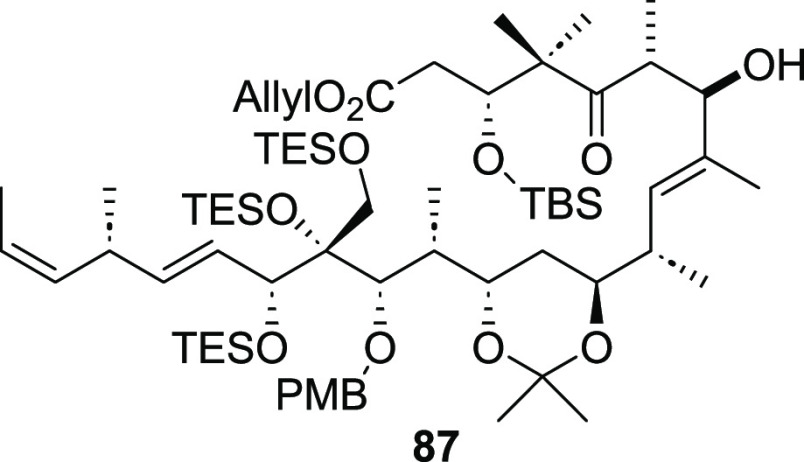



Titanium tetrachloride (1 M in CH_2_Cl_2_, 0.35 mL, 350 μmol, 1.50 equiv) and *N*,*N*-diisopropylethylamine (0.12 mL, 706 μmol, 3.02 equiv) were added successively to a solution of northern fragment **11** (132 mg, 385 μmol, 1.65 equiv) in CH_2_Cl_2_ (2.2 mL) at −78 °C. The reaction mixture was stirred for 65 min before a solution of aldehyde **50** (215 mg, 234 μmol, 1.00 equiv) in CH_2_Cl_2_ (2.2 mL) was added dropwise over the course of 5 min. The reaction mixture was stirred for 5 min at −78 °C and for 1.75 h at −20 °C before an aqueous solution of phosphate buffer (pH = 7) was added. The phases were separated and the aqueous layer was extracted with CH_2_Cl_2_ (3×). The combined organic layers were washed with brine, dried over MgSO_4_, and concentrated *in vacuo*. The crude product was purified via column chromatography (petroleum ether:EtOAc 10:1) providing alcohol **87** (237 mg, 188 μmol, 80%, mixture of two diastereomers, dr could not be determined by ^1^H NMR) as a colorless oil. Alcohol **87** was used in the next reaction without detailed characterization.

#### Allyl (3*R*,10*S*,*E*)-3-((*tert*-butyldimethylsilyl)oxy)-10-((4*S*,6*S*)-6-((2*R*,3*S*,4*R*,5*R*,6*E*,8*S*,9*Z*)-3-((4-methoxybenzyl)oxy)-8-methyl-4,5-bis((triethylsilyl)oxy)-4-(((triethylsilyl)oxy)methyl)undeca-6,9-dien-2-yl)-2,2-dimethyl-1,3-dioxan-4-yl)-4,4,6,8-tetramethyl-5-oxo-7-((triisopropylsilyl)oxy)undec-8-enoate (**51**)

2,6-Lutidine (0.08 mL, 687 μmol, 3.65 equiv) and triisopropylsilyl trifluoromethanesulfonate (0.09 mL, 335 μmol, 1.78 equiv) were added successively to a solution of alcohol **87** (237 mg, 188 μmol, 1.00 equiv) in CH_2_Cl_2_ (1.9 mL) at −78 °C. The reaction mixture was stirred for 10 min at −78 °C and for 5 h at 0 °C before a saturated aqueous solution of NaHCO_3_ was added. The phases were separated and the aqueous layer was extracted with CH_2_Cl_2_ (3×). The combined organic layers were successively washed with an aqueous solution of KHSO_4_ (1 M), a saturated aqueous solution of NaHCO_3_ and brine, dried over MgSO_4_ and concentrated *in vacuo*. The crude product was purified via column chromatography (petroleum ether:EtOAc 50:1) providing two diastereomers of TIPS-ether **51** (major: 137 mg, 96.7 μmol, 51%, minor: 76.0 mg, 53.7 μmol, 29%) as colorless oils. Analytical data major: ^**1**^**H NMR (400 MHz, CDCl**_**3**_**)** δ = 7.27–7.23 (m, 2H), 6.87–6.83 (m, 2H), 5.96–5.86 (m, 1H), 5.67 (ddd, *J* = 15.6, 9.2, 1.0 Hz, 1H), 5.40–5.29 (m, 3H), 5.24–5.21 (m, 1H), 5.18–5.12 (m, 1H), 5.09 (d, *J* = 9.6 Hz, 1H), 4.77 (d, *J* = 11.9 Hz, 1H), 4.58–4.56 (m, 2H), 4.50 (d, *J* = 9.0 Hz, 1H), 4.39 (d, *J* = 8.7 Hz, 1H), 4.31 (dd, *J* = 6.7, 3.1 Hz, 1H), 4.10 (d, *J* = 11.9 Hz, 1H), 3.89 (d, *J* = 9.8 Hz, 1H), 3.81 (s, 3H), 3.73–3.69 (m, 1H), 3.48–3.41 (m, 2H), 3.37 (d, *J* = 9.8 Hz, 1H), 3.19–3.08 (m, 2H), 2.49 (dd, *J* = 16.5, 3.2 Hz, 1H), 2.32–2.23 (m, 2H), 2.28 (dd, *J* = 16.5, 6.7 Hz, 1H), 1.78–1.71 (m, 1H), 1.62 (s, 3H), 1.57 (dd, *J* = 6.8, 1.8 Hz, 3H), 1.39–1.32 (m, 1H), 1.32 (s, 3H), 1.28 (s, 3H), 1.21 (s, 3H), 1.14 (d, *J* = 6.8 Hz, 3H), 1.09–1.04 (m, 21H), 1.00–0.87 (m, 39H), 0.87 (s, 9H), 0.68–0.52 (m, 18H), 0.09 (s, 3H), 0.01 (s, 3H) ppm; ^**13**^**C{1H}-NMR (100 MHz, CDCl**_**3**_**)** δ = 216.8, 171.9, 158.3, 137.1, 136.0, 134.5, 133.0, 132.2, 130.4, 129.1, 127.8, 122.5, 118.6, 113.4, 100.1, 84.0, 81.9, 80.3, 78.2, 74.3, 71.8, 71.0, 70.3, 65.4, 63.5, 55.4, 53.3, 47.2, 40.4, 38.0, 37.1, 34.7, 34.3, 26.1, 24.7 (2 different carbon atoms), 23.2, 20.4, 19.6, 18.5, 18.4, 18.4, 16.5, 15.9, 13.1, 12.9, 11.8, 8.4, 7.5, 7.3, 7.3, 6.9, 6.0, 4.6, – 4.2, – 4.6 ppm; **HRMS** (ESI) *m*/*z*: [M + Na]^+^ calcd for C_78_H_146_O_12_Si_5_Na 1437.9558; found 1437.9558; **[α]**_**D**_^**24.0**^ = +37.65 (*c* = 0.17, CHCl_3_); **R**_*f*_ = 0.41 (petroleum ether:EtOAc 19:1). Analytical data minor: ^**1**^**H NMR (400 MHz, CDCl**_**3**_**)** δ = 7.26–7.23 (m, 2H), 6.86–6.83 (m, 2H), 5.93–5.83 (m, 1H), 5.66 (ddd, *J* = 15.6, 9.3, 1.2 Hz, 1H), 5.40–5.26 (m, 3H), 5.23–5.11 (m, 3H), 4.77 (d, *J* = 11.9 Hz, 1H), 4.56–4.54 (m, 2H), 4.50–4.47 (m, 2H), 4.36 (d, *J* = 8.1 Hz, 1H), 4.10 (d, *J* = 11.9 Hz, 1H), 3.89 (d, *J* = 9.8 Hz, 1H), 3.80 (s, 3H), 3.72–3.68 (m, 1H), 3.48–3.40 (m, 2H), 3.36 (d, *J* = 9.8 Hz, 1H), 3.18–3.07 (m, 2H), 2.37–2.22 (m, 3H), 2.16–2.07 (m, 1H), 1.76–1.67 (m, 1H), 1.61 (d, *J* = 0.7 Hz, 3H), 1.57 (dd, *J* = 6.8, 1.7 Hz, 3H), 1.38–1.27 (m, 1H), 1.32 (s, 3H), 1.29 (s, 3H), 1.13 (s, 3H), 1.12 (d, *J* = 6.8 Hz, 3H), 1.07–1.05 (m, 21H), 0.99–0.90 (m, 39H), 0.86 (s, 9H), 0.70–0.52 (m, 18H), 0.09 (s, 3H), 0.01 (s, 3H) ppm; ^**13**^**C{1H}-NMR (100 MHz, CDCl**_**3**_**)** δ = 215.5, 171.9, 158.3, 137.2, 135.8, 134.4, 133.0, 132.1, 130.5, 129.1, 127.8, 122.5, 118.6, 113.4, 100.1, 84.0, 81.9, 80.4, 78.2, 72.2, 71.8, 70.7, 70.4, 65.4, 63.5, 55.4, 54.5, 46.7, 39.9, 37.8, 37.1, 34.6, 34.3, 26.1, 24.7, 24.7, 22.4, 20.4, 18.5, 18.4, 18.4, 17.7, 15.9, 15.7, 13.0, 12.9, 12.3, 8.5, 7.5, 7.3, 7.3, 6.9, 6.0, 4.6, – 4.3, – 4.4 ppm; **HRMS** (ESI) *m*/*z*: [M + Na]^+^ calcd for C_78_H_146_O_12_Si_5_Na 1437.9558; found 1437.9567; **[α]**_**D**_^**24.0**^ = +8.75 (*c* = 0.16, CHCl_3_); **R**_*f*_ = 0.44 (petroleum ether:EtOAc 19:1).

#### (4*S*,5*S*,*E*)-5-Hydroxy-6-((4*S*,5*R*,6*S*)-2-(4-methoxyphenyl)-5-methyl-6-((5*R*,6*R*)-3,3,9,9-tetraethyl-5-((*S*,1*E*,4*Z*)-3-methylhexa-1,4-dien-1-yl)-6-((triethylsilyl)oxy)-4,8-dioxa-3,9-disilaundecan-6-yl)-1,3-dioxan-4-yl)-2,4-dimethylhex-2-en-1-yl pivalate (**53**)

The glassware was neither dried nor purged with inert gas prior to usage. 2,3-Dichloro-5,6-dicyano-1,4-benzoquinone (9.0 mg, 39.9 μmol, 2.00 equiv) was added to a solution of acetonide **49** (20.0 mg, 19.9 μmol, 1.00 equiv) in CH_2_Cl_2_ (0.90 mL) and an aqueous solution of phosphate buffer (pH = 7, 0.10 mL) at 0 °C. The reaction mixture was stirred for 10 min at 0 °C and for 70 min at room temperature before the subsequent addition of a saturated aqueous solution of NaHCO_3_ and saturated aqueous solution of Na_2_S_2_O_3_ and MTBE. The phases were separated, and the aqueous layer was extracted with MTBE (3×). The combined organic layers were washed with brine, dried over MgSO_4_, and concentrated *in vacuo*. The crude product was purified via column chromatography (petroleum ether:EtOAc 10:1) providing PMP-acetal **53** (13.6 mg, 14.1 μmol, 71%) as a colorless oil. ^**1**^**H NMR (400 MHz, CDCl**_**3**_**)** δ = 7.41–7.38 (m, 2H), 6.89–6.85 (m, 2H), 5.74 (ddd, *J* = 15.6, 9.4, 1.0 Hz, 1H), 5.45–5.37 (m, 2H), 5.31 (s, 1H), 5.28 (d, *J* = 10.1 Hz, 1H), 5.22–5.15 (m, 1H), 4.43 (s, 2H), 4.40 (d, *J* = 9.4 Hz, 1H), 4.01–3.98 (m, 2H), 3.86 (d, *J* = 9.4 Hz, 1H), 3.81 (s, 3H), 3.66–3.61 (m, 1H), 3.53 (d, *J* = 9.4 Hz, 1H), 3.20–3.11 (m, 1H), 2.53–2.44 (m, 1H), 1.92–1.85 (m, 1H), 1.69–1.64 (m, 1H), 1.66 (d, *J* = 0.9 Hz, 3H), 1.62 (dd, *J* = 6.8, 1.7 Hz, 3H), 1.24–1.17 (m, 1H), 1.20 (s, 9H), 1.13 (d, *J* = 6.8 Hz, 3H), 1.02 (d, *J* = 6.7 Hz, 3H), 1.00 (d, *J* = 6.7 Hz, 3H), 0.99–0.85 (m, 27H), 0.64–0.51 (m, 18H) ppm; ^**13**^**C{1H}-NMR (100 MHz, CDCl**_**3**_**)** δ = 178.4, 159.8, 137.4, 134.5, 132.1, 131.2, 130.9, 128.8, 127.8, 122.9, 113.4, 102.2, 82.5, 81.9, 79.0, 78.7, 72.5, 69.8, 62.8, 55.4, 39.0, 38.8, 37.7, 34.4, 34.2, 27.3, 20.5, 16.4, 14.4, 13.0, 8.6, 7.3, 7.3, 7.1, 6.7, 6.2, 4.5 ppm; **HRMS** (ESI) *m*/*z*: [M + Na]^+^ calcd for C_53_H_96_O_9_Si_3_Na 983.6260; found 983.6257; **[α]**_**D**_^**23.7**^ = +33.33 (*c* = 0.03, CHCl_3_); **R**_*f*_ = 0.27 (petroleum ether:EtOAc 9:1).

#### (2*S*,3*S*,4*R*)-2-Hydroxy-2-((1*R*,2*E*,4*S*,5*Z*)-1-hydroxy-4-methylhepta-2,5-dien-1-yl)-3-((4-methoxybenzyl)oxy)-4-methylpentane-1,5-diyl bis(2,2-Dimethylpropanoate) (**55a**)

Pyridine (1.6 mL, 19.6 mmol, 5.00 equiv) and pivaloyl chloride (0.63 mL, 5.10 mmol, 1.30 equiv) were added successively to a solution of triol **45** (1.88 g, 3.92 mmol, 1.00 equiv) in CH_2_Cl_2_ (20.0 mL) at 0 °C. The reaction mixture was stirred for 10 min at 0 °C and for 12.5 h at room temperature before water was added. The phases were separated and the aqueous layer was extracted with CH_2_Cl_2_ (3×). The combined organic layers were successively washed with an aqueous solution of KHSO_4_ (1 M), a saturated aqueous solution of NaHCO_3_ and brine, dried over MgSO_4_, and concentrated *in vacuo*. The crude product was purified via column chromatography (petroleum ether:EtOAc 4.5:1) providing a mixture of pivalates **55a** and **55b** (1.73 g, 3.07 mmol, 78%, **55a**:**55b** = 10:1) as a colorless oil. Analytical data are given for a mixture of pivalates **55a** and **55b** (rr = 6:1, determined via^1^H NMR). Chemical shift values are given for pivalate **55a**, if possible. ^**1**^**H NMR (400 MHz, CDCl**_**3**_**)** δ = 7.27–7.24 (m, 2H), 6.90–6.87 (m, 2H), 5.70 (dd, *J* = 15.8, 5.9 Hz, 1H), 5.54 (ddd, *J* = 15.8, 7.0, 0.9 Hz, 1H), 5.47–5.36 (m, 1H), 5.21–5.15 (m, 1H), 4.61 (d, *J* = 10.7 Hz, 1H), 4.49 (d, *J* = 10.7 Hz, 1H), 4.22 (dd, *J* = 7.0, 4.4 Hz, 1H), 4.10 (d, *J* = 11.8 Hz, 1H), 4.02–3.99 (m, 3H), 3.81 (s, 3H), 3.78 (d, *J* = 2.2 Hz, 1H), 3.23–3.14 (m, 1H), 3.06 (s, 1H), 2.89–2.87 (m, 1H), 2.40–2.30 (m, 1H), 1.59 (dd, *J* = 6.8, 1.7 Hz, 3H), 1.22 (s, 9H), 1.19 (s, 9H), 1.06 (d, *J* = 7.0 Hz, 3H), 1.04 (d, *J* = 7.0 Hz, 3H) ppm; ^**13**^**C{1H}-NMR (100 MHz, CDCl**_**3**_**)** δ = 178.5, 178.0, 159.7, 139.4, 134.0, 129.7, 129.6, 125.0, 123.6, 114.2, 80.1, 77.1, 75.3, 74.7, 68.2, 65.0, 55.4, 39.0, 38.9, 34.2, 33.8, 27.4, 27.4, 20.8, 13.1, 12.3 ppm; **HRMS** ESI *m*/*z*: [M + Na]^+^ calcd for C_32_H_50_O_8_Na 585.3403; found 585.3406; **[α]**_**D**_^**22.5**^ = +70.0 (*c* = 1.00, CHCl_3_); **R**_*f*_ = 0.24 (petroleum ether:EtOAc 5:1).

#### (2*R*,3*S*,4*R*)-3-((4-Methoxybenzyl)oxy)-4-methyl-2-((1*R*,2*E*,4*S*,5*Z*)-4-methyl-1-((triethylsilyl)oxy)hepta-2,5-dien-1-yl)-2-((triethylsilyl)oxy)pentane-1,5-diyl bis(2,2-Dimethylpropanoate) (**88**)

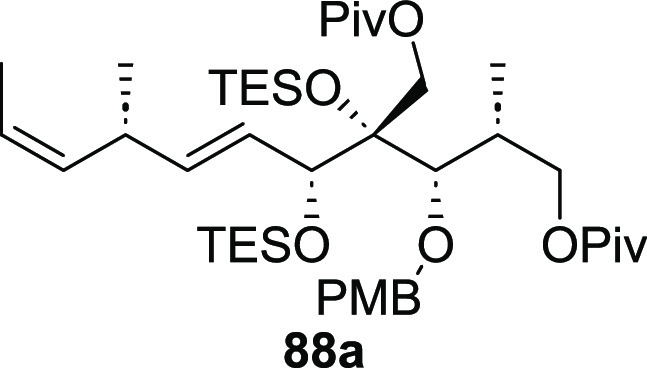



2,6-Lutidine (5.3 mL, 45.3 mmol, 15.0 equiv) and trimethylsilyl trifluoromethanesulfonate (5.1 mL, 22.7 mmol, 7.50 equiv) were added successively to a solution of pivalates **55a** and **55b** (1.70 g, 3.02 mmol, 1.00 equiv, **55a**:**55b** = 10:1) in CH_2_Cl_2_ (15.0 mL) at −78 °C. The reaction mixture was stirred for 15 min at −78 °C and for 14.5 h at room temperature before it was cooled to 0 °C and a saturated aqueous solution of NaHCO_3_ was added. The phases were separated and the aqueous layer was extracted with CH_2_Cl_2_ (3×). The combined organic layers were successively washed with an aqueous solution of KHSO_4_ (1 M), a saturated aqueous solution of NaHCO_3_ and brine, dried over MgSO_4_, and concentrated *in vacuo*. The crude product was purified via column chromatography (petroleum ether:EtOAc 20:1) providing a mixture of TES-ethers **88a** and **88b** (2.67 g, 2.99 mmol, 99%, **88a**:**88b** = 10:1) as a colorless oil. Analytical data are given for a mixture of TES-ethers **88a** and **88b** (rr = 10:1, determined via^1^H NMR). Chemical shift values are given for TES-ether **88a**, if possible. ^**1**^**H NMR (400 MHz, CDCl**_**3**_**)** δ = 7.22–7.20 (m, 2H), 6.86–6.84 (m, 2H), 5.59–5.49 (m, 2H), 5.44–5.37 (m, 1H), 5.18–5.12 (m, 1H), 4.48 (d, *J* = 10.7 Hz, 1H), 4.44 (d, *J* = 7.4 Hz, 1H), 4.40 (d, *J* = 10.7 Hz, 1H), 4.20 (d, *J* = 11.8 Hz, 1H), 4.14 (d, *J* = 11.8 Hz, 1H), 4.03 (dd, *J* = 10.5, 5.0 Hz, 1H), 3.96–3.91 (m, 1H), 3.80 (s, 3H), 3.56 (d, *J* = 2.9 Hz, 1H), 3.22–3.14 (m, 1H), 2.66–2.58 (m, 1H), 1.60 (dd, *J* = 6.8, 1.7 Hz, 3H), 1.23 (s, 9H), 1.21 (s, 9H), 1.03–1.01 (m, 6H), 0.92 (t, *J* = 7.9 Hz, 18H), 0.66–0.54 (m, 12H) ppm; ^**13**^**C{1H}-NMR (100 MHz, CDCl**_**3**_**)** δ = 178.6, 178.3, 158.9, 139.0, 133.9, 131.1, 128.4, 127.1, 123.2, 113.7, 82.2, 81.4, 74.0, 69.7, 65.8, 55.4, 39.0, 38.9, 34.1, 33.6, 27.5, 27.4, 20.4, 13.0, 12.7, 7.4, 7.0, 6.8, 5.7 ppm (1 carbon atom is overlapped by CDCl_3_); **HRMS** (ESI) *m*/*z*: [M + Na]^+^ calcd for C_44_H_78_O_8_Si_2_Na 813.5133; found 813.5135; **[α]**_**D**_^**22.4**^ = +43.3 (*c* = 0.30, CHCl_3_); **R**_*f*_ = 0.22 (petroleum ether:EtOAc 19:1).

#### (2*R*,3*S*,4*R*)-3-((4-Methoxybenzyl)oxy)-4-methyl-2-((1*R*,2*E*,4*S*,5*Z*)-4-methyl-1-((triethylsilyl)oxy)hepta-2,5-dien-1-yl)-2-((triethylsilyl)oxy)pentane-1,5-dio*l* (**89**)

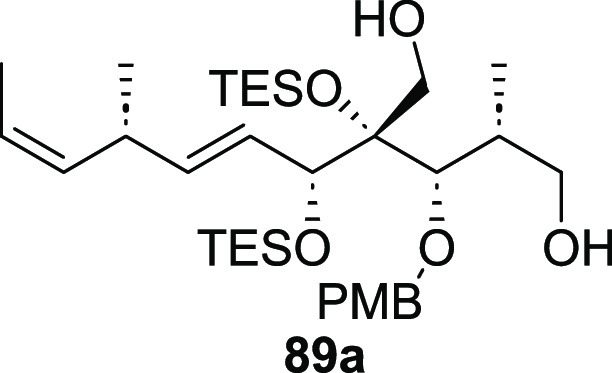



Diisobutylaluminum hydride (1 M in hexane, 15.0 mL, 15.0 mmol, 5.00 equiv) was added dropwise to a solution of TES-ethers **88a** and **88b** (2.35 g, 2.96 mmol, 1.00 equiv, **88a**:**88b** = 10:1) in CH_2_Cl_2_ (15.0 mL) at −78 °C. The reaction mixture was stirred for 1 h before MeOH (5.0 mL) was added. The resulting slurry was diluted with MTBE (100 mL) and poured into a vigorously stirred solution of Rochelle salt (100 mL) at room temperature. Stirring was continued until two phases were formed (1 h). The phases were separated, and the aqueous layer was extracted with MTBE (3×). The combined organic layers were washed with brine, dried over MgSO_4_, and concentrated *in vacuo*. The crude product was purified via column chromatography (petroleum ether:EtOAc 10:1) providing a mixture of diols **89a** and **89b** (1.84 g, 2.96 mmol, 100%, **89a**:**89b** = 10:1) as a colorless oil. Analytical data are given for a mixture of diols **89a** and **89b** (rr = 10:1, determined via^1^H NMR). Chemical shift values are given for diol **89a**, if possible. ^**1**^**H NMR (500 MHz, CDCl**_**3**_**)** δ = 7.24–7.21 (m, 2H), 6.87–6.84 (m, 2H), 5.70 (ddd, *J* = 15.6, 6.1, 1.4 Hz, 1H), 5.58 (ddd, *J* = 15.6, 5.6, 1.2 Hz, 1H), 5.49–5.40 (m, 1H), 5.24–5.19 (m, 1H), 4.80 (d, *J* = 11.0 Hz, 1H), 4.47 (d, *J* = 11.0 Hz, 1H), 4.16 (d, *J* = 10.5 Hz, 1H), 4.13 (d, *J* = 6.1 Hz, 1H), 3.80 (s, 3H), 3.79 (d, *J* = 11.4 Hz, 1H), 3.66–3.62 (m, 1H), 3.53–3.51 (m, 1H), 3.39–3.36 (m, 1H), 3.33–3.28 (m, 1H), 3.26–3.19 (m, 1H), 2.42–2.35 (m, 1H), 1.64 (dd, *J* = 6.9, 1.8 Hz, 3H), 1.28 (bs, 1H), 1.07 (d, *J* = 6.7 Hz, 3H), 1.01–0.98 (m, 9H), 0.95–0.92 (m, 12H), 0.75–0.70 (m, 6H), 0.61–0.56 (m, 6H) ppm; ^**13**^**C{1H}-NMR (125 MHz, CDCl**_**3**_**)** δ = 159.3, 136.8, 134.3, 131.1, 129.3, 126.9, 123.4, 113.9, 84.4, 82.2, 78.0, 75.6, 67.7, 67.2, 55.4, 35.9, 34.0, 20.5, 13.1, 11.1, 7.6, 7.1, 7.0, 5.2 ppm; **HRMS** (ESI) *m*/*z*: [M + Na]^+^ calcd for C_34_H_62_O_6_Si_2_Na 645.3983; found 645.3978; **[α]**_**D**_^**20.0**^ = +40.3 (*c* = 0.12, CHCl_3_); **R**_*f*_ = 0.44 (petroleum ether:EtOAc 5:1).

#### (2*R*,3*R*,4*E*,6*S*,7*Z*)-2-((4*S*,5*R*)-2-(4-Methoxyphenyl)-5-methyl-1,3-dioxan-4-yl)-6-methyl-2,3-bis((triethylsilyl)oxy)nona-4,7-dien-1-ol (**90**)

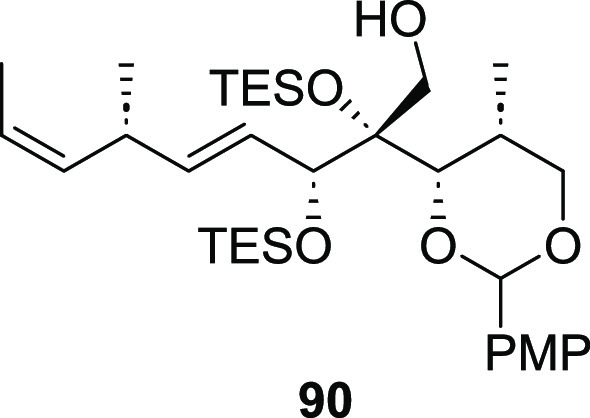



A suspension of diols **89a** and **89b** (1.84 g, 2.96 mmol, 1.00 equiv, **89a**:**89b** = 10:1) and activated molecular sieves (4 Å, powdered, 4.20 g) in CH_2_Cl_2_ (120 mL) was stirred for 1 h at room temperature before it was cooled to 0 °C. 2,3-Dichloro-5,6-dicyano-1,4-benzoquinone (1.58 g, 7.40 mmol, 2.50 equiv) was added, and the reaction mixture was stirred for 45 min before the subsequent addition of a saturated aqueous solution of NaHCO_3_ and saturated aqueous solution of Na_2_S_2_O_3_. The phases were separated and the aqueous layer was extracted with CH_2_Cl_2_ (3×). The combined organic layers were washed with brine, dried over MgSO_4_, and concentrated *in vacuo*. The crude product was purified via column chromatography (petroleum ether:EtOAc 9:1) providing PMP-acetal **90** (1.64 g, 2.64 mmol, 89%, mixture of at least two PMP-acetals, ratio not determined) as a colorless oil. Analytical data are given for a pure sample of the major diastereomer. ^**1**^**H NMR (400 MHz, CDCl**_**3**_**)** δ = 7.41–7.37 (m, 2H), 6.89–6.86 (m, 2H), 5.64–5.54 (m, 2H), 5.48–5.40 (m, 1H), 5.39 (s, 1H), 5.24–5.18 (m, 1H), 4.17 (d, *J* = 5.4 Hz, 1H), 4.09 (d, *J* = 2.0 Hz, 1H), 3.94 (dd, *J* = 11.0, 1.9 Hz, 1H), 3.90 (dd, *J* = 11.0, 1.7 Hz, 1H), 3.84–3.81 (m, 1H), 3.81 (s, 3H), 3.71 (dd, *J* = 11.3, 9.6 Hz, 1H), 3.26–3.18 (m, 1H), 2.33 (dd, *J* = 9.5, 2.3 Hz, 1H), 2.02–1.97 (m, 1H), 1.64 (dd, *J* = 6.8, 1.8 Hz, 3H), 1.32 (d, *J* = 6.9 Hz, 3H), 1.07 (d, *J* = 6.8 Hz, 3H), 0.95 (t, *J* = 7.8 Hz, 9H), 0.85 (t, *J* = 7.9 Hz, 9H), 0.61 (q, *J* = 7.8 Hz, 6H), 0.55–0.48 (m, 6H) ppm; ^**13**^**C{1H}-NMR (100 MHz, CDCl**_**3**_**)** δ = 160.2, 137.3, 134.2, 131.2, 127.9, 126.9, 123.3, 113.7, 103.4, 84.8, 81.5, 76.5, 75.9, 63.9, 55.4, 34.1, 30.5, 20.6, 14.0, 13.1, 7.3, 7.0, 6.7, 5.3 ppm; **HRMS** (ESI) *m*/*z*: [M + Na]^+^ calcd for C_34_H_60_O_6_Si_2_Na 643.3826; found 643.3831; **[α]**_**D**_^**20.4**^ = +77.4 (*c* = 0.31, CHCl_3_); **R**_*f*_ = 0.53 (petroleum ether:EtOAc 9:1).

#### (9*R*,10*R*)-12,12-Diethyl-9-((4*S*,5*R*)-2-(4-methoxyphenyl)-5-methyl-1,3-dioxan-4-yl)-2,2-dimethyl-10-((*S*,1*E*,4*Z*)-3-methylhexa-1,4-dien-1-yl)-9-((triethylsilyl)oxy)-5,7,11-trioxa-2,12-disilatetradecane (**56**)

*N*,*N*-Diisopropylethylamine (2.3 mL, 13.2 mmol, 5.00 equiv) and 2-(trimethylsilyl)ethoxymethyl chloride (1.2 mL, 6.60 mmol, 2.50 equiv) were added successively to a solution of PMP-acetal **90** (1.64 g, 2.64 mmol, 1.00 equiv, mixture of at least two PMP-acetals, ratio not determined) in CH_2_Cl_2_ (15.0 mL) at 0 °C. The reaction mixture was stirred for 5 min at 0 °C and for 15.5 h at room temperature before it was cooled to 0 °C and *N*,*N*-diisopropylethylamine (1.2 mL, 7.06 mmol, 2.67 equiv) and 2-(trimethylsilyl)ethoxymethyl chloride (0.60 mL, 3.38 mmol, 1.28 equiv) were added successively. The reaction mixture was warmed to room temperature and stirred for 5 h before it was cooled to 0 °C, and 2-(trimethylsilyl)ethoxymethyl chloride (0.20 mL, 1.13 mmol, 0.43 equiv) was added. The reaction mixture was warmed to room temperature and stirred for 40 min before a saturated aqueous solution of NaHCO_3_ was added. The phases were separated and the aqueous layer was extracted with CH_2_Cl_2_ (3×). The combined organic layers were washed with brine, dried over MgSO_4_, and concentrated *in vacuo*. The crude product was purified via column chromatography (petroleum ether:EtOAc 20:1) providing SEM-ether **56** (1.37 g, 1.83 mmol, 69%, mixture with hydrolyzed 2-(trimethylsilyl)ethoxymethyl chloride, ratio 1:0.5, 1.51 g in total, the undesired regioisomer of the pivalate protection could be fully separated at this stage) as a colorless oil. SEM-ether **56** was used in the next reaction without detailed characterization.

#### (2*R*,3*S*,4*R*,5*R*,6*E*,8*S*,9*Z*)-3-((4-Methoxybenzyl)oxy)-2,8-dimethyl-4,5-bis((triethylsilyl)oxy)-4-(((2-(trimethylsilyl)ethoxy)methoxy)methyl)undeca-6,9-dien-1-ol (**91**)

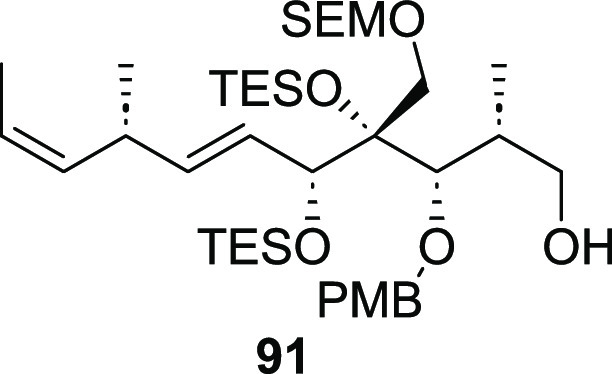



Diisobutylaluminum hydride (1 M in hexane, 6.5 mL, 6.5 mmol, 3.50 equiv) was added dropwise to a solution of SEM-ether **56** (1.37 g, 1.83 mmol, 1.00 equiv, mixture with hydrolyzed 2-(trimethylsilyl)ethoxymethyl chloride, ratio 1:0.5, 1.51 g in total) in CH_2_Cl_2_ (12.0 mL) at −78 °C. The reaction mixture was stirred for 10 min at −78 °C and for 45 min at 0 °C before MeOH (2.0 mL) was added. The resulting slurry was diluted with MTBE (100 mL) and poured into a vigorously stirred solution of Rochelle salt (100 mL) at room temperature. Stirring was continued until two phases were formed (30 min). The phases were separated and the aqueous layer was extracted with MTBE (3×). The combined organic layers were washed with brine, dried over MgSO_4_, and concentrated *in vacuo*. The crude product was purified via column chromatography (petroleum ether:EtOAc 9:1) providing alcohol **91** (1.15 g, 1.52 mmol, 84%) as a colorless oil. ^**1**^**H NMR (400 MHz, CDCl**_**3**_**)** δ = 7.25–7.22 (m, 2H), 6.87–6.83 (m, 2H), 5.65 (ddd, *J* = 15.6, 7.2, 1.2 Hz, 1H), 5.55 (dd, *J* = 15.6, 5.4 Hz, 1H), 5.47–5.39 (m, 1H), 5.24–5.18 (m, 1H), 4.72 (d, *J* = 11.3 Hz, 1H), 4.69 (d, *J* = 6.2 Hz, 1H), 4.67 (d, *J* = 6.2 Hz, 1H), 4.48 (d, *J* = 11.3 Hz, 1H), 4.38 (d, *J* = 7.3 Hz, 1H), 3.80 (s, 3H), 3.80 (d, *J* = 9.9 Hz, 1H), 3.76 (d, *J* = 9.9 Hz, 1H), 3.72–3.59 (m, 3H), 3.42–3.31 (m, 2H), 3.24–3.16 (m, 1H), 2.43–2.35 (m, 1H), 1.63 (dd, *J* = 6.9, 1.8 Hz, 3H), 1.06 (d, *J* = 6.8 Hz, 3H), 0.97–0.92 (m, 20H), 0.88 (d, *J* = 6.7 Hz, 3H), 0.70–0.63 (m, 6H), 0.62–0.55 (m, 6H), 0.02 (s, 9H) ppm; ^**13**^**C{1H}-NMR (100 MHz, CDCl**_**3**_**)** δ = 158.9, 137.3, 134.4, 132.0, 128.9, 127.5, 123.1, 113.6, 96.3, 83.9, 82.2, 75.9, 74.7, 69.0, 68.2, 66.6, 55.4, 36.2, 34.1, 20.4, 18.3, 13.0, 12.1, 7.7, 7.1, 7.1, 5.5, −1.3 ppm; **HRMS** (ESI) *m*/*z*: [M + Na]^+^ calcd for C_40_H_76_O_7_Si_3_Na 775.4797; found 775.4793; **[α]**_**D**_^**23.8**^ = +33.6 (*c* = 0.14, CHCl_3_); **R**_*f*_ = 0.45 (petroleum ether:EtOAc 9:1).

#### (2*S*,3*S*,4*R*,5*R*,6*E*,8*S*,9*Z*)-3-((4-Methoxybenzyl)oxy)-2,8-dimethyl-4,5-bis((triethylsilyl)oxy)-4-(((2-(trimethylsilyl)ethoxy)methoxy)methyl)undeca-6,9-dienal (**57**)

A suspension of alcohol **91** (612 mg, 0.81 mmol, 1.00 equiv), *N*-methylmorpholine *N*-oxide (286 mg, 2.44 mmol, 3.00 equiv), and activated molecular sieves (4 Å, powdered, 714 mg) in CH_2_Cl_2_ (4.0 mL) was stirred for 1 h at room temperature before tetrapropylammonium perruthenate (20.0 mg, 0.06 mmol, 0.07 equiv) was added. The reaction mixture was stirred for 1.5 h before it was diluted with petroleum ether (4.0 mL) and charged on a chromatography column. Purification via column chromatography (petroleum ether:EtOAc 20:1) provided southwestern fragment **57** (520 mg, 0.69 mmol, 85%) as a colorless oil. Southwestern fragment **57** was used in the next reaction without detailed characterization.

#### (2*E*,4*S*,7*S*,8*R*,9*S*,10*R*,11*R*,12*E*,14*S*,15*Z*)-7-Hydroxy-9-((4-methoxybenzyl)oxy)-2,4,8,14-tetramethyl-5-oxo-10,11-bis((triethylsilyl)oxy)-10-(((2-(trimethylsilyl)ethoxy)methoxy)methyl)heptadeca-2,12,15-trien-1-yl Pivalate (**58**)

A solution of eastern fragment **15** (357 mg, 1.58 mmol, 2.29 equiv) in THF (2.0 mL) was added dropwise to a solution of lithium bis(trimethylsilyl)amide (1 M in THF, 1.4 mL, 1.40 mmol, 2.00 equiv) at −78 °C. The reaction mixture was stirred for 1.5 h before a solution of southwestern fragment **57** (520 mg, 0.69 mmol, 1.00 equiv) in THF (3.5 mL) was added. The reaction mixture was stirred before the subsequent addition of a saturated aqueous solution of NH_4_Cl (6.0 mL) and MTBE (6.0 mL). The phases were separated, and the aqueous layer was extracted with MTBE (3×). The combined organic layers were successively washed with a saturated aqueous solution of NaHCO_3_ and brine, dried over MgSO_4_, and concentrated *in vacuo*. The crude product was purified via column chromatography (petroleum ether:EtOAc 10:1) providing nonreacted southwestern fragment **57** (95.0 mg, 0.13, 18%) and alcohol **58** (372 mg, 0.38 mmol, 55%, 67% brsm, mixture with eastern fragment **15**, 411 mg in total) as colorless oils. Alcohol **58** was used in the next reaction without detailed characterization.

#### (2*E*,4*S*,5*S*,7*S*,8*R*,9*S*,10*R*,11*R*,12*E*,14*S*,15*Z*)-5,7-Dihydroxy-9-((4-methoxybenzyl)oxy)-2,4,8,14-tetramethyl-10,11-bis((triethylsilyl)oxy)-10-(((2-(trimethylsilyl)ethoxy)methoxy)methyl)heptadeca-2,12,15-trien-1-yl pivalate (**92**)

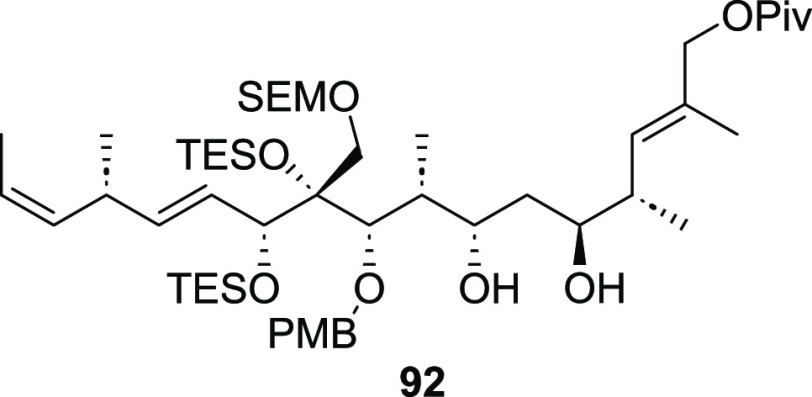



Acetic acid (21.5 mL) was added to a suspension of tetramethylammonium triacetoxyborohydride (930 mg, 3.52 mmol, 8.20 equiv) in MeCN (21.5 mL) at 0 °C. The reaction mixture was stirred for 1 h before a solution of alcohol **58** (420 mg, 0.43 mmol, 1.00 equiv) in MeCN (9.0 mL) was added. The reaction mixture was stirred for 14.5 h before a solution of Rochelle salt was added. The reaction mixture was warmed to room temperature and diluted with CH_2_Cl_2_, and Na_2_CO_3_ (25.0 g) was added. The phases were separated, and the aqueous layer was extracted with CH_2_Cl_2_ (3×). The combined organic layers were washed with brine, dried over MgSO_4_, and concentrated *in vacuo*. The crude product was purified via column chromatography (petroleum ether:EtOAc 5:1) providing diol **92** (283 mg, 289 μmol, 67%) as a colorless oil. ^**1**^**H NMR (600 MHz, CDCl**_**3**_**)** δ = 7.24–7.21 (m, 2H), 6.86–6.83 (m, 2H), 5.63 (ddd, *J* = 15.6, 6.2, 1.1 Hz, 1H), 5.57 (dd, *J* = 15.6, 5.3 Hz, 1H), 5.47–5.42 (m, 1H), 5.29–5.26 (m, 1H), 5.23–5.19 (m, 1H), 4.93 (d, *J* = 11.2 Hz, 1H), 4.70 (d, *J* = 6.2 Hz, 1H), 4.68 (d, *J* = 6.2 Hz, 1H), 4.47 (d, *J* = 11.2 Hz, 1H), 4.43 (d, *J* = 12.9 Hz, 1H), 4.40 (d, *J* = 12.9 Hz, 1H), 4.36 (d, *J* = 6.3 Hz, 1H), 3.93 (d, *J* = 10.2 Hz, 1H), 3.84 (d, *J* = 10.0 Hz, 1H), 3.79 (s, 3H), 3.76 (d, *J* = 1.7 Hz, 1H), 3.73 (d, *J* = 10.0 Hz, 1H), 3.71–3.68 (m, 1H), 3.64–3.60 (m, 2H), 3.24–3.19 (m, 1H), 2.58 (bs, 1H), 2.50–2.44 (m, 1H), 2.31–2.27 (m, 1H), 2.00 (bs, 1H), 1.67–1.63 (m, 1H), 1.65 (d, *J* = 1.5 Hz, 3H), 1.64 (dd, *J* = 6.8, 1.6 Hz, 3H), 1.24–1.23 (m, 1H), 1.19 (s, 9H), 1.07 (d, *J* = 6.6 Hz, 3H), 1.01 (d, *J* = 6.6 Hz, 3H), 0.97–0.92 (m, 20H), 0.87 (d, *J* = 7.0 Hz, 3H), 0.72–0.64 (m, 6H), 0.61–0.57 (m, 6H), 0.02 (s, 9H) ppm; ^**13**^**C{1H}-NMR (150 MHz, CDCl**_**3**_**)** δ = 178.4, 159.1, 137.4, 134.4, 131.8, 131.5, 130.7, 128.9, 126.9, 123.3, 113.9, 96.4, 88.3, 84.0, 75.2, 74.6, 74.0, 72.7, 70.1, 68.9, 66.7, 55.4, 39.3, 39.0, 38.9, 38.3, 34.1, 27.4, 20.6, 18.4, 16.5, 14.4, 13.1, 8.3, 7.8, 7.3, 7.1, 5.4, −1.3 ppm; **HRMS** (ESI) *m*/*z*: [M + Na]^+^ calcd for C_53_H_98_O_10_Si_3_Na 1001.6366; found 1001.6364; **[α]**_**D**_^**19.4**^ = +29.4 (*c* = 0.17, CHCl_3_); **R**_*f*_ = 0.41 (petroleum ether:EtOAc 5:1).

#### (2*E*,4*S*,5*S*,7*S*,8*S*,9*S*,10*R*,11*R*,12*E*,14*S*,15*Z*)-9-((4-Methoxybenzyl)oxy)-2,4,8,14-tetramethyl-5,7,10,11-tetrakis((triethylsilyl)oxy)-10-(((2-(trimethylsilyl)ethoxy)methoxy)methyl)heptadeca-2,12,15-trien-1-yl Pivalate (**93**)

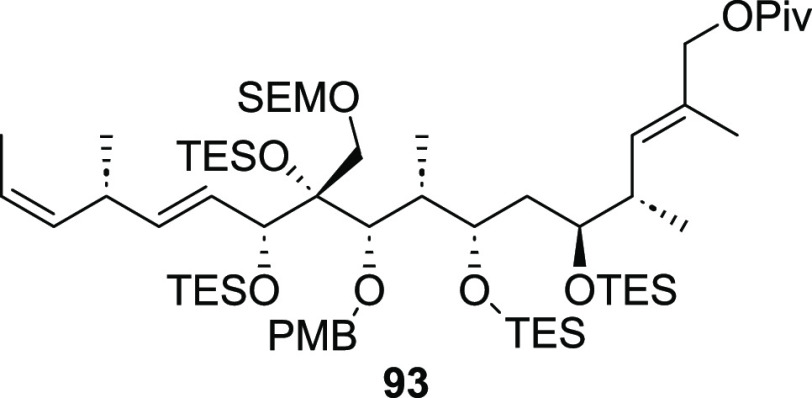



2,6-Lutidine (0.34 mL, 2.88 mmol, 10.0 equiv) and trimethylsilyl trifluoromethanesulfonate (0.33 mL, 1.44 mmol, 5.00 equiv) were added successively to a solution of diol **92** (282 mg, 0.29 mmol, 1.00 equiv) in CH_2_Cl_2_ (2.9 mL) at −78 °C. The reaction mixture was stirred for 5 min at −78 °C and for 40 min at 0 °C before a saturated aqueous solution of NaHCO_3_ was added. The phases were separated and the aqueous layer was extracted with CH_2_Cl_2_ (3×). The combined organic layers were successively washed with an aqueous solution of KHSO_4_ (1 M), a saturated aqueous solution of NaHCO_3_ and brine, dried over MgSO_4_, and concentrated *in vacuo*. The crude product was purified via column chromatography (petroleum ether:EtOAc 50:1) providing TES-ether **93** (326 mg, 0.27 mmol, 94%) as a colorless oil. ^**1**^**H NMR (500 MHz, CDCl**_**3**_**)** δ = 7.25–7.22 (m, 2H), 6.86–6.83 (m, 2H), 5.68 (ddd, *J* = 15.6, 9.7, 1.5 Hz, 1H), 5.52 (d, *J* = 8.4 Hz, 1H), 5.40–5.33 (m, 2H), 5.16–5.11 (m, 1H), 4.69 (d, *J* = 12.2 Hz, 1H), 4.65 (d, *J* = 6.4 Hz, 1H), 4.60 (d, *J* = 6.4 Hz, 1H), 4.60 (d, *J* = 9.7 Hz, 1H), 4.45 (d, *J* = 12.0 Hz, 1H), 4.37 (d, *J* = 12.0 Hz, 1H), 4.17 (d, *J* = 12.2 Hz, 1H), 3.89 (d, *J* = 9.7 Hz, 1H), 3.80 (s, 3H), 3.73–3.68 (m, 2H), 3.65–3.61 (m, 2H), 3.59 (d, *J* = 2.7 Hz, 1H), 3.30 (d, *J* = 9.7 Hz, 1H), 3.15–3.08 (m, 1H), 2.41–2.34 (m, 1H), 2.19–2.14 (m, 1H), 1.82–1.72 (m, 2H), 1.70 (d, *J* = 1.1 Hz, 3H), 1.56 (dd, *J* = 6.8, 1.8 Hz, 3H), 1.21 (s, 9H), 1.00–0.89 (m, 47H), 0.68–0.53 (m, 24H), 0.02 (s, 9H) ppm; ^**13**^**C{1H}-NMR (125 MHz, CDCl**_**3**_**)** δ = 178.6, 158.3, 138.2, 134.3, 133.8, 132.7, 128.8, 128.7, 127.3, 122.7, 113.5, 96.0, 84.0, 79.3, 79.0, 71.6, 71.4 (2 different carbon atoms), 70.8, 69.7, 66.2, 55.4, 40.8, 39.0, 37.9, 36.1, 34.0, 27.3, 20.0, 18.3, 14.8, 13.3, 12.9, 9.0, 7.5, 7.3, 7.2, 7.1, 6.9, 6.1, 5.7, 5.4, −1.3 ppm; **HRMS** (ESI) *m*/*z*: [M + Na]^+^ calcd for C_65_H_126_O_10_Si_5_Na 1229.8095; found 1229.8102; **[α]**_**D**_^**21.0**^ = +64.3 (*c* = 0.14, CHCl_3_); **R**_*f*_ = 0.53 (petroleum ether:EtOAc 19:1).

#### (2*E*,4*S*,5*S*,7*S*,8*S*,9*S*,10*R*,11*R*,12*E*,14*S*,15*Z*)-9-((4-Methoxybenzyl)oxy)-2,4,8,14-tetramethyl-5,7,10,11-tetrakis((triethylsilyl)oxy)-10-(((2-(trimethylsilyl)ethoxy)methoxy)methyl)heptadeca-2,12,15-trien-1-ol (**94**)

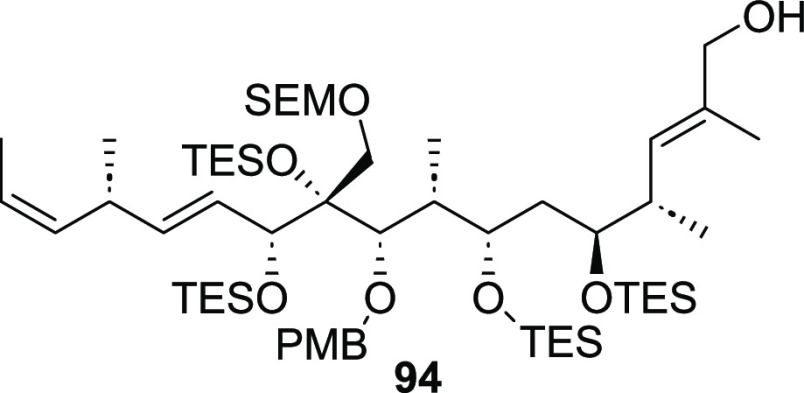



Diisobutylaluminum hydride (1 M in hexane, 0.73 mL, 730 μmol, 2.70 equiv) was added dropwise to a solution of TES-ether **93** (325 mg, 269 μmol, 1.00 equiv) in CH_2_Cl_2_ (2.0 mL) at −78 °C. The reaction mixture was stirred for 1.25 h before MeOH (0.50 mL) was added. The resulting slurry was diluted with MTBE (75 mL) and poured into a vigorously stirred solution of Rochelle salt (75 mL) at room temperature. Stirring was continued until two phases were formed (15 min). The phases were separated and the aqueous layer was extracted with MTBE (3×). The combined organic layers were washed with brine, dried over MgSO_4_, and concentrated *in vacuo*. The crude product was purified via column chromatography (petroleum ether:EtOAc 10:1) providing alcohol **94** (287 mg, 255 μmol, 95%) as a colorless oil. ^**1**^**H NMR (400 MHz, CDCl**_**3**_**)** δ = 7.25–7.22 (m, 2H), 6.86–6.83 (m, 2H), 5.71 (ddd, *J* = 15.6, 9.6, 1.4 Hz, 1H), 5.45 (d, *J* = 8.6 Hz, 1H), 5.41–5.31 (m, 1H), 5.38 (dd, *J* = 15.6, 5.7 Hz, 1H), 5.17–5.10 (m, 1H), 4.68 (d, *J* = 12.2 Hz, 1H), 4.65 (d, *J* = 6.4 Hz, 1H), 4.60 (d, *J* = 6.4 Hz, 1H), 4.57 (d, *J* = 9.5 Hz, 1H), 4.22 (d, *J* = 12.2 Hz, 1H), 3.95 (d, *J* = 4.4 Hz, 2H), 3.86 (d, *J* = 9.6 Hz, 1H), 3.80 (s, 3H), 3.71–3.57 (m, 5H), 3.31 (d, *J* = 9.6 Hz, 1H), 3.15–3.06 (m, 1H), 2.42–2.34 (m, 1H), 2.19–2.12 (m, 1H), 1.75 (t, *J* = 7.0 Hz, 2H), 1.71 (d, *J* = 1.2 Hz, 3H), 1.56 (dd, *J* = 6.6, 1.9 Hz, 3H), 1.34–1.31 (bm, 1H), 1.01–0.89 (m, 47H), 0.69–0.53 (m, 24H), 0.02 (s, 9H) ppm; ^**13**^**C{1H}-NMR (100 MHz, CDCl**_**3**_**)** δ = 158.3, 138.3, 134.4, 133.7, 132.8, 130.4, 128.8, 127.4, 122.6, 113.5, 96.0, 84.0, 79.5, 78.8 (HSQC), 72.1 (2 different carbon atoms), 71.5, 69.9, 69.5, 66.3, 55.4, 40.7, 38.2, 36.4, 34.0, 20.1, 18.3, 14.5, 13.9, 12.9, 9.2, 7.5, 7.3, 7.2, 7.1, 7.0, 6.0, 5.7, 5.5, −1.3 ppm; **HRMS** (ESI) *m*/*z*: [M + Na]^+^ calcd for C_60_H_118_O_9_Si_5_Na 1145.7520; found 1145.7533; **[α]**_**D**_^**23.4**^ = +48.7 (*c* = 0.37, CHCl_3_); **R**_*f*_ = 0.59 (petroleum ether:EtOAc 9:1).

#### (2*E*,4*S*,5*S*,7*S*,8*S*,9*S*,10*R*,11*R*,12*E*,14*S*,15*Z*)-9-((4-Methoxybenzyl)oxy)-2,4,8,14-tetramethyl-5,7,10,11-tetrakis((triethylsilyl)oxy)-10-(((2-(trimethylsilyl)ethoxy)methoxy)methyl)heptadeca-2,12,15-trienal (**59**)

A suspension of alcohol **94** (306 mg, 272 μmol, 1.00 equiv), *N*-methylmorpholine *N*-oxide (96.0 mg, 817 μmol, 3.00 equiv), and activated molecular sieves (4 Å, powdered, 239 mg) in CH_2_Cl_2_ (1.8 mL) was stirred for 1 h at room temperature before tetrapropylammonium perruthenate (10.1 mg, 28.7 μmol, 0.11 equiv) was added. The reaction mixture was stirred for 30 min before it was diluted with petroleum ether (4.0 mL) and put onto a chromatography column. Purification via column chromatography (petroleum ether:EtOAc 30:1) provided aldehyde **59** (296 mg, 264 μmol, 97%) as a colorless oil. Aldehyde **59** was used in the next reaction without detailed characterization.

#### Allyl (3*R*,6*R*,7*R*,8*E*,10*S*,11*S*,13*S*,14*S*,15*S*,16*R*,17*R*,18*E*,20*S*,21*Z*)-3-((*tert*-Butyldimethylsilyl)oxy)-7-hydroxy-15-((4-methoxybenzyl)oxy)-4,4,6,8,10,14,20-heptamethyl-5-oxo-11,13,16,17-tetrakis((triethylsilyl)oxy)-16-(((2-(trimethylsilyl)ethoxy)methoxy)methyl)tricosa-8,18,21-trienoate (**95**)

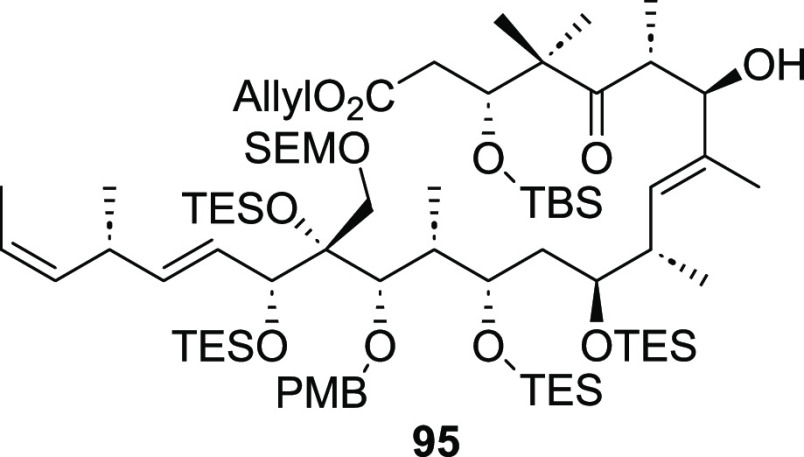



Titanium tetrachloride (1 M in CH_2_Cl_2_, 0.40 mL, 400 μmol, 1.52 equiv) and *N*,*N*-diisopropylethylamine (0.13 mL, 791 μmol, 3.00 equiv) were added successively to a solution of northern fragment **11** (145 mg, 422 μmol, 1.60 equiv) in CH_2_Cl_2_ (2.2 mL) at −78 °C. The reaction mixture was stirred for 70 min before a solution of aldehyde **59** (296 mg, 264 μmol, 1.00 equiv) in CH_2_Cl_2_ (2.7 mL) was added dropwise over the course of 5 min. The reaction mixture was stirred for 10 min at −78 °C and for 4.25 h at −20 °C before an aqueous solution of phosphate buffer (pH = 7) was added. The phases were separated and the aqueous layer was extracted with CH_2_Cl_2_ (3×). The combined organic layers were washed with brine, dried over MgSO_4_ and concentrated *in vacuo*. The crude product was purified via column chromatography (petroleum ether:EtOAc 20:1) providing alcohol **95** (207 mg, 141 μmol, 54%) and its diastereomer (133 mg, 90.8 μmol, 34%, mixture with northern fragment **11**, 189 mg in total) as colorless oils. Alcohol **95** was used in the next reaction without detailed characterization.

#### Allyl (3*R*,6*R*,7*R*,8*E*,10*S*,11*S*,13*S*,14*S*,15*S*,16*R*,17*R*,18*E*,20*S*,21*Z*)-3-((*tert*-Butyldimethylsilyl)oxy)-15-((4-methoxybenzyl)oxy)-4,4,6,8,10,14,20-heptamethyl-5-oxo-11,13,16,17-tetrakis((triethylsilyl)oxy)-7-((triisopropylsilyl)oxy)-16-(((2-(trimethylsilyl)ethoxy)methoxy)methyl)tricosa-8,18,21-trienoate (**60**)

2,6-Lutidine (0.33 mL, 2.83 mmol, 20.0 equiv) and triisopropylsilyl trifluoromethanesulfonate (0.38 mL, 1.41 mmol, 10.0 equiv) were added successively to a solution of alcohol **95** (207 mg, 0.14 mmol, 1.00 equiv) in CH_2_Cl_2_ (1.4 mL) at −78 °C. The reaction mixture was stirred for 10 min at −78 °C and for 4.25 h at room temperature before a saturated aqueous solution of NaHCO_3_ was added. The phases were separated and the aqueous layer was extracted with CH_2_Cl_2_ (3×). The combined organic layers were successively washed with an aqueous solution of KHSO_4_ (1 M), a saturated aqueous solution of NaHCO_3_ and brine, dried over MgSO_4_, and concentrated *in vacuo*. The crude product was purified via column chromatography (petroleum ether:EtOAc 60:1) providing TIPS-ether **60** (209 mg, 0.13 mmol, 91%) as a colorless oil. ^**1**^**H NMR (500 MHz, CDCl**_**3**_**)** δ = 7.25–7.22 (m, 2H), 6.86–6.83 (m, 2H), 5.93–5.85 (m, 1H), 5.71 (ddd, *J* = 15.6, 9.5, 1.4 Hz, 1H), 5.45 (d, *J* = 8.9 Hz, 1H), 5.40 (dd, *J* = 15.6, 5.7 Hz, 1H), 5.39–5.32 (m, 1H), 5.30 (dq, *J* = 17.1, 1.5 Hz, 1H), 5.21 (dq, *J* = 10.4, 1.5 Hz, 1H), 5.17–5.12 (m, 1H), 4.67 (d, *J* = 11.8 Hz, 1H), 4.65 (d, *J* = 6.4 Hz, 1H), 4.59 (d, *J* = 6.4 Hz, 1H), 4.58–4.54 (m, 4H), 4.35 (d, *J* = 5.9 Hz, 1H), 4.23 (d, *J* = 11.8 Hz, 1H), 3.89 (d, *J* = 9.6 Hz, 1H), 3.80 (s, 3H), 3.72–3.59 (m, 5H), 3.29 (d, *J* = 9.6 Hz, 1H), 3.15–3.08 (m, 2H), 2.40–2.28 (m, 3H), 2.16–2.10 (m, 1H), 1.82–1.77 (m, 1H), 1.74–1.68 (m, 1H), 1.65 (d, *J* = 0.9 Hz, 3H), 1.56 (dd, *J* = 6.9, 1.6 Hz, 3H), 1.18 (s, 3H), 1.09 (d, *J* = 6.9 Hz, 3H), 1.07–1.05 (m, 21H), 1.00–0.88 (m, 50H), 0.84 (s, 9H), 0.68–0.54 (m, 24H), 0.06 (s, 3H), 0.02 (s, 12H) ppm; ^**13**^**C{1H}-NMR (100 MHz, CDCl**_**3**_**)** δ = 214.7, 171.9, 158.3, 138.4, 135.7, 134.4, 132.7, 132.2, 131.1, 128.7, 127.5, 122.6, 118.5, 113.4, 96.0, 84.0, 80.0, 79.3, 78.8, 72.1 (2 different carbon atoms), 71.9, 71.6, 69.6, 66.3, 65.4, 55.4, 53.9, 46.6, 40.9, 39.8, 38.2, 37.0, 34.1, 26.2, 21.4, 20.1, 18.5, 18.5, 18.5, 18.4, 18.3, 15.0, 13.4, 13.0, 12.9, 12.6, 9.5, 7.6, 7.3, 7.3, 7.2, 7.0, 6.1, 5.7, 5.7, −1.3, −4.3, −4.4 ppm; **HRMS** (ESI) *m*/*z*: [M + Na]^+^ calcd for C_87_H_170_O_13_Si_7_Na 1642.0924; found: 1642.0933; **[α]**_**D**_^**24.2**^ = +30.8 (*c* = 0.13, CH_2_Cl_2_); **R**_*f*_ = 0.38 (petroleum ether:EtOAc 19:1).

#### Allyl (3*R*,6*R*,7*R*,8*E*,10*S*,11*S*,13*S*,14*S*,15*S*,16*R*,17*R*,18*E*,20*S*,21*Z*)-3-((*tert*-Butyldimethylsilyl)oxy)-15-hydroxy-4,4,6,8,10,14,20-heptamethyl-5-oxo-11,13,16,17-tetrakis((triethylsilyl)oxy)-7-((triisopropylsilyl)oxy)-16-(((2-(trimethylsilyl)ethoxy)methoxy)methyl)tricosa-8,18,21-trienoate (**96**)

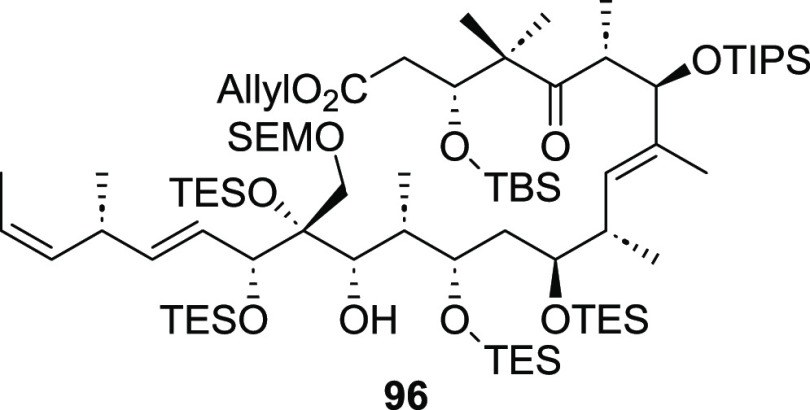



The glassware was neither dried nor purged with inert gas prior to usage. 2,3-Dichloro-5,6-dicyano-1,4-benzoquinone (41.0 mg, 181 μmol, 1.40 equiv) was added to a solution of TIPS-ether **60** (210 mg, 130 μmol, 1.00 equiv) in CH_2_Cl_2_ (5.2 mL) and an aqueous solution of phosphate buffer (pH = 7, 1.3 mL) at 0 °C. The reaction mixture was stirred for 20.5 h before the subsequent addition of a saturated aqueous solution of NaHCO_3_ and saturated aqueous solution of Na_2_S_2_O_3_. The phases were separated and the aqueous layer was extracted with CH_2_Cl_2_ (3×). The combined organic layers were washed with brine, dried over MgSO_4_, and concentrated *in vacuo*. The crude product was purified via column chromatography (petroleum ether:EtOAc 60:1) providing alcohol **96** (175 mg, 117 μmol, 90%) as a colorless oil. ^**1**^**H NMR (400 MHz, CDCl**_**3**_**)** δ = 5.94–5.84 (m, 1H), 5.78 (ddd, *J* = 15.4, 9.2, 1.3 Hz, 1H), 5.47 (dd, *J* = 15.4, 6.1 Hz, 1H), 5.44–5.37 (m, 2H), 5.30 (dq, *J* = 17.2, 1.4 Hz, 1H), 5.26–5.20 (m, 2H), 4.66 (d, *J* = 6.4 Hz, 1H), 4.62 (d, *J* = 6.4 Hz, 1H), 4.55 (dt, *J* = 5.8, 1.4 Hz, 2H), 4.51 (t, *J* = 5.2 Hz, 1H), 4.37 (d, *J* = 7.9 Hz, 1H), 4.26 (d, *J* = 9.1 Hz, 1H), 4.07 (d, *J* = 7.2 Hz, 1H), 3.70–3.58 (m, 4H), 3.55 (d, *J* = 10.0 Hz, 1H), 3.44 (d, *J* = 10.0 Hz, 1H), 3.24–3.12 (m, 2H), 2.48 (d, *J* = 7.2 Hz, 1H), 2.40–2.31 (m, 1H), 2.33 (d, *J* = 5.2 Hz, 2H), 1.82–1.75 (m, 1H), 1.67–1.55 (m, 5H), 1.62 (dd, *J* = 6.7, 1.8 Hz, 3H), 1.15 (s, 3H), 1.11 (d, *J* = 6.8 Hz, 3H), 1.07–1.05 (m, 24H), 0.99–0.87 (m, 41H), 0.88 (d, *J* = 7.0 Hz, 3H), 0.85 (s, 9H), 0.85–0.84 (m, 3H), 0.72–0.52 (m, 24H), 0.07 (s, 3H), 0.03 (s, 9H), 0.01 (s, 3H) ppm; ^**13**^**C{1H}-NMR (100 MHz, CDCl**_**3**_**)** δ = 215.1, 171.9, 137.6, 135.4, 134.5, 132.2, 131.4, 128.7, 122.9, 118.5, 96.3, 82.0, 80.5, 76.5, 75.3, 73.4, 72.1, 70.4, 69.6, 66.2, 65.4, 54.3, 46.5, 40.6, 39.9, 39.7, 37.8, 34.2, 26.2, 21.8, 20.4, 18.5, 18.5, 18.4, 18.3, 17.9, 15.7, 14.2, 13.0, 13.0, 12.2, 10.2, 7.7, 7.3, 7.3, 7.1, 7.1, 5.7, 5.7, 5.6, −1.3, −4.3 (2 different carbon atoms) ppm; **HRMS** (ESI) *m*/*z*: [M + Na]^+^ calcd for C_79_H_162_O_12_Si_7_Na 1522.0349; found 1522.0339; **[α]**_**D**_^**23.5**^ = +21.1 (*c* = 0.19, CH_2_Cl_2_); **R**_*f*_ = 0.39 (petroleum ether:EtOAc 19:1).

#### Allyl (3*R*,6*R*,7*R*,8*E*,10*S*,11*S*,13*S*,14*R*,16*S*,17*R*,18*E*,20*S*,21*Z*)-3-((*tert*-Butyldimethylsilyl)oxy)-4,4,6,8,10,14,20-heptamethyl-5,15-dioxo-11,13,16,17-tetrakis((triethylsilyl)oxy)-7-((triisopropylsilyl)oxy)-16-(((2-(trimethylsilyl)ethoxy)methoxy)methyl)tricosa-8,18,21-trienoate (**61**)

Dess–Martin periodinane^32^ (249 mg, 587 μmol, 5.02 equiv) was added to a suspension of alcohol **96** and NaHCO_3_ (103 mg, 1.23 mmol, 10.5 equiv) in CH_2_Cl_2_ (2.3 mL) at 0 °C. The reaction mixture was stirred for 6 h at 40 °C before it was cooled to 0 °C, and a saturated aqueous solution of NaHCO_3_ and saturated aqueous solution of Na_2_S_2_O_3_ were added successively. The phases were separated and the aqueous layer was extracted with CH_2_Cl_2_ (3×). The combined organic layers were washed with brine, dried over MgSO_4_, and concentrated *in vacuo*. The crude product was purified via column chromatography (petroleum ether:EtOAc 60:1) providing ketone **61** (162 mg, 108 μmol, 92%) as a colorless oil. ^**1**^**H NMR (400 MHz, CDCl**_**3**_**)** δ = 5.94–5.85 (m, 1H), 5.52 (dd, *J* = 15.6, 5.4 Hz, 1H), 5.48–5.38 (m, 2H), 5.33–5.28 (m, 2H), 5.24–5.16 (m, 2H), 4.56–4.48 (m, 5H), 4.36 (d, *J* = 7.5 Hz, 1H), 4.30 (d, *J* = 8.6 Hz, 1H), 4.02 (q, *J* = 5.7 Hz, 1H), 3.66–3.62 (m, 1H), 3.59–3.48 (m, 3H), 3.29 (d, *J* = 10.1 Hz, 1H), 3.25–3.13 (m, 2H), 3.00–2.93 (m, 1H), 2.35–2.28 (m, 1H), 2.34 (d, *J* = 5.5 Hz, 2H), 1.63 (dd, *J* = 6.8, 1.8 Hz, 3H), 1.60 (d, *J* = 0.8 Hz, 3H), 1.49 (t, *J* = 5.8 Hz, 2H), 1.16 (s, 3H), 1.13 (d, *J* = 7.3 Hz, 3H), 1.10 (d, *J* = 7.0 Hz, 3H), 1.07–1.05 (m, 24H), 1.03–0.93 (m, 30H), 0.91–0.84 (m, 5H) 0.89 (t, *J* = 8.0 Hz, 9H), 0.85 (s, 9H), 0.77–0.60 (m, 18H), 0.51 (t, *J* = 7.9 Hz, 6H), 0.08 (s, 3H), 0.01 (s, 12H) ppm; ^**13**^**C{1H}-NMR (100 MHz, CDCl**_**3**_**)** δ = 215.6, 215.2, 171.9, 138.2, 135.5, 133.9, 132.2, 131.3, 127.6, 123.2, 118.5, 95.9, 88.8, 80.6, 77.2, 73.4, 73.3, 72.2, 71.0, 65.8, 65.4, 54.3, 48.1, 46.5, 42.8, 39.9, 38.9, 34.0, 26.2, 21.9, 20.3, 18.5, 18.5, 18.4, 18.2, 17.9, 15.7, 14.7, 13.0, 13.0, 12.1, 11.8, 7.5, 7.5, 7.3, 7.0, 6.9, 6.2, 5.7, 5.3, −1.3, −4.3, −4.4 ppm; **HRMS** (ESI) *m*/*z*: [M + Na]^+^ calcd for C_79_H_160_O_12_Si_7_Na 1520.0193; found 1520.0195; **[α]**_**D**_^**24.9**^ = +19.4 (*c* = 0.17, CH_2_Cl_2_); **R**_*f*_ = 0.34 (petroleum ether:EtOAc 19:1).

#### Allyl (3*R*,6*R*,7*R*,8*E*,10*S*,11*S*,13*S*,14*R*,16*S*,17*R*,18*E*,20*S*,21*Z*)-3-((*tert*-Butyldimethylsilyl)oxy)-11,13-dihydroxy-16-(hydroxymethyl)-4,4,6,8,10,14,20-heptamethyl-5,15-dioxo-16,17-bis((triethylsilyl)oxy)-7-((triisopropylsilyl)oxy)tricosa-8,18,21-trienoate (**62**)

Nitromethane (0.16 mL, 3.03 mmol, 28.0 equiv) was added to a suspension of magnesium bromide ethyl etherate (391 mg, 1.51 mmol, 14.2 equiv) in Et_2_O (0.70 mL) at room temperature. The mixture was stirred until full dissolution of the solids (10 min) and added to a solution of ketone **61** in Et_2_O (1.5 mL) afterward. The reaction mixture was stirred for 23.5 h before EtOAc and water were added. The phases were separated and the aqueous layer was extracted with EtOAc (3×). The combined organic layers were washed with brine, dried over MgSO_4_, and concentrated *in vacuo*. The crude product was purified via column chromatography (petroleum ether:EtOAc 20:1 → 5:1 → 3:1) providing triol **62** (47.0 mg, 41.2 μmol, 38%) and a mixture of partially deprotected starting material (16.0 mg) as a colorless oil. The obtained mixture was subjected to the same reaction conditions providing triol **62** (4.0 mg, 3.51 μmol, 3%, 41% in total) as a colorless oil. Triol **62** was used in the next reaction without detailed characterization.

#### Allyl (3*R*,6*R*,7*R*,10*S*,*E*)-3-((*tert*-Butyldimethylsilyl)oxy)-10-((4*S*,6*S*)-6-((2*R*,4*S*,5*R*,6*E*,8*S*,9*Z*)-4-(hydroxymethyl)-8-methyl-3-oxo-4,5-bis((triethylsilyl)oxy)undeca-6,9-dien-2-yl)-2,2-dimethyl-1,3-dioxan-4-yl)-4,4,6,8-tetramethyl-5-oxo-7-((triisopropylsilyl)oxy)undec-8-enoate (**64**)

The glassware was not dried prior to usage. Glassware for ketal cleavage was not purged with inert gas. Pyridinium *p*-toluenesulfonate (2.2 mg, 8.75 μmol, 0.20 equiv) was added to a solution of triol **62** and 2,2-dimethoxypropane (0.80 mL, 6.53 mmol, 149 equiv) in acetone (0.80 mL) at 0 °C. The reaction mixture was stirred for 2.25 h before a saturated aqueous solution of NaHCO_3_ was added. The phases were separated and the aqueous layer was extracted with MTBE (3×). The combined organic layers were washed with brine, dried over MgSO_4_, and concentrated *in vacuo*. The crude product was purified via column chromatography (petroleum ether:MTBE 9:1) providing acetonide **64** (30.0 mg, 25.4 μmol, 58%) and ketal **63** (7.5 mg, 5.99 μmol, 14%) as colorless oils. Pyridinium *p*-toluenesulfonate (0.7 mg, 2.79 μmol, 0.47 equiv) was added to a solution of ketal **63** (7.5 mg, 5.99 μmol, 1.00 equiv) in CH_2_Cl_2_ (0.48 mL) and MeOH (40 μL) at 0 °C. The reaction mixture was stirred for 1.5 h at 0 °C and for 6 h at room temperature before a saturated aqueous solution of NaHCO_3_ was added. The phases were separated and the aqueous layer was extracted with CH_2_Cl_2_ (3×). The combined organic layers were washed with brine, dried over MgSO_4_ and concentrated *in vacuo*. The crude product was purified via column chromatography (petroleum ether:EtOAc 10:1 → 5:1) providing acetonide **64** (1.0 mg, 0.85 μmol, 14%, 60% (63% brsm) over 2 steps) and triol **62** (3.0 mg, 2.63 μmol, 6% over 2 steps) as colorless oils. ^**1**^**H NMR (400 MHz, CDCl**_**3**_**)** δ = 5.96–5.87 (m, 1H), 5.56 (dd, *J* = 15.5, 5.8 Hz, 1H), 5.46–5.40 (m, 2H), 5.34–5.29 (m, 1H), 5.25–5.18 (m, 2H), 5.07 (d, *J* = 8.6 Hz, 1H), 4.58 (d, *J* = 5.8 Hz, 2H), 4.48 (dd, *J* = 5.4, 4.8 Hz, 1H), 4.32 (d, *J* = 8.6 Hz, 1H), 4.28 (d, *J* = 8.4 Hz, 1H), 3.99 (q, *J* = 7.2 Hz, 1H), 3.83 (d, *J* = 10.7 Hz, 1H), 3.50–3.42 (m, 2H), 3.36 (d, *J* = 10.7 Hz, 1H), 3.25–3.15 (m, 3H), 2.34–2.33 (m, 2H), 2.25–2.16 (m, 1H), 1.78–1.72 (m, 1H), 1.63 (dd, *J* = 6.7, 1.7 Hz, 3H), 1.58 (s, 3H), 1.46–1.38 (m, 1H), 1.31 (s, 6H), 1.14 (s, 3H), 1.11–1.01 (m, 39H), 0.93 (d, *J* = 6.6 Hz, 3H), 0.90–0.86 (m, 12H), 0.85 (s, 9H), 0.77 (q, *J* = 8.0 Hz, 6H), 0.52–0.46 (m, 6H), 0.06 (s, 3H), 0.01 (s, 3H) ppm; ^**13**^**C{1H}-NMR (100 MHz, CDCl**_**3**_**)** δ = 218.5, 217.4, 171.7, 138.3, 136.1, 134.0, 132.2, 130.2, 127.8, 123.1, 118.7, 100.1, 90.1, 80.9, 77.2, 72.5, 70.7, 69.7, 67.0, 65.5, 54.5, 46.8, 45.5, 39.9, 37.8, 34.1, 33.8, 26.2, 25.8, 24.9, 21.4, 20.4, 18.8, 18.5, 18.4, 18.4, 16.3, 16.1, 13.0, 12.9, 12.9, 12.0, 7.4, 7.1, 6.9, 5.2, −4.3, −4.4 ppm; **HRMS** (ESI) *m*/*z*: [M + Na]^+^ calcd for C_64_H_122_O_11_Si_4_Na 1201.7962; found 1201.7917; **[α]**_**D**_^**25.4**^ = +11.54 (*c* = 0.26, CH_2_Cl_2_); **R**_*f*_ = 0.34 (petroleum ether:EtOAc 19:1); 0.72 (petroleum ether:MTBE 9:1).

#### (1*S*,2*R*,4*S*,9*R*,12*R*,13*R*,16*S*,17*S*,*E*)-9-((*tert*-Butyldimethylsilyl)oxy)-2,10,10,12,14,16,19,19-octamethyl-4-((1*R*,2*E*,4*S*,5*Z*)-4-methyl-1-((triethylsilyl)oxy)hepta-2,5-dien-1-yl)-4-((triethylsilyl)oxy)-13-((triisopropylsilyl)oxy)-6,18,20-trioxabicyclo[15.3.1]henicos-14-ene-3,7,11-trione (**97**)

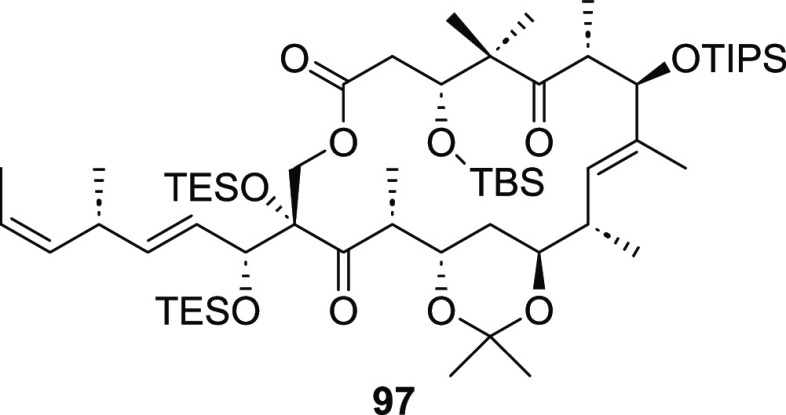



Glassware for the macrolactonization was not dried prior usage. Tri-*n*-butyltin hydride (19.1 μL, 72.1 μmol, 5.67 equiv) was added to a solution of acetonide **41** (15.0 mg, 12.7 μmol, 1.00 equiv) and tetrakis(triphenylphosphine)palladium(0) (2.3 mg, 1.99 μmol, 0.16 equiv) in CH_2_Cl_2_ (1.3 mL) at room temperature. The reaction mixture was stirred for 35 min before a saturated aqueous solution of NH_4_Cl was added. The phases were separated and the aqueous layer was extracted with CH_2_Cl_2_ (4×). The combined organic layers were washed with brine, dried over MgSO_4_, and concentrated *in vacuo* providing a yellow oil that was used in the next reaction without detailed characterization. The obtained yellow oil was dissolved in CH_2_Cl_2_ (2.5 mL) and *N*,*N*-diisopropylethylamine (0.07 mL, 412 μmol, 32.4 equiv), 4-dimethylaminopyridine (22.0 mg, 180 μmol, 14.2 equiv), and 2,4,6-trichlorbenzoyl chloride (1.66 M in CH_2_Cl_2_, 100 μL, 166 μmol, 13.1 equiv) were added successively. The reaction mixture was stirred for 2.75 h before a saturated aqueous solution of NaHCO_3_ was added and stirring was continued for 15 min. The phases were separated, and the aqueous layer was extracted with CH_2_Cl_2_ (4×). The combined organic layers were washed with an aqueous solution of KHSO_4_ (1 M), a saturated aqueous solution of NaHCO_3_ and brine, dried over MgSO_4_, and concentrated *in vacuo*. The crude product was purified via column chromatography (petroleum ether:EtOAc 60:1) providing macrolactone **97** (impure, 6.7 mg, < 5.97 μmol, < 47% over 2 steps) as a colorless oil. Macrolactone **97** was used in the next reaction without detailed characterization.

#### (4*R*,7*R*,8*R*,11*S*,12*S*,14*S*,15*R*,17*S*,*E*)-4-((*tert*-Butyldimethylsilyl)oxy)-12,14-dihydroxy-5,5,7,9,11,15-hexamethyl-17-((1*R*,2*E*,4*S*,5*Z*)-4-methyl-1-((triethylsilyl)oxy)hepta-2,5-dien-1-yl)-17-((triethylsilyl)oxy)-8-((triisopropylsilyl)oxy)oxacyclooctadec-9-ene-2,6,16-trione (**65**)

The glassware was neither dried nor purged with inert gas prior to usage. Pyridinium *p*-toluenesulfonate (2.3 mg, 9.15 μmol, 1.53 equiv) was added to a solution of macrolactone **97** (6.7 mg, 5.97 μmol, 1.00 equiv) in CH_2_Cl_2_ (0.75 mL) and MeOH (0.15 mL) at room temperature. The reaction mixture was stirred for 20 h before a saturated aqueous solution of NaHCO_3_ was added. The phases were separated and the aqueous layer was extracted with CH_2_Cl_2_ (3×). The combined organic layers were washed with brine, dried over MgSO_4_, and concentrated *in vacuo*. The crude product was purified via column chromatography (petroleum ether:EtOAc 20:1 → 10:1) providing diol **65** (4.5 mg, 4.16 μmol, 70%) as a colorless oil. ^**1**^**H NMR (600 MHz, CDCl**_**3**_**)** δ = 5.58 (dd, *J* = 15.7, 5.7 Hz, 1H), 5.49–5.44 (m, 2H), 5.21–5.16 (m, 2H), 4.45 (d, *J* = 8.7 Hz, 1H), 4.29 (d, *J* = 9.1 Hz, 1H), 4.23 (dd, *J* = 7.2, 2.0 Hz, 1H), 4.12 (t, *J* = 6.6 Hz, 1H), 4.06 (d, *J* = 11.4 Hz, 1H), 3.80 (d, *J* = 11.4 Hz, 1H), 3.66–3.63 (m, 1H), 3.33 (q, *J* = 7.2 Hz, 1H), 3.26–3.20 (m, 1H), 3.18–3.13 (m, 1H), 3.12 (bs, 1H), 2.65 (dd, *J* = 16.8, 2.0 Hz, 1H), 2.52–2.47 (m, 1H), 2.10 (dd, *J* = 16.8, 7.2 Hz, 1H), 1.99 (bs, 1H), 1.64–1.59 (m, 1H), 1.63 (dd, *J* = 6.8, 1.5 Hz, 3H), 1.61 (s, 3H), 1.43–1.38 (m, 1H), 1.20 (d, *J* = 6.8 Hz, 3H), 1.15 (d, *J* = 7.2 Hz, 3H), 1.14 (s, 3H), 1.10 (s, 3H), 1.08 (d, *J* = 7.0 Hz, 3H), 1.06–1.04 (m, 21H), 1.02 (t, *J* = 8.0 Hz, 9H), 0.95 (d, *J* = 6.9 Hz, 3H), 0.89 (t, *J* = 8.0 Hz, 9H), 0.84 (s, 9H), 0.74 (q, *J* = 8.0 Hz, 6H), 0.52 (q, *J* = 8.0 Hz, 6H), 0.07 (s, 3H), – 0.04 (s, 3H) ppm; ^**13**^**C{1H}-NMR (150 MHz, CDCl**_**3**_**)** δ = 221.0, 217.0, 171.7, 139.2, 136.6, 133.5, 130.9, 126.5, 123.7, 87.6, 81.8, 76.6, 73.9, 73.1, 69.1, 69.0, 52.9, 47.4, 44.8, 40.2, 38.5, 36.4, 34.0, 26.3, 24.0, 20.3, 19.4, 18.6, 18.5, 18.4, 17.7, 15.1, 13.0, 12.9, 11.9, 8.7, 7.4, 7.0, 6.9, 5.2, – 4.0, – 4.5 ppm; **HRMS** (ESI) *m*/*z*: [M + Na]^+^ calcd for C_58_H_112_O_10_Si_4_Na 1103.7230; found 1103.7228; **[α]**_**D**_^**24.6**^ = +42.3 (*c* = 0.44, CH_2_Cl_2_); **R**_*f*_ = 0.37 (petroleum ether:EtOAc 9:1).

#### (4*R*,7*R*,8*R*,11*S*,12*S*,14*S*,15*R*,17*S*,*E*)-4,8,12,14,17-Pentahydroxy-17-((1*R*,2*E*,4*S*,5*Z*)-1-hydroxy-4-methylhepta-2,5-dien-1-yl)-5,5,7,9,11,15-hexamethyloxacyclooctadec-9-ene-2,6,16-trione (**66**)

The glassware was not dried prior to usage. Triethylamine trihydrofluoride (0.28 mL, 0.12 mL/μmol **65**) was added to a solution of diol **65** (2.5 mg, 2.31 μmol, 1.00 equiv) and trimethylamine (0.24 mL) in MeCN (0.33 mL). The reaction mixture stood for 6 d before it was cooled to 0 °C. While stirring a saturated aqueous solution of NaHCO_3_ and EtOAc were added successively. The phases were separated and the aqueous layer was extracted with EtOAc (3 x). The combined organic layers were washed with brine, dried over MgSO_4_ and concentrated *in vacuo*. The crude product was purified via column chromatography (petroleum ether:EtOAc 1:1 → 1:2 → EtOAc) providing desepoxy-tedanolide C (**66**) (impure, 0.4 mg, < 0.68 μmol, < 30%) as a colorless wax. The NMR data of desepoxy-tedanolide C (**66**) are incomplete, since the compound decomposed during the measurement. Due to the same reason, no optical rotation value could be measured. ^**1**^**H NMR (600 MHz, CD**_**3**_**OD)** δ = 5.68 (dd, *J* = 15.8, 6.2 Hz, 1H), 5.57 (ddd, *J* = 15.8, 8.4, 1.3 Hz, 1H), 5.47–5.41 (m, 1H), 5.26–5.21 (m, 1H), 5.19 (d, *J* = 9.4 Hz, 1H), 4.27 (d, *J* = 8.4 Hz, 1H), 4.15 (dd, *J* = 10.8, 3.1 Hz, 1H), 4.08–4.04 (m, 1H), 4.06 (d, *J* = 9.6 Hz, 1H), 3.96 (d, *J* = 11.1 Hz, 1H), 3.88 (d, *J* = 11.1 Hz, 1H), 3.56–3.52 (m, 1H), 3.31 (COSY & HSQC, overlapped by CD_3_OD 1H), 3.27–3.21 (m, 2H), 2.23 (COSY & HSQC, 1H), 2.21 (COSY & HSQC, 1H), 2.16 (COSY & HSQC, 1H), 1.63 (dd, *J* = 6.8, 1.8 Hz, 3H), 1.56 (d, *J* = 1.2 Hz, 3H), 1.41 (COSY & HSQC, 1H), 1.24 (s, 3H), 1.23 (d, *J* = 6.4 Hz, 3H), 1.22 (d, *J* = 6.8 Hz, 3H), 1.17 (s, 3H), 1.17 (COSY & HSQC, 1H), 1.07 (d, *J* = 6.9 Hz, 3H), 0.99 (d, *J* = 6.8 Hz, 3H); ^**13**^**C{1H}-NMR (150 MHz, CD**_**3**_**OD)** δ = 220.2 (HMBC), 140.3 (HSQC), 135.5 (HMBC), 135.0 (HSQC), 134.1 (HSQC), 126.7 (HSQC), 124.3 (HSQC), 81.1 (HSQC), 75.7 (HSQC), 73.5 (HSQC), 73.0 (HSQC), 70.9 (HSQC), 70.2 (HSQC), 53.0 (HMBC), 49.0 (HSQC, overlapped by CD_3_OD), 46.8 (HSQC), 41.4 (HSQC), 40.2 (HSQC), 39.2 (HSQC), 35.3 (HSQC), 25.0 (HSQC), 21.2 (HSQC), 18.5 (HSQC), 17.5 (HSQC), 17.4 (HSQC), 13.2 (HSQC), 12.6 (HSQC), 11.1 (HSQC); **HRMS** (ESI) *m*/*z*: [M + Na]^+^ calcd for C_31_H_50_O_10_Na 605.3302; found 605.3308; **R**_*f*_ = 0.44 (EtOAc).

## Data Availability

The data underlying this study are available in the published article and its Supporting Information.

## Supporting Information Available

Structure elucidation of alcohols **23a**/**b**, **48**, **58**, **78**, **85**, **92**, and **95**, detailed comparison of NMR data of **8** and **66**, and ^1^H and ^13^C NMR spectra of all characterized compounds (PDF)

## References

[ref1] aTaylor R. E. Tedanolide and the evolution of polyketide inhibitors in eukaryotic protein synthesis *Nat*. Prod. Rep. 2008, 25, 854 10.1039/b805700c. 18820754

[ref2] Schmitz F. J.; Gunasekera S. P.; Yalamanchili G.; Hossain M. B.; van der Helm D. Tedanolide: A Potent Cytotoxic Macrolide from Caribbean Sponge. Tedania ignis J. Am. Chem. Soc. 1984, 106, 7251 10.1021/ja00335a069.

[ref3] Fusetani N.; Sugawara T.; Matsunaga S.; Hirota H. Bioactive marine metabolites. Part 35. Cytotoxic metabolites of the marine sponge Mycale adhaerens Lambe. J. Org. Chem. 1991, 56, 4971 10.1021/jo00016a031.

[ref4] Chevallier C.; Bugni T. S.; Feng X.; Harper M. K.; Orendt A. M.; Ireland C. M. Tedanolide C: A Potent New 18-Membered Ring Cytotoxic Macrolide Isolated from Papua New Guinea Marine Sponge *Ircinia sp*. J. Org. Chem. 2006, 71, 2510 10.1021/jo052285+. 16526806 PMC2533847

[ref5] aNishimura S.; Matsunaga S.; Yoshida M.; Hirota H.; Yokoyama S.; Fusetani N. 13-Deoxytedanolide, a marine sponge-derived antitumor macrolide, binds to the 60S large ribosomal subunit. Bioorg. Med. Chem. 2005, 13, 449 10.1016/j.bmc.2004.10.012. 15598566

[ref6] aMeragelman T. L.; Willis R. H.; Woldemichael G. M.; Heaton A.; Murphy P. T.; Snader K. M.; Newman D. J.; van Soest R.; Boyd M. R.; Cardellina J. H.; McKee T. Candidaspongiolides, Distinctive Analogues of Tedanolide from Sponges of Genus *Candidaspongia*. J. Nat. Prod. 2007, 70, 1133 10.1021/np0700974. 17564468 PMC2288652

[ref7] aEhrlich G.; Hassfeld J.; Eggert U.; Kalesse M. The Total Synthesis of (+)-Tedanolide. J. Am. Chem. Soc. 2006, 128, 14038 10.1021/ja0659572. 17061881

[ref8] aSmith A. B. III; Adams C. M.; Lodise Barbosa S. A.; Degnan A. P. Total Synthesis of (+)-13-Deoxytedanolide. J. Am. Chem. Soc. 2003, 125, 350 10.1021/ja0289649. 12517144

[ref9] aBarth R.; Roush W. R. Enantioselective Synthesis of α-Methylene-β-hydroxy Carboxylic Acid Derivatives via a Diastereoselective Aldol/β-Elimination Sequence: Application to the C(15)-C(21) Fragment of Tedanolide C. Org. Lett. 2010, 12, 2342 10.1021/ol1006955. 20405855 PMC2872132

[ref10] Smith T. E.; Fink S. J.; Levine Z. G.; McClelland K. A.; Zackheim A. A.; Daub M. E. Stereochemically Versatile Synthesis of the C1-C12 Fragment of Tedanolide C. Org. Lett. 2012, 14, 1452 10.1021/ol300194x. 22375885 PMC3312041

[ref11] Zambrana J.; Romea P.; Urpí F. Studies towards the synthesis of tedanolide C. Construction of the C13-*epi* C1-C15 fragment. Org. Biomol. Chem. 2016, 14, 5219 10.1039/C6OB00896H. 27215808

[ref12] aLücke D.; Kalesse M. Synthesis of Desepoxy-Tedanolide C. Chem.—Eur. J. 2021, 27, 7085. 33769622 10.1002/chem.202100553 PMC8251979

[ref13] aAlhamadsheh M. M.; Hudson R. A.; Viranga Tillekeratne L. M. Design, Total Synthesis, and Evaluation of Novel Open-Chain Epothilon Analogues. Org. Lett. 2006, 8, 685 10.1021/ol0528787. 16468742

[ref14] aTrost B. M.; O’Boyle B. M.; Hund D. Total Synthesis and Stereochemical assignment of (−)-Ushikulide A. J. Am. Chem. Soc. 2009, 131, 41. 10.1021/ja906056v PMC2791109 19775093

[ref15] Koch G.; Loiseleur O.; Fuentes D.; Jantsch A.; Altmann K.-H. Diastereoselective Titanium Enolate Aldol Reaction for the Total Synthesis of Epothilones. Org. Lett. 2002, 4, 3811 10.1021/ol026480b. 12599465

[ref16] Bülow L.; Naini A.; Fohrer J.; Kalesse M. A Kiyooka Aldol Approach for the synthesis of the C(14)-C(23) Segment of the Diastereomeric Analog of Tedanolide C. Org. Lett. 2011, 13, 6038 10.1021/ol202515x. 22026452

[ref17] Kiyooka S.; Kaneko Y.; Komura M.; Matsuo H.; Nakano M. Enantioselective chiral borane-mediated aldol reactions of silyl ketene acetals with aldehydes. The novel effect of the trialkylsilyl group of the silyl ketene acetal on the reaction course. J. Org. Chem. 1991, 56, 2276 10.1021/jo00007a003.

[ref18] Lücke D.; Kalesse M. Chiral Polyoxygenated Tertiary Alcohols through a Kiyooka aldol reaction. Synlett 2022, 33, 1117 10.1055/a-1775-7590.

[ref19] Martin N.; Thomas E. J. Total Synthesis of epothilones using functionalized allylstannanes for remote stereocontrol. Org. Biomol. Chem. 2012, 10, 7952 10.1039/c2ob26310f. 22940725

[ref20] aGriffith W. P.; Ley S. V.; Whitcombe G. P.; White A. D. Preparation and use of tetra-n-butylammonium per-ruthenate (TBAP reagent) and tetra-n-propylammonium per-ruthenate (TPAP reagent) as new catalytic oxidants for alcohols. J. Chem. Soc., Chem. Commun. 1987, 1625 10.1039/c39870001625.

[ref21] Nagasawa T.; Kuwahara S. Formal Total Synthesis of Lactimidomycin. Org. Lett. 2013, 15, 3002 10.1021/ol401214f. 23731346

[ref22] Omura K.; Sharma A. K.; Swern D. Dimethyl sulfoxide-trifluoroacetic anhydride. New reagent for oxidation of alcohols to carbonyls. J. Org. Chem. 1976, 41, 957 10.1021/jo00868a012.

[ref23] aNicolaou K. C.; Patron A. P.; Ajito K.; Richter P. K.; Khatuya H.; Bertinato P.; Miller R. A.; Tomaszewski M. J. Total Synthesis of Swinholide A, Preswinholide A and Hemiswinholide A. Chem.—Eur. J. 1996, 2, 847.

[ref24] Cram D. J.; Kopecky K. R. Studies in Stereochemistry. XXX. Models for Steric Control of Asymmetric Induction. J. Am. Chem. Soc. 1959, 81, 2748 10.1021/ja01520a036.

[ref25] aJulia M.; Paris J.-M. Syntheses a l’aide de sulfones v^(+)^-methode de synthese generale de double liaisons. Tetrahedron Lett. 1973, 14, 4833 10.1016/S0040-4039(01)87348-2.

[ref26] aMulzer J.; Dupre S.; Buschmann J.; Luger P. Linear Total Synthesis of (−)-ACRL Toxin III B. Angew. Chem., Int. Ed. 1993, 32, 1452 10.1002/anie.199314521.

[ref27] aSaksena A. K.; Mangiaracina P. Recent studies on veratrum alkaloids: a new reaction of sodium triacetoxyborohydride [NaBH(OAc)_3_]. Tetrahedron Lett. 1983, 24, 273 10.1016/S0040-4039(00)81383-0.

[ref28] Inanaga J.; Hirata K.; Saeki H.; Katsuki T.; Yamaguchi M. A Rapid Esterefication by Means of Mixed Anhydride and Its Application to Large-ring Lactonization. Bull. Chem. Soc. Jpn. 1979, 52, 1989 10.1246/bcsj.52.1989.

[ref29] Vakalopoulos A.; Hoffmann H. M. R. Novel Deprotection of SEM Ethers: A Very Mild and Selective Method Using Magnesium Bromide. Org. Lett. 2000, 2, 1447 10.1021/ol0057784. 10814469

[ref30] Dess D. B.; Martin J. C. Readily Accessible 12-I-5-Oxidant for the Conversion of Primary and Secondary Alcohols to Aldehydes and Ketones. J. Org. Chem. 1983, 48, 4155 10.1021/jo00170a070.

[ref31] Larsen B. J.; Sun Z.; Nagorny P. Synthesis of Eukaryotic Translation Elongation Inhibitor Lactimidomycin via Zn(II)-Mediated Horner–Wadsworth–Emmons Macrocyclization. Org. Lett. 2013, 15, 2998 10.1021/ol401186f. 23731327

[ref32] Ireland R. E.; Liu L. An Improved Procedure for the Preparation of the Dess–Martin Periodinane. J. Org. Chem. 1993, 58, 2899 10.1021/jo00062a040.

