# Supplementary material for: Development of the Synthesis of Desepoxy-Tedanolide C
Source: J Org Chem. 2024 Jan 25;89(4):2408–30. doi: 10.1021/acs.joc.3c02437 (PMC10877616; doi:10.1021/acs.joc.3c02437)

Supporting Information for

Development of the Synthesis of Desepoxy-Tedanolide C

Daniel Lücke, and Markus Kalesse*

Institute of Organic Chemistry, Gottfried Wilhelm Leibniz Universität Hannover, Schneiderberg 1B, 30167 Hannover, Germany; Centre of Biomolecular Drug Research (BMWZ), Gottfried Wilhelm Leibniz Universität Hannover, Schneiderberg 38, 30167 Hannover, Germany

Corresponding Author

*email: markus.kalesse@oci.uni-hannover.de

Table of Contents

1. Structure elucidation of alcohols **23a/b**, **48**, **58**, **78**, **85**, **92** and **95** S4

2. Comparison of NMR data of isolated tedanolide C (**8**) and desepoxy-tedanolide **66**.......... S19

3. References S20

4. NMR data S21

Northern fragment **11** S21

TBS-ether **67** S23

Alcohol **21** S25

Pivalate **68** S27

(*S*)-Mosher ester **S1** S29

(*R*)-Mosher ester **S2** S30

(*S*)-Mosher ester **S3** S31

(*R*)-Mosher ester **S4** S32

TBS-ether **69a** S33

TBS-ether **69b** S35

Aldehyde **28** S37

Ketone **25** S39

Ketone **30** S41

Ketone **31** S43

TBS-ether **73** S45

Alcohol **74** S47

Pivalate **36** S49

Alcohol **75** S51

Eastern fragment **15** S53
Alcohol **76** S55
Pivalate **77** S57
Alcohol **78** S59
Acetonide **S5** S61
TES-ether **79** S63
Sulfide **81** S65
Sulfone **44** S67
Alkene **82** S69
Triol **45** S71
TES-ether **83** S73
Alcohol **84** S75
Alcohol **48** S77
(*S*)-Mosher ester **S6** S79
(*R*)-Mosher ester **S7** S80
Diol **85** S81
Acetonide **49** S83
Alcohol **86** S85
TIPS-ether **51** S87
PMP-acetal **53** S91
Pivalate **55a** S93
TES-ether **88a** S95
Diol **89a** S97
PMP-acetal **90** S99
Alcohol **91** S101
PMP-acetal **S8** S103
Diol **92** S105
Acetonide **S9** S107
TES-ether **93** S109
Alcohol **94** S111
(*S*)-Mosher ester **S10** S113
(*R*)-Mosher ester **S11** S114
TIPS-ether **60** S115
Alcohol **96** S117
Ketone **61** S119
Acetonide **64** S121

Diol **65** S123
Desepoxy-tedanolide C (**66**) S125

1. Structure elucidation of alcohols **23a/b**, **48**, **58**, **78**, **85**, **92** and **95**

Stereochemistry at C7 (tedanolide C numbering) of alcohols **23a** and **23b** were determined by the Mosher ester analysis,[1] which revealed the (*R*) configuration for **23a** and the (*S*) configuration for **23b**. For the methyl group at C6 a *syn* relation was assumed, since titanium tetrachloride derived enolates usually provide *syn* aldol products.[2]

(*S*)-Mosher ester **S1**

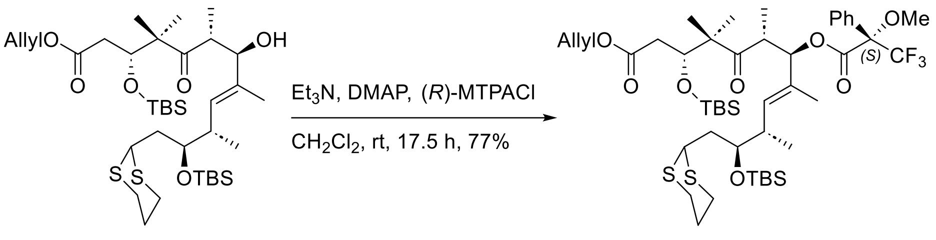

23a **S1**

(*R*)-(−)-α-Methoxy-α-(trifluoromethyl)phenylacetyl chloride (10.0 µL, 55.4 µmol, 2.65 equiv) was added to a solution of alcohol **23a** (15.0 mg, 20.9 µmol, 1.00 equiv), triethylamine (15.0 µL, 112 µmol, 5.36 equiv) and 4-dimethylaminopyridine (3.6 mg, 29.3 µmol, 1.40 equiv) in CH_2Cl_2 (0.50 mL) at room temperature. The reaction mixture was stirred for 17.5 h before it was diluted with EtOAc (5.0 mL). The organic phase was successively washed with an aqueous solution of $KHSO_4$ (1 M, 3 x), an aqueous solution of NaOH (1 M), a saturated aqueous solution of $NaHCO_3$ (3 x) and brine, dried over $MgSO_4$ and concentrated *in vacuo*. The crude product was purified *via* column chromatography (petroleum ether:EtOAc 20:1) providing (*S*)-mosher ester **S1** (15.0 mg, 16.1 µmol, 77%) as a colorless oil.

^{1}H-NMR (400 MHz, C_6D_6) δ = 7.77 – 7.75 (m, 2H), 7.18 – 7.14 (m, 2H), 7.10 – 7.06 (m, 1H), 5.92 (d, *J* = 7.9 Hz, 1H), 5.82 – 5.72 (m, 1H), 5.71 (d, *J* = 9.6 Hz, 1H), 5.17 – 5.12 (m, 1H), 5.01 – 4.98 (m, 1H), 4.87 (dd, *J* = 7.2, 3.4 Hz, 1H), 4.50 – 4.48 (m, 2H), 4.20 (dd, *J* = 8.2, 6.1 Hz, 1H), 3.93 – 3.89 (m, 1H), 3.52 (s, 3H), 3.30 (p, *J* = 7.2 Hz, 1H), 2.58 – 2.38 (m, 7H), 2.07 – 1.95 (m, 2H), 1.72 – 1.59 (m, 1H), 1.54 – 1.47 (m, 1H), 1.44 (s, 3H), 1.17 (d, *J* = 6.8 Hz, 3H), 1.08 (s, 3H), 1.04 (s, 9H), 1.01 (s, 3H), 0.96 (d, *J* = 5.7 Hz, 3H), 0.95 (s, 9H), 0.25 (s, 3H), 0.24 (s, 3H), 0.22 (s, 3H), 0.18 (s, 3H) ppm.

(*R*)-Mosher ester **S2**

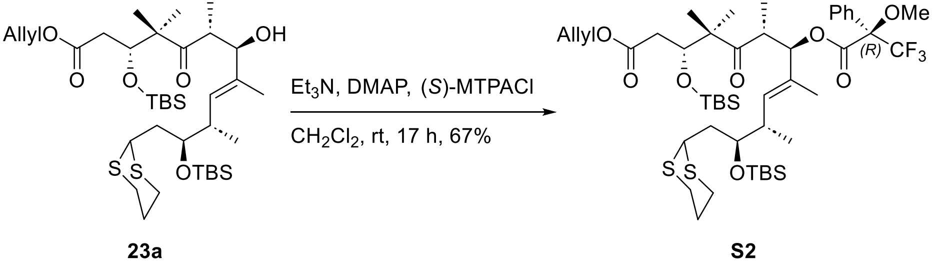

23a **S2**

(*S*)-(+)-α-Methoxy-α-(trifluoromethyl)phenylacetyl chloride (10.0 µL, 55.4 µmol, 2.65 equiv) was added to a solution of alcohol **23a** (15.0 mg, 20.9 µmol, 1.00 equiv), triethylamine (15.0 µL, 112 µmol, 5.36 equiv) and 4-dimethylaminopyridine (3.6 mg, 29.3 µmol, 1.40 equiv) in CH_2Cl_2 (0.50 mL) at room temperature. The reaction mixture was stirred for 17 h before it was diluted with EtOAc (5.0 mL). The

organic phase was successively washed with an aqueous solution of $KHSO_4$ (1 M, 3 x), an aqueous solution of NaOH (1 M), a saturated aqueous solution of $NaHCO_3$ (3 x) and brine, dried over $MgSO_4$ and concentrated *in vacuo*. The crude product was purified *via* column chromatography (petroleum ether:EtOAc 20:1) providing (*S*)-mosher ester **S2** (13.0 mg, 13.9 µmol, 67%) as a colorless oil.

^{1}H-NMR (400 MHz, C_6D_6) δ = 7.73 – 7.68 (m, 2H), 7.16 – 7.12 (m, 2H), 7.08 – 7.04 (m, 1H), 6.04 (d, *J* = 8.5 Hz, 1H), 5.85 – 5.72 (m, 2H), 5.17 – 5.12 (m, 1H), 5.02 – 4.98 (m, 1H), 4.85 (dd, *J* = 7.2, 3.1 Hz, 1H), 4.50 – 4.48 (m, 2H), 4.21 (dd, *J* = 7.9, 6.5 Hz, 1H), 3.95 – 3.91 (m, 1H), 3.54 (s, 3H), 3.33 – 3.25 (m, 1H), 2.60 – 2.35 (m, 7H), 2.08 – 1.96 (m, 2H), 1.71 – 1.64 (m, 1H), 1.64 (s, 3H), 1.55 – 1.49 (m, 1H), 1.06 (d, *J* = 6.7 Hz, 3H), 1.05 (s, 3H), 1.03 (s, 9H), 0.98 (s, 3H), 0.95 (s, 9H), 0.95 – 0.93 (m, 3H), 0.25 (s, 3H), 0.23 (s, 3H), 0.22 (s, 3H), 0.17 (s, 3H) ppm.

Table S1. Chemical shift values of alcohols **23a** Mosher ersters.

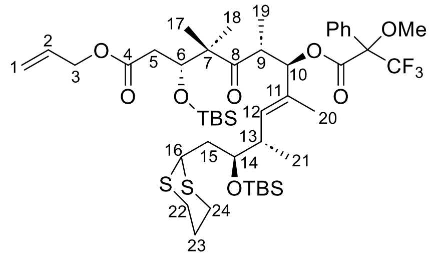

Atom	δ$_S$ in ppm	δ$_R$ in ppm	Δδ = δ$_S$ – δ$_R$
1	5.17 – 5.12 & 5.01 – 4.98	5.17 – 5.12 & 5.02 – 4.98	0 & –0.005
2	5.82 – 5.72	5.85 – 5.72	–0.015
3	4.50 – 4.48	4.50 – 4.48	0
5	2.58 – 2.38	2.60 – 2.35	+0.005
6	4.87	4.85	+0.02
9	3.30	3.33 – 3.25	+0.01
12	5.71	5.85 – 5.72	–0.075
13	2.58 – 2.38	2.60 – 2.35	+0.005
14	3.93 – 3.89	3.95 – 3.91	–0.02
15	2.07 – 1.95	2.08 – 1.96	–0.01
16	4.20	4.21	–0.01
17	1.08	1.05	+0.03
18	1.01	0.98	+0.03
19	1.17	1.06	+0.11
20	1.44	1.64	–0.2
21	0.96	0.95 – 0.93	+0.02
22	2.58 – 2.38	2.60 – 2.35	+0.005
23	1.72 – 1.59 & 1.54 – 1.47	1.71 – 1.64 & 1.55 – 1.49	–0.02 & –0.015
24	2.58 – 2.38	2.60 – 2.35	+0.005

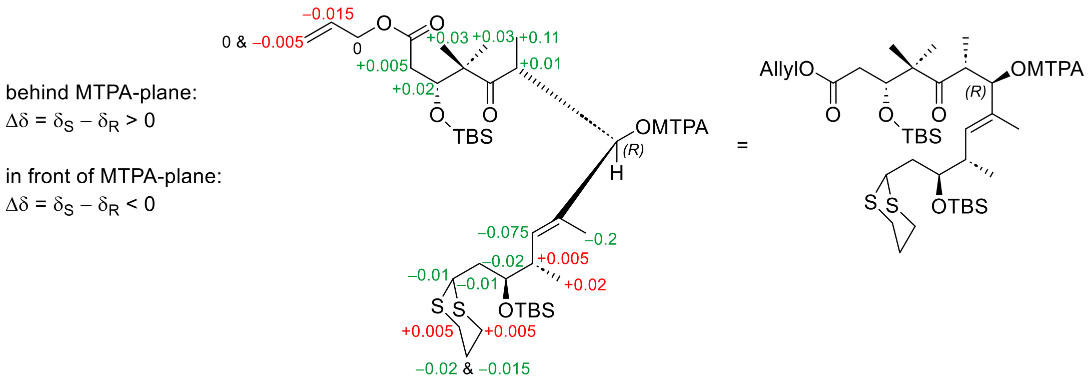

Figure S1. Assigned stereochemistry based on Mosher ester analysis. Numbers highlighted in green are in accordance with the assigned stereochemistry.

(*S*)-Mosher ester **S3**

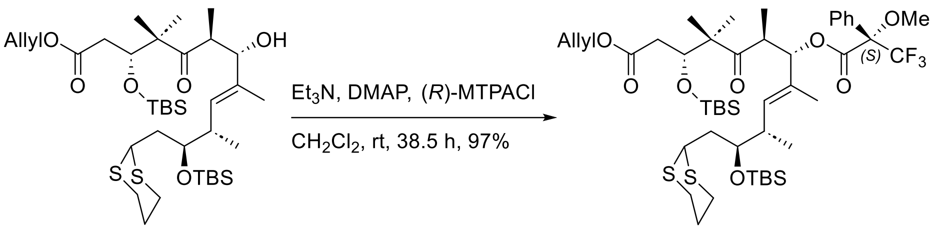

23b **S3**

(*R*)-(−)-α-Methoxy-α-(trifluoromethyl)phenylacetyl chloride (10.0 µL, 55.4 µmol, 2.65 equiv) was added to a solution of alcohol **23b** (15.0 mg, 20.9 µmol, 1.00 equiv), triethylamine (15.0 µL, 112 µmol, 5.36 equiv) and 4-dimethylaminopyridine (3.6 mg, 29.3 µmol, 1.40 equiv) in CH_2Cl_2 (0.50 mL) at room temperature. The reaction mixture was stirred for 38.5 h before it was diluted with EtOAc (5.0 mL). The organic phase was successively washed with an aqueous solution of $KHSO_4$ (1 M, 3 x), an aqueous solution of NaOH (1 M), a saturated aqueous solution of $NaHCO_3$ (3 x) and brine, dried over $MgSO_4$ and concentrated *in vacuo*. The crude product was purified *via* column chromatography (petroleum ether:EtOAc 20:1) providing (*S*)-mosher ester **S3** (19.0 mg, 20.4 µmol, 97%) as a colorless oil.

^{1}H-NMR (400 MHz, C_6D_6) δ = 7.79 – 7.73 (m, 2H), 7.23 – 7.19 (m, 2H), 7.12 – 7.08 (m, 1H), 6.01 (d, *J* = 8.2 Hz, 1H), 5.78 – 5.68 (m, 2H), 5.11 (d, *J* = 17.1 Hz, 1H), 4.97 (d, *J* = 10.6 Hz, 1H), 4.59 (dd, *J* = 6.8, 3.1 Hz, 1H), 4.48 – 4.44 (m, 2H), 4.21 (t, *J* = 7.2 Hz, 1H), 3.97 (q, *J* = 5.6 Hz, 1H), 3.57 (s, 3H), 3.36 – 3.29 (m, 1H), 2.61 – 2.35 (m, 7H), 2.10 – 2.06 (m, 2H), 1.67 (s, 3H), 1.67 – 1.55 (m, 1H), 1.52 – 1.45 (m 1H), 1.08 (s, 3H), 1.02 (s, 12H), 0.99 (d, *J* = 6.8 Hz, 3H), 0.94 (d, *J* = 6.8 Hz, 3H), 0.94 (s, 9H), 0.20 (s, 3H), 0.16 (s, 3H), 0.15 (s, 3H), 0.14 (s, 3H) ppm.

(*R*)-Mosher ester **S4**

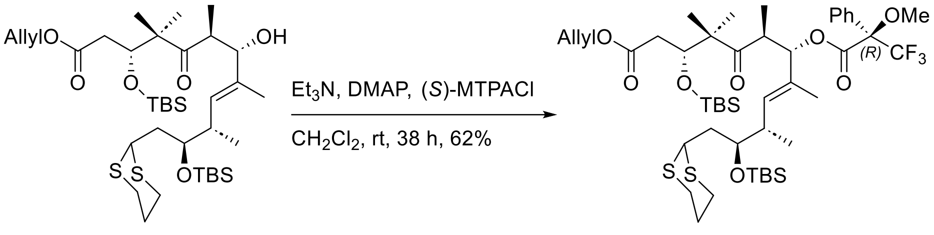

23b **S4**

(*S*)-(+)-α-Methoxy-α-(trifluoromethyl)phenylacetyl chloride (10.0 µL, 55.4 µmol, 2.65 equiv) was added to a solution of alcohol **23b** (15.0 mg, 20.9 µmol, 1.00 equiv), triethylamine (15.0 µL, 112 µmol, 5.36 equiv) and 4-dimethylaminopyridine (3.6 mg, 29.3 µmol, 1.40 equiv) in CH_2Cl_2 (0.50 mL) at room temperature. The reaction mixture was stirred for 38 h before it was diluted with EtOAc (5.0 mL). The organic phase was successively washed with an aqueous solution of $KHSO_4$ (1 M, 3 x), an aqueous solution of NaOH (1 M), a saturated aqueous solution of $NaHCO_3$ (3 x) and brine, dried over $MgSO_4$ and concentrated *in vacuo*. The crude product was purified *via* column chromatography (petroleum ether:EtOAc 20:1) providing (*S*)-mosher ester **S4** (12.0 mg, 12.9 µmol, 62%) as a colorless oil.

^{1}H-NMR (400 MHz, C_6D_6) δ = 7.84 – 7.79 (m, 2H), 7.25 – 7.21 (m, 2H), 7.13 – 7.09 (m, 1H), 5.86 (d, *J* = 7.2 Hz, 1H), 5.78 – 5.68 (m, 1H), 5.51 (d, *J* = 9.9 Hz, 1H), 5.15 – 5.09 (m, 1H), 4.99 – 4.96 (m, 1H), 4.58 (dd, *J* = 6.5, 3.2 Hz, 1H), 4.48 – 4.45 (m, 2H), 4.22 (dd, *J* = 8.2, 6.5 Hz, 1H), 3.93 (q, *J* = 5.7 Hz, 1H), 3.63 (s, 3H), 3.39 – 3.33 (m, 1H), 2.63 (dd, *J* = 16.6, 3.2 Hz, 1H), 2.55 – 2.36 (m, 6H), 2.12 – 2.00 (m, 2H), 1.68 – 1.56 (m, 1H), 1.50 (s, 3H), 1.50 – 1.46 (m, 1H), 1.13 (s, 3H), 1.12 (d, *J* = 5.3 Hz, 3H), 1.04 (s, 3H), 1.02 (s, 9H), 0.94 (s, 9H), 0.87 (d, *J* = 6.8 Hz, 3H), 0.23 (s, 3H), 0.19 (s, 3H), 0.15 (s, 3H), 0.14 (s, 3H) ppm.

Table S2. Chemical shift values of alcohols **23b** Mosher ersters.

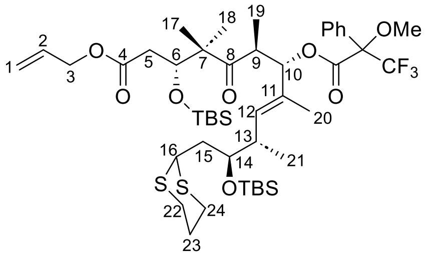

Atom	δ_S in ppm	δ_R in ppm	$\Delta\delta = \delta_S - \delta_R$
1	5.11 & 4.97	5.15 – 5.09 & 4.99 – 4.96	–0.01 & –0.005
2	5.78 – 5.68	5.78 – 5.68	0
3	4.48 – 4.44	4.48 – 4.45	–0.005
5	2.61 – 2.35	2.63 & 2.55 – 2.36	–0.15 & +0.025
6	4.59	4.58	+0.01
9	3.36 – 3.29	3.39 – 3.33	–0.035
12	5.78 – 5.68	5.51	+0.22
13	2.61 – 2.35	2.55 – 2.36	+0.025
14	3.97	3.93	+0.04
15	2.10 – 2.06	2.12 – 2.00	+0.02
16	4.21	4.22	–0.01
17	1.08	1.13	–0.05
18	1.02	1.04	–0.02
19	0.99	1.12	–0.13
20	1.67	1.50	+0.17
21	0.94	0.87	+0.07
22	2.61 – 2.35	2.55 – 2.36	+0.025
23	1.67 – 1.55 & 1.52 – 1.45	1.68 – 1.56 & 1.50 – 1.46	–0.01 & + 0.005
24	2.61 – 2.35	2.55 – 2.36	+0.025

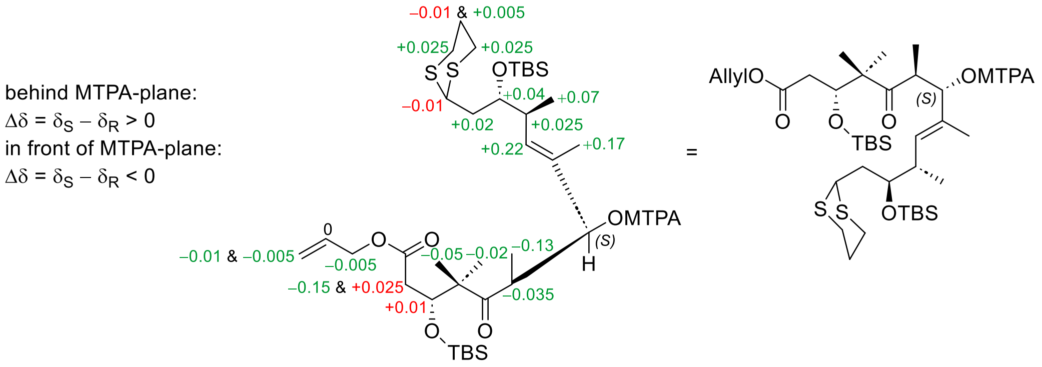

Figure S2. Assigned stereochemistry based on Mosher ester analysis. Numbers highlighted in green are in accordance with the assigned stereochemistry.

Acetonide **S5**

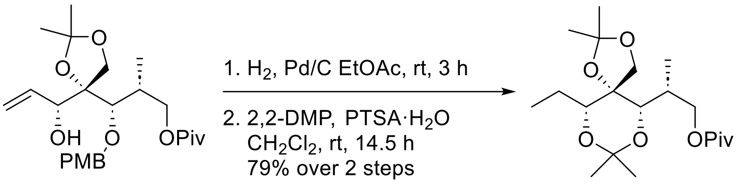

The Glassware for hydrogenation was not dried prior to usage.

Palladium on carbon (10% palladium by weight, 11.0 mg, 10.2 µmol, 0.10 equiv) was added to a solution of alcohol **78** (46.0 mg, 102 µmol, 1.00 equiv) in EtOAc (2.0 mL) at room temperature. Hydrogen was bubbled through the suspension for 15 min and stirring was continued for 2.75 h under a hydrogen atmosphere. The palladium was filtered off by using Celite® (EtOAc) and the filtrate was concentrated *in vacuo*. The crude product was purified *via* column chromatography (petroleum ether:EtOAc 4:1) providing a colorless oil (29.0 mg), used in the next reaction without further characterization.

The obtained oil (29.0 mg) was dissolved in CH_2Cl_2 (0.90 mL) and 2,2-dimethoxypropane (0.32 mL, 2.62 mmol, 25.7 equiv) and *p*-Toluenesulfonic acid monohydrate (1.7 mg, 8.72 µmol, 0.09 equiv) were added successively at room temperature. The reaction mixture was stirred for 14.5 h before a saturated aqueous solution of $NaHCO_3$ was added. The phases were separated and the aqueous layer was extracted with CH_2Cl_2 (3 x). The combined organic layers were washed with brine, dried over $MgSO_4$ and concentrated *in vacuo*. The crude product was purified *via* column chromatography (petroleum ether:EtOAc 10:1) providing acetonide **S5** (30.0 mg, 80.5 µmol, 79% over 2 steps) as a colorless oil.

^{1}H-NMR (400 MHz, $CDCl_3$) δ = 3.91 (d, *J* = 7.4 Hz, 2H), 3.74 – 3.67 (m, 3H), 3.49 (dd, *J* = 9.6, 2.9 Hz, 1H), 2.39 – 2.30 (m, 1H), 1.69 – 1.60 (m, 2H), 1.46 (s, 3H), 1.46 (s, 3H), 1.42 (s, 3H), 1.36 (s, 3H), 1.20 (s, 9H), 1.08 (d, *J* = 7.0 Hz, 3H), 0.97 (t, *J* = 7.4 Hz, 3H) ppm;

^{13}C{1H}-NMR (100 MHz, $CDCl_3$) δ = 178.4, 112.1, 98.3, 81.0, 77.0, 71.7, 68.4, 67.5, 38.9, 32.5, 29.7, 27.4, 27.0, 26.9, 22.8, 19.0, 11.8, 10.9 ppm;

HRMS (ESI) m/z: $[M+Na]^+$ calcd for $C_{20}H_{36}O_6Na$ 395.2410; found 395.2409;

$[\alpha]_D^{27.5}$ = −5.33 (*c* = 1.00, $CHCl_3$);

R_f = 0.46 (petroleum ether:EtOAc 9:1).

Stereochemistry at C17 (tedanolide C numbering, highlighted in red) was determined by the acetonide method.[3] The chemical shift values measured for the highlighted carbon atoms of the acetonide can only occur in a chair, confirming a *syn*-relation between the stereocenters at C15 and C17 and thereby the required stereochemistry.

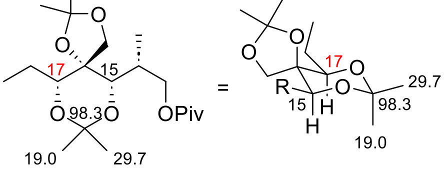

Figure S3. Determination of stereochemistry at C17 by ^{13}C-NMR chemical shift values.

Stereochemistry at C7 (tedanolide C numbering) of alcohol **48** was determined by the Mosher ester analysis,[1] which revealed the (*S*) configuration.

(*S*)-Mosher ester **S6**

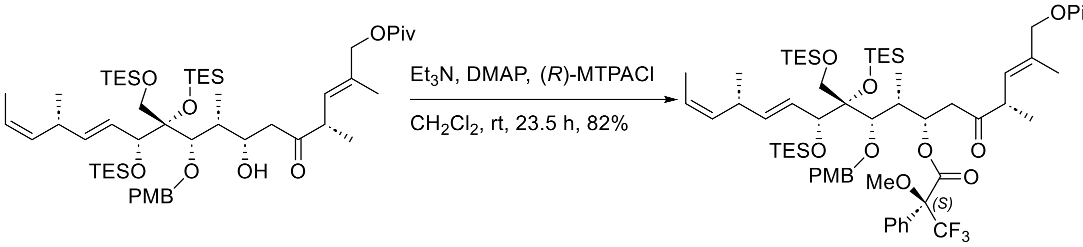

48 **S6**

(*R*)-(−)-α-Methoxy-α-(trifluoromethyl)phenylacetyl chloride (4.7 µL, 25.0 µmol, 6.00 equiv) was added to a solution of alcohol **48** (4.0 mg, 4.16 µmol, 1.00 equiv), triethylamine (5.8 µL, 41.6 µmol, 10.0 equiv) and 4-dimethylaminopyridine (2.0 mg, 16.4 µmol, 3.94 equiv) in CH_2Cl_2 (0.50 mL) at room temperature. The reaction mixture was stirred for 18 h before triethylamine (8.0 µL, 60.1 µmol, 14.4 equiv), 4-dimethylaminopyridine (4.0 mg, 32.7 µmol, 7.87 equiv) and (*R*)-(−)-α-Methoxy-α-(trifluoromethyl)phenylacetyl chloride (6.0 µL, 32.1 µmol, 7.71 equiv) were added successively. The reaction mixture was stirred for 5.5 h before it was diluted with EtOAc (5.0 mL). The organic phase was successively washed with an aqueous solution of NaOH (1 M), a saturated aqueous solution of $NaHCO_3$ (3 x), an aqueous solution of $CuSO_4$ (1 M) and brine, dried over $MgSO_4$ and concentrated *in vacuo*. The crude product was purified *via* column chromatography (petroleum ether:EtOAc 20:1) providing (*S*)-mosher ester **S6** (4.0 mg, 3.40 µmol, 82%) as a colorless oil.

^{1}H-NMR (400 MHz, C_6D_6) δ = 7.76 – 7.72 (m, 2H), 7.50 – 7.47 (m, 2H), 7.20 – 7.11 (m, 2H), 7.08 – 7.05 (m, 1H), 6.91 – 6.87 (m, 2H), 6.02 – 5.94 (m, 2H), 5.67 (dd, *J* = 15.7, 5.8 Hz, 1H), 5.52 – 5.43 (m, 1H), 5.42 – 5.36 (m, 1H), 5.22 – 5.19 (m, 1H), 4.91 (d, *J* = 9.6 Hz, 1H), 4.86 (d, *J* = 11.8 Hz, 1H), 4.57 (d, *J* = 11.8 Hz, 1H), 4.41 (d, *J* = 12.5 Hz, 1H), 4.36 (d, *J* = 12.5 Hz, 1H), 4.19 (d, *J* = 9.8 Hz, 1H), 4.02 (d, *J* = 3.4 Hz, 1H), 3.93 (d, *J* = 9.8 Hz, 1H), 3.46 (s, 3H), 3.32 – 3.24 (m, 1H), 3.31 (s, 3H), 3.21 – 3.11 (m, 2H), 3.09 – 3.01 (m, 1H), 2.72 (dd, *J* = 17.8, 6.2 Hz, 1H), 1.58 (dd, *J* = 6.6, 1.4 Hz, 3H), 1.53 (d, *J* = 1.4 Hz, 3H), 1.29 (d, *J* = 7.3 Hz, 3H), 1.18 (s, 9H), 1.18 – 1.11 (m, 30H), 1.04 (d, *J* = 6.7 Hz, 3H), 0.92 – 0.77 (m, 18H) ppm.

(*R*)-Mosher ester **S7**

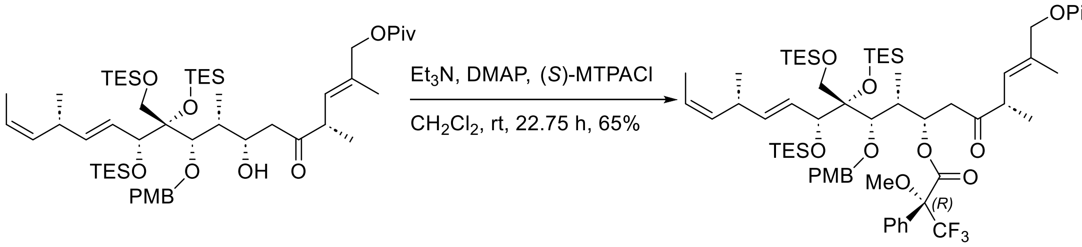

48 **S7**

(*S*)-(+)-α-Methoxy-α-(trifluoromethyl)phenylacetyl chloride (4.7 µL, 25.0 µmol, 6.00 equiv) was added to a solution of alcohol **48** (4.0 mg, 4.16 µmol, 1.00 equiv), triethylamine (5.8 µL, 41.6 µmol, 10.0 equiv) and 4-dimethylaminopyridine (2.0 mg, 16.4 µmol, 3.94 equiv) in CH_2Cl_2 (0.50 mL) at room temperature. The reaction mixture was stirred for 22.75 h before it was diluted with EtOAc (5.0 mL).

The organic phase was successively washed with an aqueous solution of $CuSO_4$ (1 M), an aqueous solution of NaOH (1 M), a saturated aqueous solution of $NaHCO_3$ (3 x) and brine, dried over $MgSO_4$ and concentrated *in vacuo*. The crude product was purified *via* column chromatography (petroleum ether:EtOAc 20:1) providing (*R*)-mosher ester **S7** (3.2 mg, 2.72 µmol, 65%) as a colorless oil.

^{1}H-NMR (400 MHz, C_6D_6) δ = 7.84 – 7.80 (m, 2H), 7.45 – 7.41 (m, 2H), 7.23 – 7.19 (m, 2H), 7.12 – 7.08 (m, 1H), 6.91 – 6.88 (m, 2H), 5.96 – 5.84 (m, 2H), 5.65 (dd, *J* = 15.7, 5.3 Hz, 1H), 5.49 – 5.42 (m, 1H), 5.38 – 5.29 (m, 2H), 4.88 (d, *J* = 9.5 Hz, 1H), 4.75 (d, *J* = 11.6 Hz, 1H), 4.41 (d, *J* = 12.8 Hz, 1H), 4.38 (d, *J* = 12.8 Hz, 1H), 4.15 – 4.12 (m, 2H), 3.93 (d, *J* = 9.7 Hz, 1H), 3.82 (d, *J* = 3.2 Hz, 1H), 3.55 (s, 3H), 3.31 (s, 3H), 3.24 – 3.17 (m, 3H), 3.09 – 3.02 (m, 1H), 2.83 (dd, *J* = 17.6, 6.8 Hz, 1H), 1.56 (dd, *J* = 6.7, 1.5 Hz, 3H), 1.53 (d, *J* = 1.2 Hz, 3H), 1.31 (d, *J* = 7.2 Hz, 3H), 1.19 (s, 9H), 1.19 – 1.07 (m, 33H), 0.88 – 0.72 (m, 18H) ppm.

Table S3. Chemical shift values of alcohols **48** Mosher ersters.

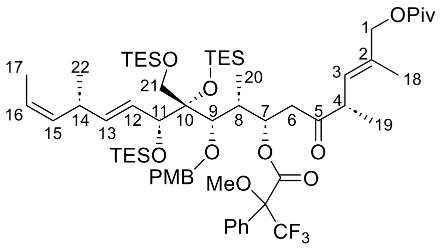

Atom	δS in ppm	δR in ppm	Δδ = δS – δR
1	4.41 & 4.36	4.41 & 4.38	0 & –0.02
3	5.22 – 5.19	5.38 – 5.29	–0.13
4	3.21 – 3.11	3.24 – 3.17	–0.045
6	3.21 – 3.11 & 2.72	3.24 – 3.17 & 2.83	–0.045 & –0.11
8	3.09 – 3.01	3.09 – 3.02	–0.005
9	4.02	3.82	+0.2
11	4.91	4.88	+0.03
12	6.02 – 5.94	5.96 – 5.84	+0.08
13	5.67	5.65	+0.02
14	3.32 – 3.24	3.24 – 3.17	+0.075
15	5.42 – 5.36	5.38 – 5.29	+0.055
16	5.52 – 5.43	5.49 – 5.42	+0.02
17	1.58	1.56	+0.02
18	1.53	1.53	0
19	1.04	1.08 (COSY & HSQC)	–0.04
20	1.29	1.31	–0.02
21	4.19 & 3.93	4.15 – 4.12 & 3.93	+0.055 & 0
22	1.13 (COSY & HSQC)	1.09 (COSY & HSQC)	+0.04
Piv	1.18	1.19	–0.01

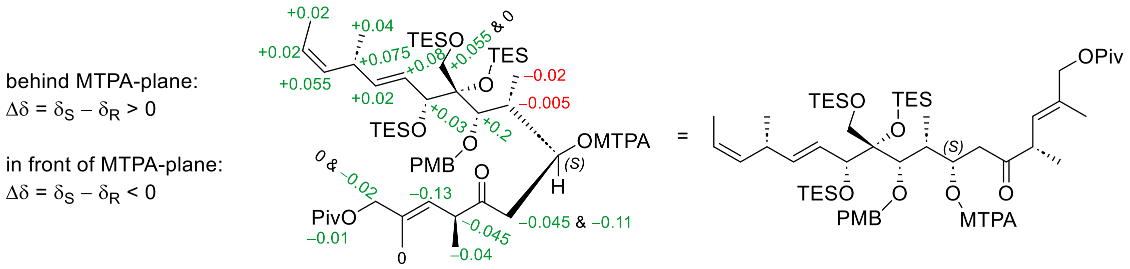

Figure S4. Assigned stereochemistry based on Mosher ester analysis. Numbers highlighted in green are in accordance with the assigned stereochemistry.

Stereochemistry at C11 (tedanolide C numbering, highlighted in red) of Diol **85** was determined by the acetonide method (synthesis of acetonide **49** and its analytical data are given in the manuscript).[3] The chemical shift values measured for the highlighted carbon atoms of the acetonide are typical for a twisted system, confirming an *anti*-relation between the stereocenters at C11 and C13 and thereby the required stereochemistry.

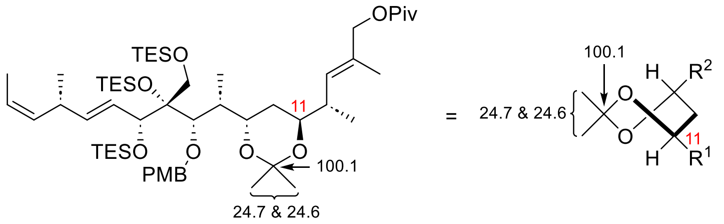

49

Figure S5. Determination of stereochemistry at C11 by ^{13}C-NMR chemical shift values.

PMP-acetal **S8**

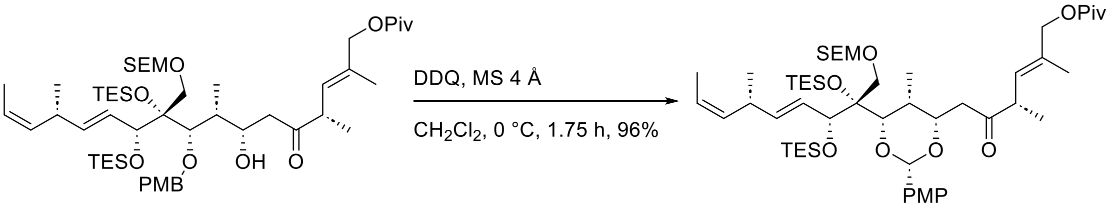

58 **S8**

A suspension of alcohol **58** (23.0 mg, 23.5 µmol, 1.00 equiv) and activated molecular sieves (4 Å, powdered, 29.0 mg) in CH_2Cl_2 (0.80 mL) was stirred for 1 h at room temperature before it was cooled to 0 °C. 2,3-Dichloro-5,6-dicyano-1,4-benzoquinone (12.0 mg, 51.1 µmol, 2.17 equiv) was added and the reaction mixture was stirred for 45 min before the subsequent addition of a saturated aqueous solution of $NaHCO_3$ and saturated aqueous solution of $Na_2S_2O_3$. The phases were separated and the aqueous layer was extracted with CH_2Cl_2 (3 x). The combined organic layers were washed with brine, dried over $MgSO_4$ and concentrated *in vacuo*. The crude product was purified *via* column chromatography (petroleum ether:EtOAc 10:1) providing PMP-acetal **S8** (22.0 mg, 22.6 µmol, 96%) as a colorless oil.

^{1}H NMR (500 MHz, C_6D_6) δ = 7.61 – 7.59 (m, 2H), 6.87 – 6.83 (m, 2H), 6.01 (dd, *J* = 15.6, 8.0 Hz, 1H), 5.78 (dd, *J* = 15.6, 6.1 Hz, 1H), 5.64 (s, 1H), 5.54 – 5.48 (m, 1H), 5.43 – 5.39 (m, 1H), 5.31 (d, *J* = 9.7 Hz, 1H),

4.76 – 4.69 (m, 3H), 4.58 – 4.55 (m, 1H), 4.36 (d, J = 12.6 Hz, 1H), 4.32 (d, J = 12.6 Hz, 1H), 4.31 (s, 1H), 4.05 (d, J = 9.6 Hz, 1H), 3.99 (d, J = 9.6 Hz, 1H), 3.76 – 3.72 (m, 2H), 3.36 – 3.29 (m, 1H), 3.27 (s, 3H), 3.17 – 3.11 (m, 1H), 2.81 (dd, J = 17.0, 7.1 Hz, 1H), 2.39 (dd, J = 17.0, 6.0 Hz, 1H), 2.27 – 2.22 (m, 1H), 1.64 (d, J = 6.7 Hz, 3H), 1.46 (d, J = 0.9 Hz, 3H), 1.41 – 1.40 (m, 3H), 1.17 – 1.15 (m, 3H), 1.15 (s, 9H), 1.14 – 1.09 (m, 18H), 1.03 – 1.00 (m, 5H), 0.87 – 0.74 (m, 12H), 0.05 (s, 9H) ppm;

^{13}C{1H}-NMR (125 MHz, C_6D_6) δ = 207.3, 177.3, 160.5, 137.9, 134.7, 133.5, 132.1, 128.3, 128.3, 127.6, 123.5, 113.7, 103.3, 96.4, 84.8, 82.0, 78.6, 77.7, 69.0, 68.6, 66.1, 54.7, 46.9, 43.6, 38.9, 34.8, 33.3, 27.4, 20.8, 18.4, 16.3, 14.1, 13.2, 8.9, 7.8, 7.4, 7.2, 6.1, −1.2 ppm;

HRMS (ESI) m/z: $[M+Na]^+$ calcd for $C_{53}H_{94}O_{10}Si_3Na$ 997.6053; found 997.6047;

$[\alpha]_D^{20.5}$ = +78.1 (c = 0.32, $CHCl_3$);

R_f = 0.39 (petroleum ether:EtOAc 9:1).

Stereochemistry at C13 (Tedanolide C numbering) was determined by nOe correlations of the protons at C13, C14, C15 and the benzylic position of PMP-acetal **S8**. The shown correlations indicate that it is very likely that all protons except the one at C14 occupy axial positions in a chair conformation leading to a *syn*-relation of the stereocenters at C13 and C15 and thereby the required stereochemistry.

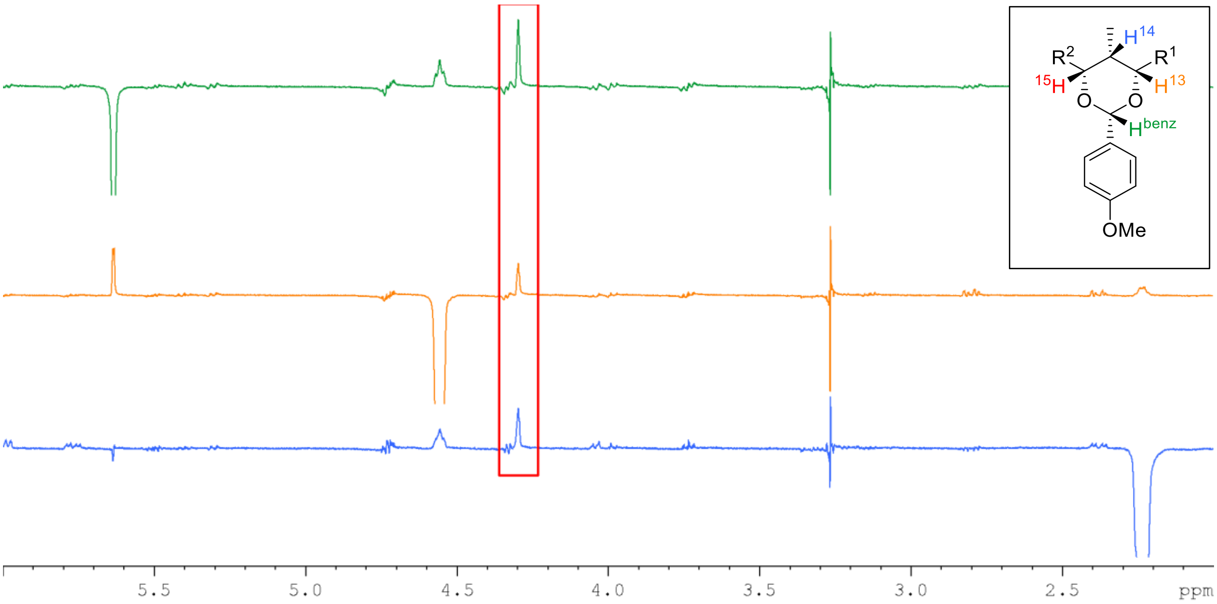

Figure S6. NOe correlations of PMP-acetal **S8**.

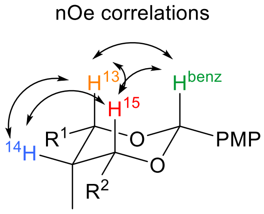

Figure S7. Graphical representation of the nOe correlations of PMP-acetal **S8**.

Acetonide **S9**

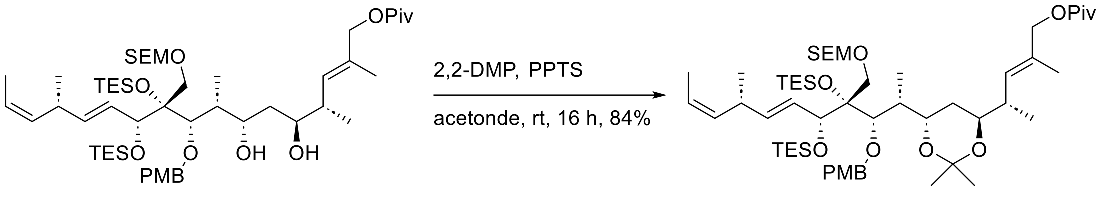

The glassware was not dried prior to usage.

p-Toluenesulfonic acid monohydrate (1.7 mg, 6.76 µmol, 0.74 equiv) was added to a solution of diol **92** (9.0 mg, 9.19 µmol, 1.00 equiv) and 2,2-dimethoxypropan (0.14 mL, 1.10 mmol, 120 equiv) in acetone (0.14 mL) at room temperature. The reaction mixture was stirred for 16 h before the subsequent addition of a saturated aqueous solution of $NaHCO_3$ and MTBE. The phases were separated and the aqueous layer was extracted with MTBE (3 x). The combined organic layers were washed with brine, dried over $MgSO_4$ and concentrated *in vacuo*. The crude product was purified *via* column chromatography (petroleum ether:EtOAc 20:1) providing acetonide **S9** (7.9 mg, 7.75 µmol, 84%) as a colorless oil.

^{1}H-NMR (500 MHz, $CDCl_3$) δ = 7.27 – 7.24 (m, 2H), 6.85 – 6.82 (m, 2H), 5.59 (ddd, *J* = 15.5, 7.8, 1.3 Hz, 1H), 5.49 (dd, *J* = 15.5, 5.7 Hz, 1H), 5.44 – 5.38 (m, 1H), 5.23 – 5.16 (m, 2H), 4.63 (s, 2H), 4.60 (d, *J* = 11.1 Hz, 1H), 4.43 (d, *J* = 12.2 Hz, 1H), 4.40 (d, *J* = 11.1 Hz, 1H), 4.39 (d, *J* = 12.2 Hz, 1H), 4.34 (d, *J* = 7.7 Hz, 1H), 3.79 (s, 3H), 3.75 – 3.71 (m, 1H), 3.69 – 3.56 (m, 4H), 3.51 (d, *J* = 1.8 Hz, 1H), 3.48 – 3.43 (m, 1H), 3.21 – 3.14 (m, 1H), 2.46 – 2.35 (m, 2H), 1.72 – 1.66 (m, 1H), 1.65 (d, *J* = 1.2 Hz, 3H), 1.61 (dd, *J* = 6.8, 1.8 Hz, 3H), 1.44 – 1.37 (m, 1H), 1.33 (s, 3H), 1.30 (s, 3H), 1.21 (s, 9H), 1.04 (d, *J* = 6.8 Hz, 3H), 0.98 (d, *J* = 6.6 Hz, 3H), 0.94 – 0.89 (m, 23H), 0.63 (q, *J* = 7.9 Hz, 6H), 0.56 (q, *J* = 8.0 Hz, 6H), 0.00 (s, 9H) ppm;

^{13}C{1H}-NMR (125 MHz, $CDCl_3$) δ = 178.4, 158.7, 137.1, 134.4, 132.6, 130.8, 130.7, 128.6, 128.2, 123.0, 113.5, 100.2, 96.2, 84.2, 83.2, 76.2, 73.5, 71.1,[1] 69.9, 69.0, 66.3, 55.4, 39.0, 37.9, 36.8, 34.5, 34.1, 27.4, 24.8, 24.6, 20.5, 18.3, 17.0, 14.4, 13.0, 8.7, 7.7, 7.1, 7.1, 5.6, −1.3 ppm;

HRMS (ESI) m/z: $[M+Na]^+$ calcd for $C_{56}H_{102}O_{10}Si_3Na$ 1041.6679; found 1041.6676;

$[\alpha]_D^{20.7}$ = +36.4 (*c* = 0.11, $CHCl_3$);

R_f = 0.37 (petroleum ether:EtOAc 19:1).

Stereochemistry at C11 (tedanolide C numbering, highlighted in red) of Diol **92** was determined by the acetonide method.[3] The chemical shift values measured for the highlighted carbon atoms of the acetonide are typical for a twisted system, confirming an *anti*-relation between the stereocenters at C11 and C13 and thereby the required stereochemistry.

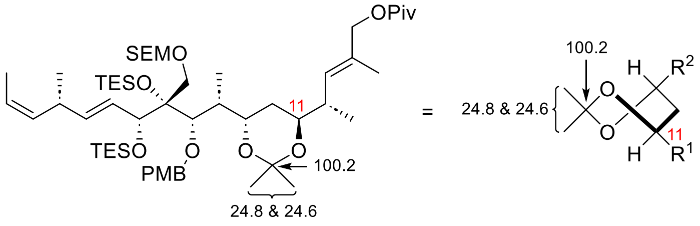

S9

Figure S8. Determination of stereochemistry at C11 by ^{13}C-NMR chemical shift values.

[1] Two different carbon atoms.

Stereochemistry at C7 (tedanolide C numbering) of alcohol **95** was determined by the Mosher ester analysis,[1] which revealed the (*R*) configuration. For the methyl group at C6 a *syn* relation was assumed, since titanium tetrachloride derived enolates usually provide *syn* aldol products.[2]

(*S*)-Mosher ester **S10**

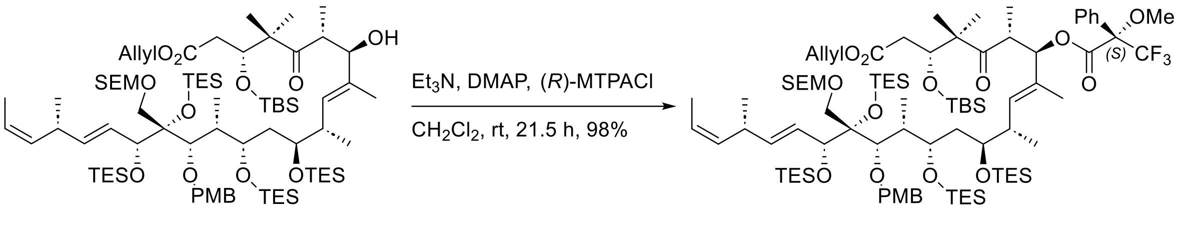

(*R*)-(−)-α-Methoxy-α-(trifluoromethyl)phenylacetyl chloride (5.1 µL, 27.3 µmol, 6.00 equiv) was added to a solution of alcohol **95** (10.0 mg, 6.83 µmol, 1.00 equiv), triethylamine (7.6 µL, 54.6 µmol, 10.0 equiv) and 4-dimethylaminopyridine (1.2 mg, 9.56 µmol, 1.40 equiv) in CH_2Cl_2 (0.50 mL) at room temperature. The reaction mixture was stirred for 14 h before triethylamine (0.05 mL, 361 µmol, 52.8 equiv), 4-dimethylaminopyridine (5.0 mg, 40.9 µmol, 5.99 equiv) and (*R*)-(−)-α-Methoxy-α-(trifluoromethyl)phenylacetyl chloride (20.0 µL, 107 µmol, 15.6 equiv) were added successively. The reaction mixture was stirred for 5.5 h before 4-dimethylaminopyridine (10.0 mg, 81.9 µmol, 12.0 equiv) was added. The reaction mixture was stirred for 2 h before it was diluted with EtOAc (5.0 mL). The organic phase was successively washed with an aqueous solution of $KHSO_4$ (1 M, 3 x), an aqueous solution of NaOH (1 M), a saturated aqueous solution of $NaHCO_3$ and brine, dried over $MgSO_4$ and concentrated *in vacuo*. The crude product was purified *via* column chromatography (petroleum ether:EtOAc 20:1) providing (*S*)-mosher ester **S10** (11.2 mg, 6.66 µmol, 98%) as a colorless oil.

^{1}H-NMR (400 MHz, C_6D_6) δ = 7.86 – 7.82 (m, 2H), 7.57 – 7.53 (m, 2H), 7.26 – 7.21 (m, 2H), 7.16 – 7.10 (m, 1H), 6.94 – 6.91 (m, 2H), 6.10 (d, *J* = 9.1 Hz, 1H), 6.08 (dd, *J* = 15.4, 9.4 Hz, 1H), 5.98 (d, *J* = 5.1 Hz, 1H), 5.80 – 5.70 (m, 2H), 5.52 – 5.39 (m, 2H), 5.14 (dq, *J* = 17.2, 1.5 Hz, 1H), 5.06 (d, *J* = 11.8 Hz, 1H), 5.00 – 4.97 (m, 1H), 4.94 – 4.91 (m, 2H), 4.85 (d, *J* = 6.2 Hz, 1H), 4.80 (d, *J* = 6.2 Hz, 1H), 4.68 (d, *J* = 11.8 Hz, 1H), 4.50 – 4.48 (m, 2H), 4.20 (d, *J* = 9.7 Hz, 1H), 4.07 – 4.03 (m, 1H), 4.01 – 3.97 (m, 2H), 3.87 – 3.75 (m, 2H), 3.65 (d, *J* = 9.7 Hz, 1H), 3.58 (s, 3H), 3.43 – 3.38 (m, 1H), 3.36 – 3.27 (m, 1H), 3.34 (s, 3H), 2.69 – 2.61 (m, 1H), 2.55 (dd, *J* = 16.4, 3.2 Hz, 1H), 2.54 – 2.47 (m, 1H), 2.45 (dd, *J* = 16.4, 7.1 Hz, 1H), 2.22 – 2.15 (m, 1H), 2.12 – 2.05 (m, 1H), 1.77 (s, 3H), 1.59 (d, *J* = 5.2 Hz, 3H), 1.41 (d, *J* = 6.9 Hz, 3H), 1.28 (d, *J* = 6.8 Hz, 3H), 1.22 (d, *J* = 7.0 Hz, 3H), 1.21 (s, 3H), 1.21 – 1.05 (m, 44H) 0.97 (s, 9H), 0.94 – 0.77 (m, 24H), 0.23 (s, 3H), 0.21 (s, 3H), 0.08 (s, 9H) ppm.

(*R*)-Mosher ester **S11**

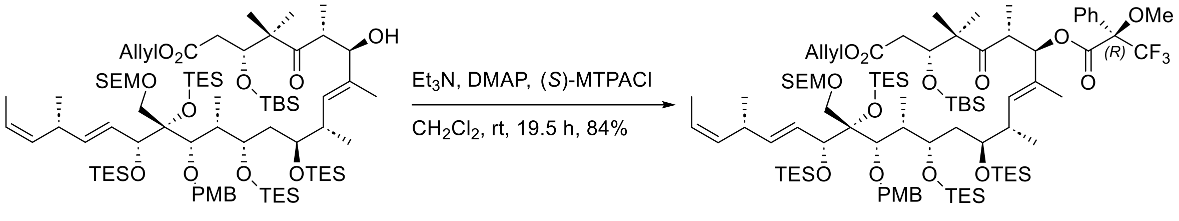

(*S*)-(+)-α-Methoxy-α-(trifluoromethyl)phenylacetyl chloride (5.1 µL, 27.3 µmol, 6.00 equiv) was added to a solution of alcohol **95** (10.0 mg, 6.83 µmol, 1.00 equiv), triethylamine (7.6 µL, 54.6 µmol, 10.0 equiv) and 4-dimethylaminopyridine (1.2 mg, 9.56 µmol, 1.40 equiv) in CH_2Cl_2 (0.50 mL) at room temperature. The reaction mixture was stirred for 14 h before triethylamine (0.05 mL, 361 µmol, 52.8 equiv), 4-dimethylaminopyridine (10.0 mg, 81.9 µmol, 12.0 equiv) and (*S*)-(+)-α-Methoxy-α-(trifluoromethyl)phenylacetyl chloride (20.0 µL, 107 µmol, 15.6 equiv) were added successively. The reaction mixture was stirred for 5.5 h before it was diluted with EtOAc (5.0 mL). The organic phase was successively washed with an aqueous solution of $KHSO_4$ (1 M, 3 x), an aqueous solution of NaOH (1 M), a saturated aqueous solution of $NaHCO_3$ and brine, dried over $MgSO_4$ and concentrated *in vacuo*. The crude product was purified *via* column chromatography (petroleum ether:EtOAc 20:1) providing (*R*)-mosher ester **S11** (9.6 mg, 5.71 µmol, 84%) as a colorless oil.

^{1}H-NMR (400 MHz, C_6D_6) δ = 7.78 – 7.74 (m, 2H), 7.58 – 7.53 (m, 2H), 7.19 – 7.16 (m, 2H), 7.10 – 7.04 (m, 1H), 6.94 – 6.91 (m, 2H), 6.13 (d, *J* = 7.0 Hz, 1H), 6.10 (dd, *J* = 16.6, 9.5 Hz, 1H), 6.02 (d, *J* = 6.4 Hz, 1H), 5.80 – 5.68 (m, 2H), 5.52 – 5.40 (m, 2H), 5.13 (dq, *J* = 17.2, 1.5 Hz, 1H), 5.06 (d, *J* = 11.8 Hz, 1H), 5.00 – 4.97 (m, 1H), 4.92 (d, *J* = 9.1 Hz, 1H), 4.89 (dd, *J* = 7.1, 3.1 Hz, 1H), 4.86 (d, *J* = 6.0 Hz, 1H), 4.82 (d, *J* = 6.0 Hz, 1H), 4.74 (d, *J* = 11.8 Hz, 1H), 4.49 – 4.47 (m, 2H), 4.19 (d, *J* = 9.6 Hz, 1H), 4.08 – 4.03 (m, 1H), 4.03 – 3.98 (m, 2H), 3.85 – 3.74 (m, 2H), 3.70 (d, *J* = 9.6 Hz, 1H), 3.66 (s, 3H), 3.36 – 3.29 (m, 2H), 3.31 (s, 3H), 2.75 – 2.66 (m, 1H), 2.56 – 2.49 (m, 1H), 2.55 (dd, *J* = 16.5, 3.1 Hz, 1H), 2.44 (dd, *J* = 16.5, 7.1 Hz, 1H), 2.24 – 2.17 (m, 1H), 2.10 – 2.03 (m, 1H), 1.96 (s, 3H), 1.59 (d, *J* = 5.3 Hz, 3H), 1.41 (d, *J* = 7.2 Hz, 3H), 1.21 – 1.05 (m, 53H), 0.96 (s, 9H), 0.95 – 0.78 (m, 24H), 0.22 (s, 3H), 0.18 (s, 3H), 0.07 (s, 9H) ppm.

Table S4. Chemical shift values of alcohols **95** Mosher ersters.

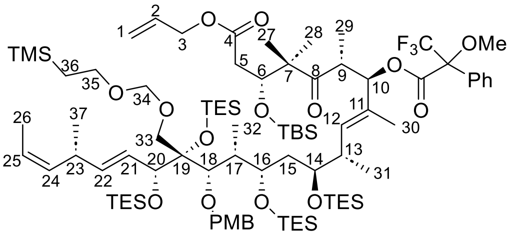

Atom	δ_S in ppm	δ_R in ppm	$\Delta\delta = \delta_S - \delta_R$
1	5.14 & 5.00 – 4.97	5.13 & 5.00 – 4.97	+0.01 & 0
2	5.80 – 5.70	5.80 – 5.68	+0.01
3	4.50 – 4.48	4.49 – 4.47	+0.01
5	2.55 & 2.45	2.55 & 2.44	0 & + 0.01
6	4.94 – 4.91	4.89	+0.035
9	3.43 – 3.38	3.36 – 3.29	+0.08
12	6.10	6.13	–0.03
13	2.69 – 2.61	2.75 – 2.66	–0.055
14	4.01 – 3.97	4.03 – 3.98	–0.015
15	2.22 – 2.15 & 2.12 – 2.05	2.24 – 2.17 & 2.10 – 2.03	–0.02 & +0.02
16	4.07 – 4.03	4.08 – 4.03	–0.005
17	2.54 – 2.47	2.56 – 2.49	–0.02
18	4.01 – 3.97	4.03 – 3.98	–0.015
20	4.94 – 4.91	4.92	+0.005
21	6.08	6.10	–0.02
22	5.80 – 5.70	5.80 – 5.68	+0.01
23	3.36 – 3.27	3.36 – 3.29	–0.01
24	5.52 – 5.39	5.52 – 5.40	–0.005
25	5.52 – 5.39	5.52 – 5.40	–0.005
26	1.59	1.59	0
27	1.21	1.17	+0.04
28	1.14	1.09	+0.05
29	1.28	1.12 (COSY & HMBC)	+0.16
30	1.77	1.96	–0.2
31	1.22 (COSY & HSQC)	1.20	+0.02
32	1.41	1.41	0
33	4.20 & 3.65	4.19 & 3.70	+0.01 & –0.05
34	4.85 & 4.80	4.86 & 4.82	–0.01 & –0.02
35	3.87 – 3.75	3.85 – 3.74	+0.015
37	1.16 (COSY & HSQC)	1.16 (COSY & HSQC)	0
TBS (Me)	0.23 & 0.21	0.22 & 0.18	+0.01 & 0
TBS (^{t}Bu)	0.97	0.96	+0.01
TMS	0.08	0.07	+0.01

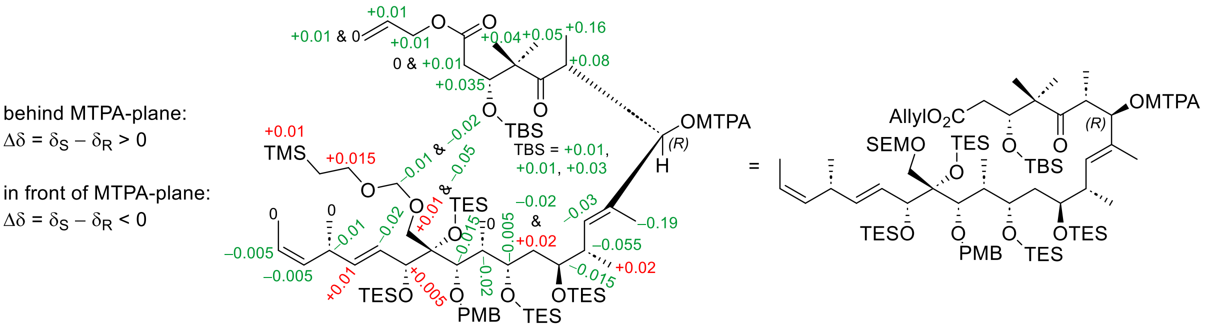

Figure S9. Assigned stereochemistry based on Mosher ester analysis. Numbers highlighted in green are in accordance with the assigned stereochemistry.

2. Comparison of NMR data of isolated tedanolide C (**8**) and desepoxy-tedanolide **66**

Table S5. Comparison of NMR-data of isolated tedanolide C (**8**) and desepoxy-tedanolide C (**66**).

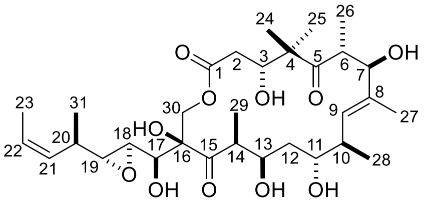

tedanolid C (**8**)
(proposed structure)

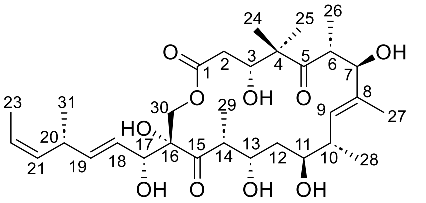

desepoxy-tedanolide C (**66**)

Atom	Tedanolide C δ_H in ppm, mult (J in Hz)[4]	Desepoxy-tedanolide C δ_H in ppm, mult (J in Hz)	Tedanolide C δ_C in ppm[4]	Desepoxy-tedanolide C δ_C in ppm
1			172.6	-
2	2.26, dd (14.6, 11.2) & 2.17, dd (14.6, 2.8)	2.23 & 2.16 (COSY)	39.3	39.2
3	4.14, dd (11.2, 2.8)	4.15, dd (10.8, 3.1)	73.1	73.0
4			53.2	53.0
5			220.5	-
6	3.21, dq (9.2, 7.2)	3.27 – 3.21, m	46.9	46.8
7	4.06, d (9.2)	4.06, d (9.6)	81.3	81.1
8			136.0	135.5
9	5.18, d (9.2)	5.19, d (9.4)	134.1	134.1
10	2.20, ddq (9.2, 6.9, <1.0)	2.21 (COSY + HSQC)	40.4	40.2
11	3.53, dd (10.3, 2.9)	3.56 – 3.52, m	73.8	73.5
12	1.40, ddd (15.3, 9.9, 2.9) & 1.15, ddd (15.3, 10.3, 3.6)	1.41 & 1.17 (COSY + HSQC)	41.5	41.4
13	4.05, ddd (9.9, 3.6, 3.3)	4.08 – 4.04, m	70.2	70.2
14	3.31, dq (7.2, 3.3)	3.31 (overlapped by CD_3OD)	48.9	49.0 (overlapped by CD_3OD)
15			219.7	220.2
16			84.5	-
17	3.51, d (7.4)	4.27, d (8.4)	75.1	75.7
18	2.99, dd (7.4, 2.0)	5.57, ddd (15.8, 8.4, 1.3)	59.1	126.7
19	2.74, dd (6.9, 2.0)	5.68, dd (15.8, 6.2)	62.3	140.3
20	2.41, ddq (10.3, 6.9, 6.9)	3.27 – 3.21, m	35.3	35.3
21	5.27, ddq (10.8, 10.3, 1.6)	5.26 – 5.21, m	131.6	135.0
22	5.54, dq (10.8, 6.8)	5.47 – 5.41, m	127.1	124.3
23	1.61, dd (6.8, 1.6)	1.63, dd (6.8, 1.8)	13.5	13.2
24	1.16, s	1.24 or 1.17, s	25.0	25.0 (1.17) or 18.5 (1.24)
25	1.22, s	1.24 or 1.17, s	18.7	25.0 (1.17) or 18.5 (1.24)
26	1.21, d (7.2)	1.23, d (6.4)	17.6	17.4
27	1.54, s	1.56, d (1.2)	11.3	11.1
28	0.97, d (6.8)	0.99, d (6.8)	17.7	17.5
29	1.21, d (7.2)	1.22, d (6.8)	12.6	12.6
30	4.18, d (11.0) & 4.02, d (11.0)	3.96, d (11.1) & 3.88, d (11.1)	70.3	70.9
31	1.06, d (6.9)	1.07, d (6.9)	18.0	21.2

3. References

[1] a) Dale, J. A.; Dull, D. L.; Mosher, H. S. .alpha.-Methoxy-.alpha.-trifluoromethylphenylacetic acid, a versatile reagent for the determination of enantiomeric composition of alcohols and amines *J. Org. Chem.* **1969**, *34*, 2543. b) Hoye, T. R.; Jeffrey, C. S.; Shao, F. Mosher ester analysis for the determination of absolute configuration of stereogenic (chiral) carbinol carbons *Nat. Protoc.* **2007**, *2*, 2451.

[2] a) Harrison, C. R. Transient titanium enolate aldol condensations *Tetrahedron Lett.* **1987**, *28*, 4135. b) Evans, D. A.; Clark, J. S.; Metternich, R.; Novack, V. J.; Sheppard, G. S. Diastereoselective aldol reactions using .beta.-keto imide derived enolates. A versatile approach to the assemblage of polypropionate systems *J. Am. Chem. Soc.* **1990**, *112*, 866. c) Evans, D. A.; Rieger, D. L.; Bilodeau, M. T.; Urpi, F. Stereoselective aldol reactions of chlorotitanium enolates. An efficient method for the assemblage of polypropionate-related synthons *J. Am. Chem. Soc.* **1991**, *113*, 1047.

[3] a) Rychnovsky, S. D.; Skalitzky, D. J. Stereochemistry of alternating polyol chains: ^{13}C NMR analysis of 1,3-diol acetonides *Tetrahedron Lett.* **1990**, *31*, 945. b) Evans, D. A.; Rieger, D. L.; J. R. Gage, ^{13}C NMR chemical shift correlations in 1,3-diol acetonides. Implications for the stereochemical assignment of propionate-derived polyols *Tetrahedron Lett.* **1990**, *31*, 7099. c) Rychnovsky, S. D.; Rogers, B. N.; Richardson, T. I. Configurational Assignment of Polyene Macrolide Antibiotics Using the [^{13}C]Acetonide Analysis *Acc. Chem. Res.* **1998**, *31*, 9.

[4] Chevallier, C.; Bugni, T. S.; Feng, X.; Harper, M. K.; Orendt, A. M.; Ireland, C. M. Tedanolide C: A Potent New 18-Membered Ring Cytotoxic Macrolide Isolated from Papua New Guinea Marine Sponge *Ircinia* sp. *J. Org. Chem*. **2006**, *71*, 2510.

4. NMR data

Northern fragment **11**

^{1}H-NMR (400 MHz, $CDCl_3$)

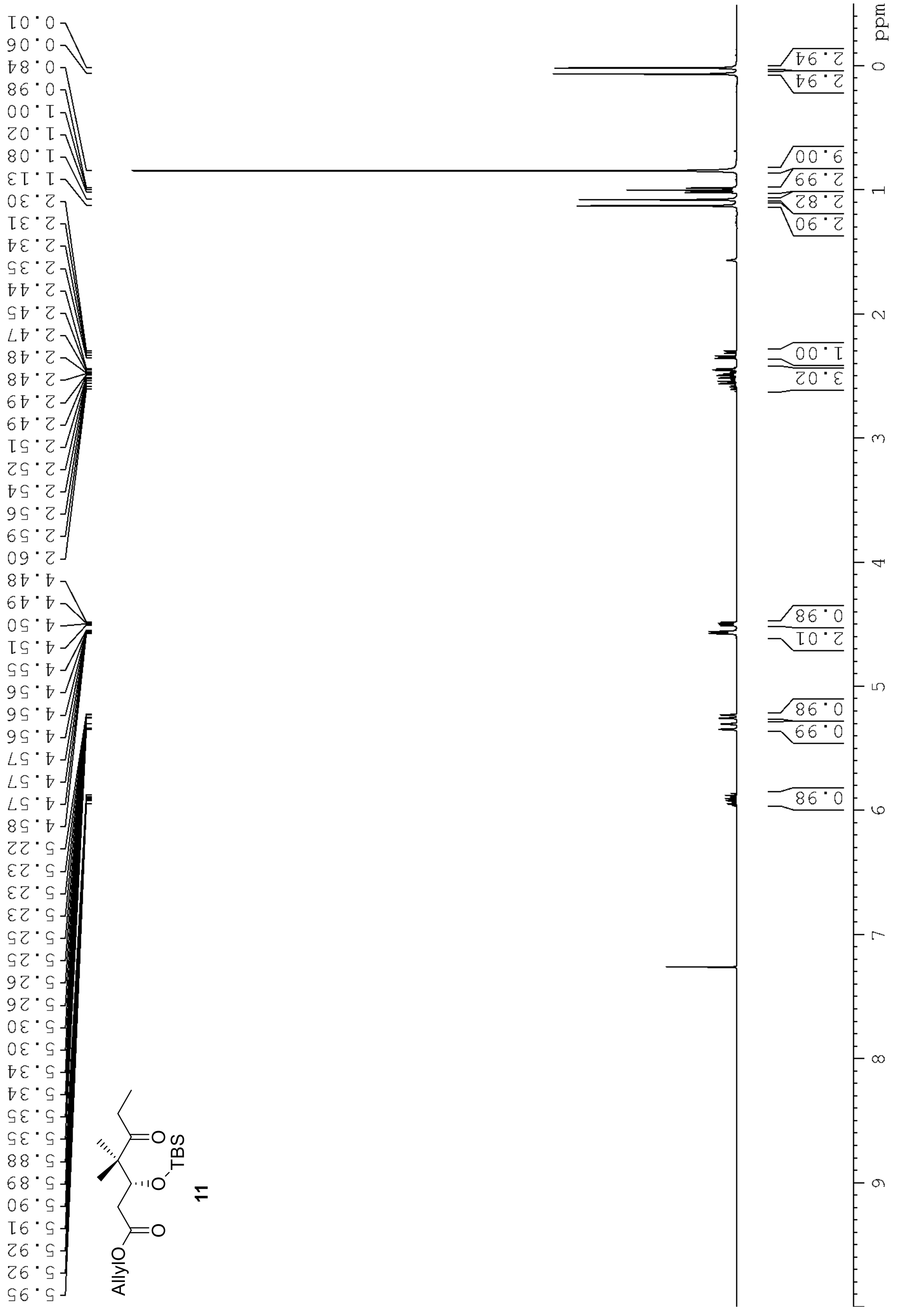

$^{13}C\{1H\}$-NMR (100 MHz, $CDCl_3$)

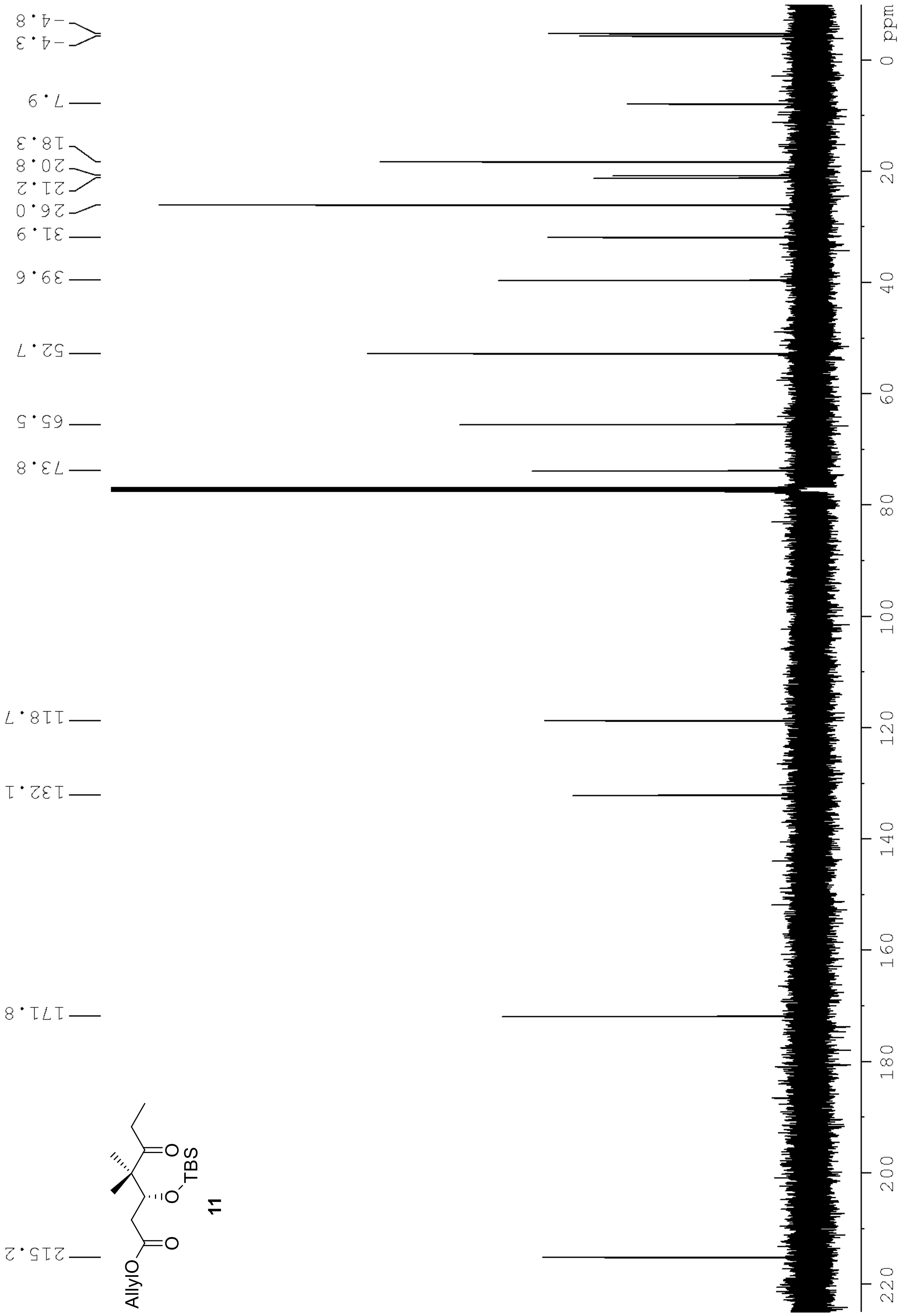

TBS-ether **67**

^{1}H-NMR (400 MHz, $CDCl_3$)

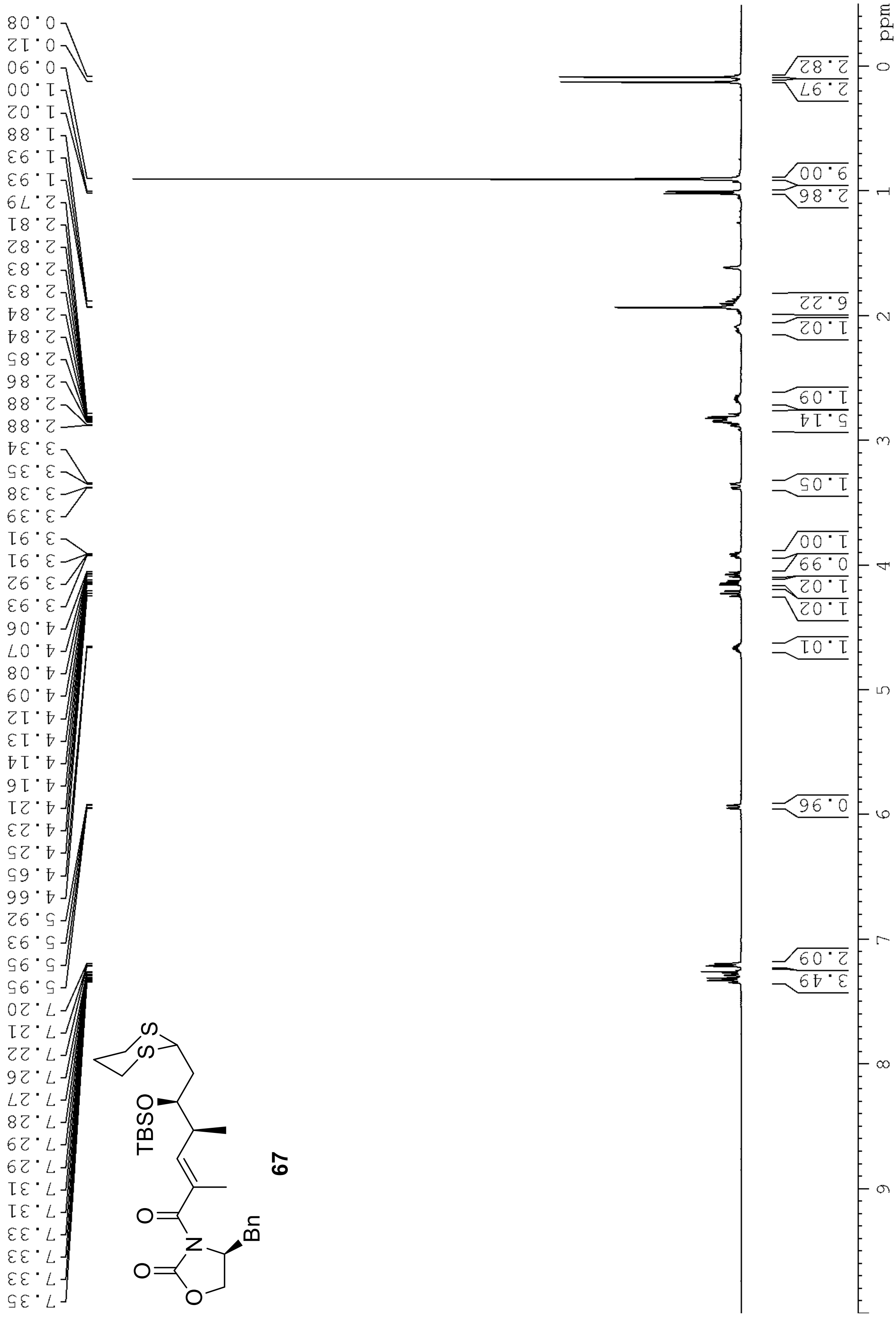

$^{13}C\{1H\}$-NMR (100 MHz, $CDCl_3$)

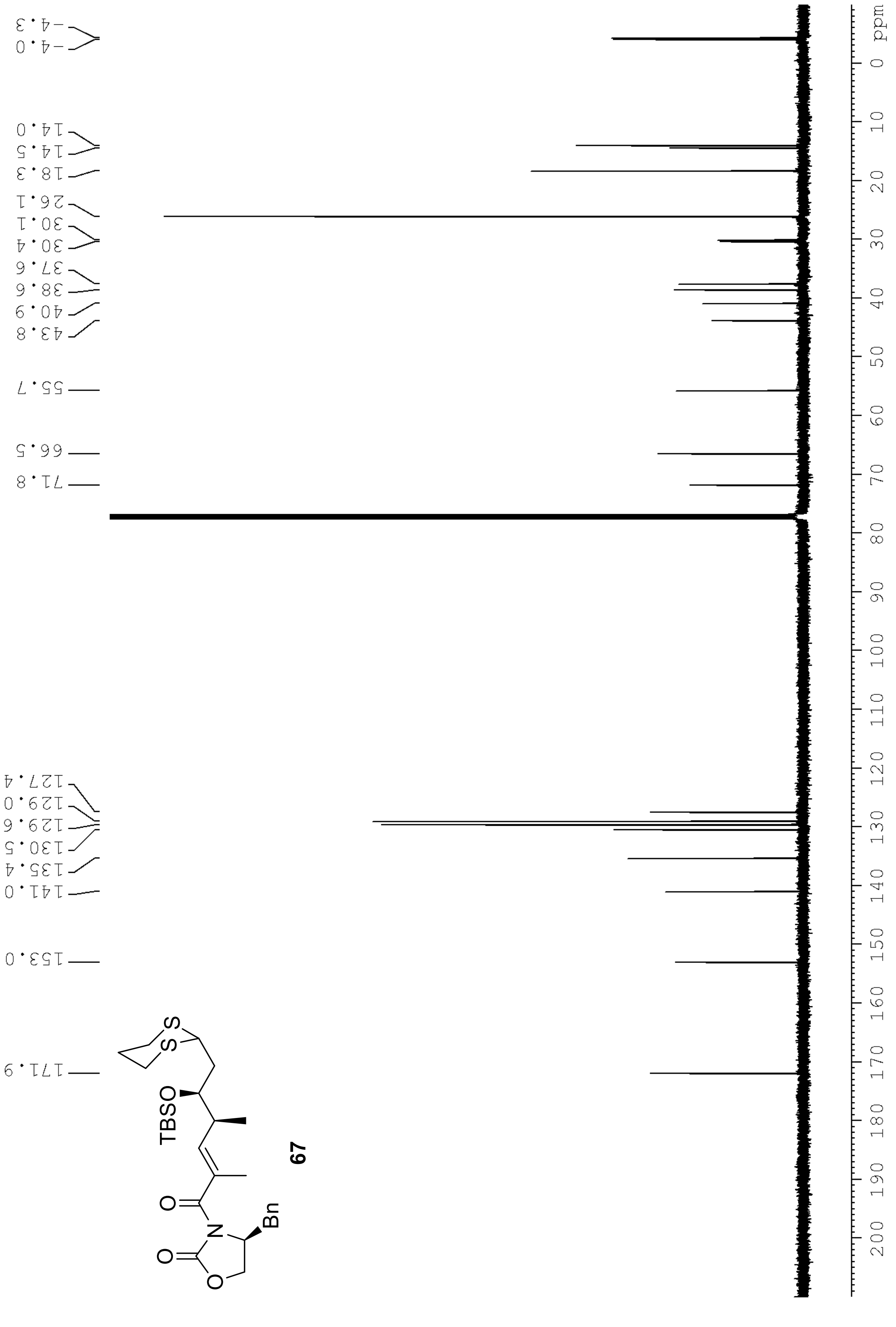

Alcohol **21**

^{1}H-NMR (400 MHz, $CDCl_3$)

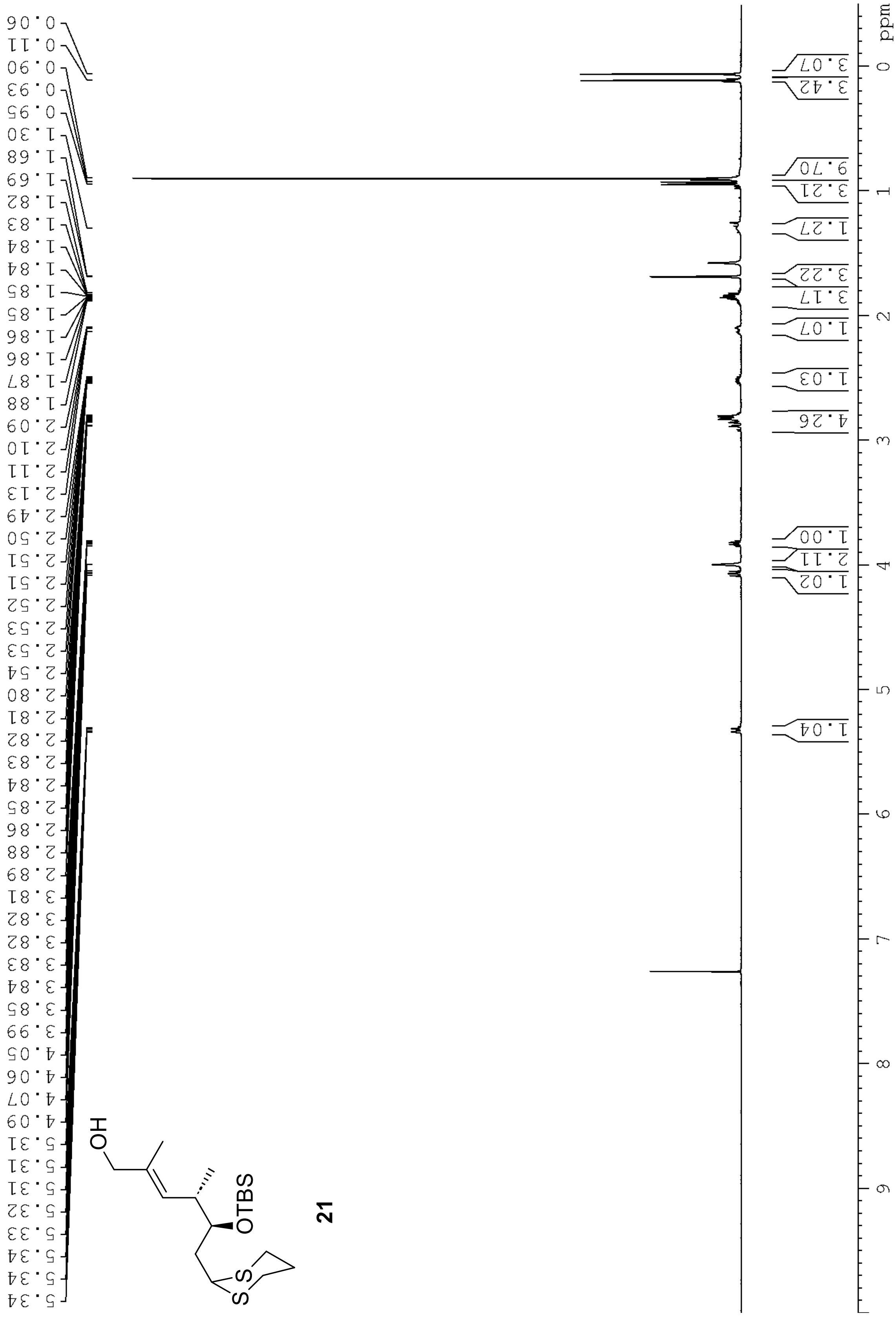

$^{13}C\{1H\}$-NMR (100 MHz, $CDCl_3$)

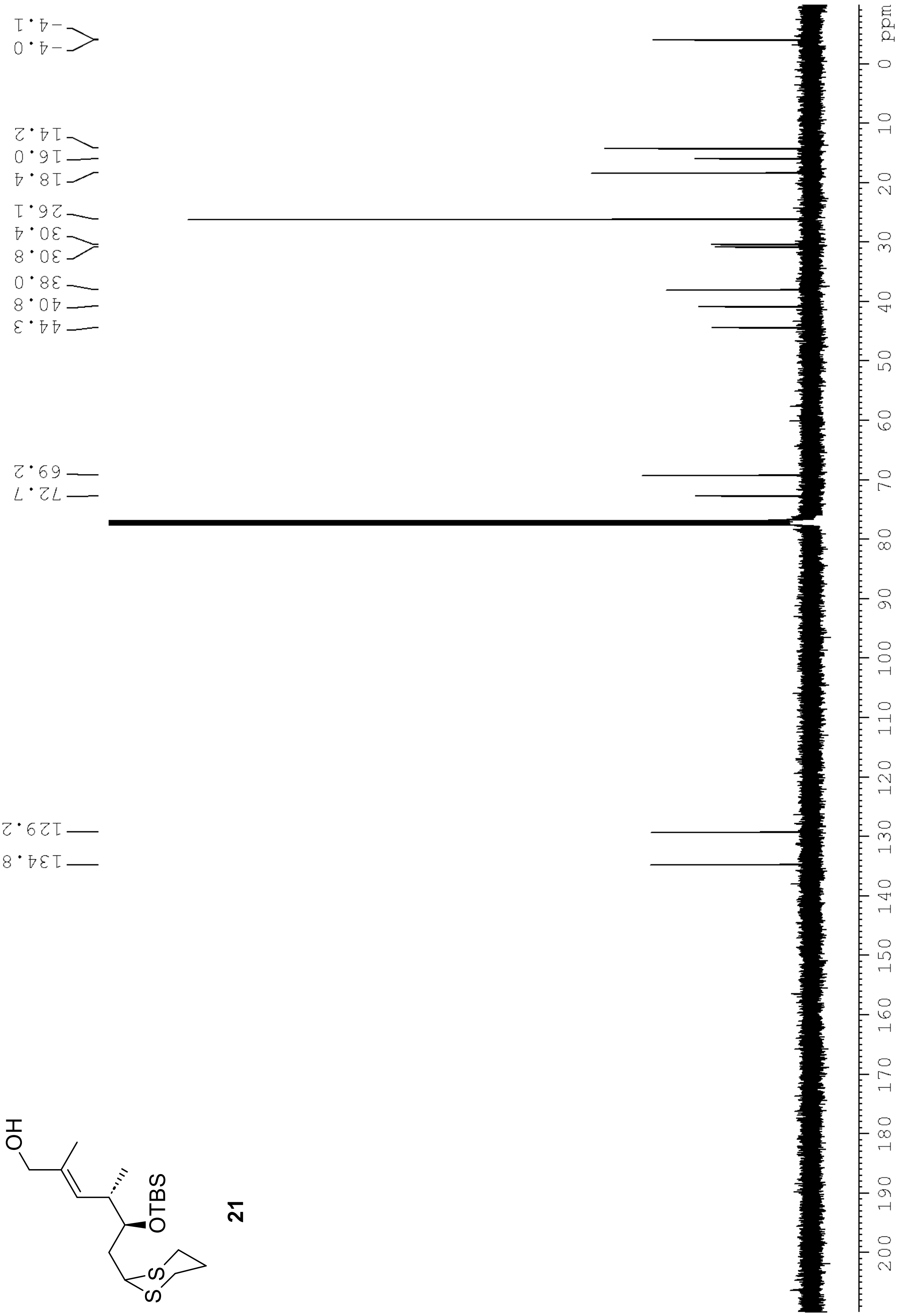

21

Pivalate **68**

^{1}H-NMR (400 MHz, $CDCl_3$)

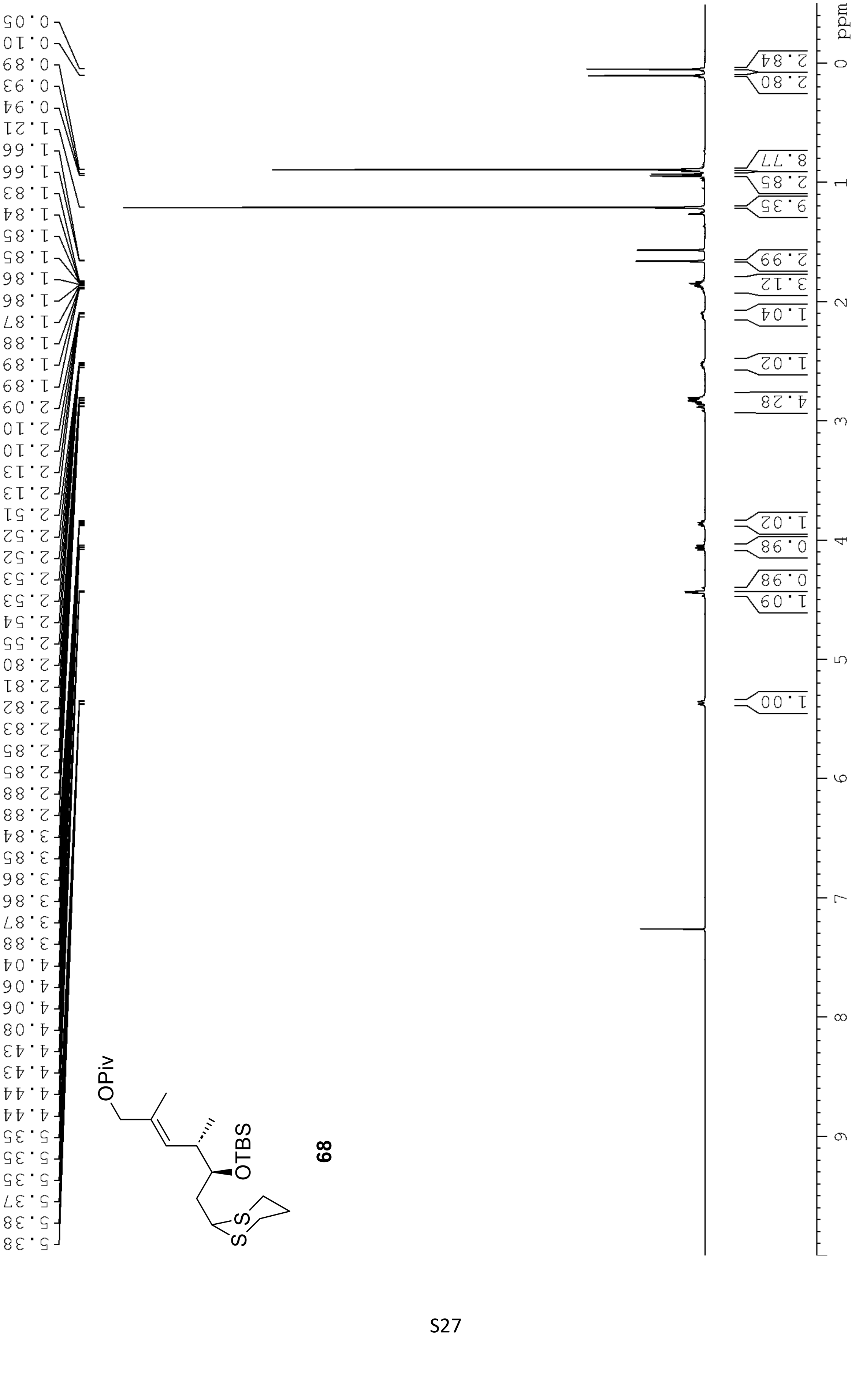

$^{13}C\{1H\}$-NMR (100 MHz, $CDCl_3$)

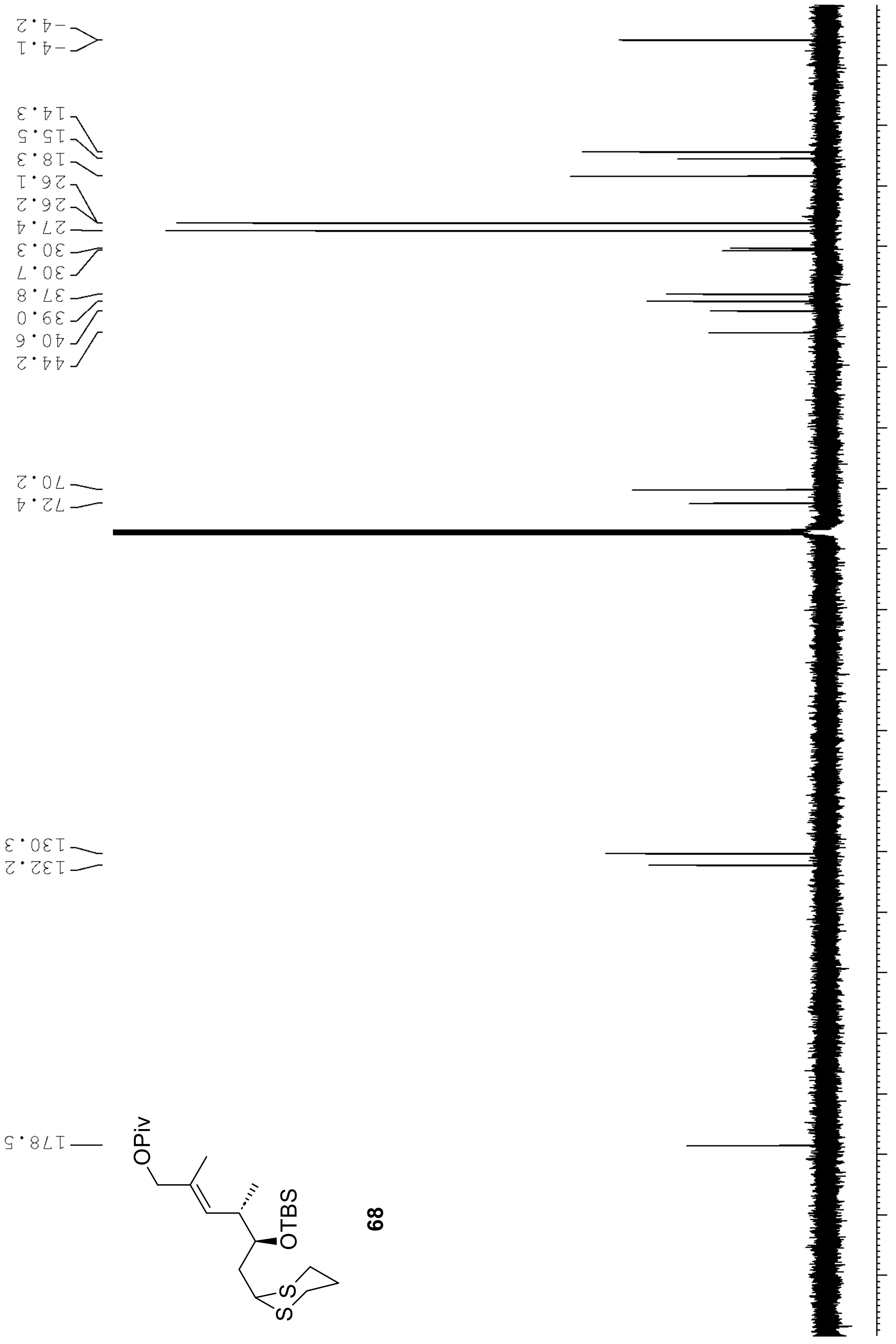

68

(*S*)-Mosher ester **S1**

^{1}H-NMR (400 MHz, C_6D_6)

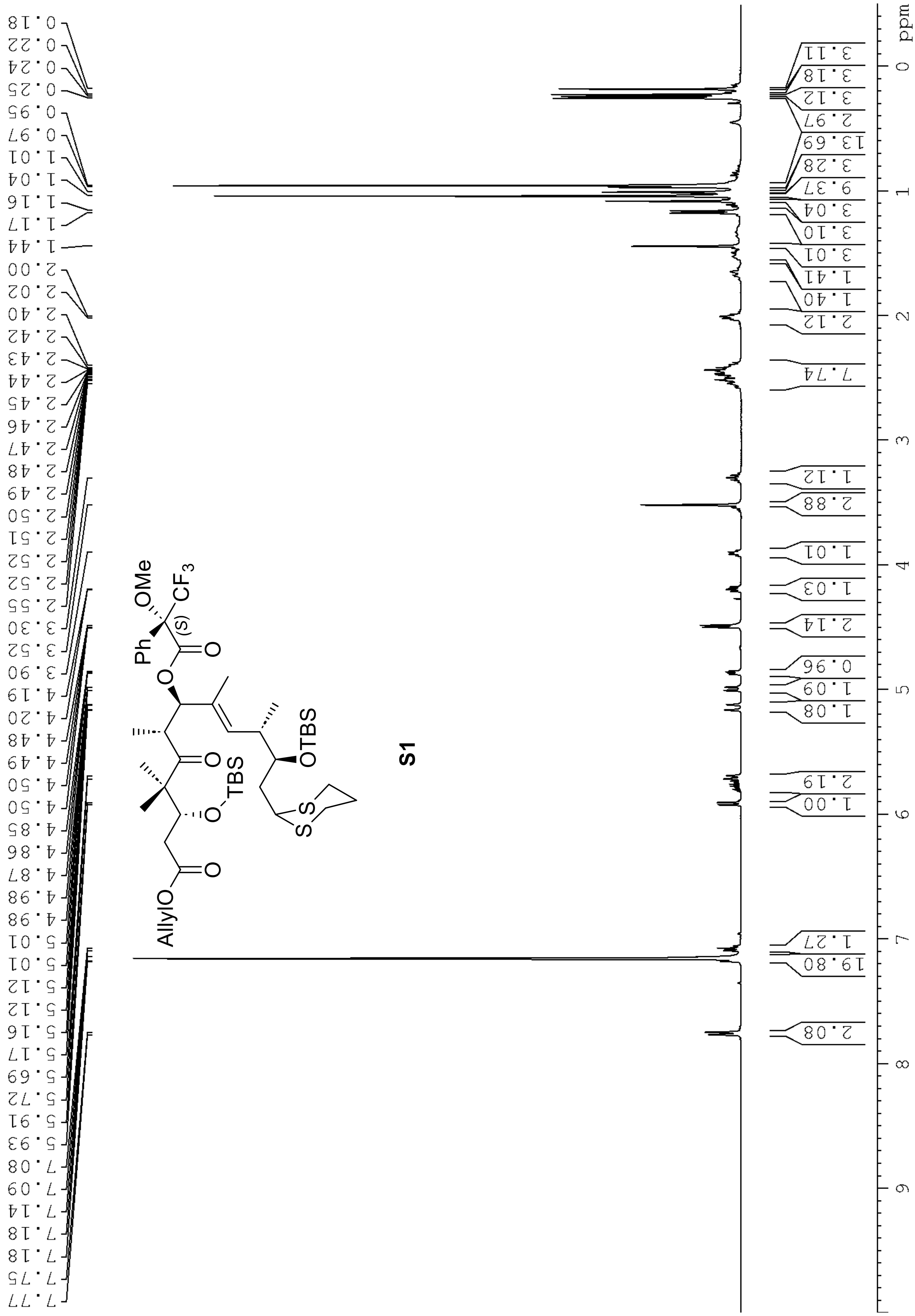

(*R*)-Mosher ester **S2**

^{1}H-NMR (400 MHz, C_6D_6)

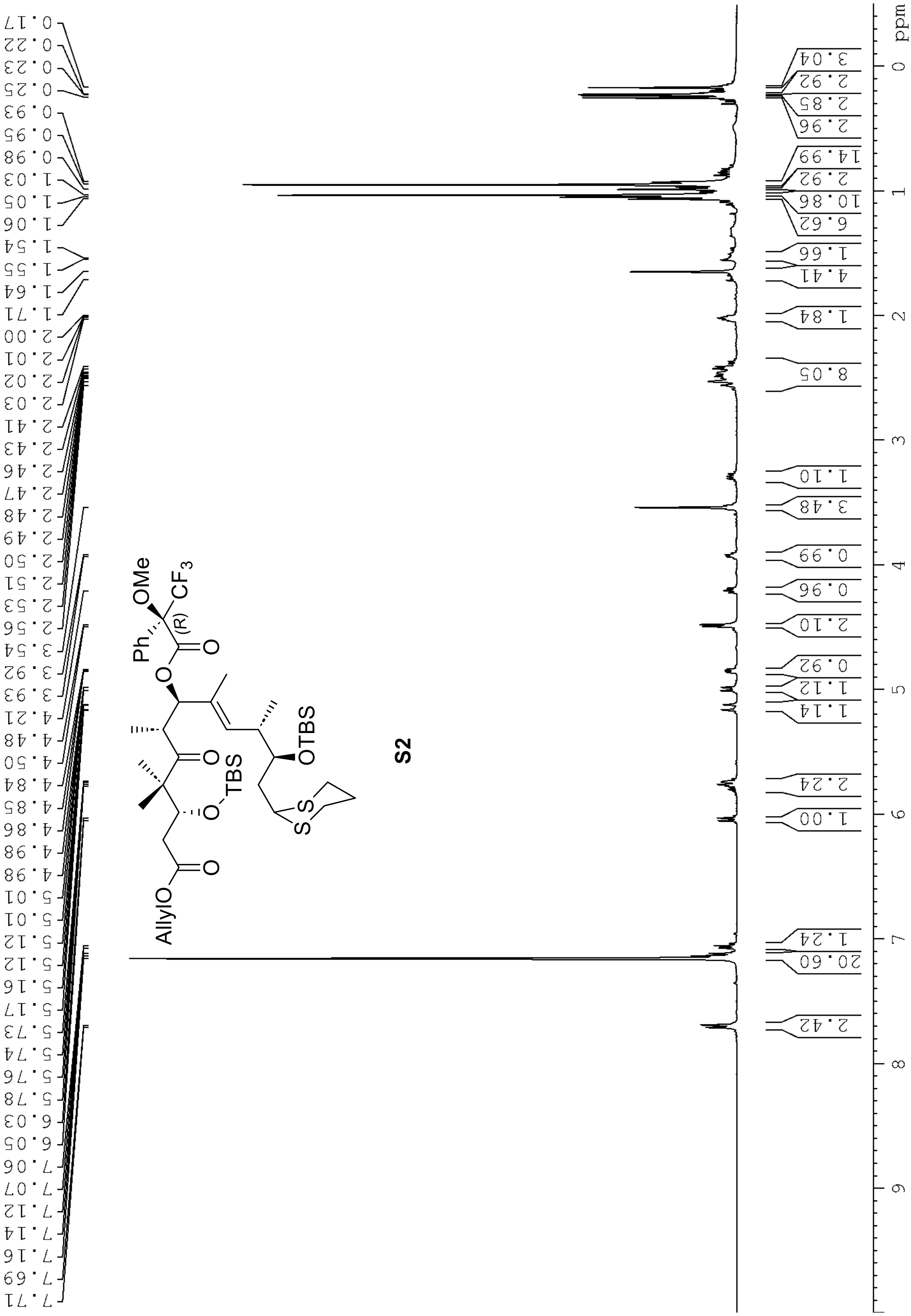

(*S*)-Mosher ester **S3**

^{1}H-NMR (400 MHz, C_6D_6)

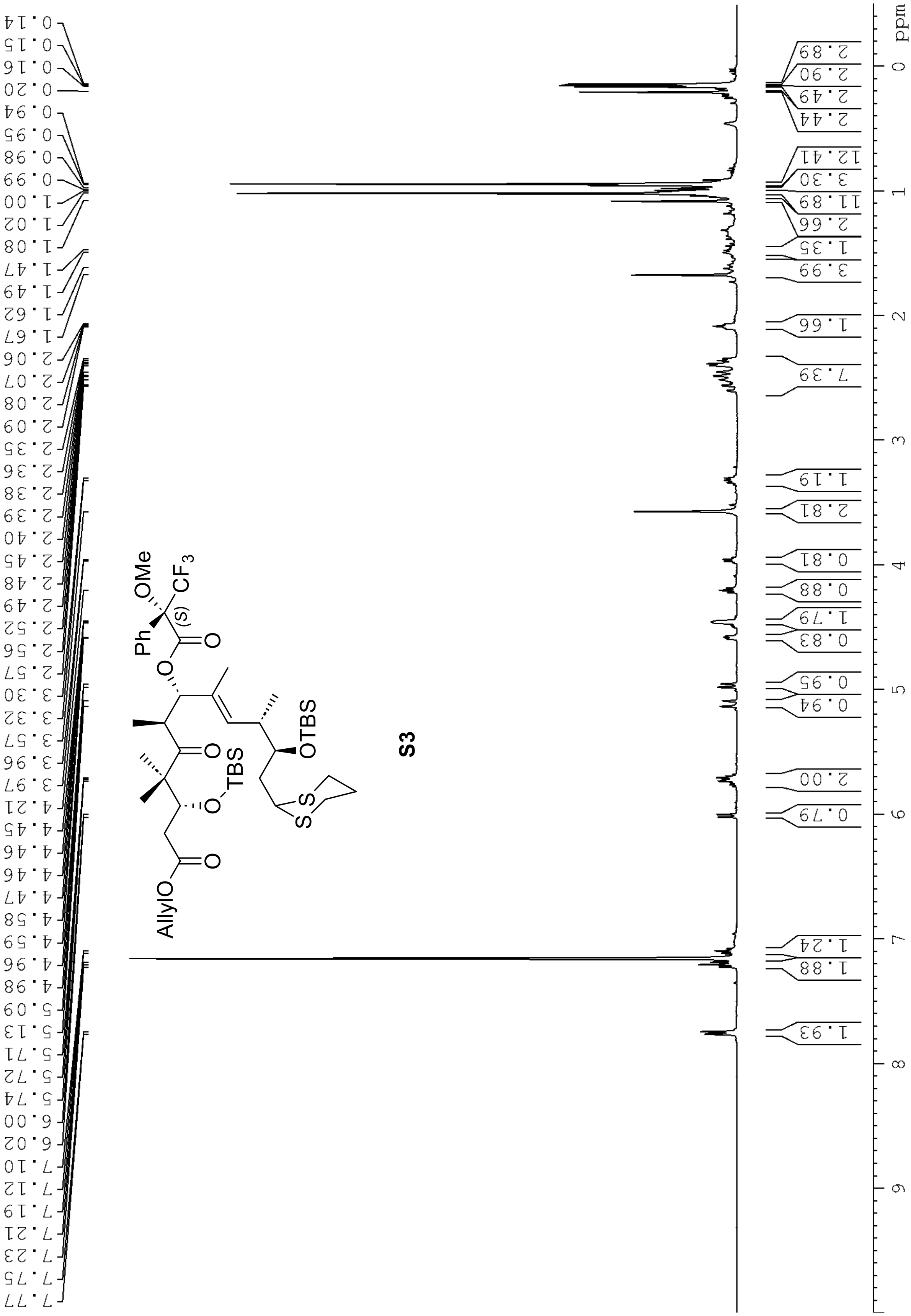

S3

(*R*)-Mosher ester **S4**

^{1}H-NMR (400 MHz, C_6D_6)

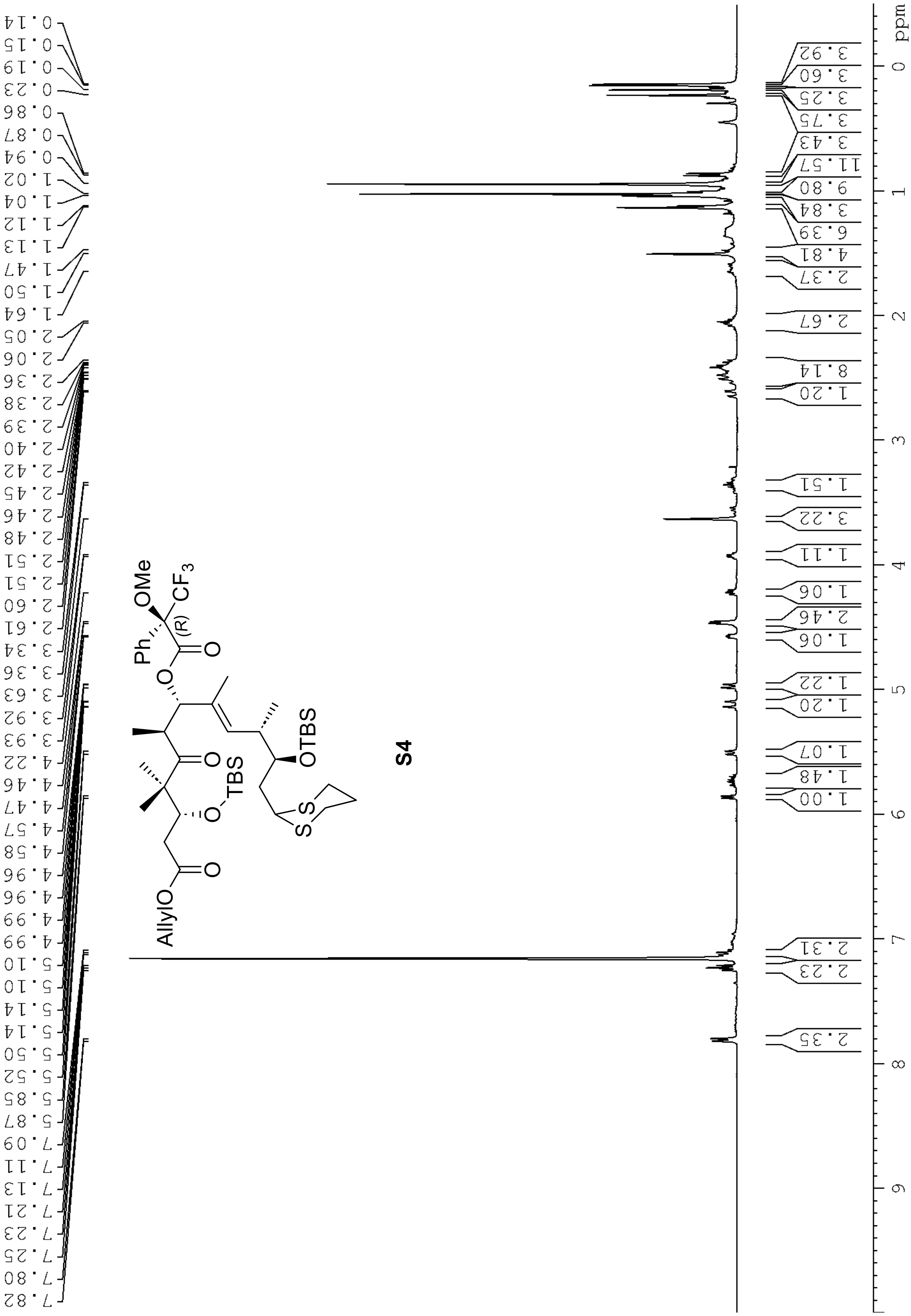

TBS-ether **69a**

^{1}H-NMR (400 MHz, $CDCl_3$)

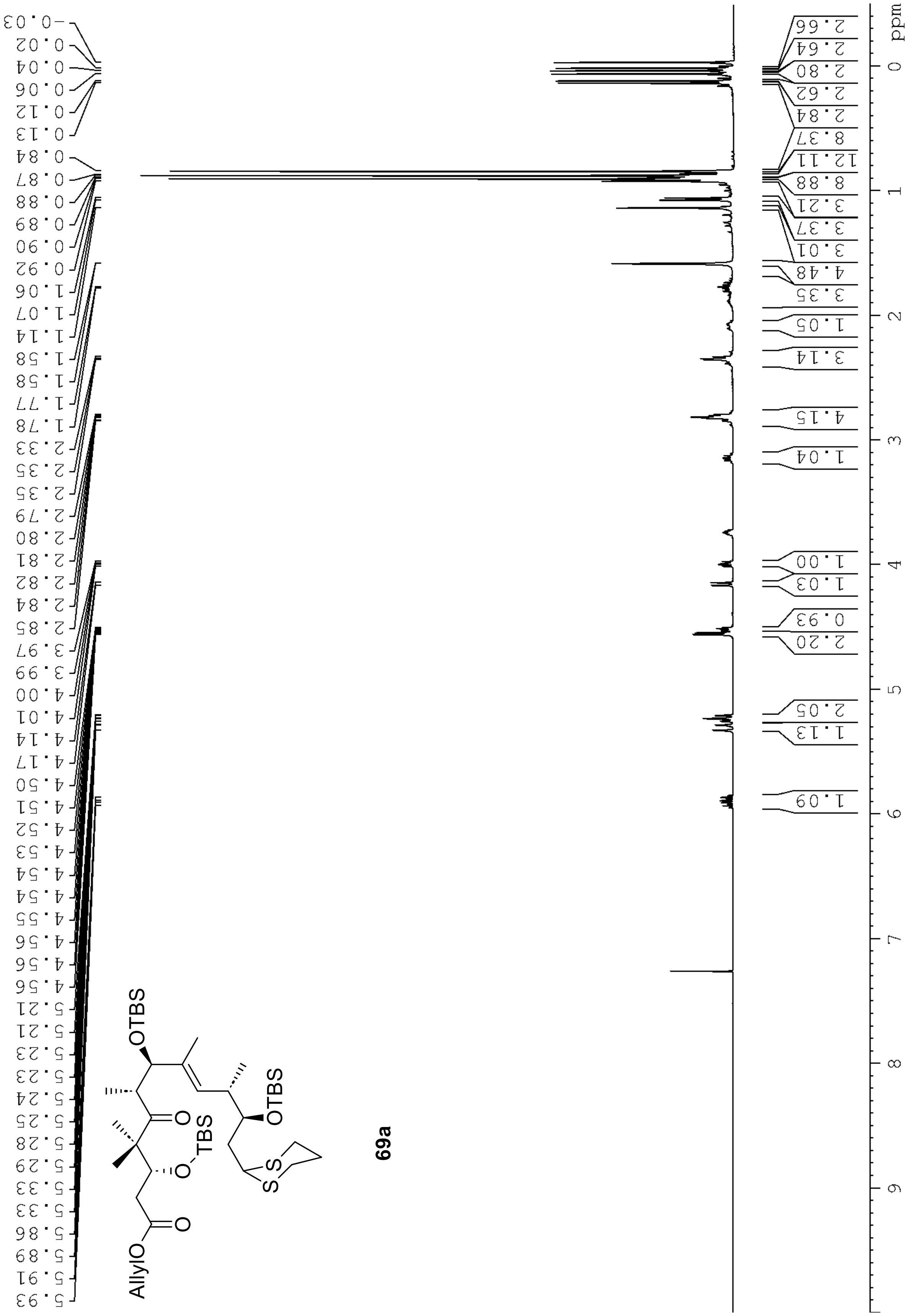

$^{13}C\{1H\}$-NMR (100 MHz, $CDCl_3$)

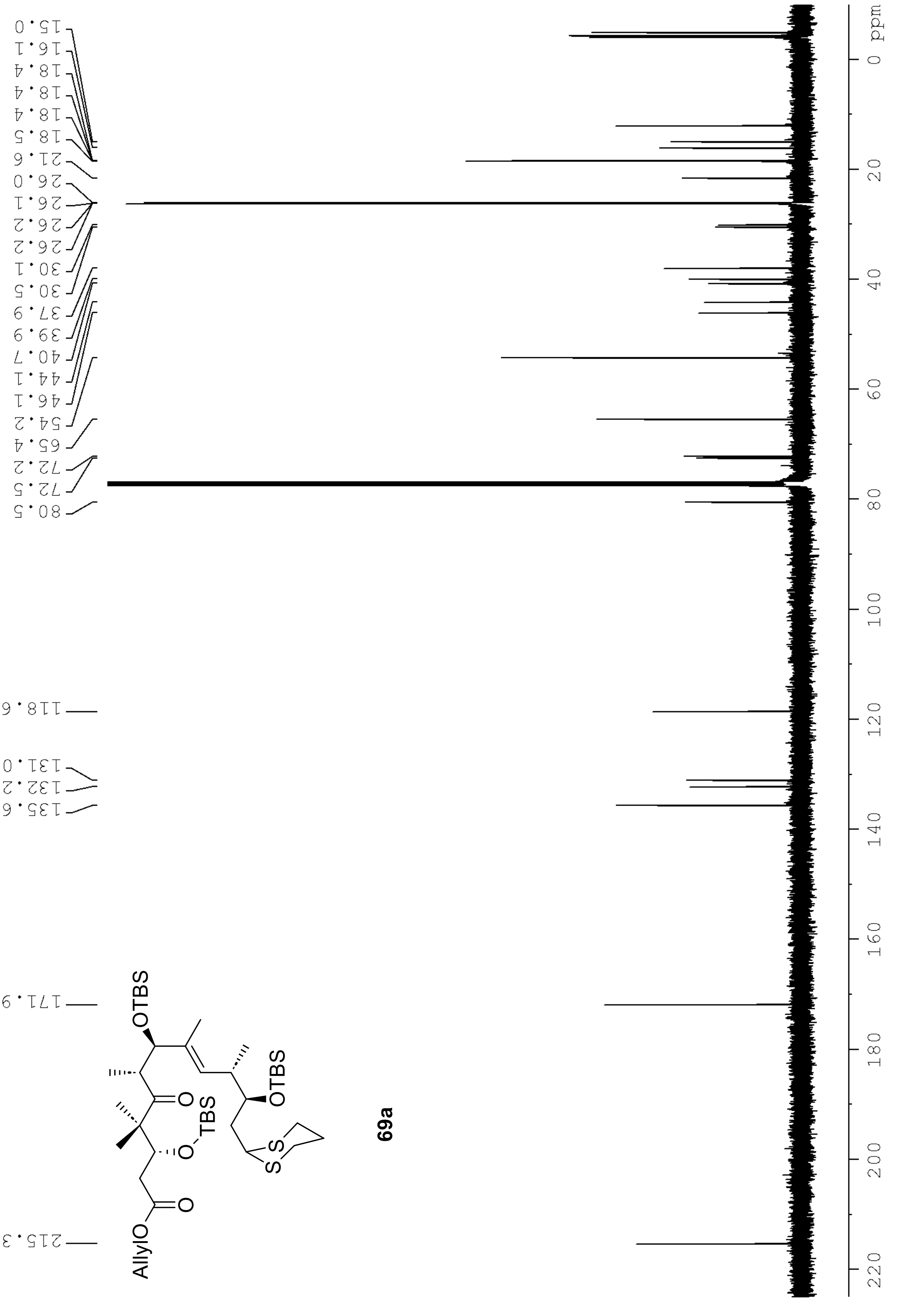

TBS-ether **69b**

^{1}H-NMR (400 MHz, $CDCl_3$)

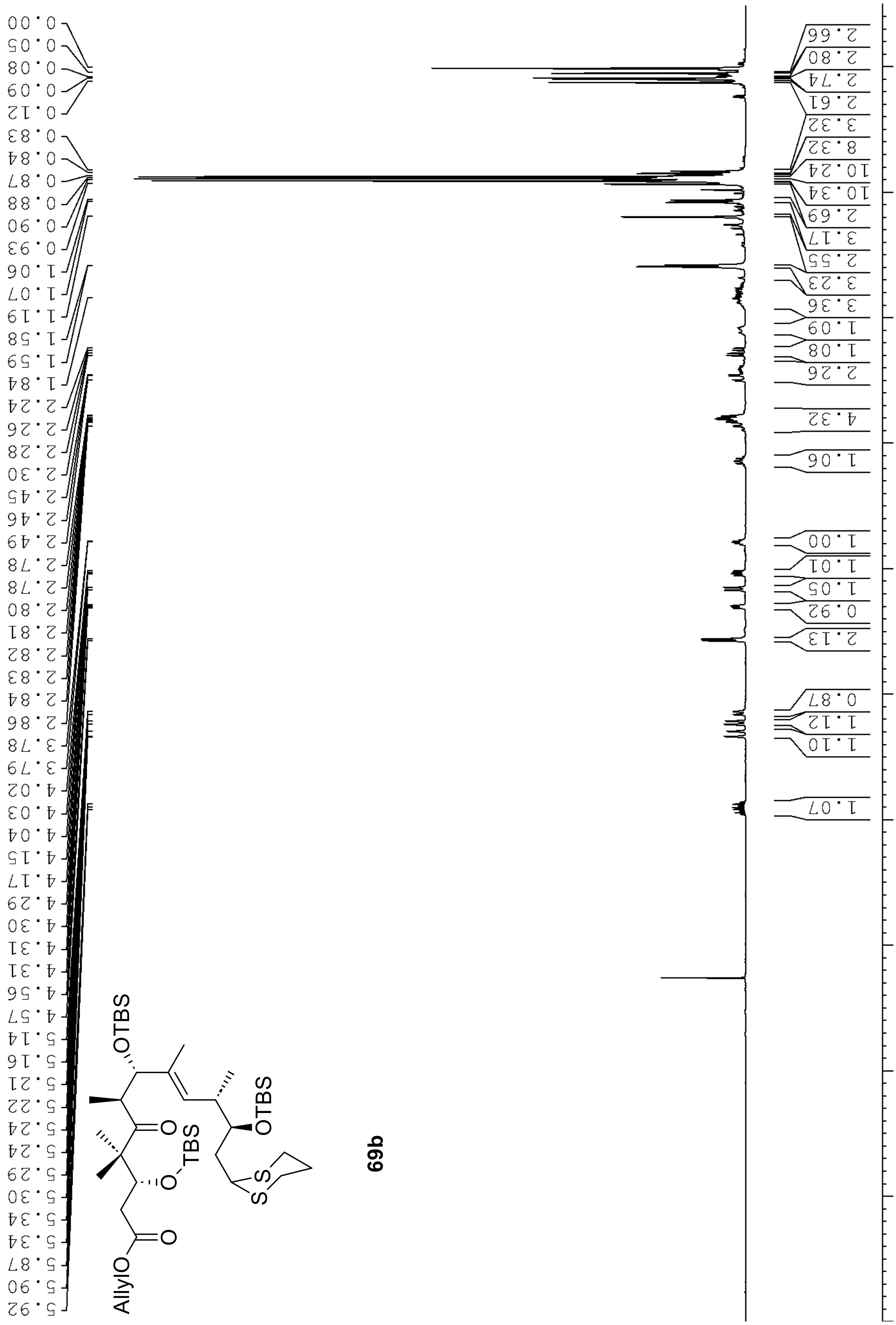

$^{13}C\{1H\}$-NMR (100 MHz, $CDCl_3$)

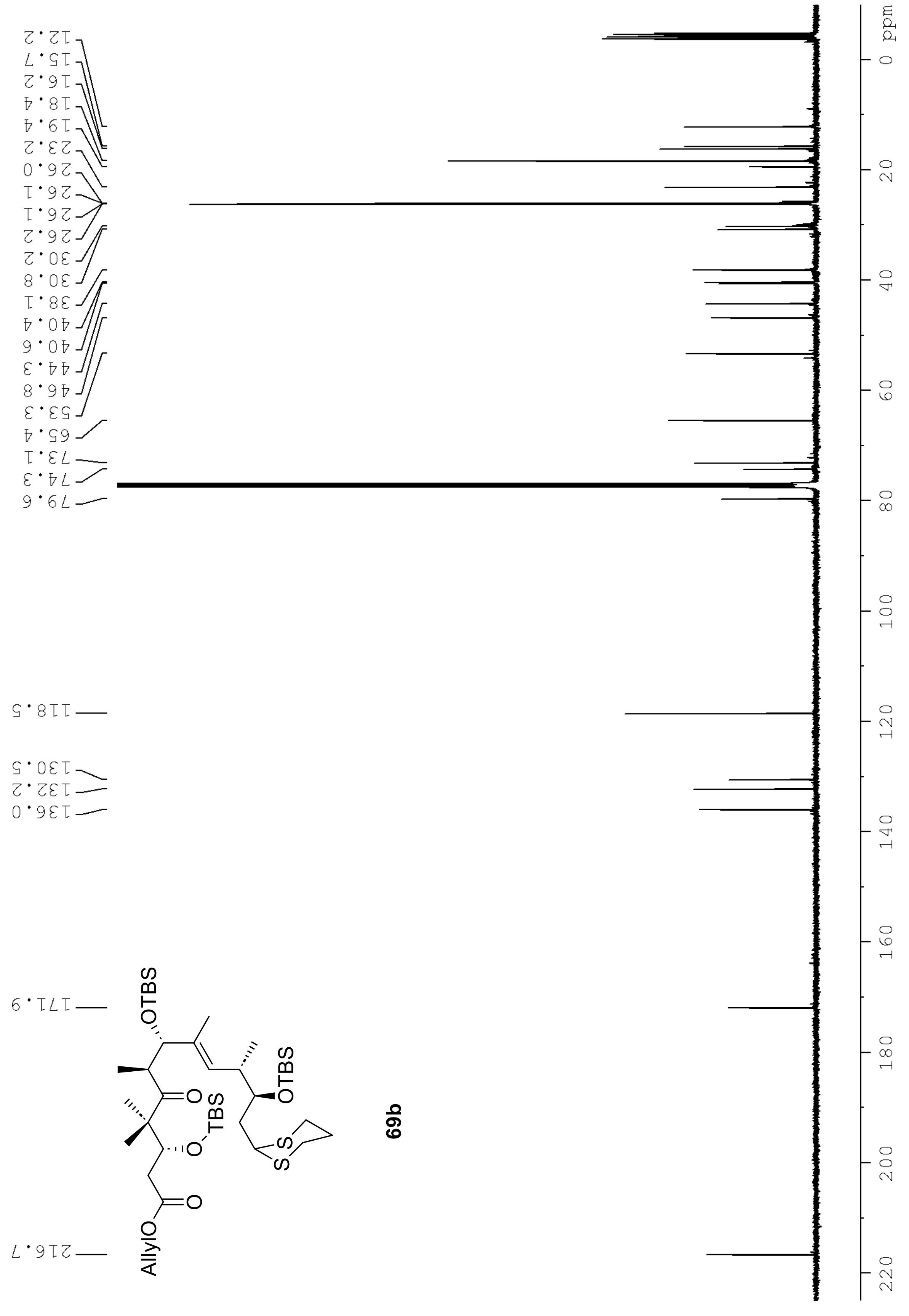

Aldehyde **28**

^{1}H-NMR (400 MHz, $CDCl_3$)

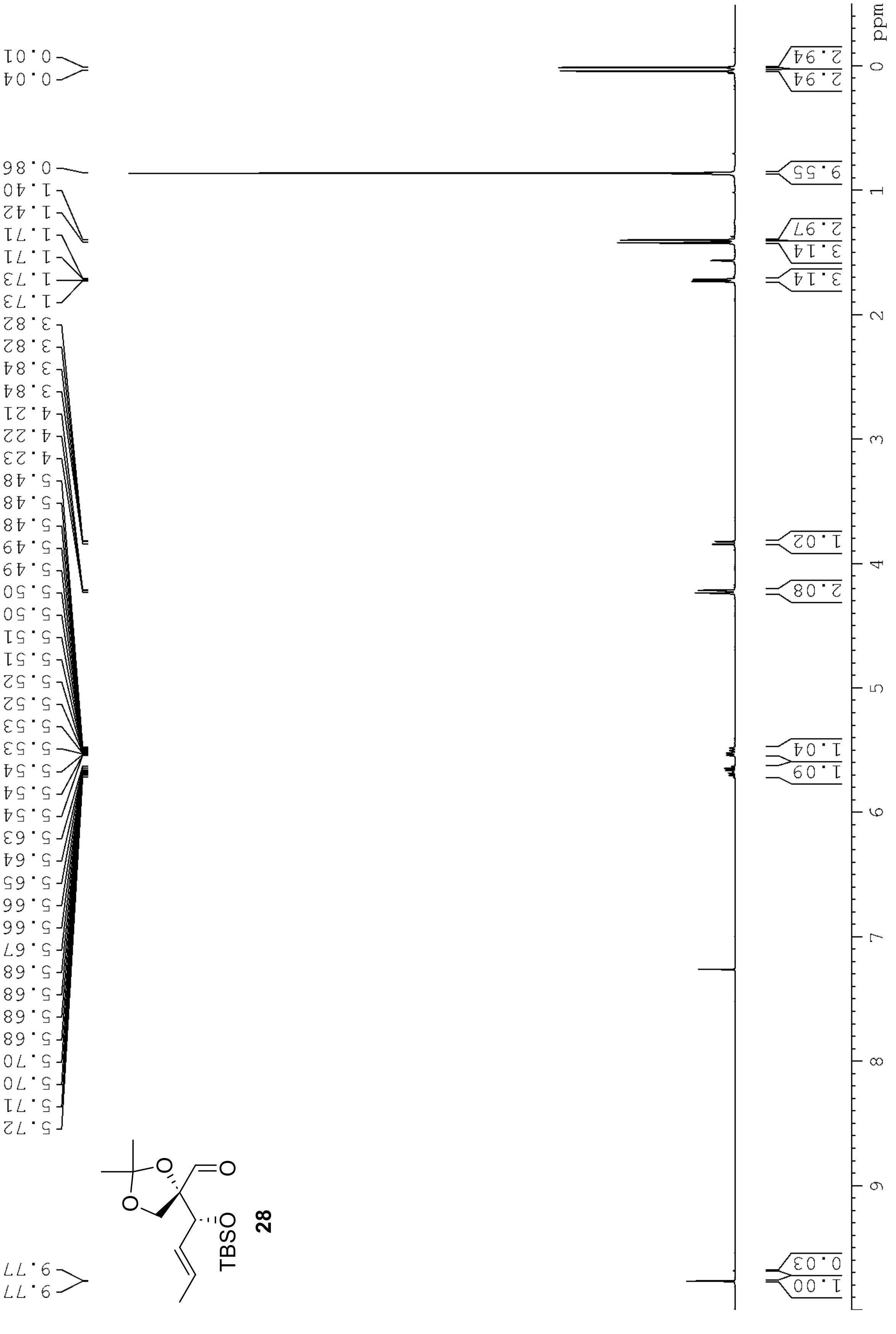

$^{13}C\{1H\}$-NMR (100 MHz, $CDCl_3$)

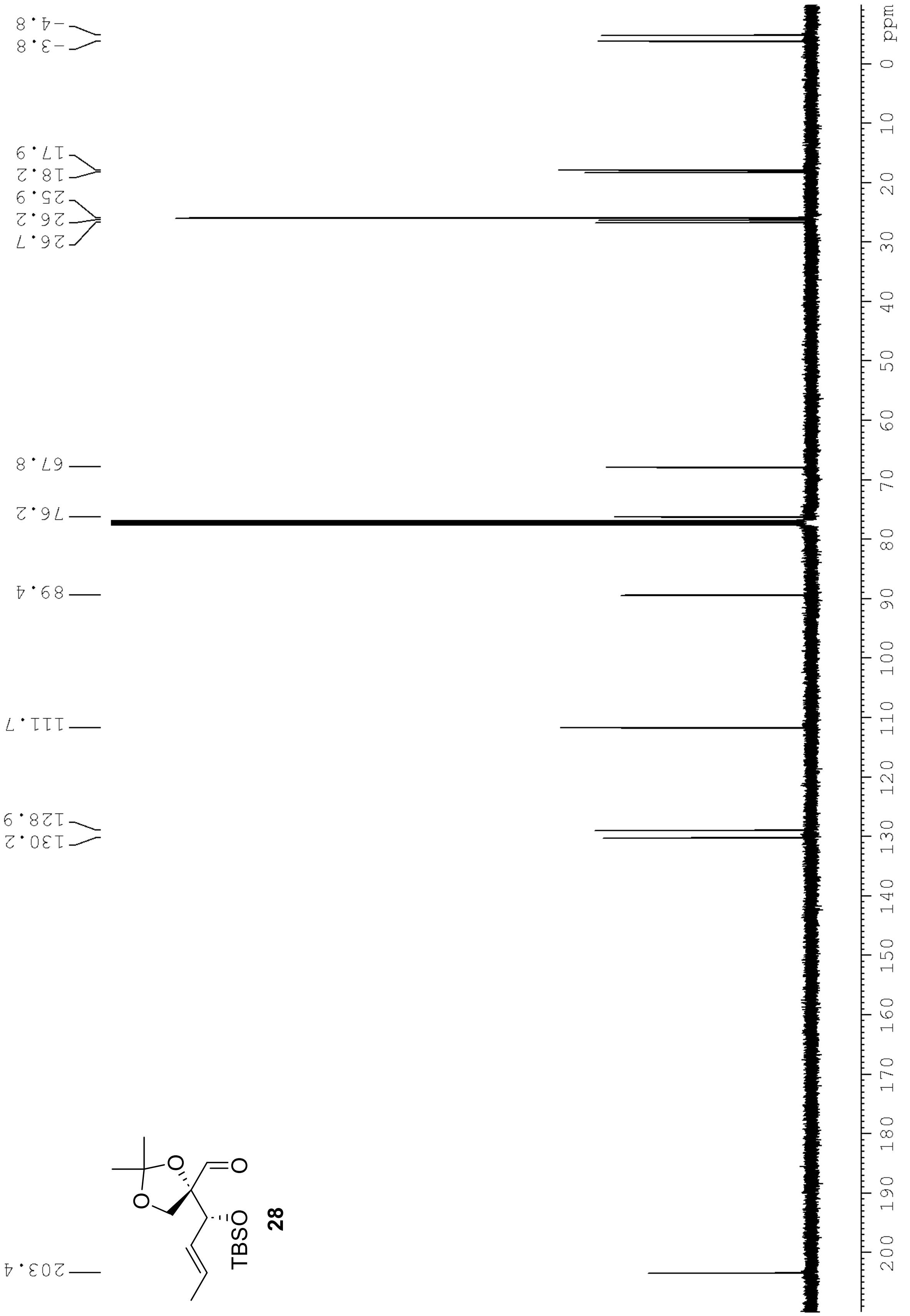

Ketone **25**

^{1}H-NMR (400 MHz, $CDCl_3$)

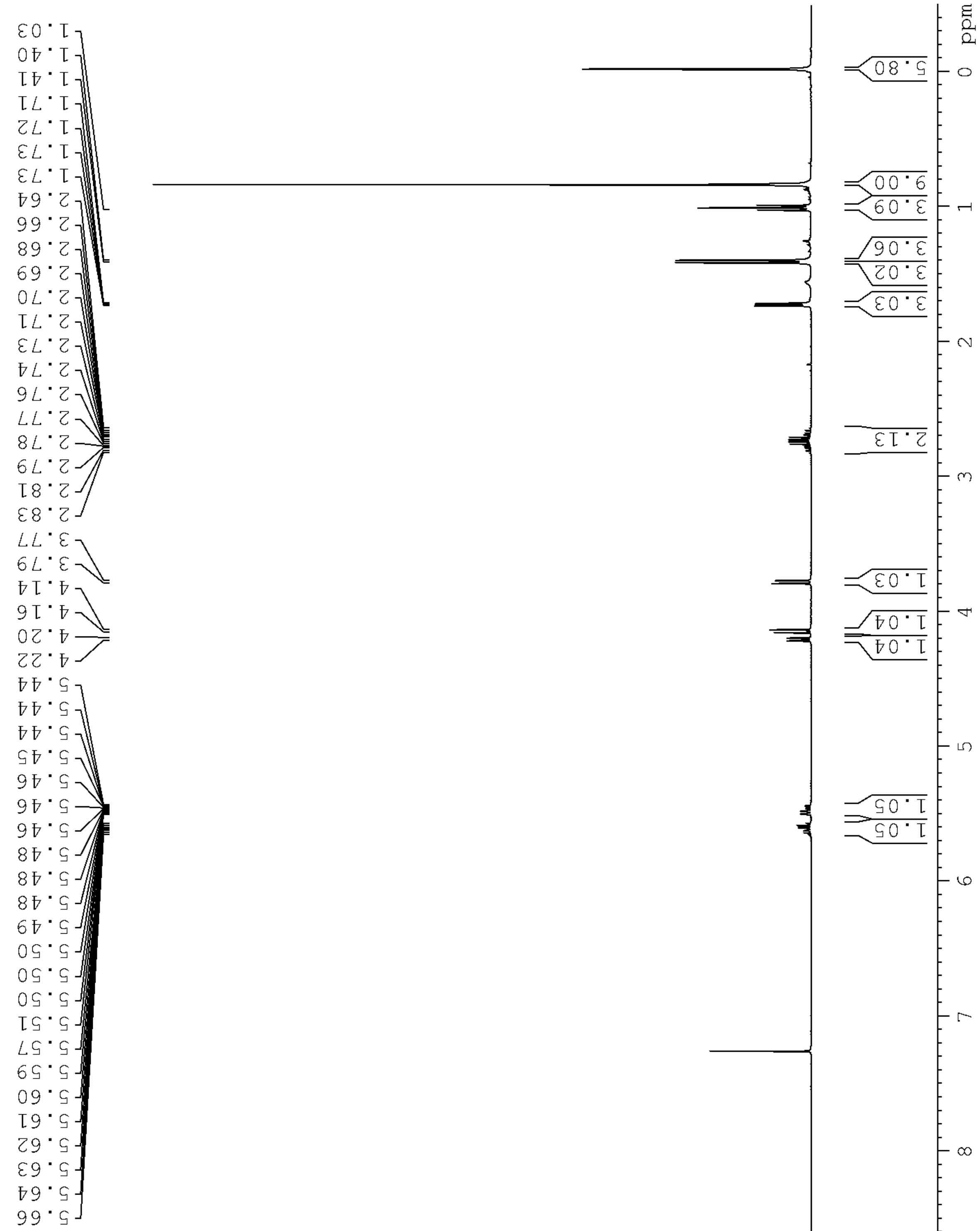

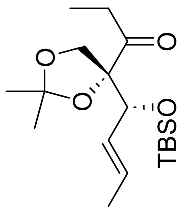

25

$^{13}C\{1H\}$-NMR (100 MHz, $CDCl_3$)

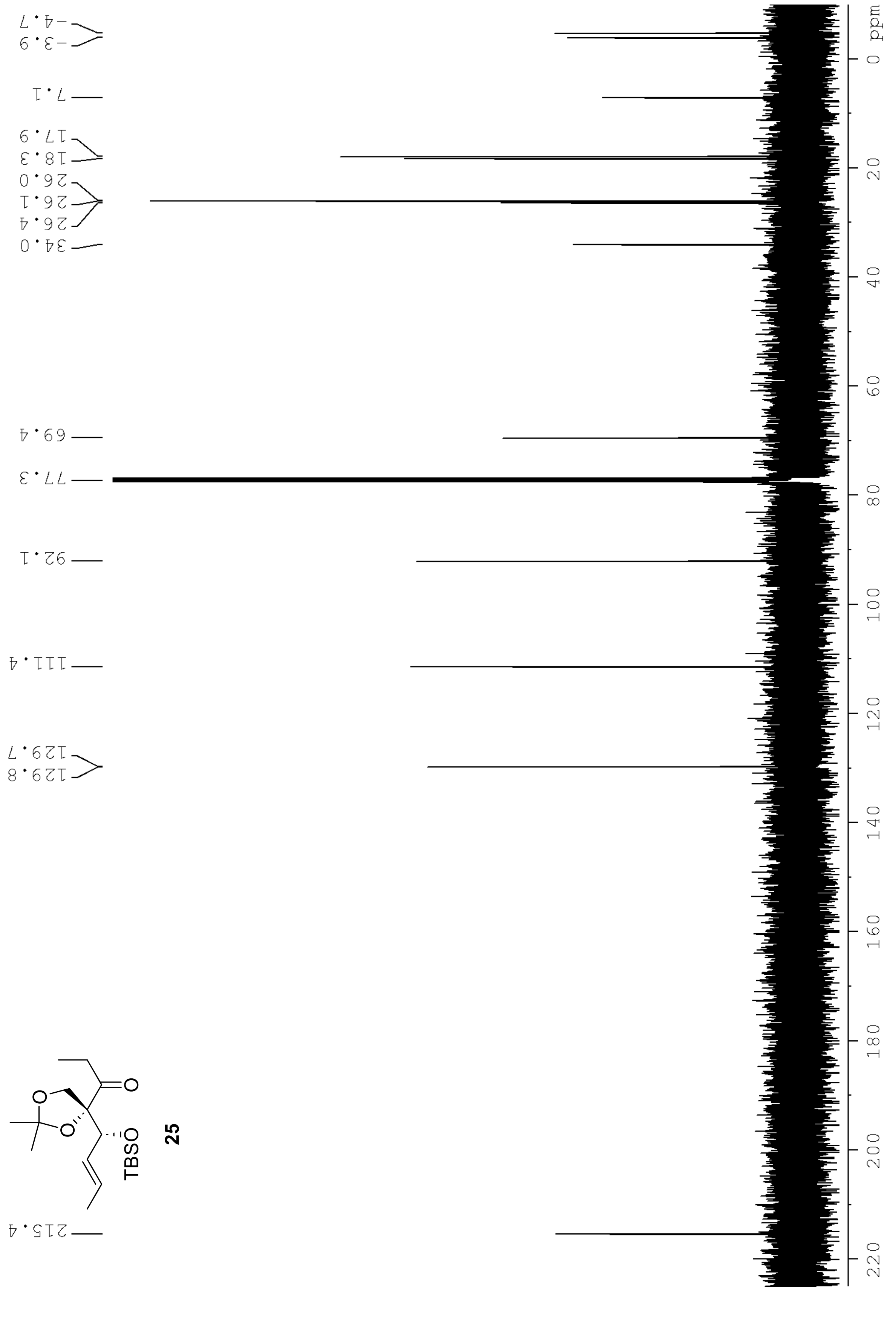

25

Ketone **30**

^{1}H-NMR (400 MHz, $CDCl_3$)

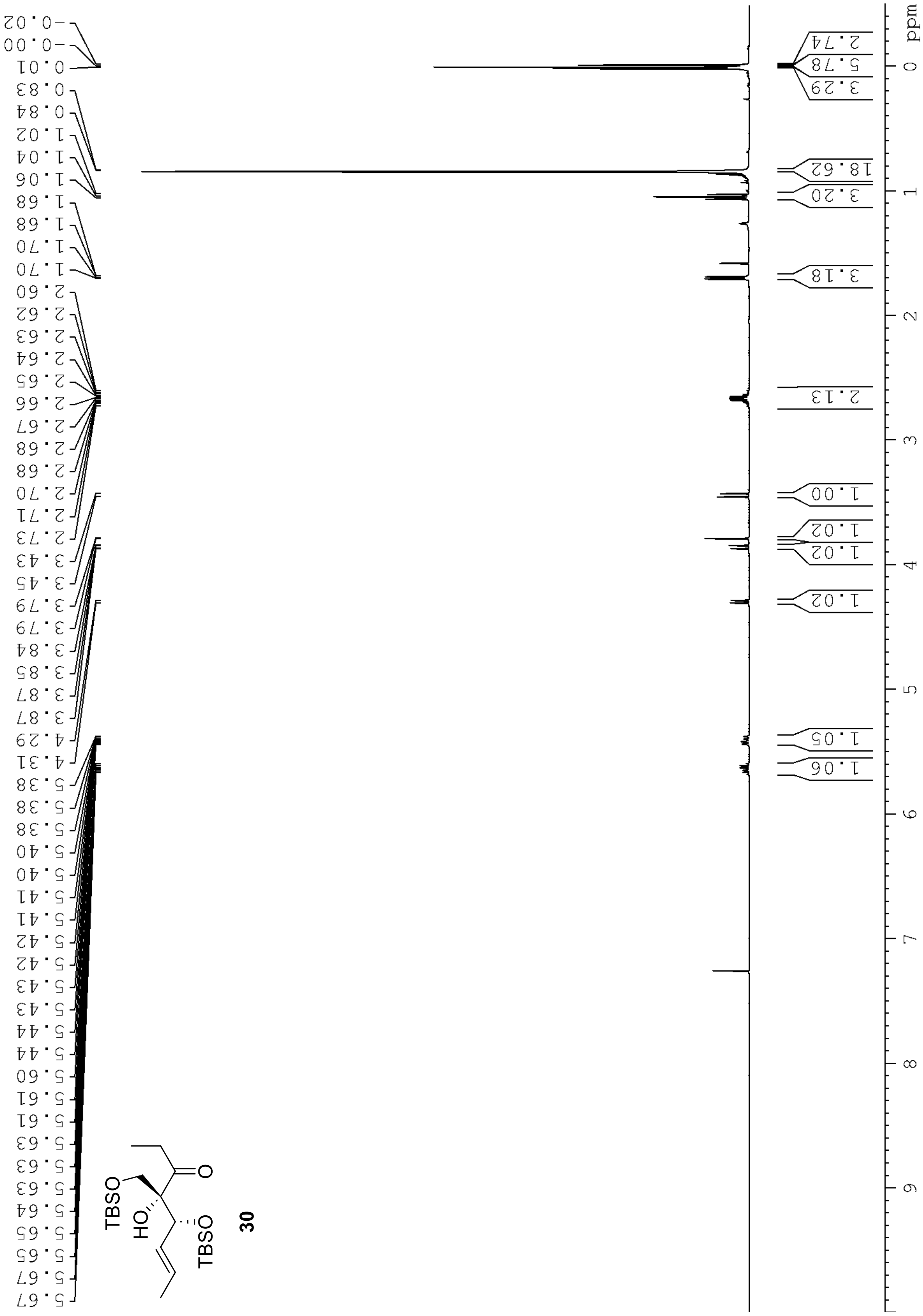

^{13}C{1H}-NMR (100 MHz, $CDCl_3$)

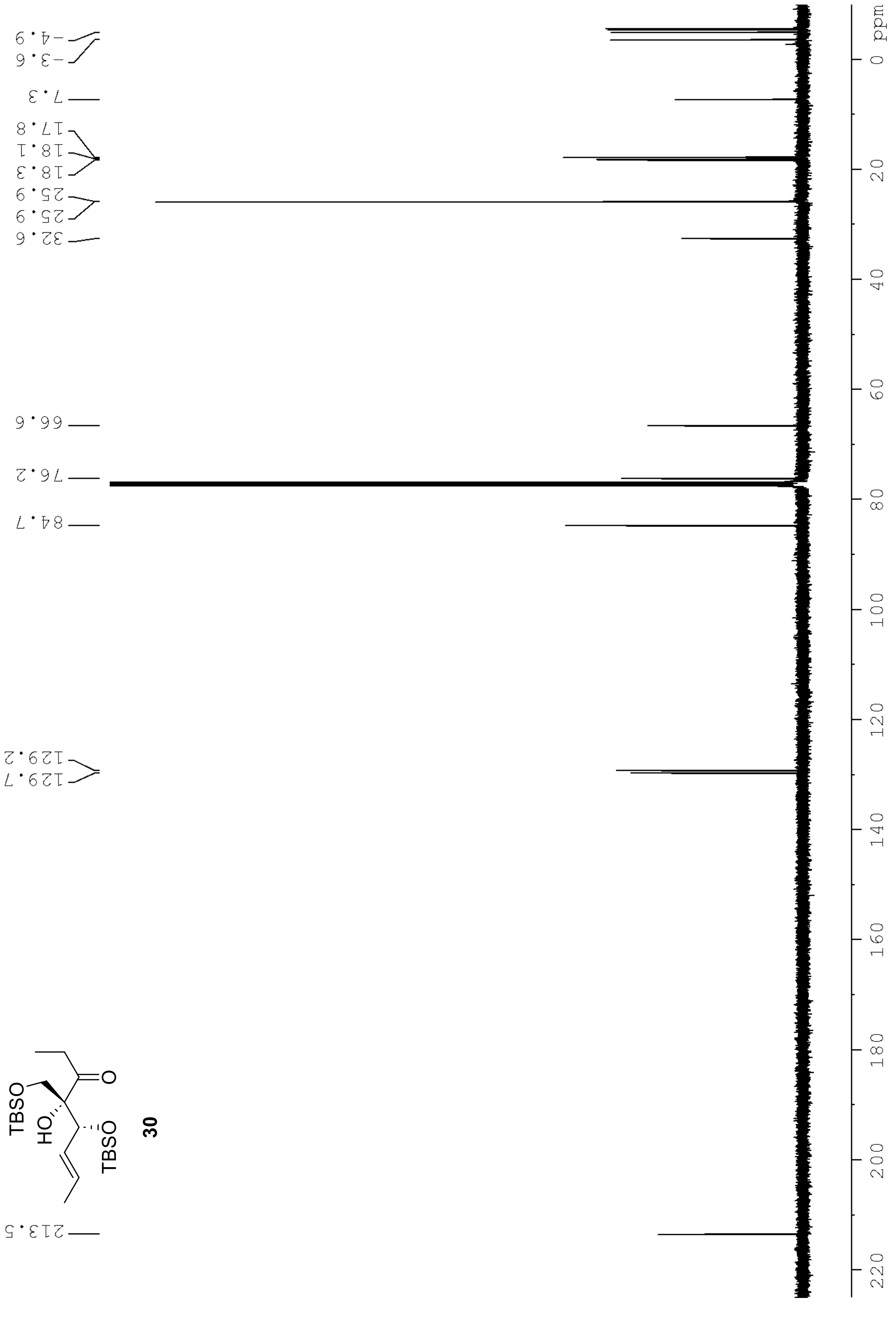

Ketone **31**

^{1}H-NMR (400 MHz, $CDCl_3$)

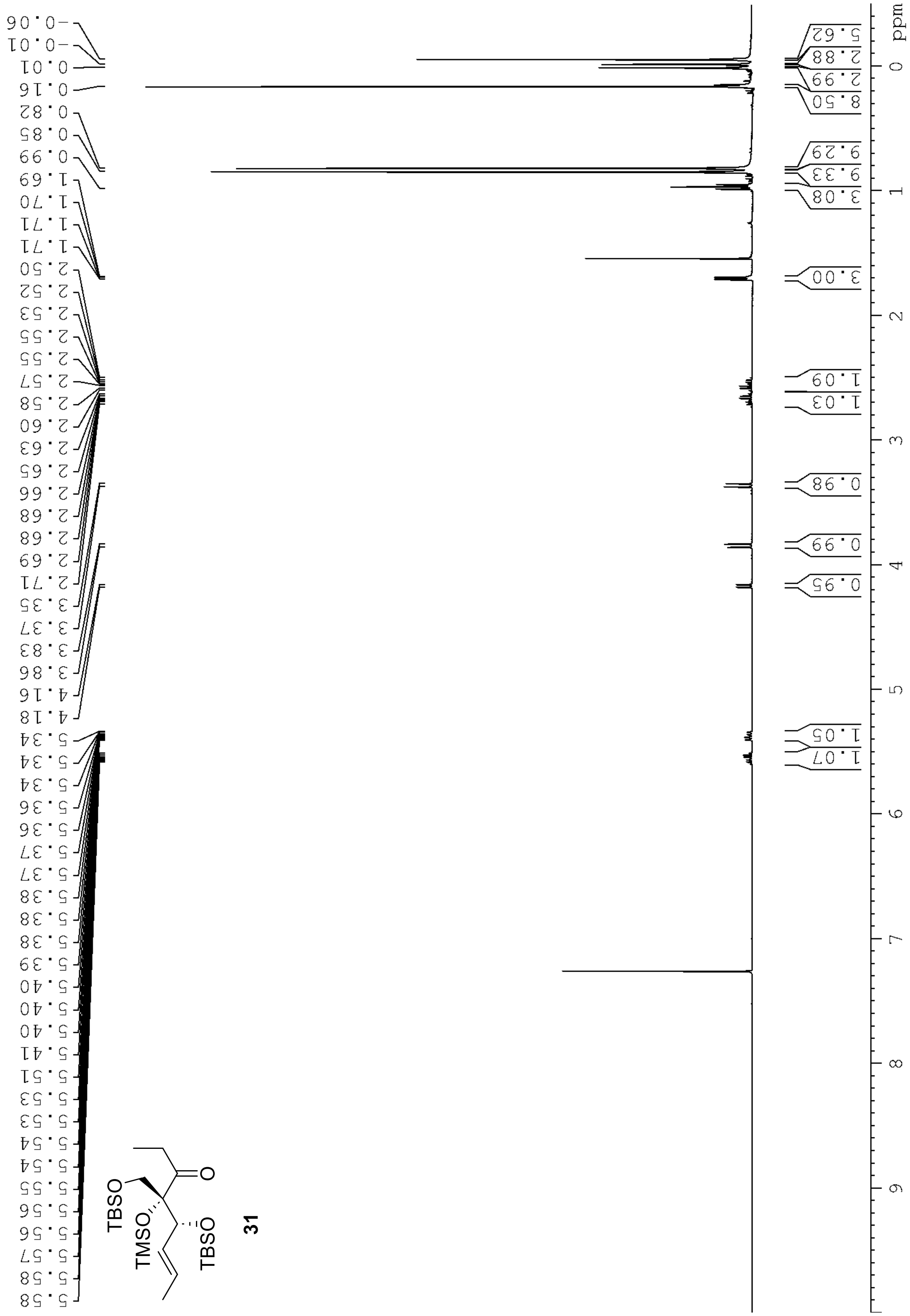

$^{13}C\{1H\}$-NMR (100 MHz, $CDCl_3$)

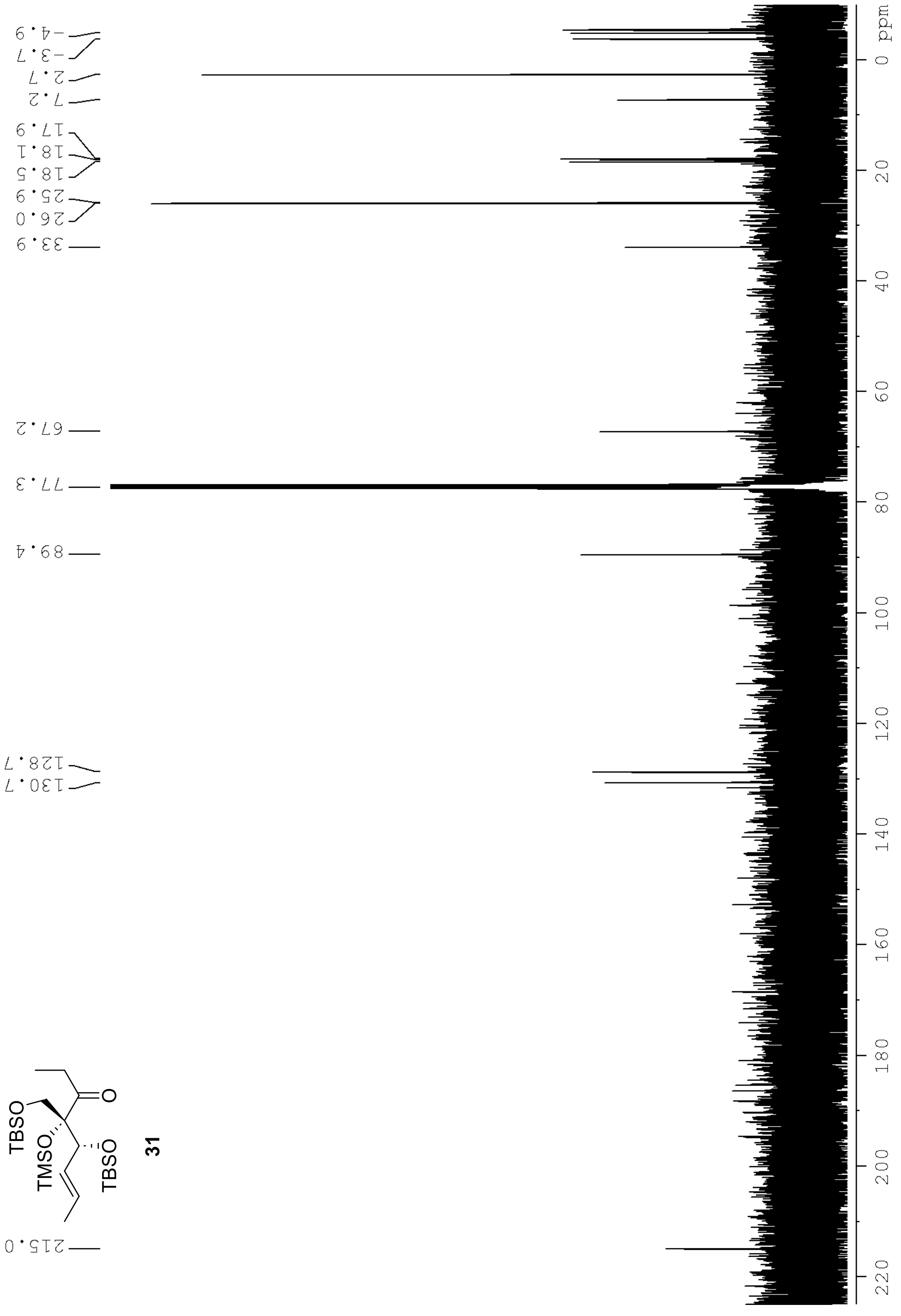

TBS-ether **73**

^{1}H-NMR (400 MHz, $CDCl_3$)

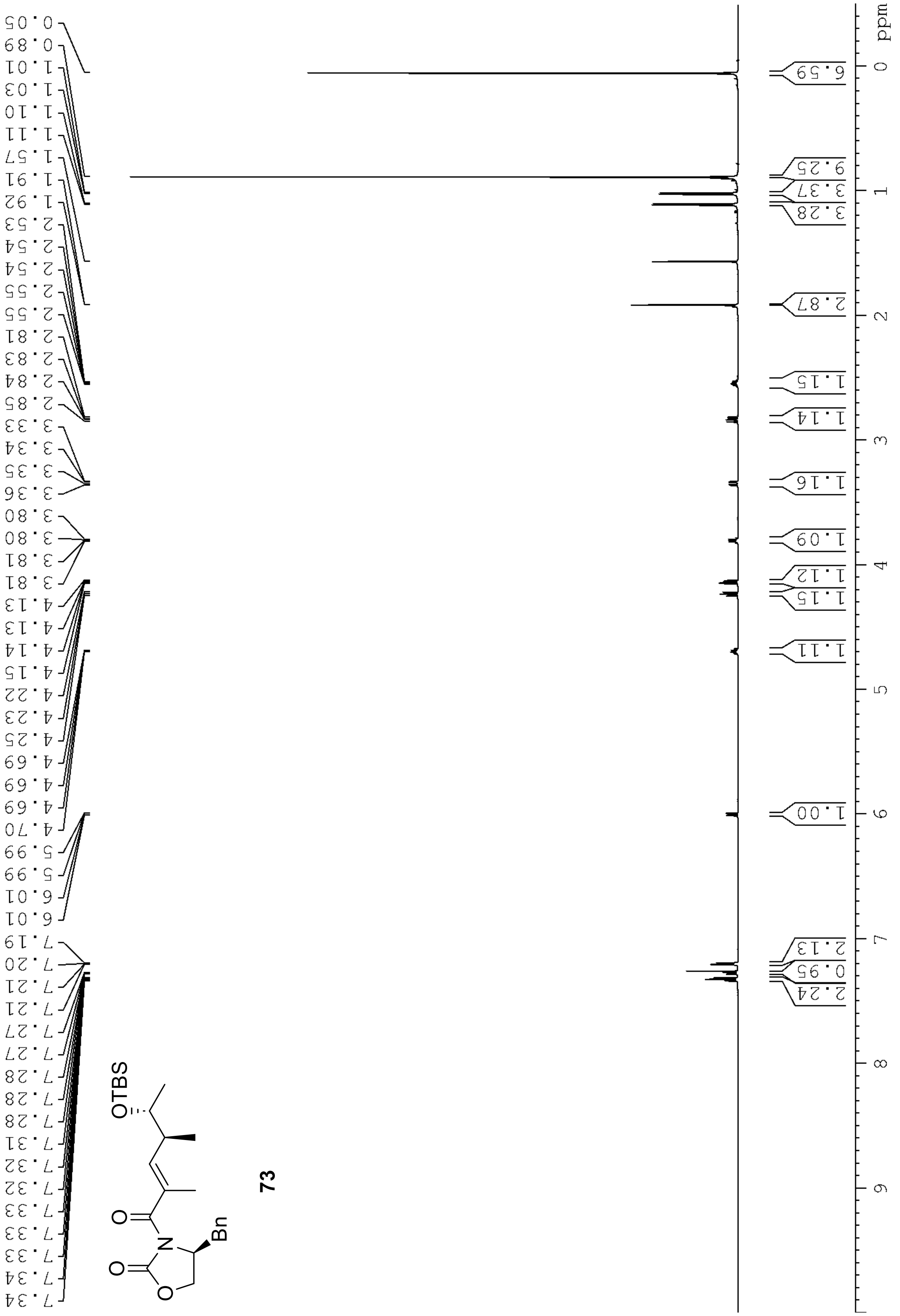

$^{13}C\{^1H\}$-NMR (100 MHz, $CDCl_3$)

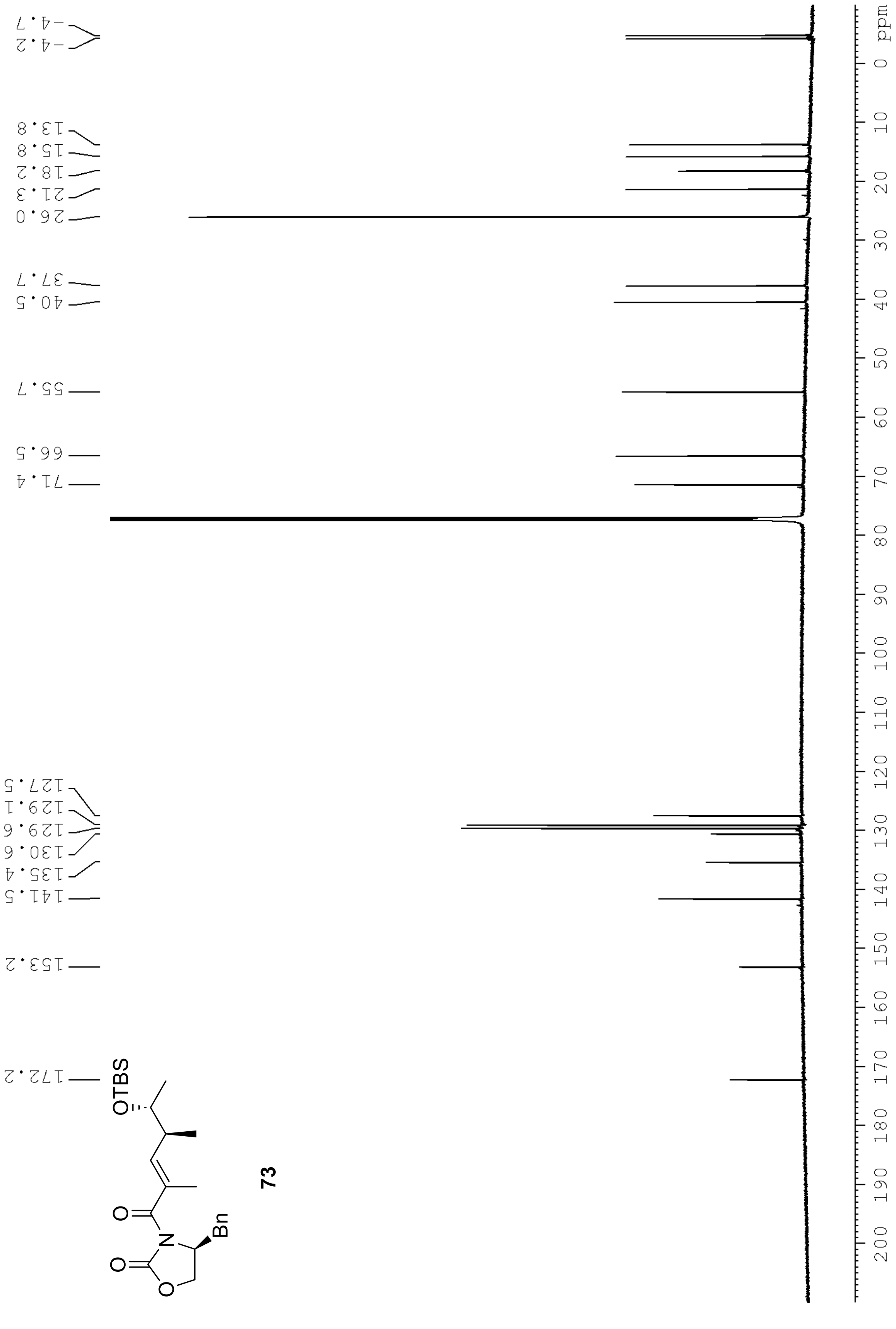

Alcohol **74**

^{1}H-NMR (400 MHz, $CDCl_3$)

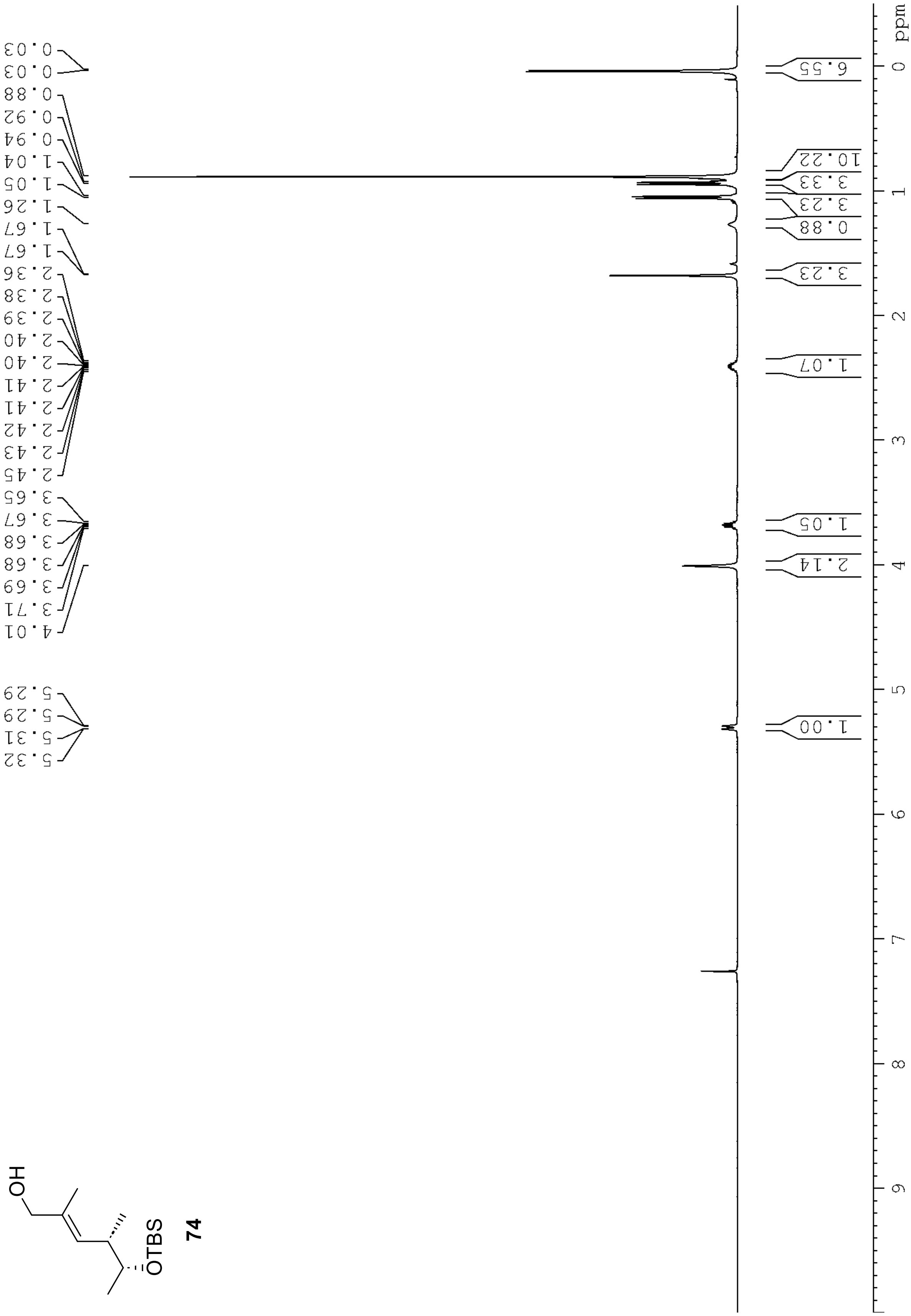

^{13}C{1H}-NMR (100 MHz, $CDCl_3$)

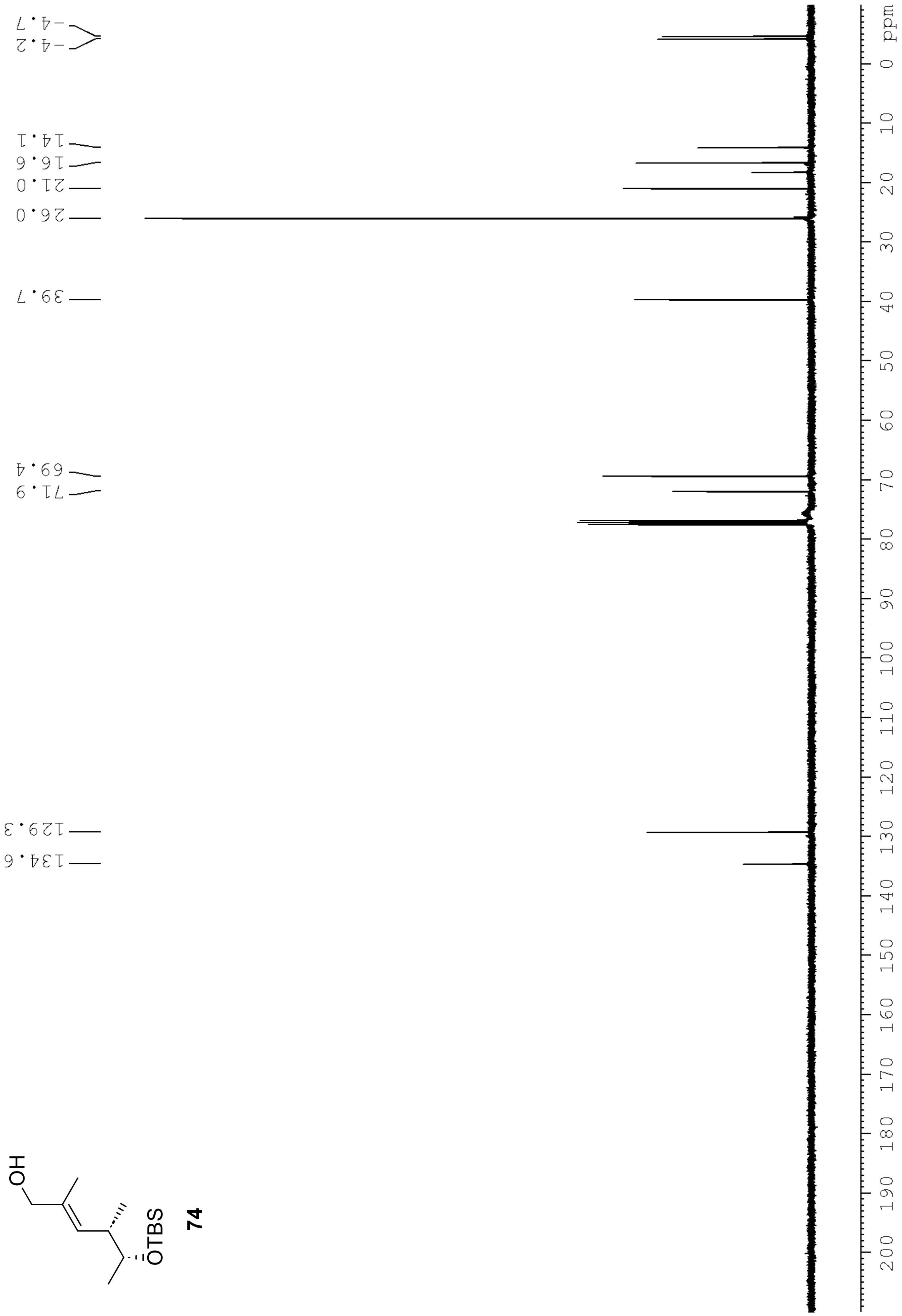

Pivalate **36**

^{1}H-NMR (400 MHz, $CDCl_3$)

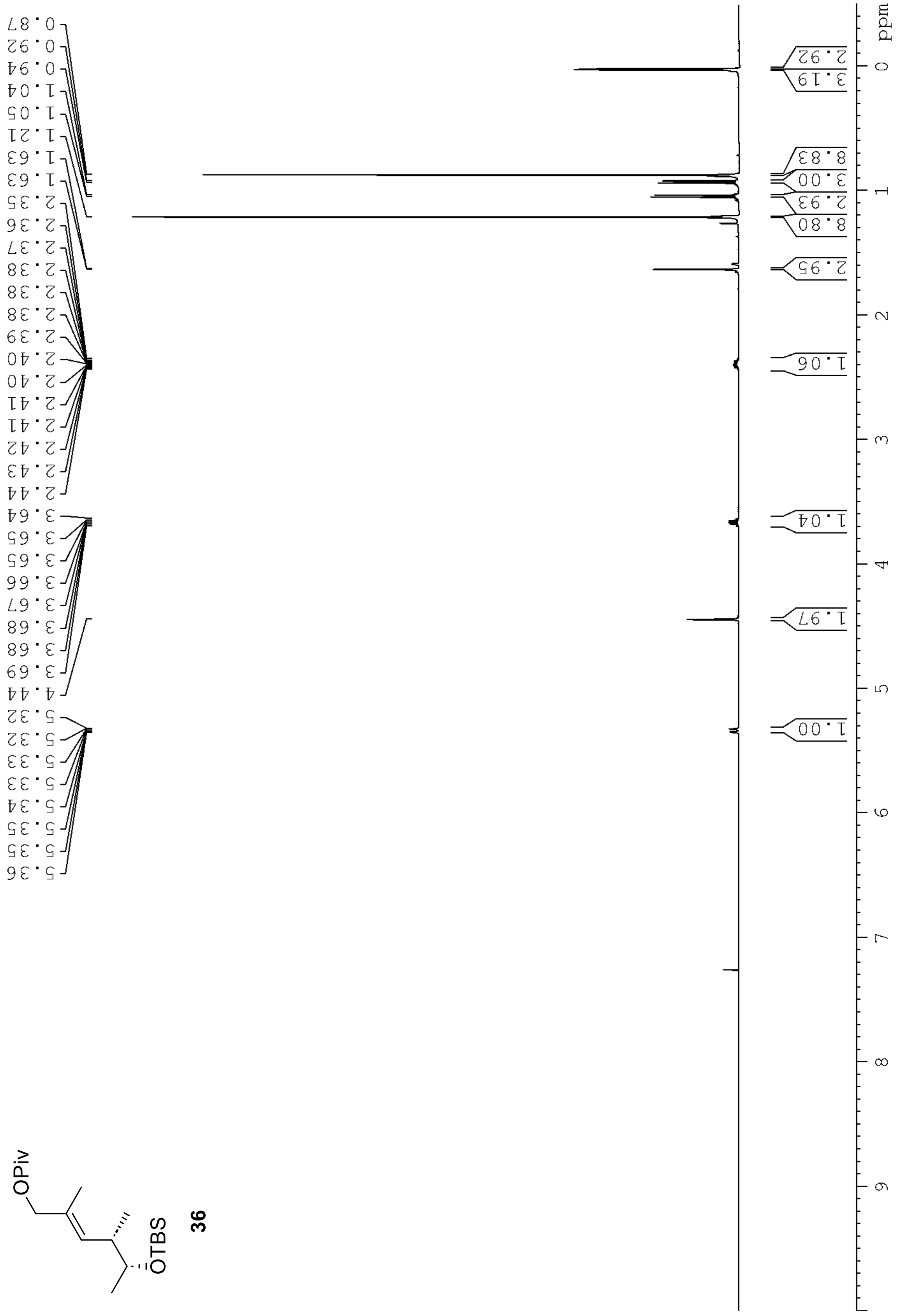

$^{13}C\{1H\}$-NMR (100 MHz, $CDCl_3$)

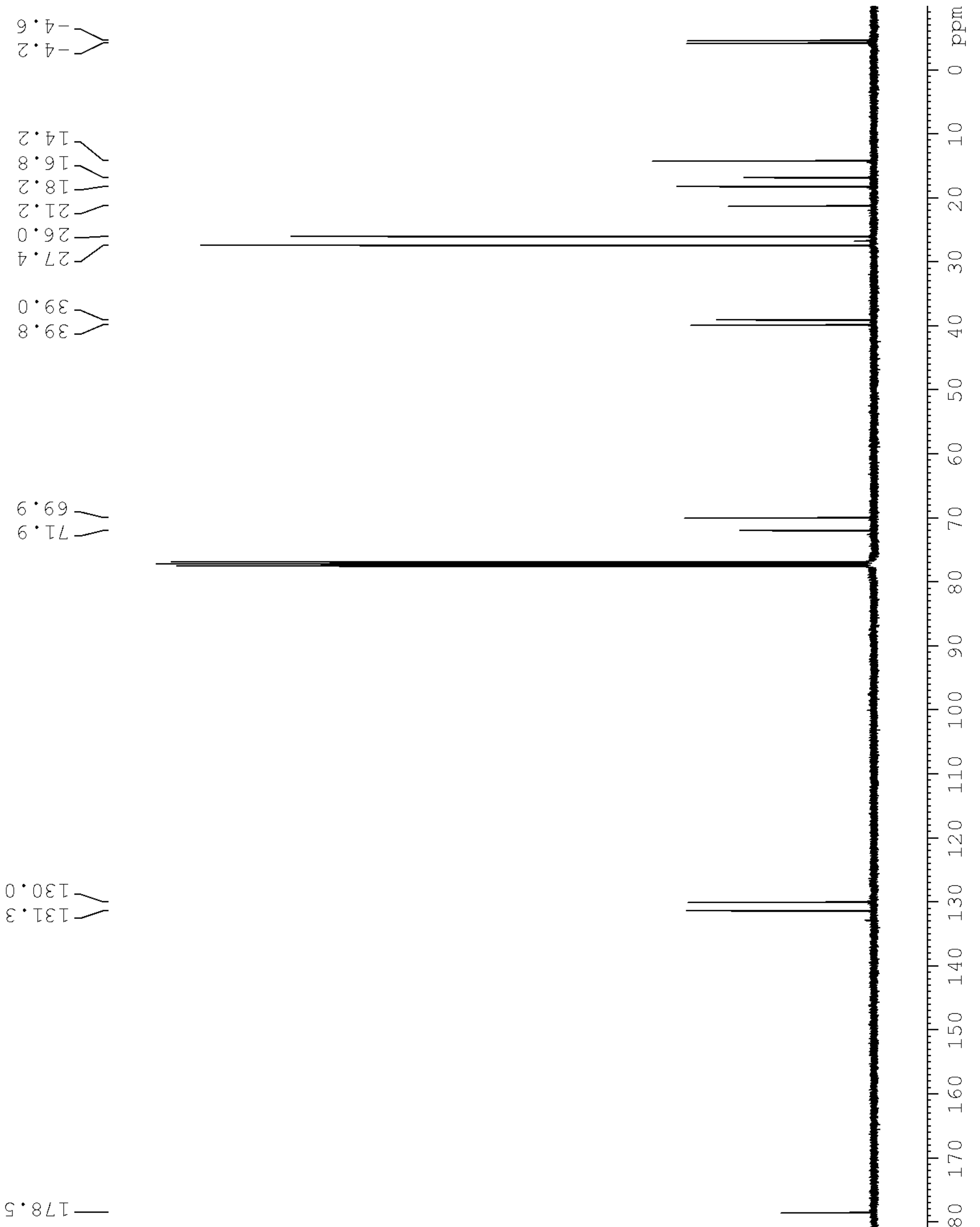

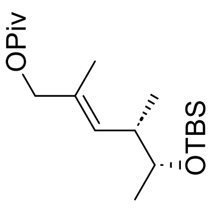

36

Alcohol **75**

^{1}H-NMR (400 MHz, $CDCl_3$)

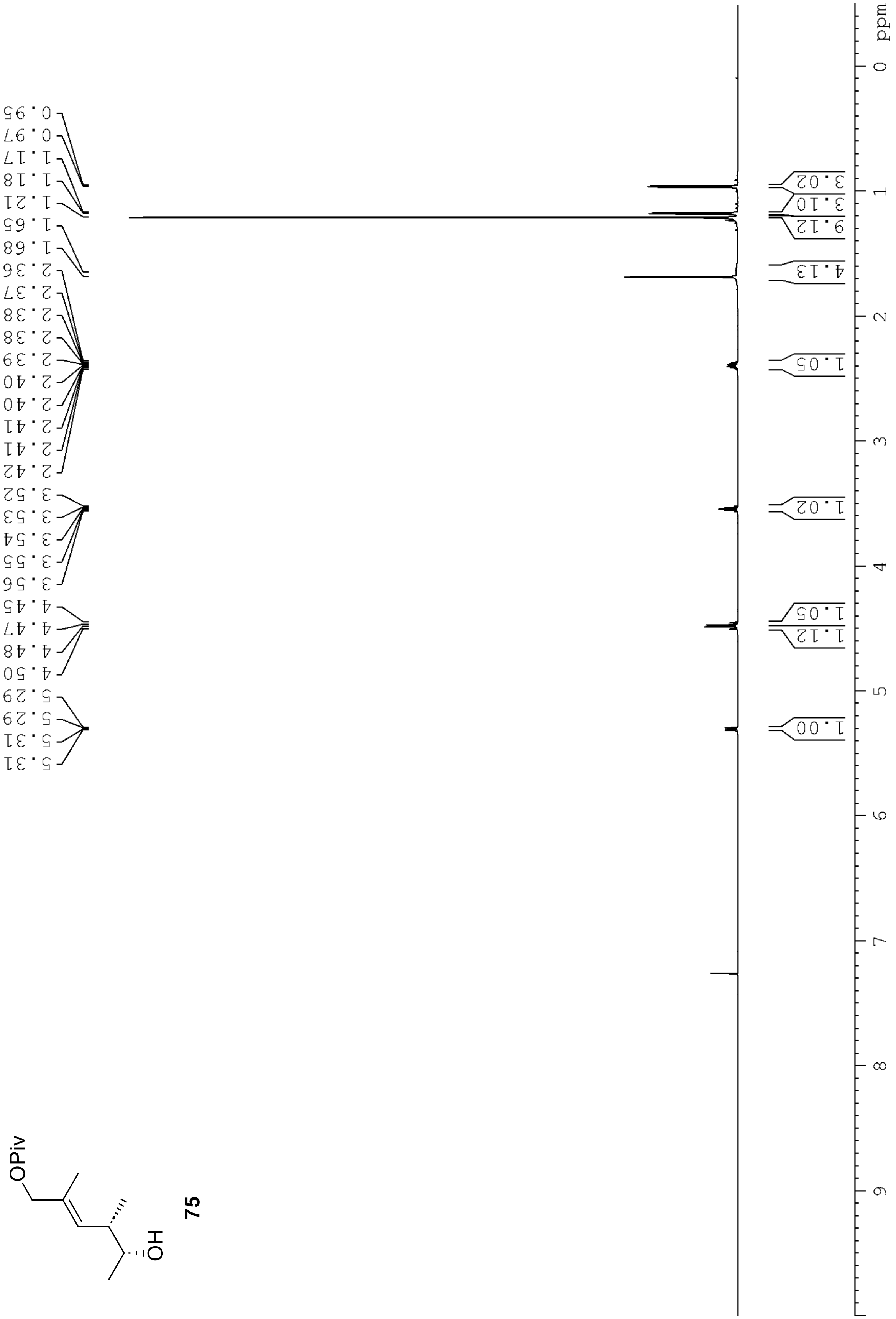

75

$^{13}C\{1H\}$-NMR (100 MHz, $CDCl_3$)

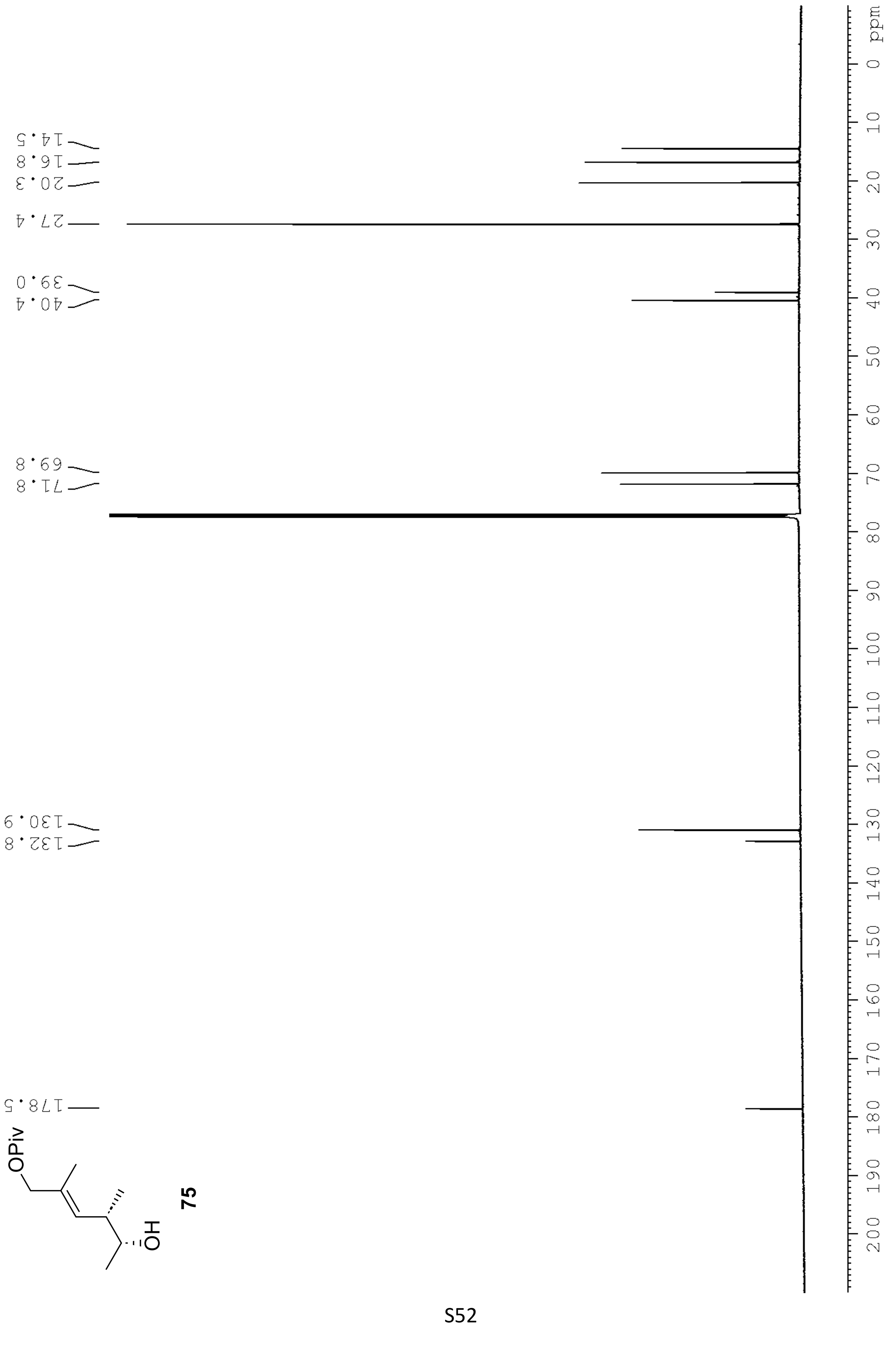

75

Eastern fragment **15**

^{1}H-NMR (400 MHz, $CDCl_3$)

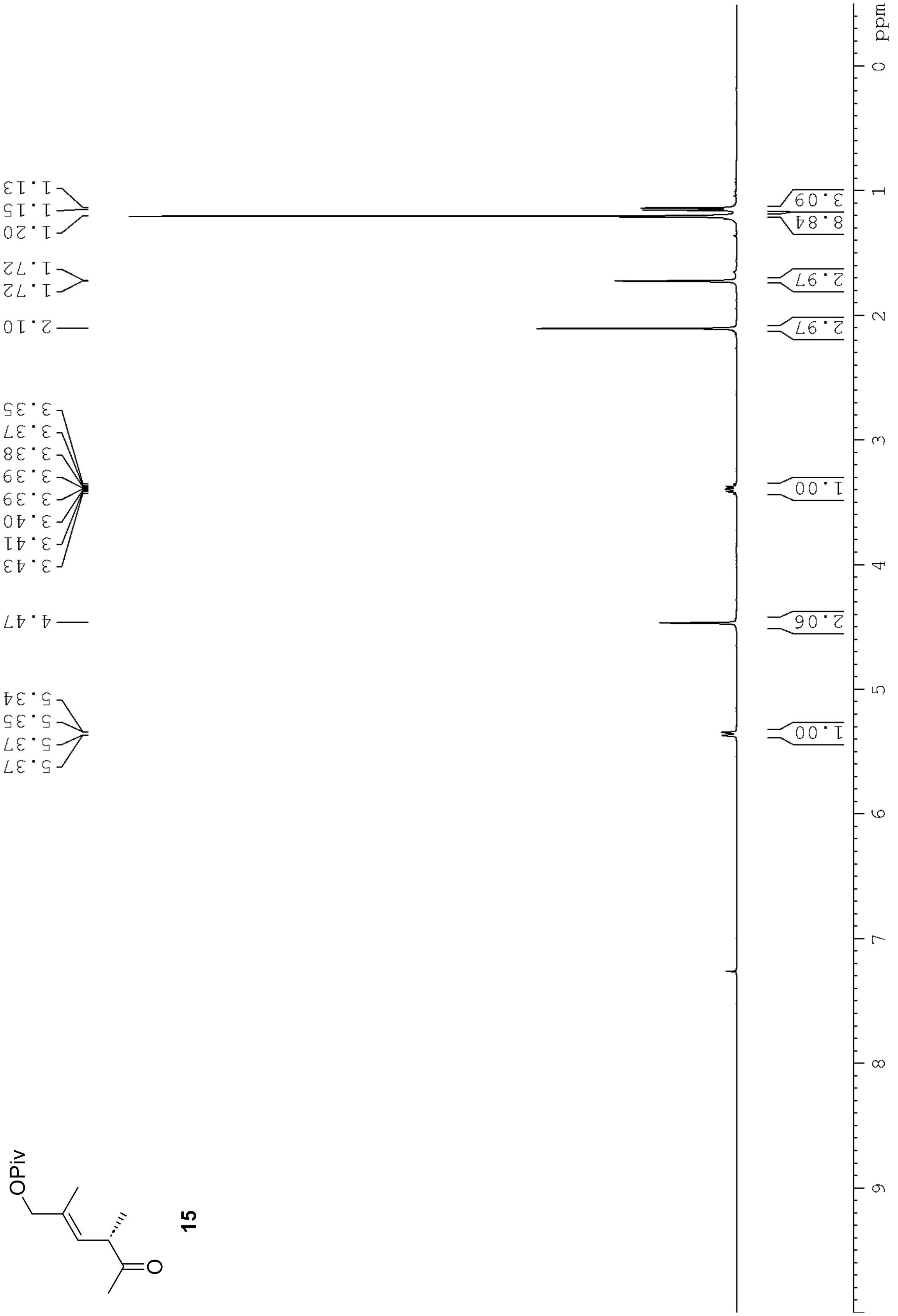

15

$^{13}C\{1H\}$-NMR (100 MHz, $CDCl_3$)

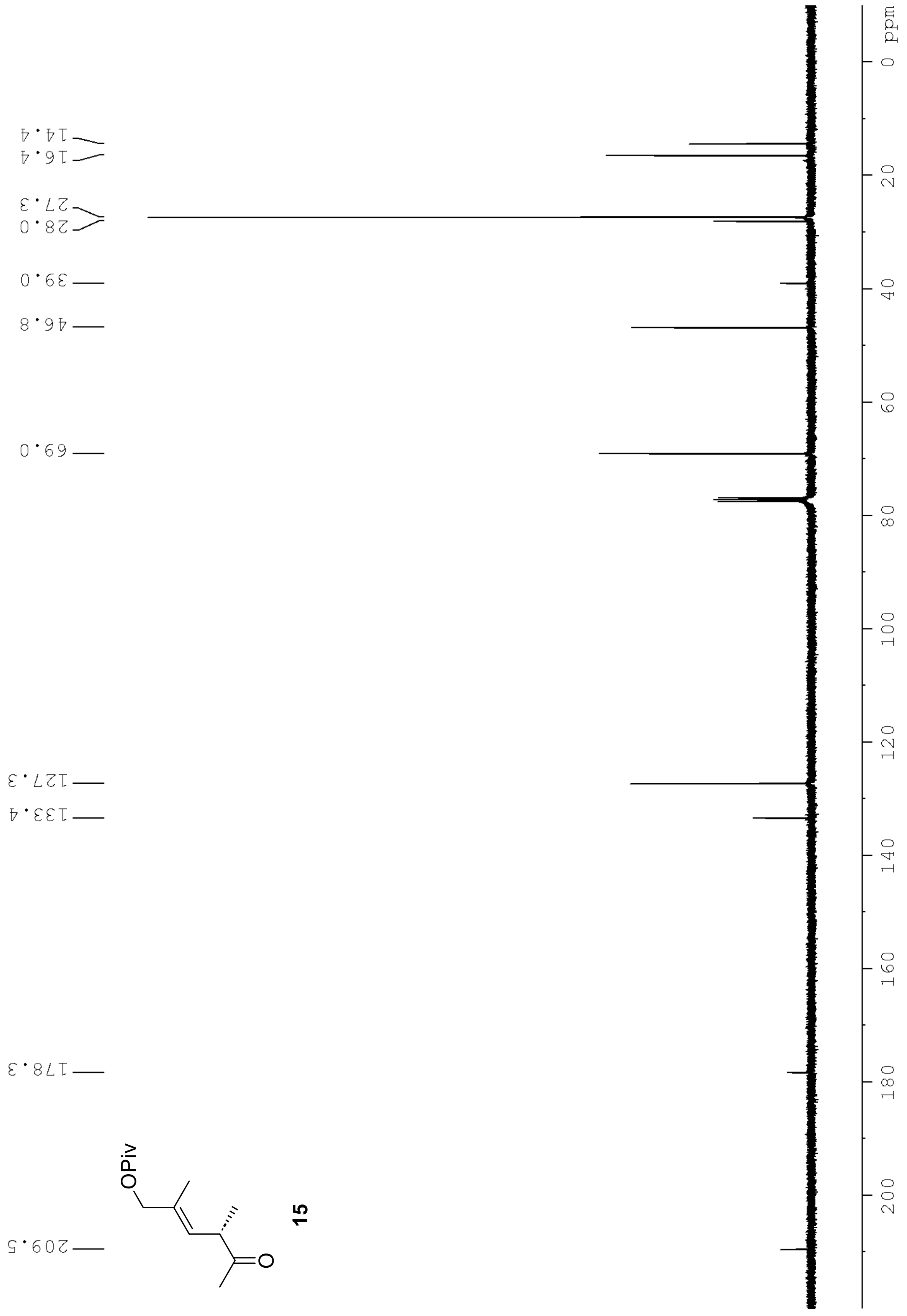

Alcohol **76**

^{1}H-NMR (400 MHz, $CDCl_3$)

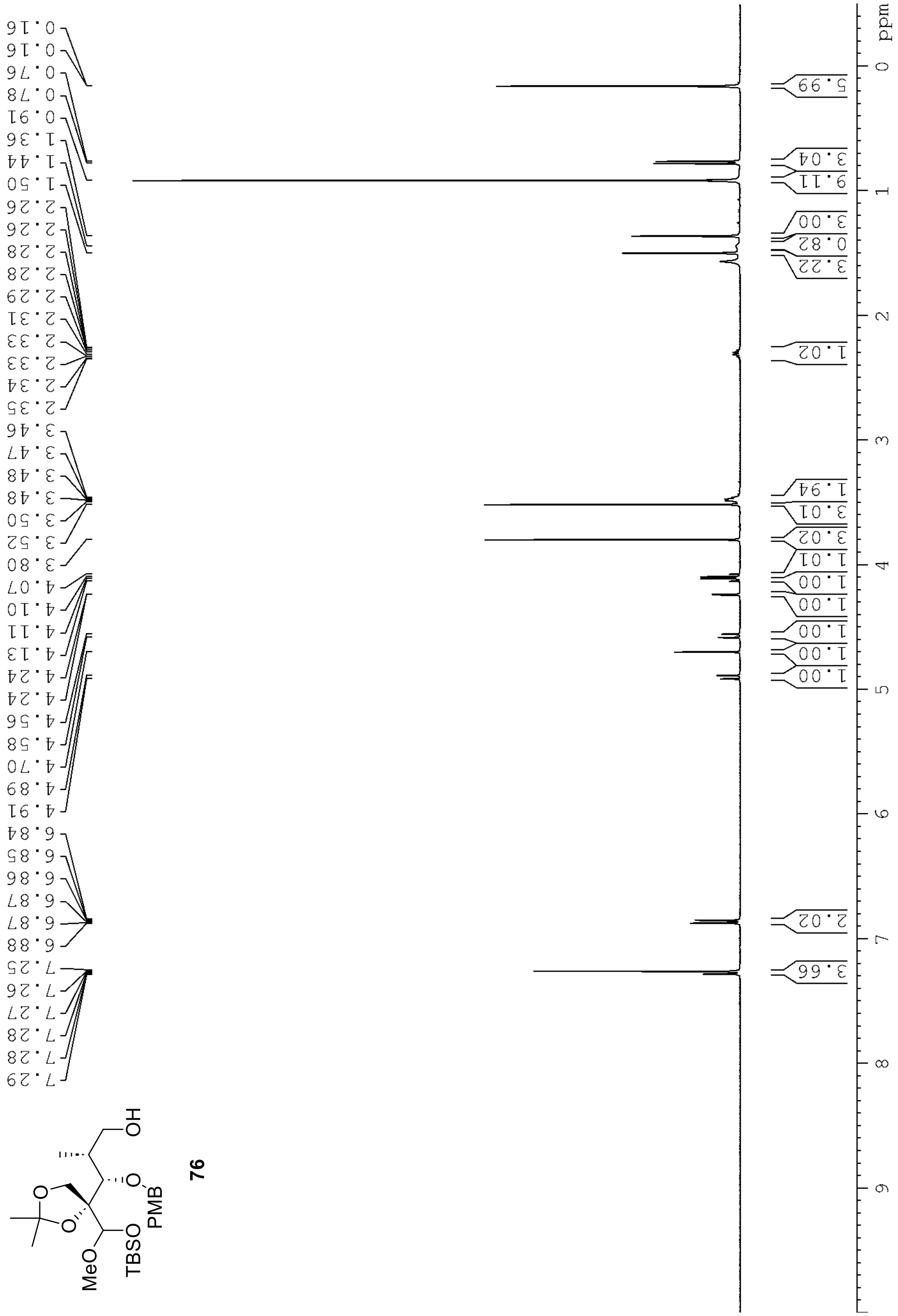

76

$^{13}C\{1H\}$-NMR (100 MHz, $CDCl_3$)

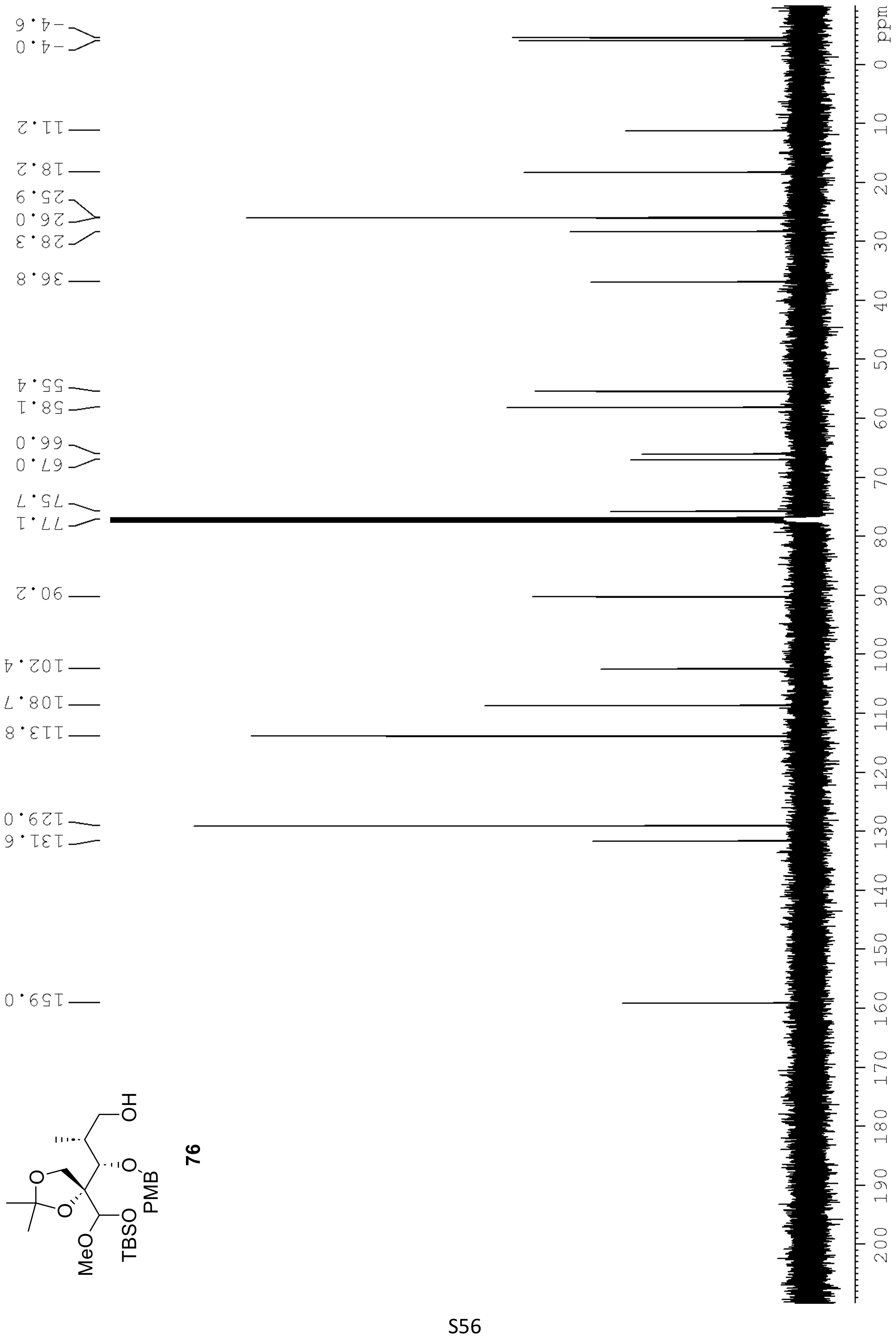

76

Pivalate **77**

^{1}H-NMR (400 MHz, $CDCl_3$)

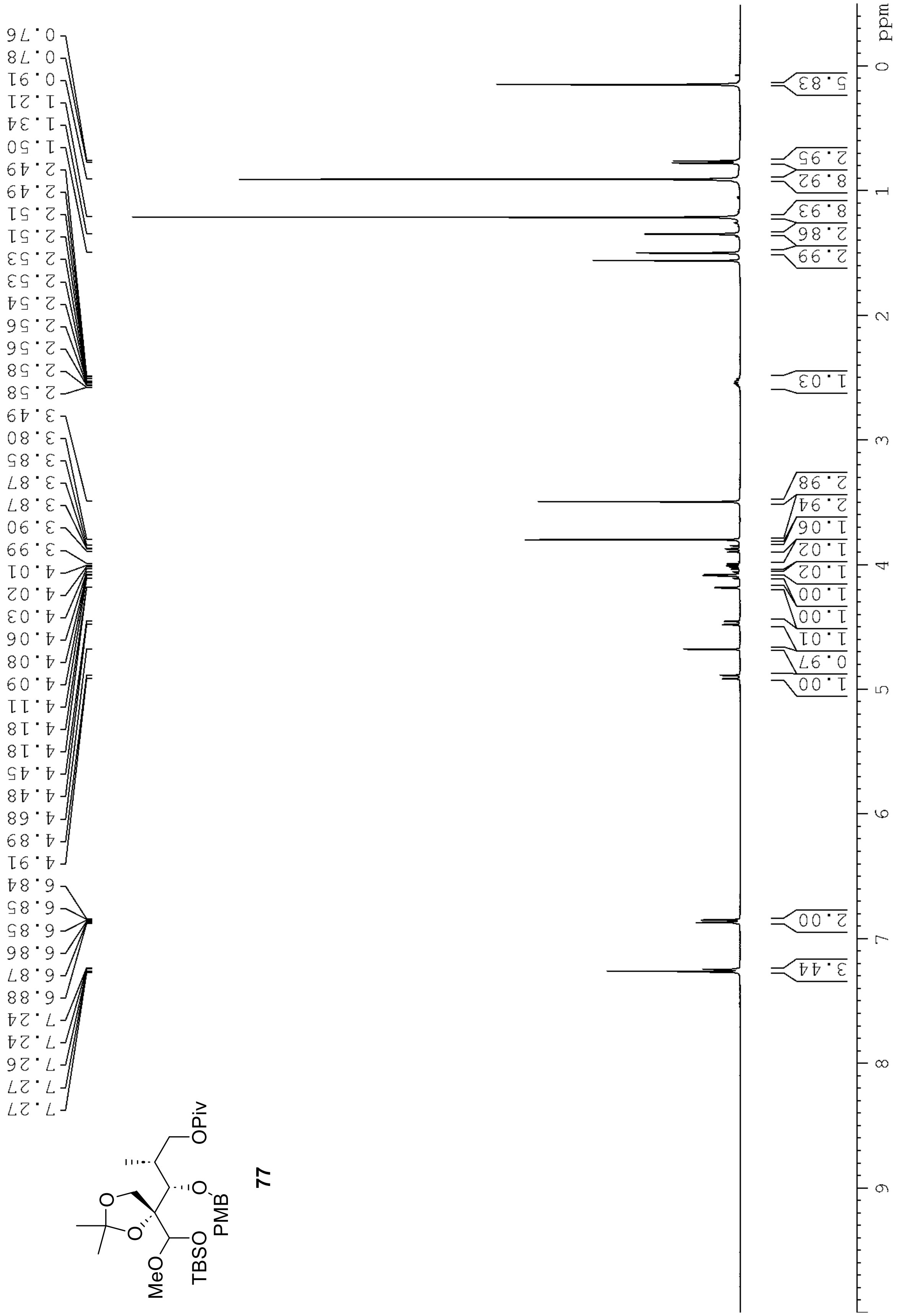

$^{13}C\{1H\}$-NMR (100 MHz, $CDCl_3$)

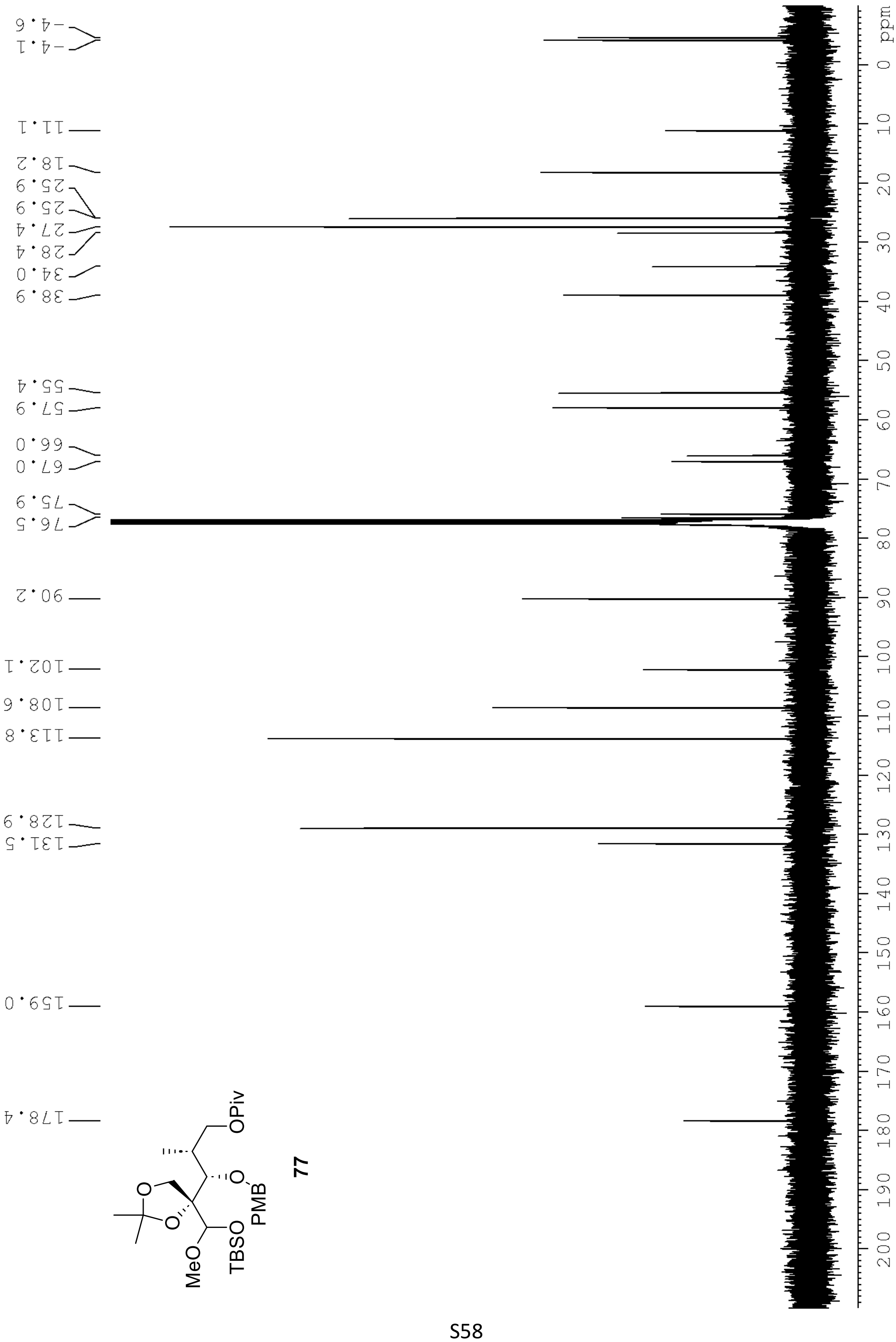

Alcohol **78**

^{1}H-NMR (400 MHz, $CDCl_3$)

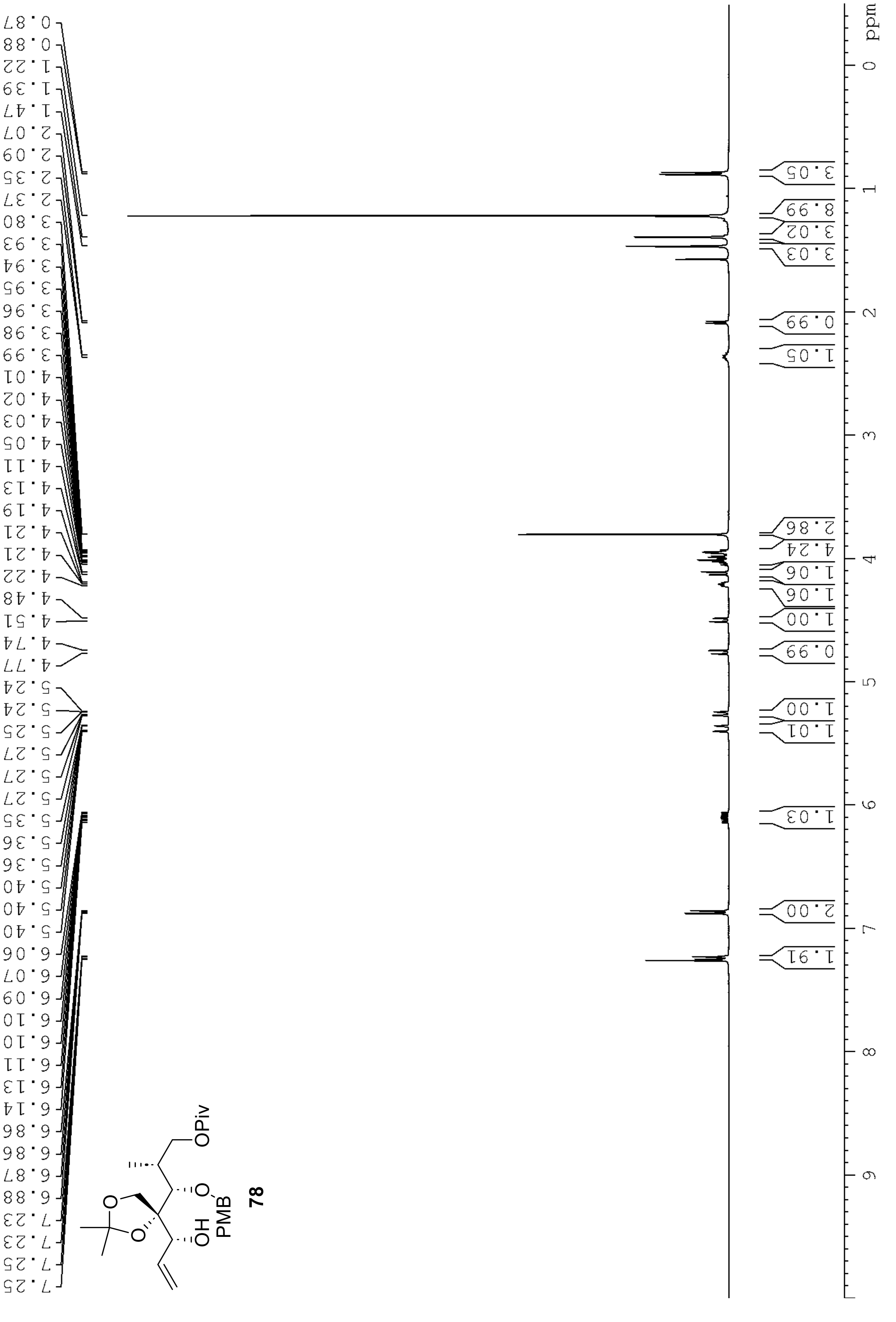

$^{13}C\{1H\}$-NMR (100 MHz, $CDCl_3$)

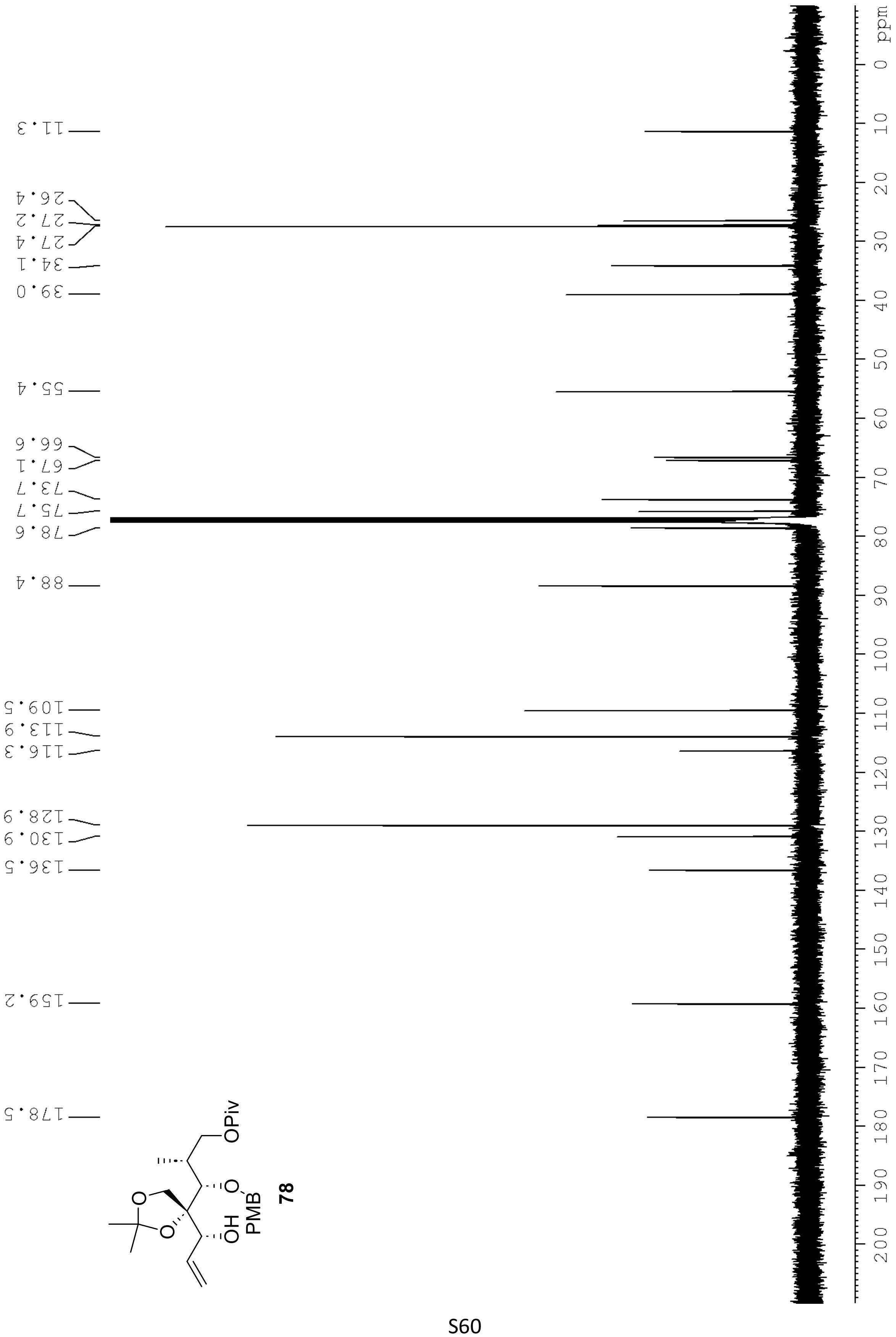

78

Acetonide **S5**

^{1}H-NMR (400 MHz, $CDCl_3$)

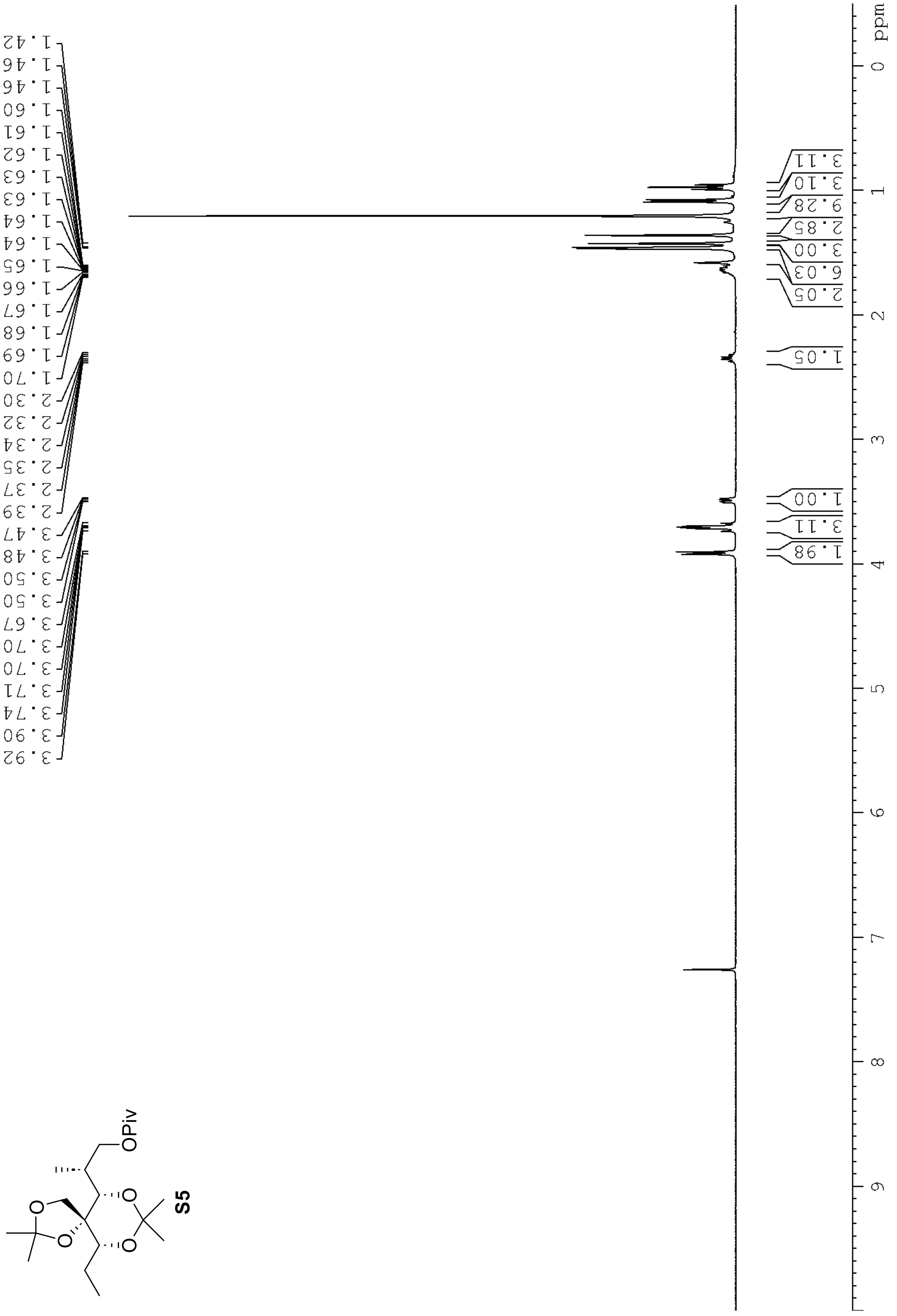

^{13}C{1H}-NMR (100 MHz, $CDCl_3$)

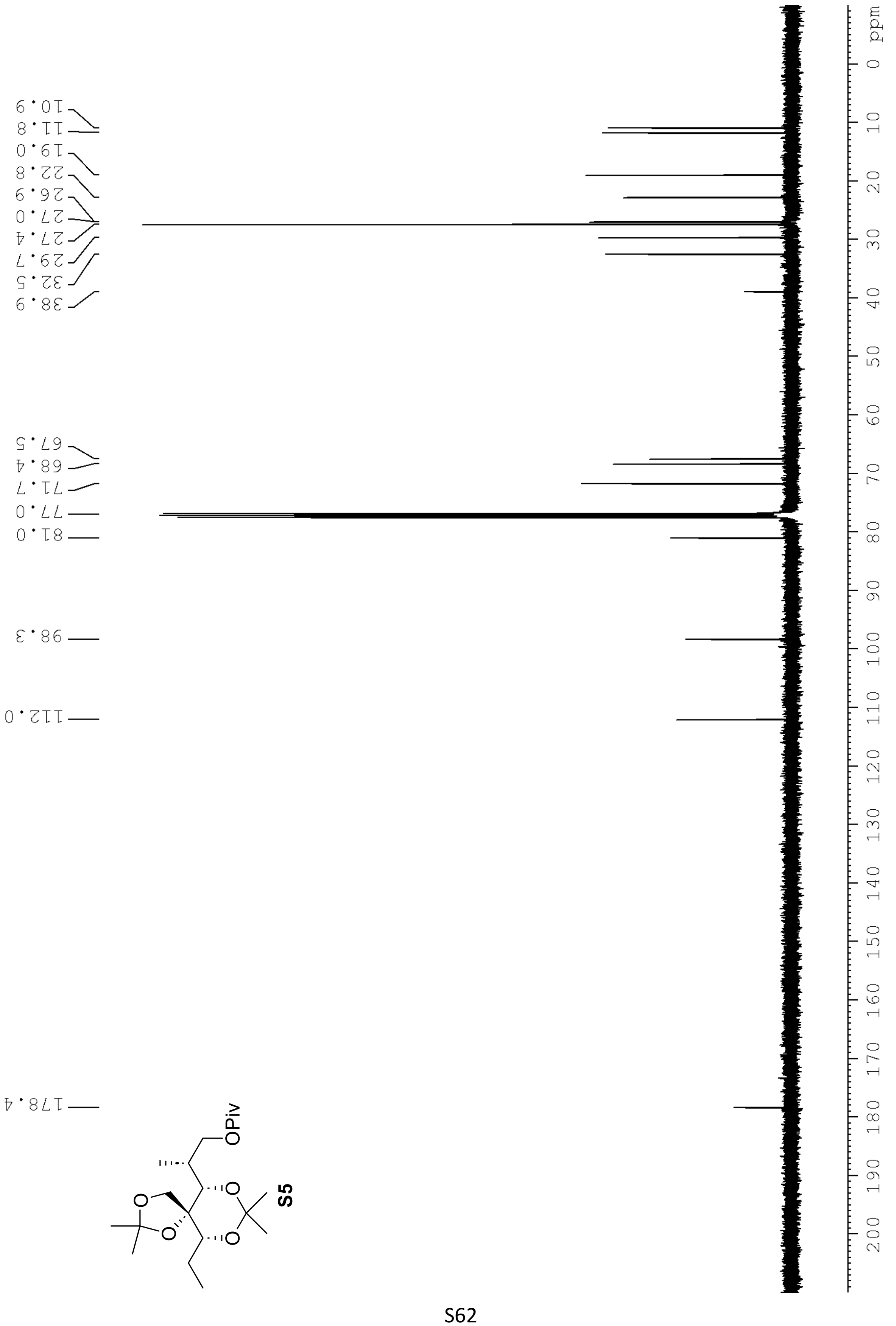

TES-ether **79**
^{1}H-NMR (400 MHz, $CDCl_3$)

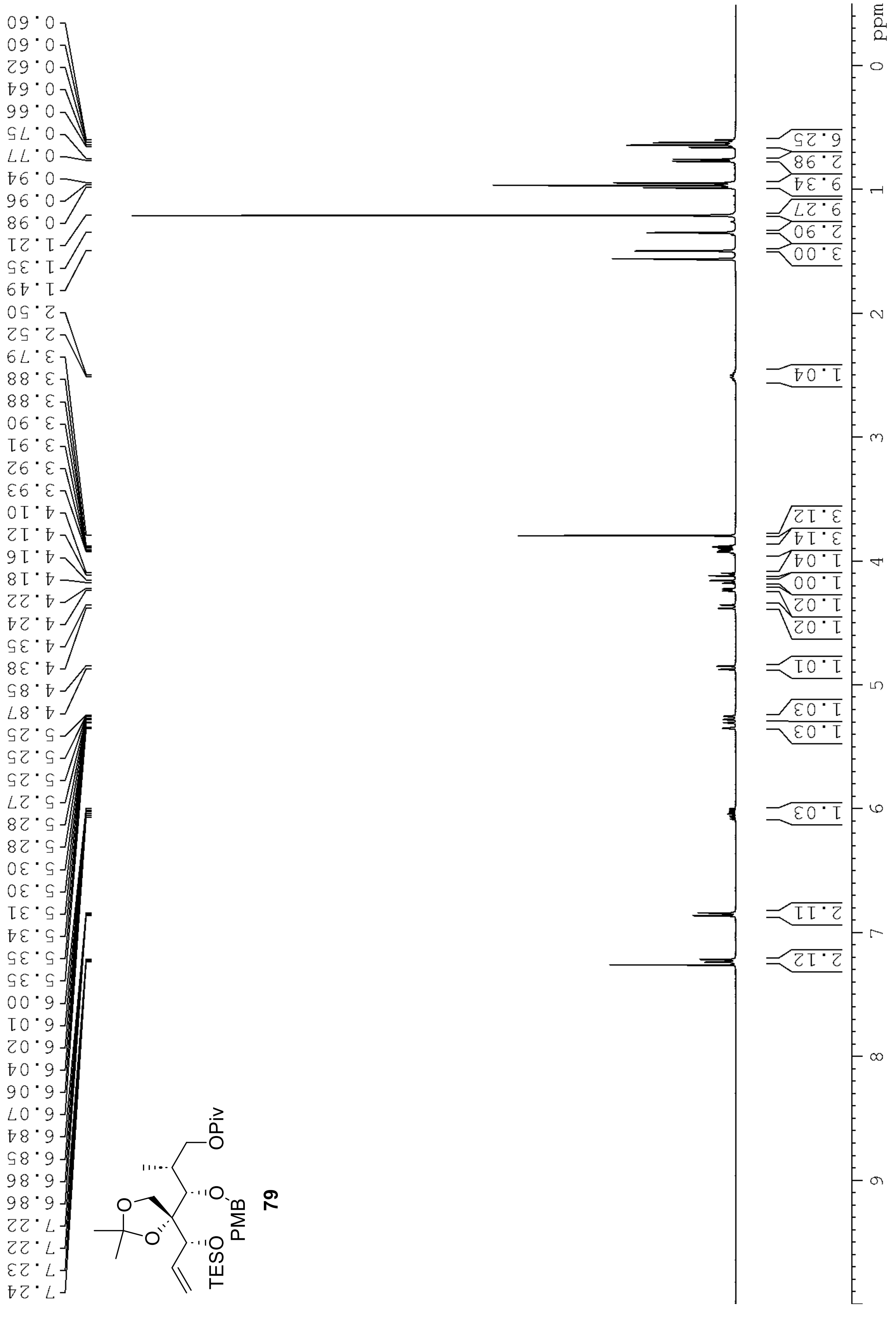

$^{13}C\{1H\}$-NMR (100 MHz, $CDCl_3$)

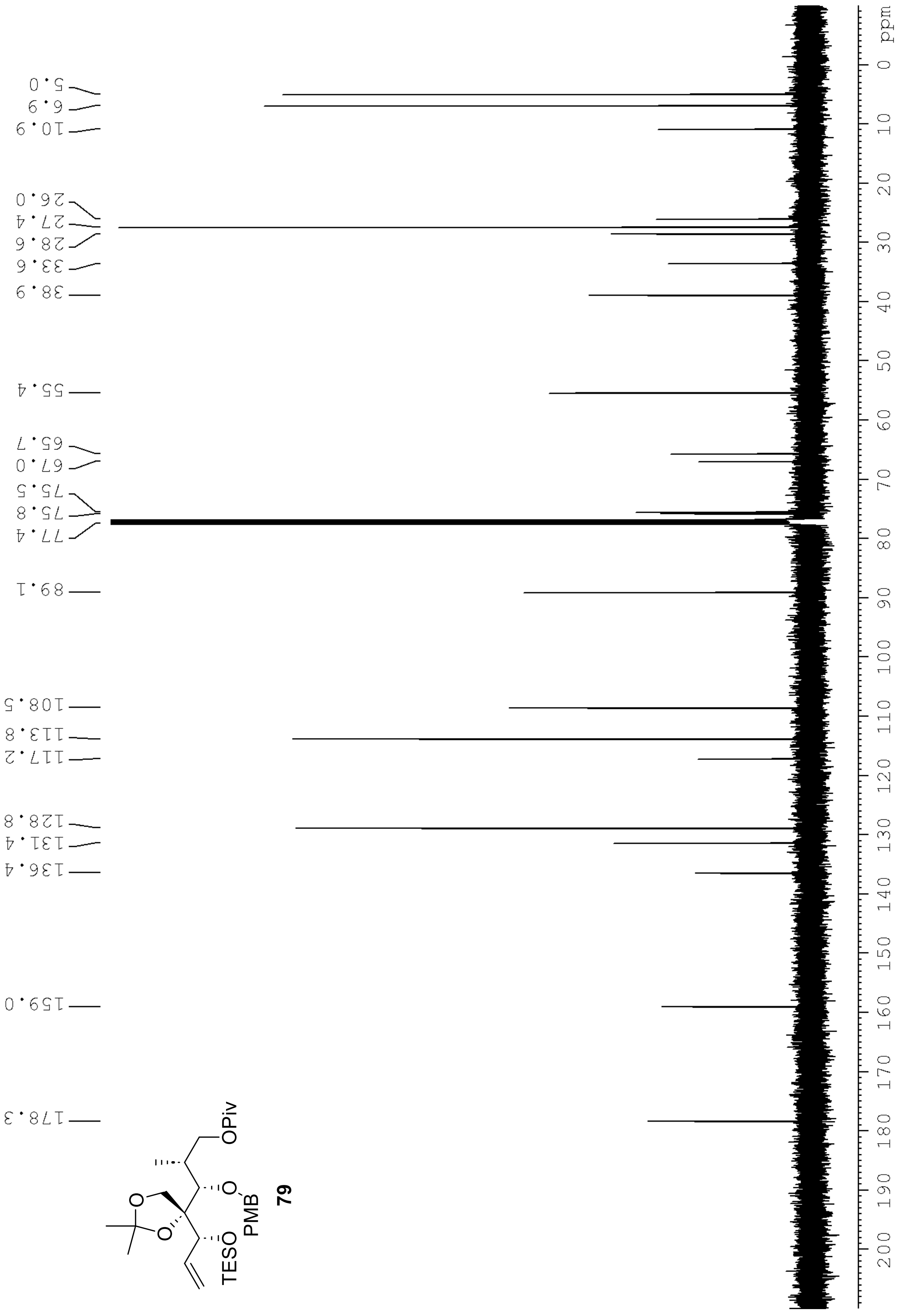

Sulfide **81**

^{1}H-NMR (400 MHz, $CDCl_3$)

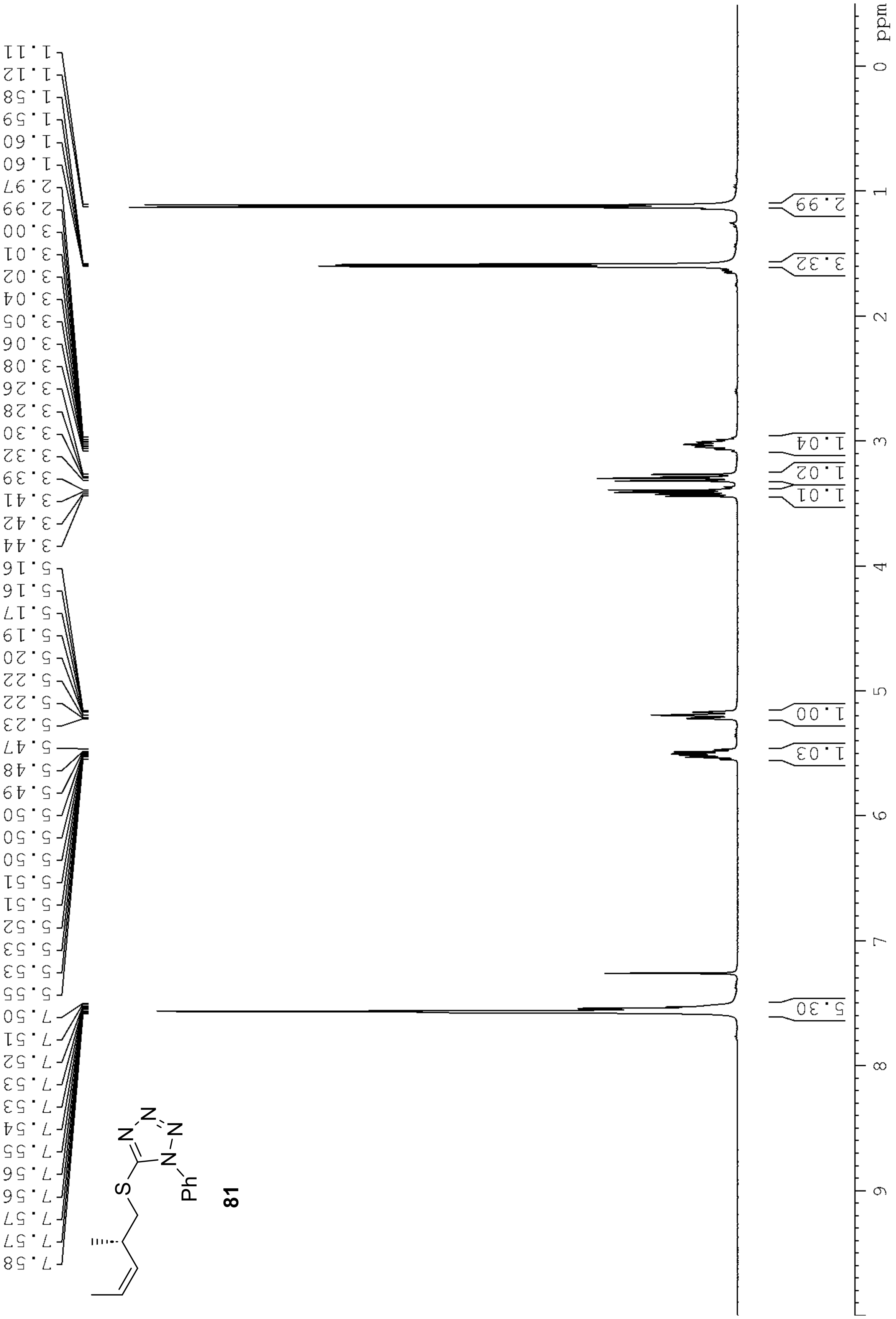

$^{13}C\{1H\}$-NMR (100 MHz, $CDCl_3$)

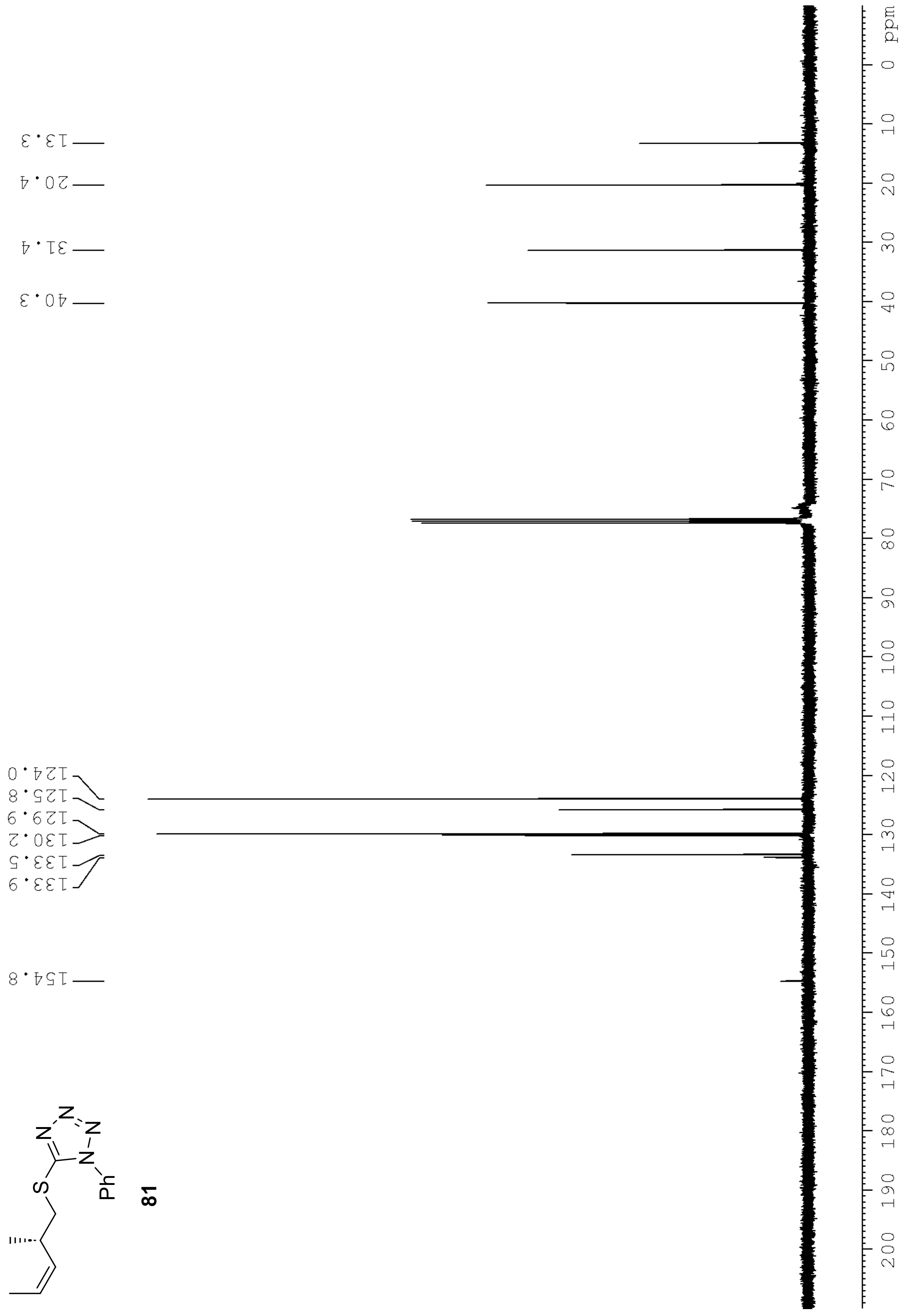

81

Sulfone **44**

^{1}H-NMR (400 MHz, $CDCl_3$)

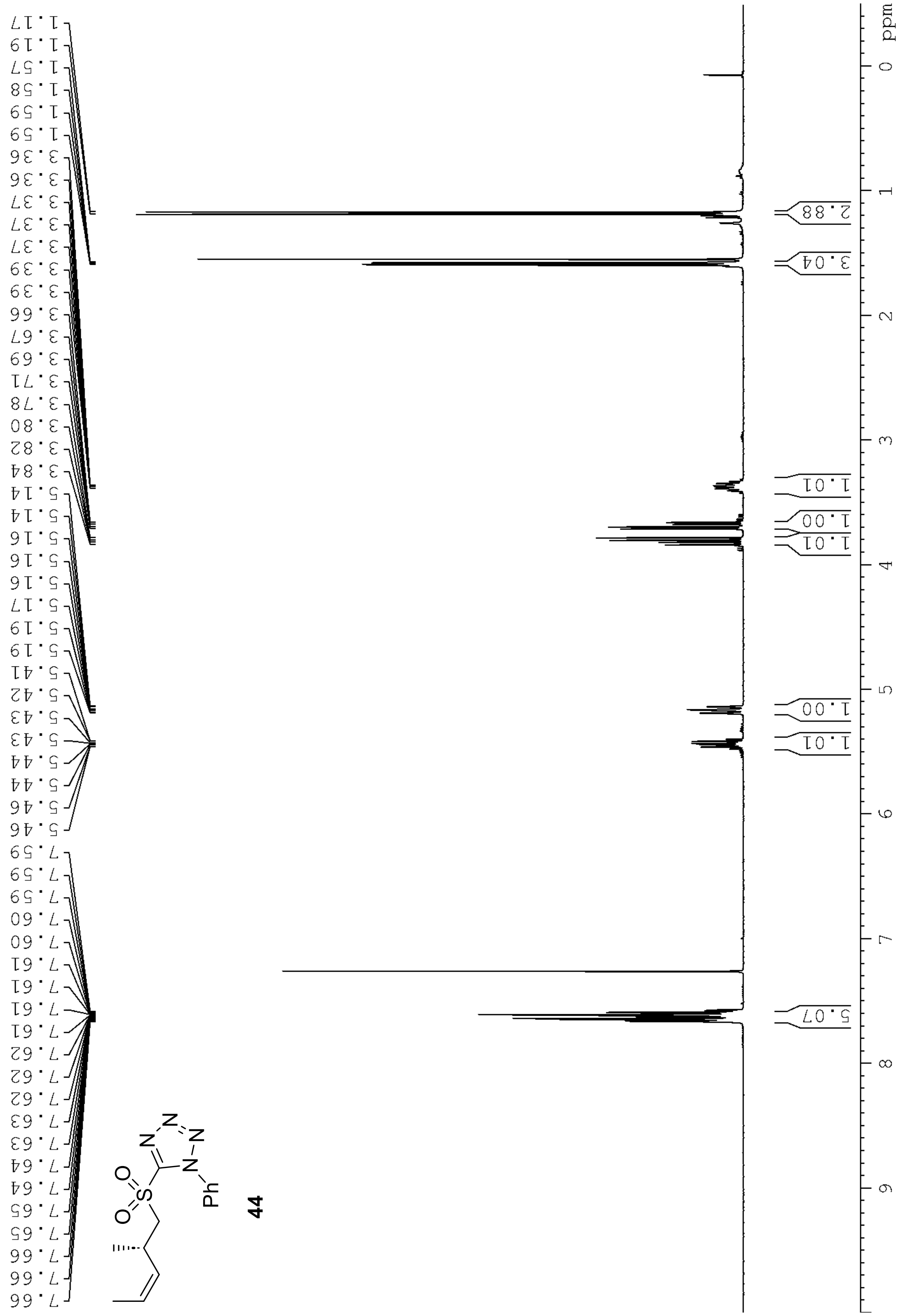

$^{13}C\{1H\}$-NMR (100 MHz, $CDCl_3$)

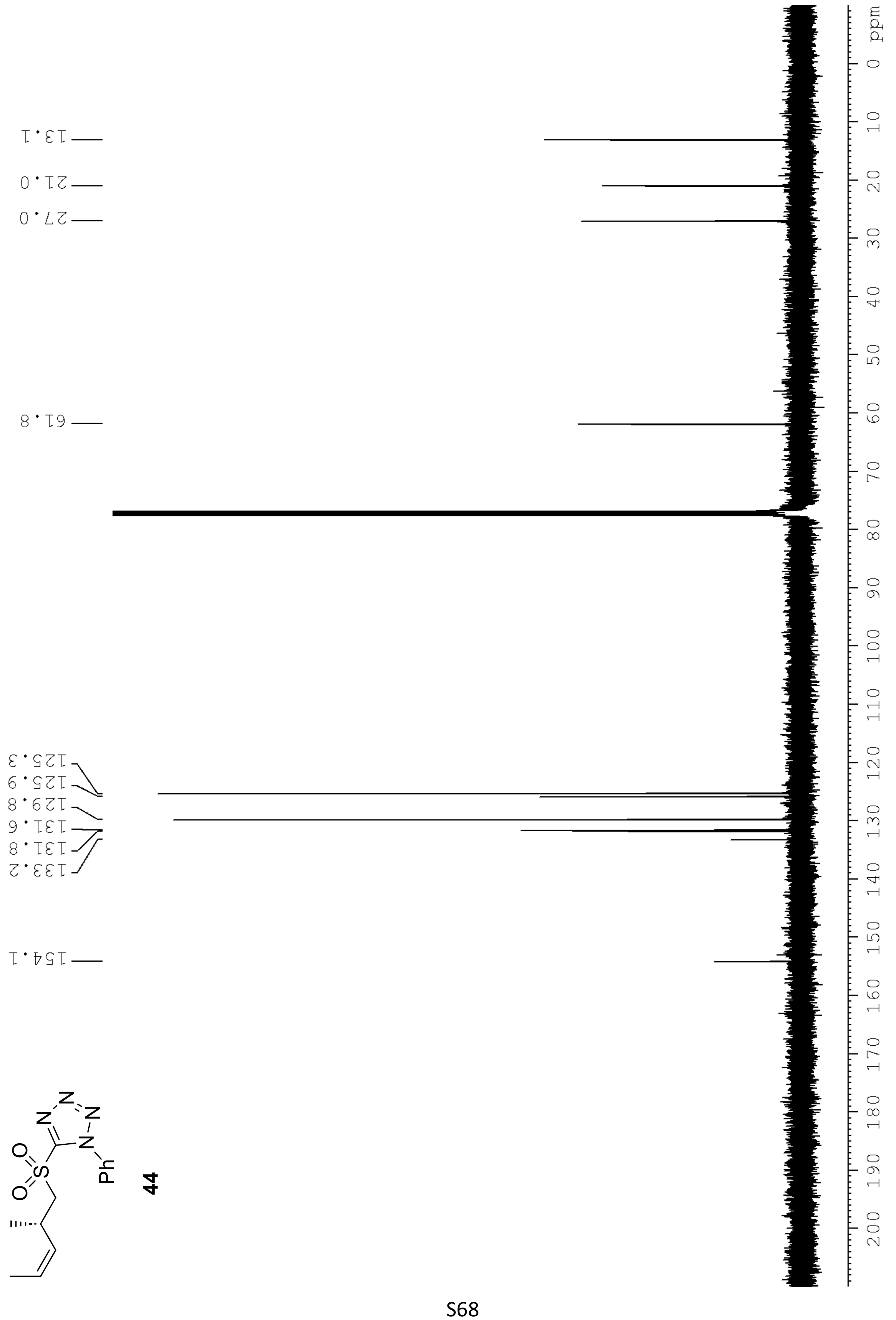

Alkene **82**

^{1}H-NMR (400 MHz, $CDCl_3$)

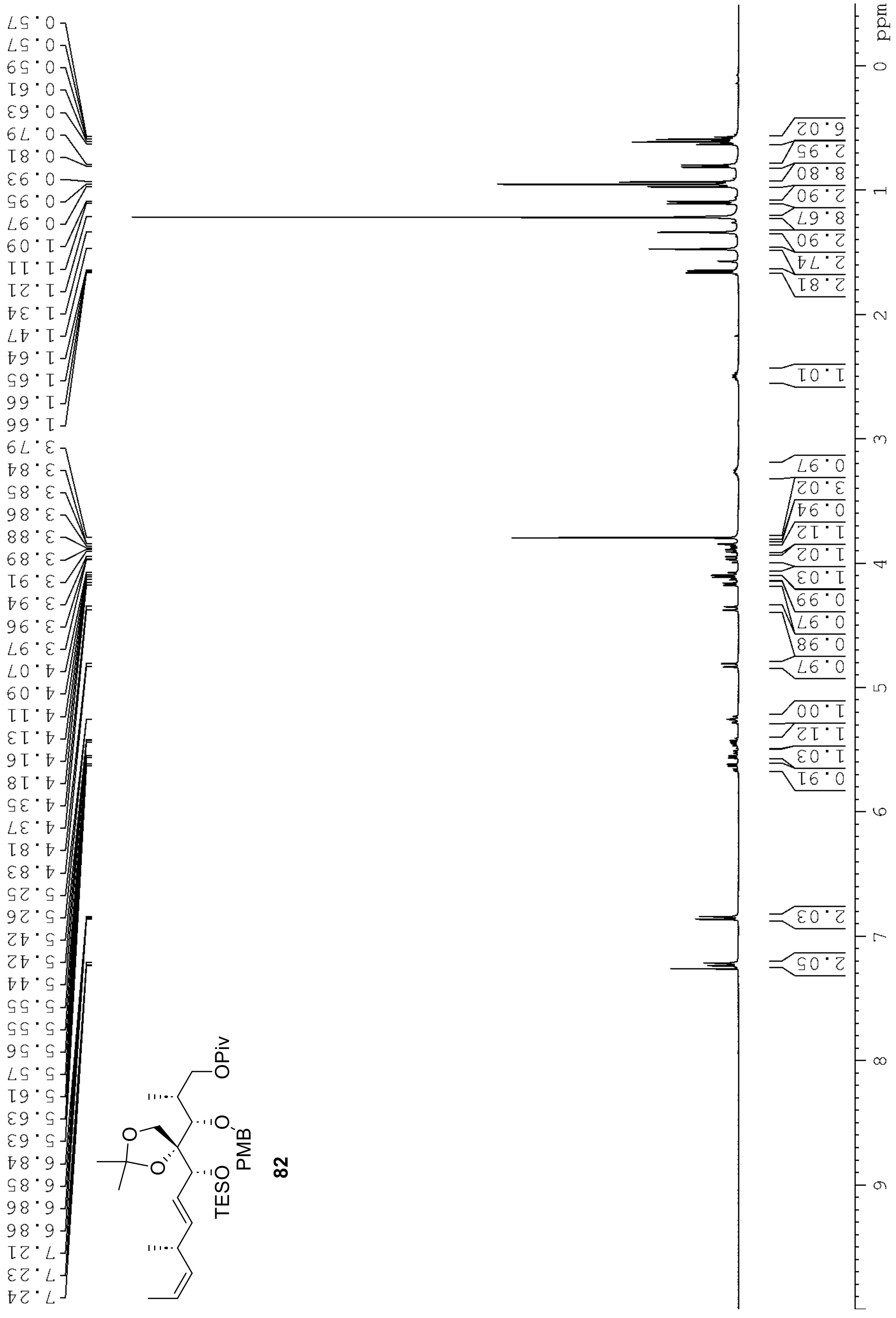

$^{13}C\{1H\}$-NMR (100 MHz, $CDCl_3$)

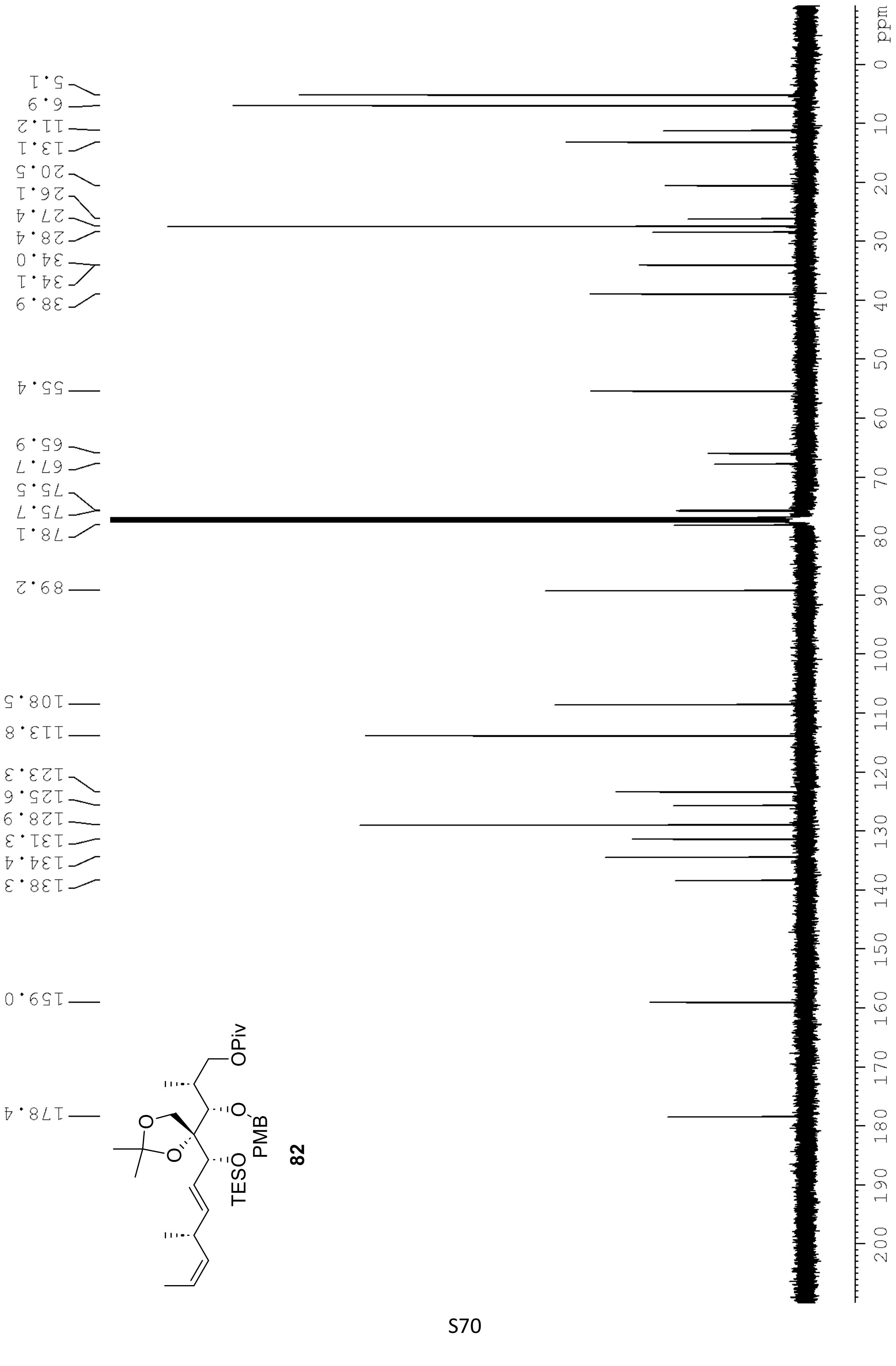

Triol **45**

^{1}H-NMR (400 MHz, $CDCl_3$)

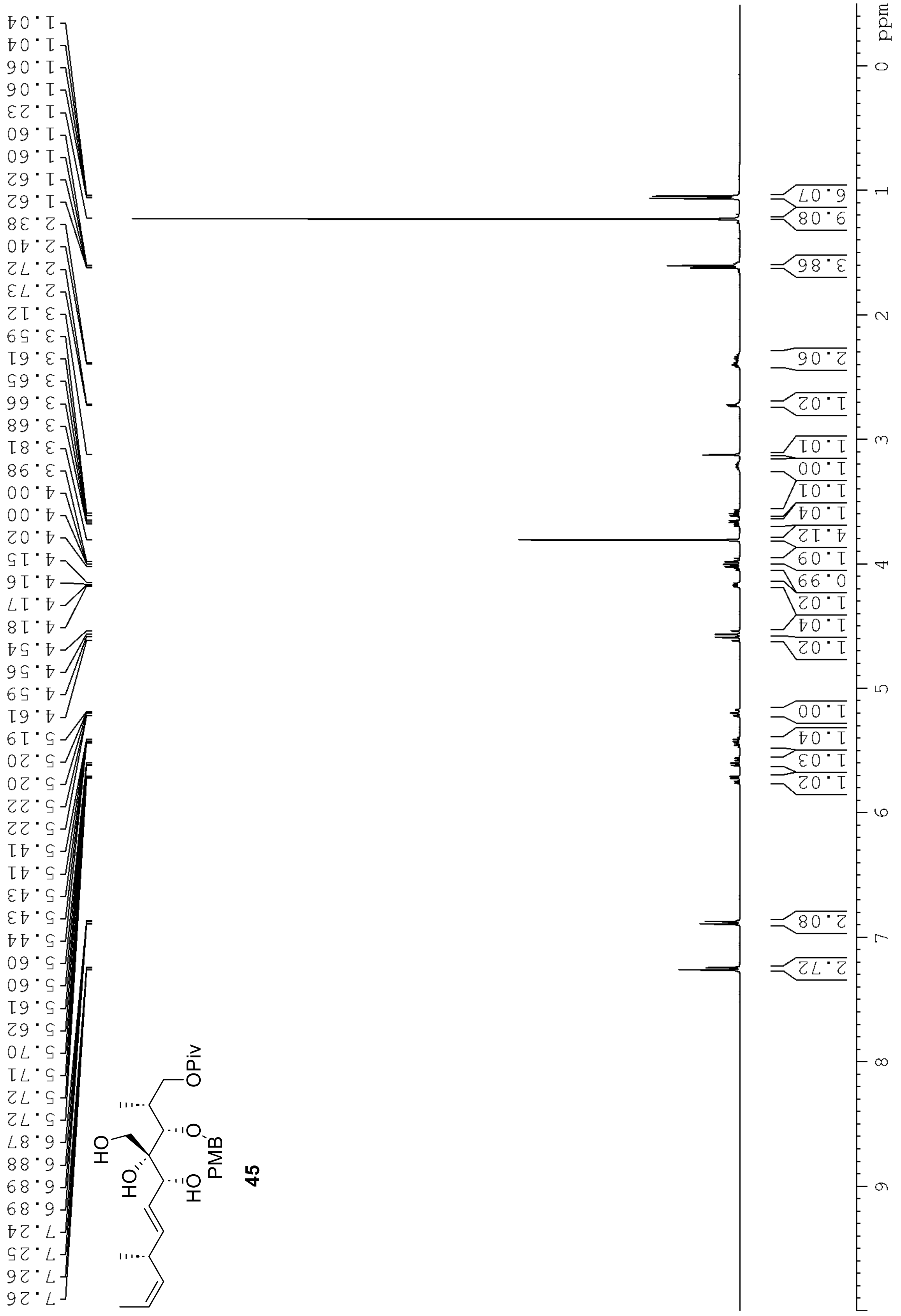

$^{13}C\{^{1}H\}$-NMR (100 MHz, $CDCl_3$)

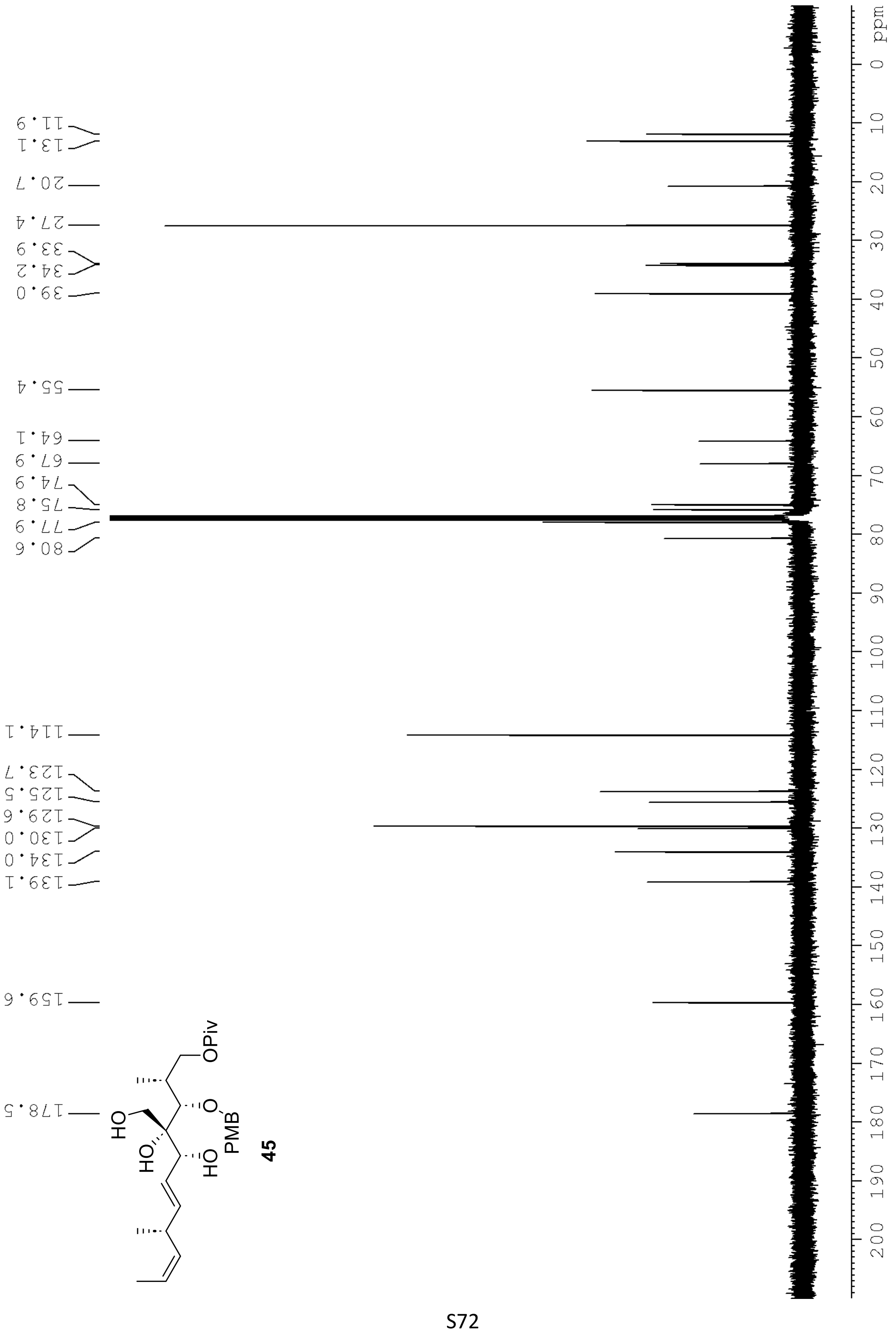

TES-ether **83**

^{1}H-NMR (400 MHz, $CDCl_3$)

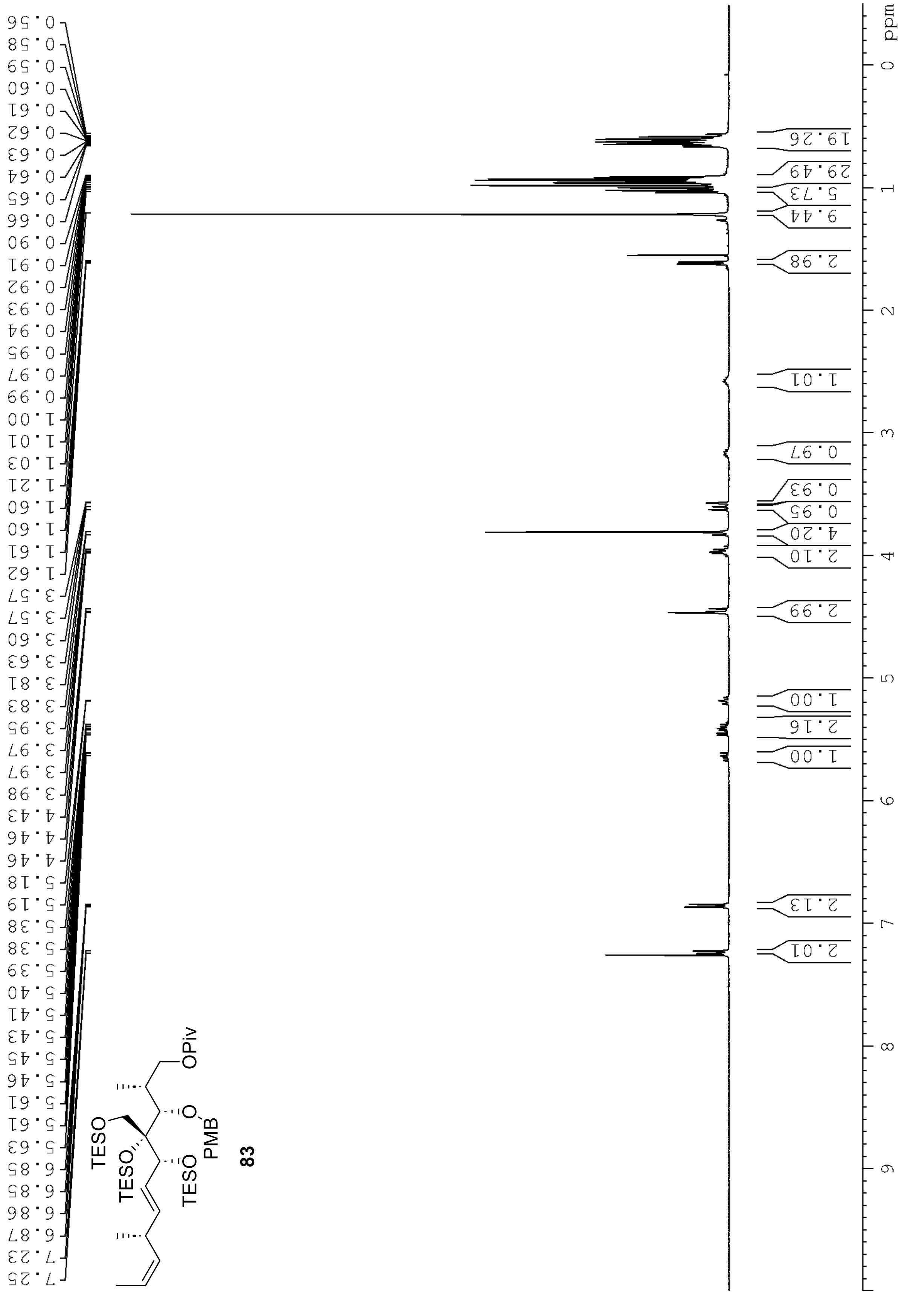

$^{13}C\{1H\}$-NMR (100 MHz, $CDCl_3$)

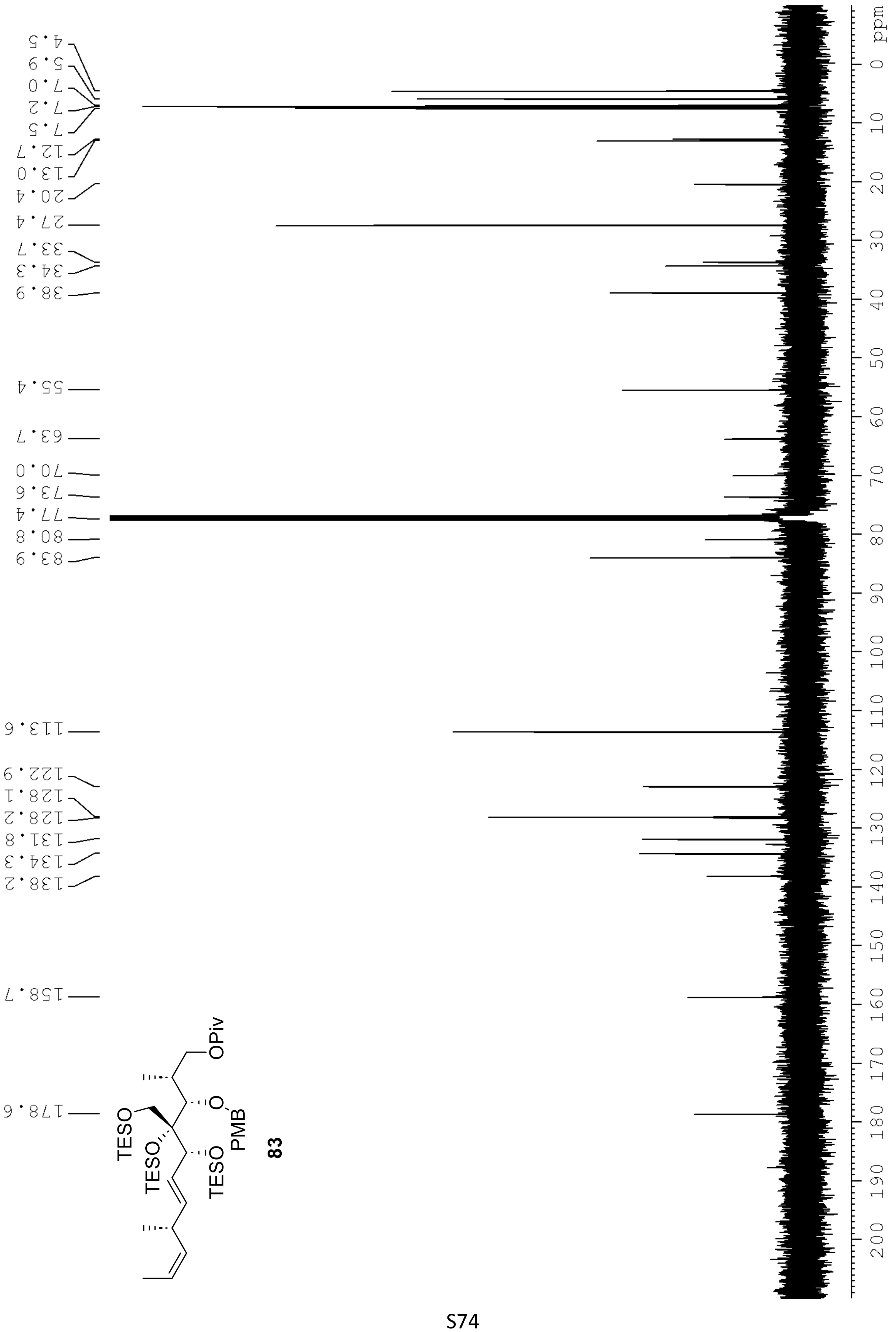

Alcohol **84**

^{1}H-NMR (400 MHz, $CDCl_3$)

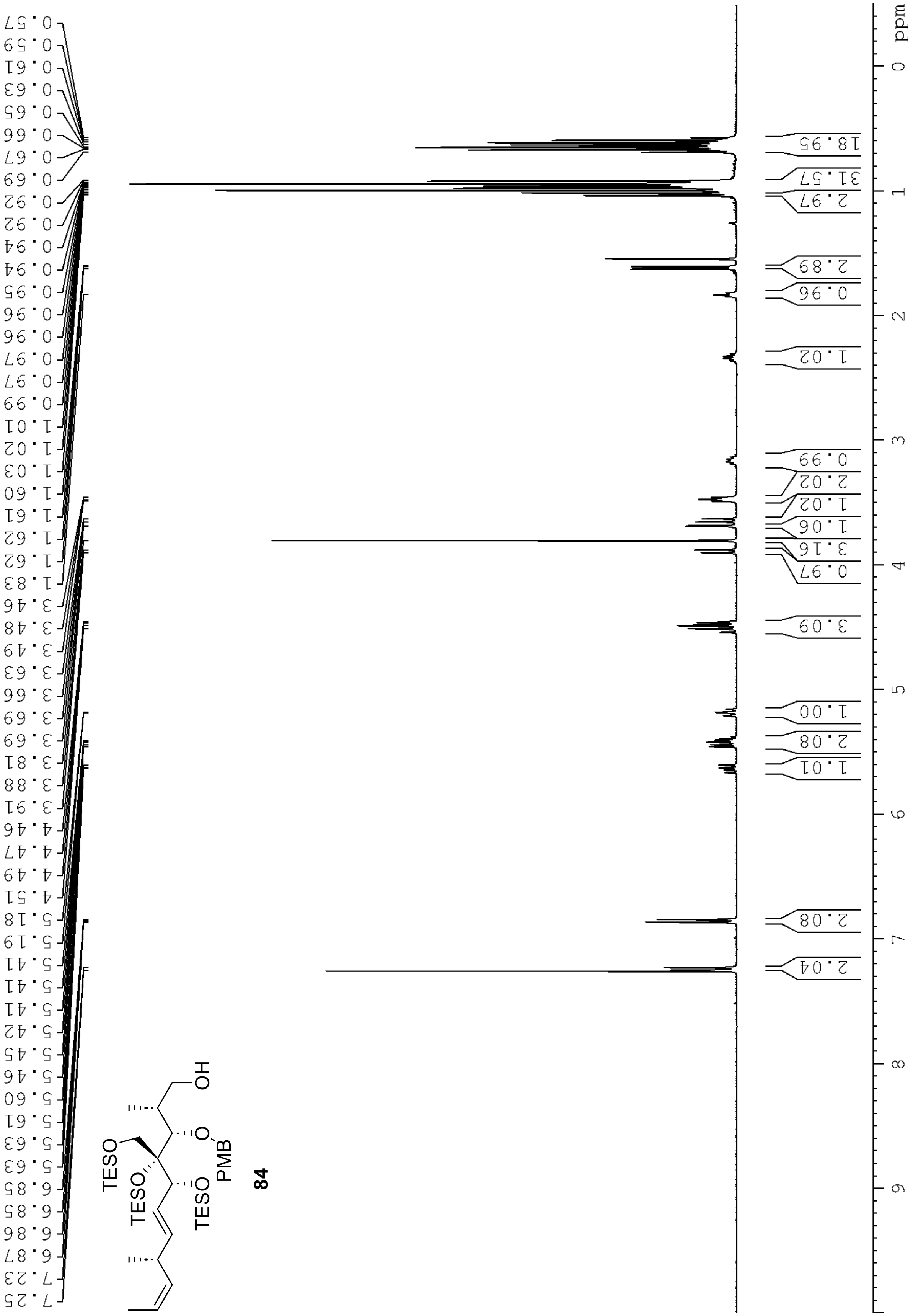

$^{13}C\{^{1}H\}$-NMR (100 MHz, $CDCl_3$)

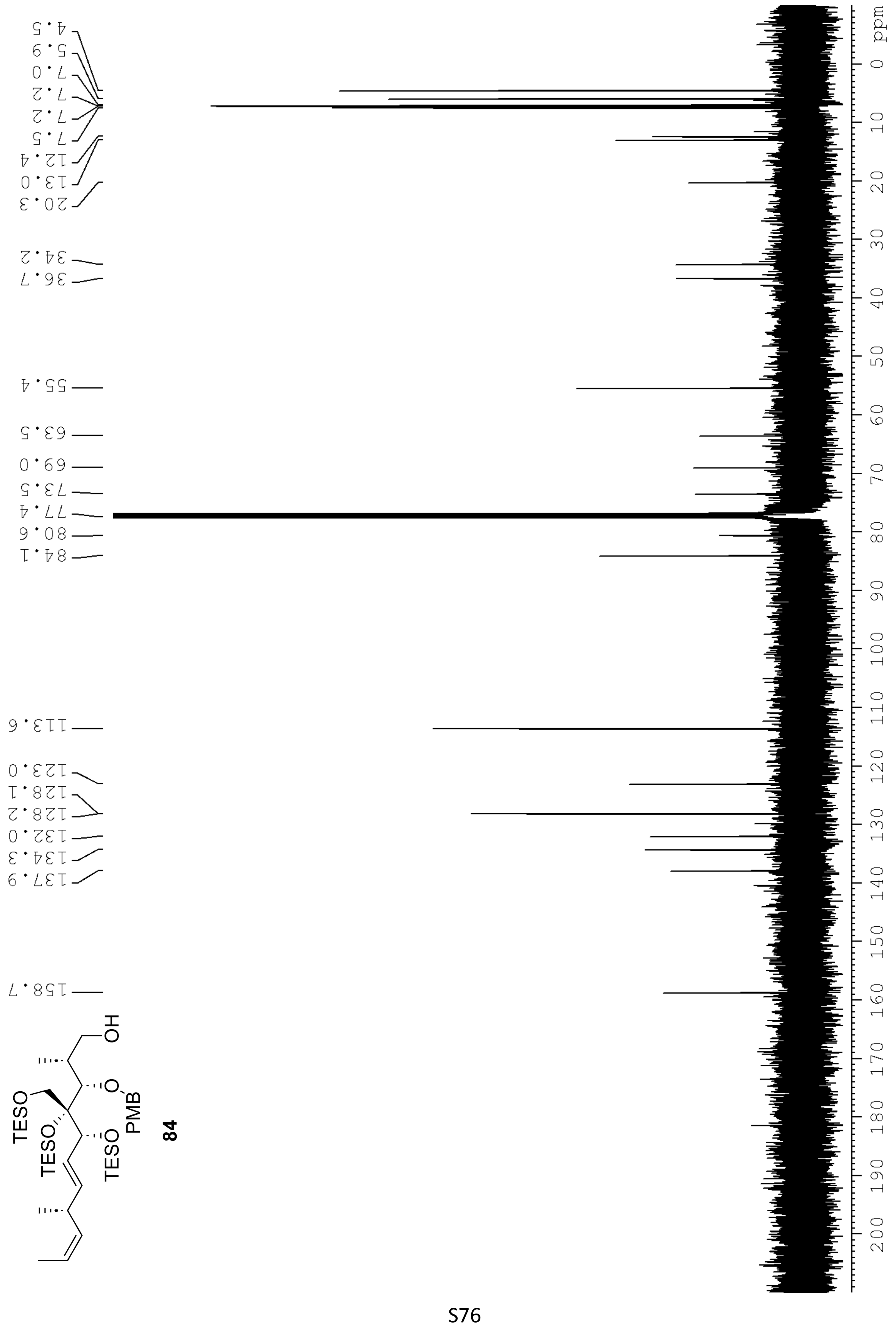

Alcohol **48**
^{1}H-NMR (400 MHz, $CDCl_3$)

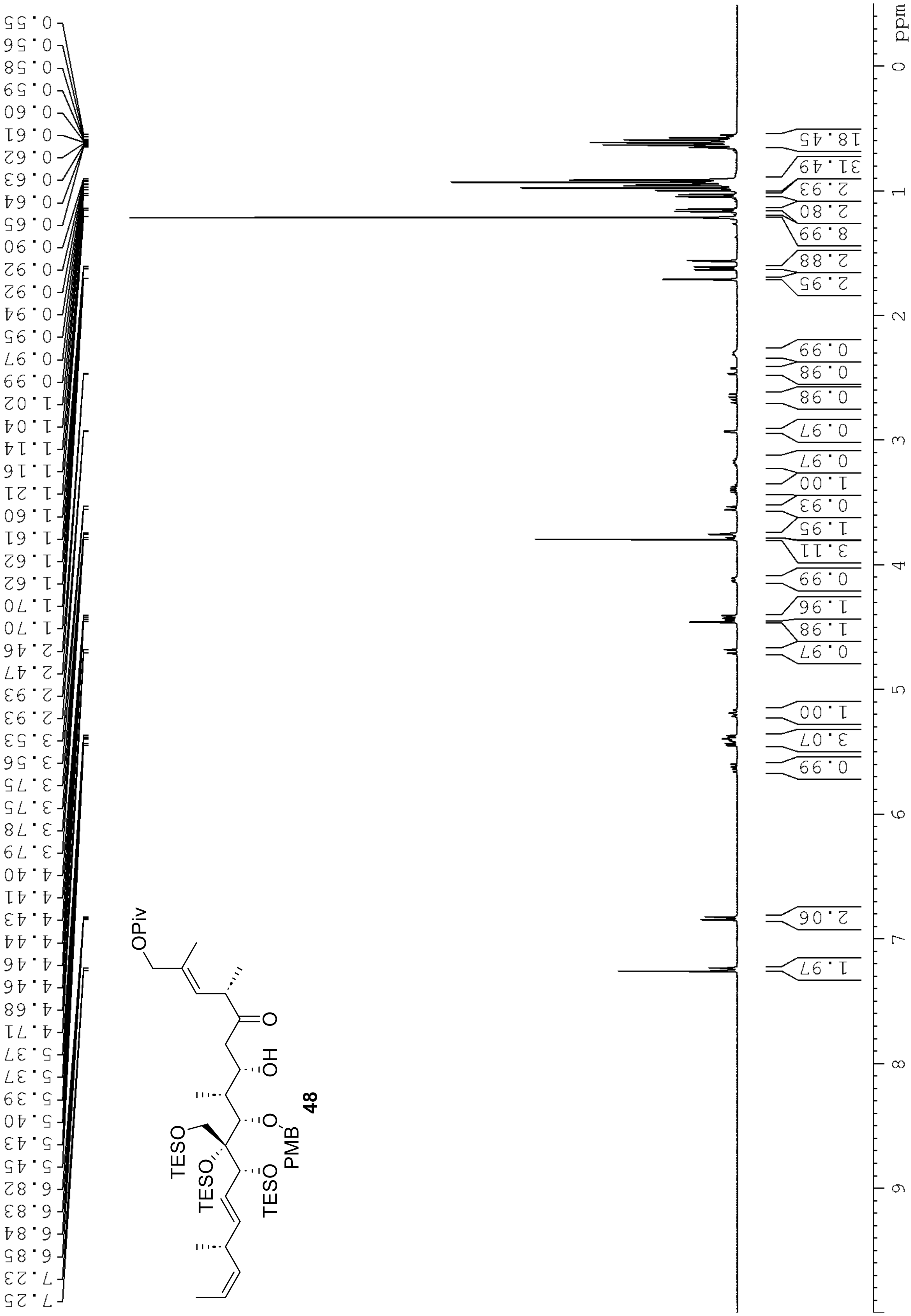

$^{13}C\{^1H\}$-NMR (100 MHz, $CDCl_3$)

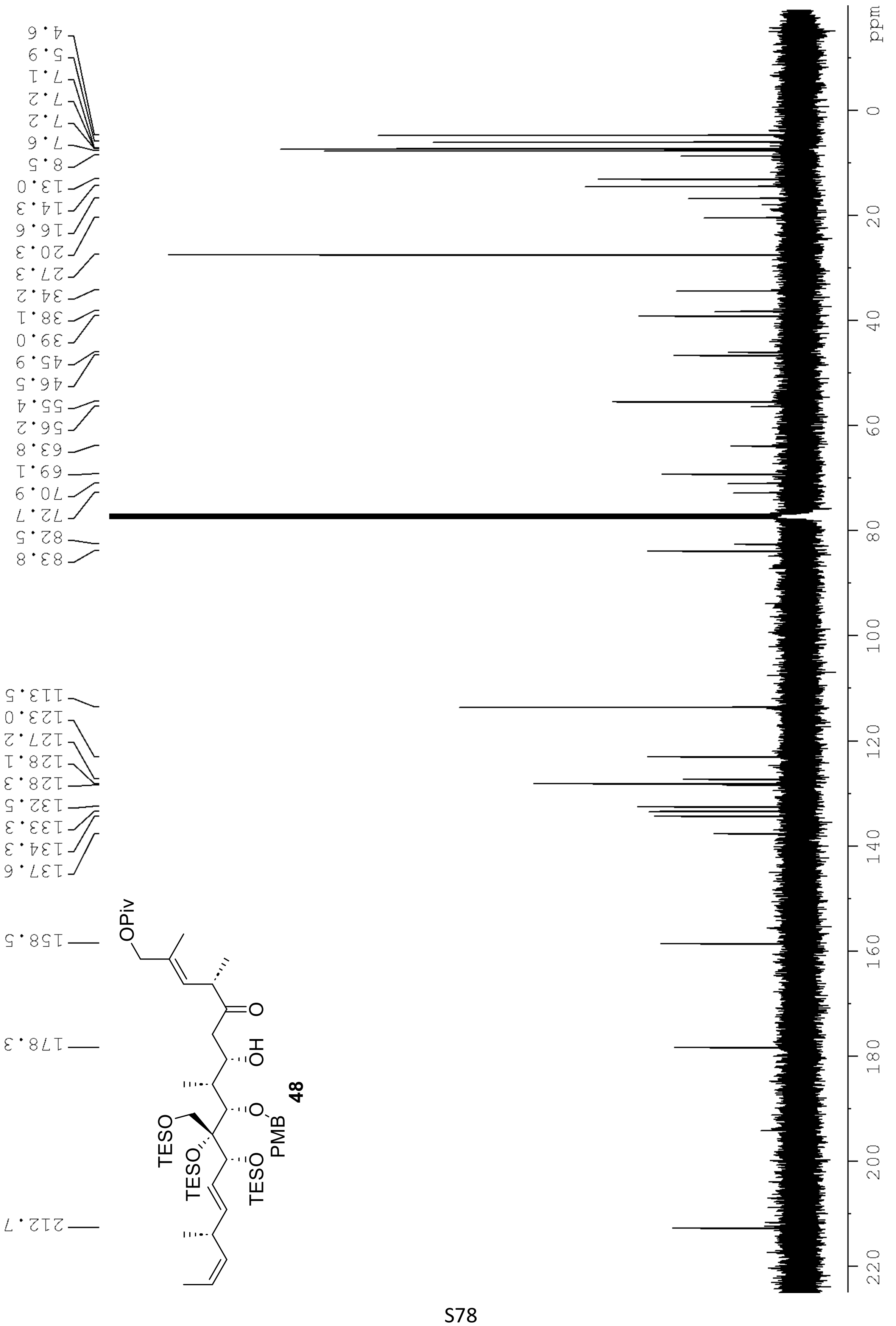

(*S*)-Mosher ester **S6**

^{1}H-NMR (400 MHz, C_6D_6)

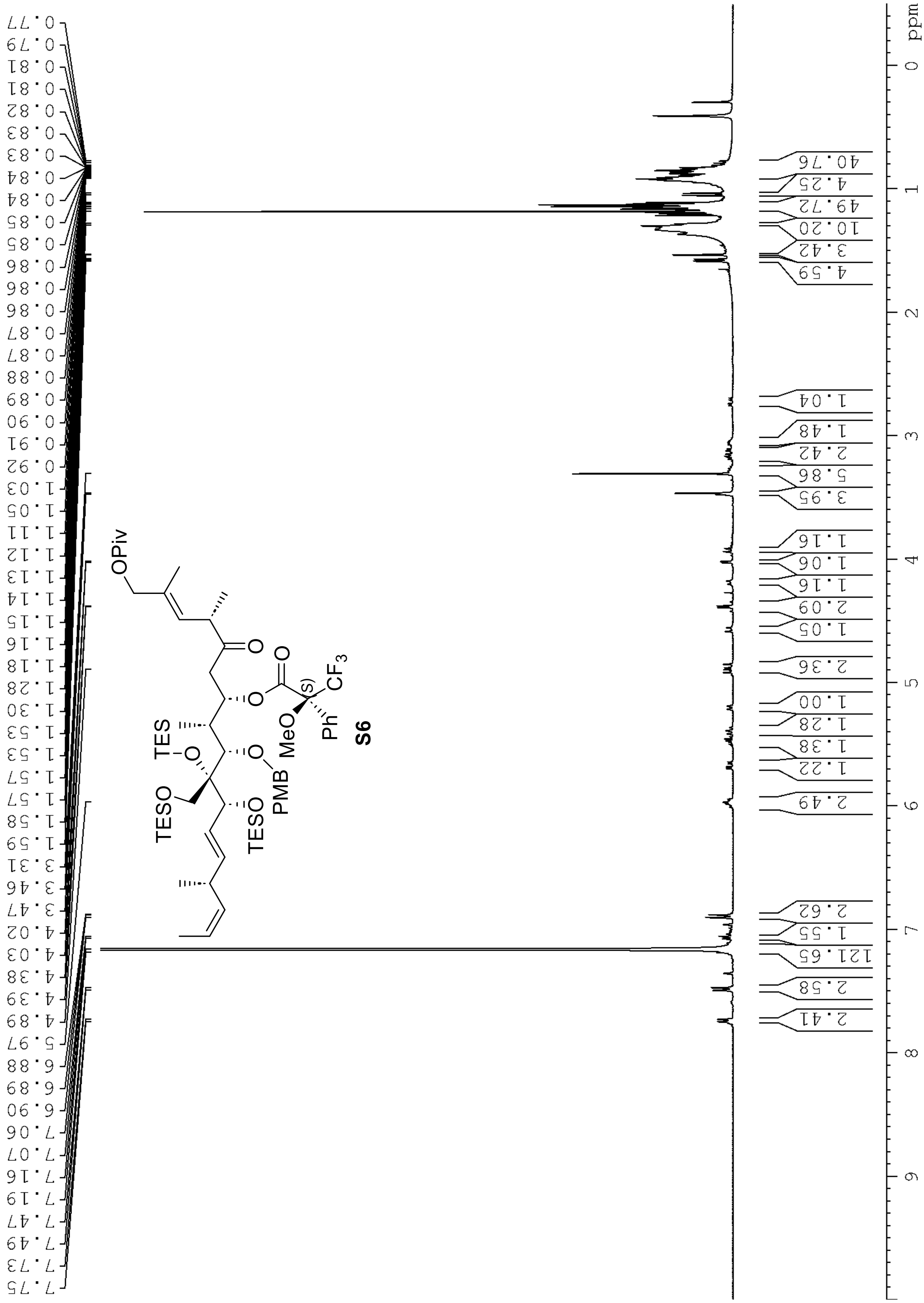

(*R*)-Mosher ester **S7**

^{1}H-NMR (400 MHz, C_6D_6)

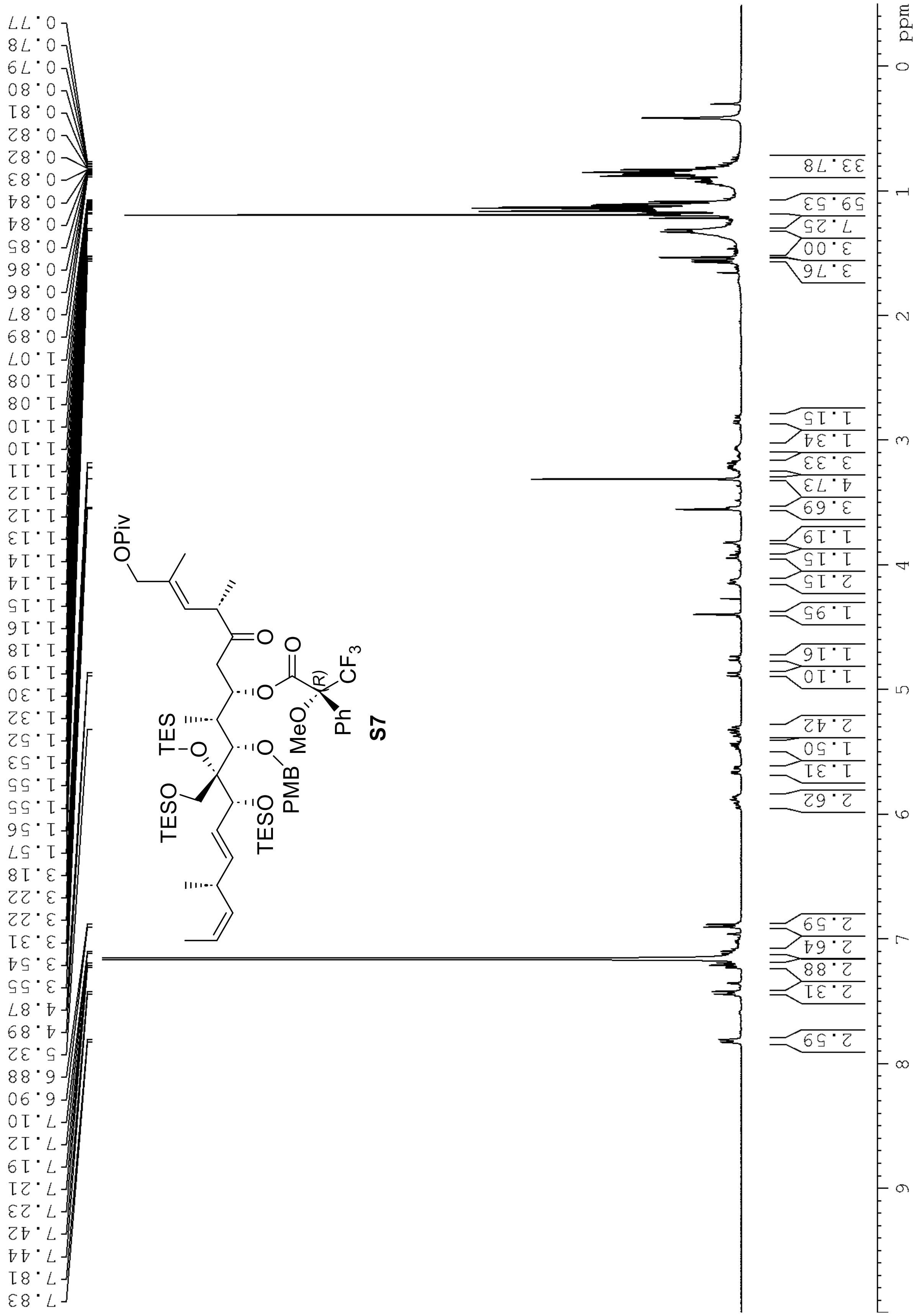

Diol **85**

^{1}H-NMR (400 MHz, $CDCl_3$)

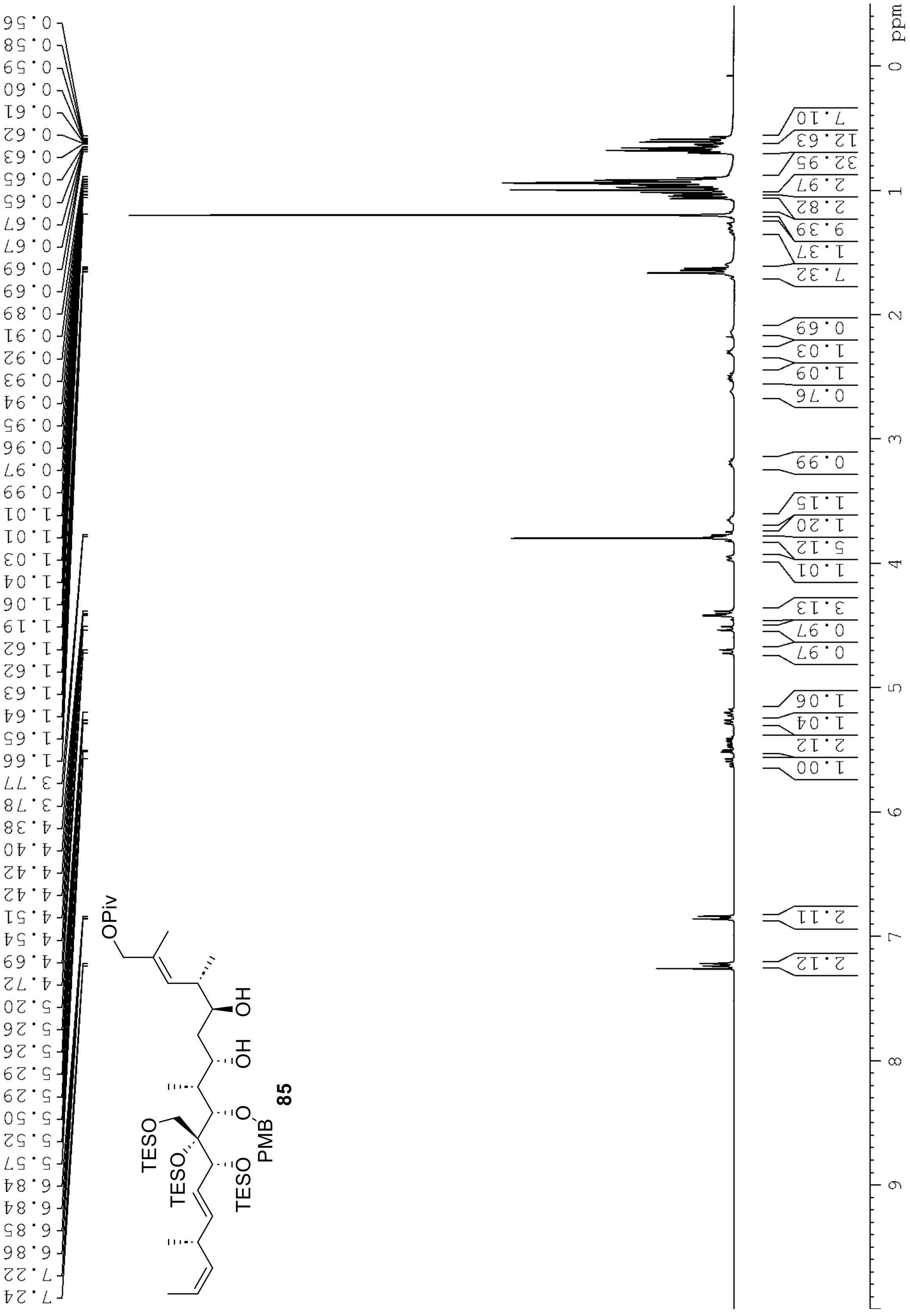

$^{13}C\{1H\}$-NMR (100 MHz, $CDCl_3$)

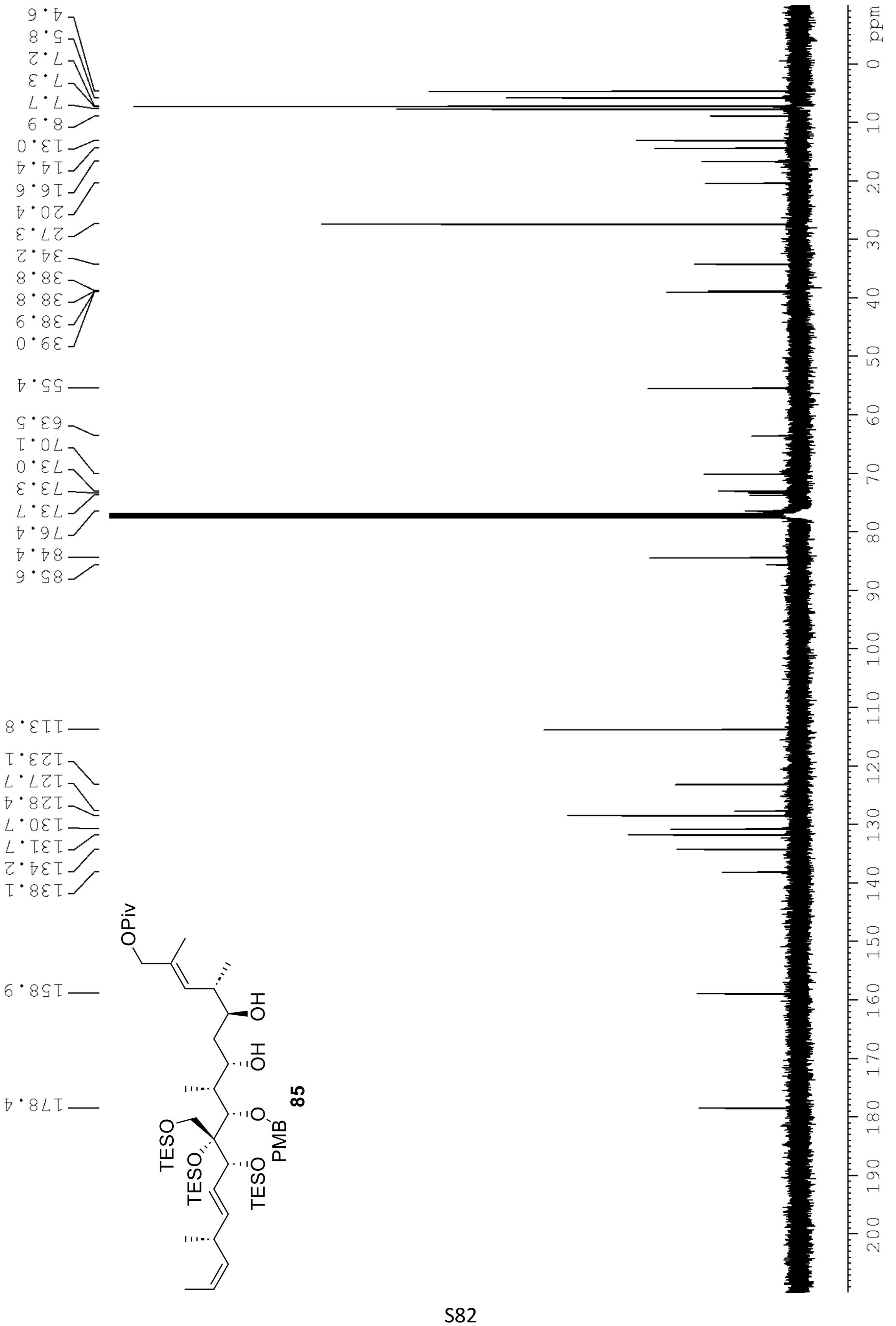

Acetonide **49**

^{1}H-NMR (400 MHz, $CDCl_3$)

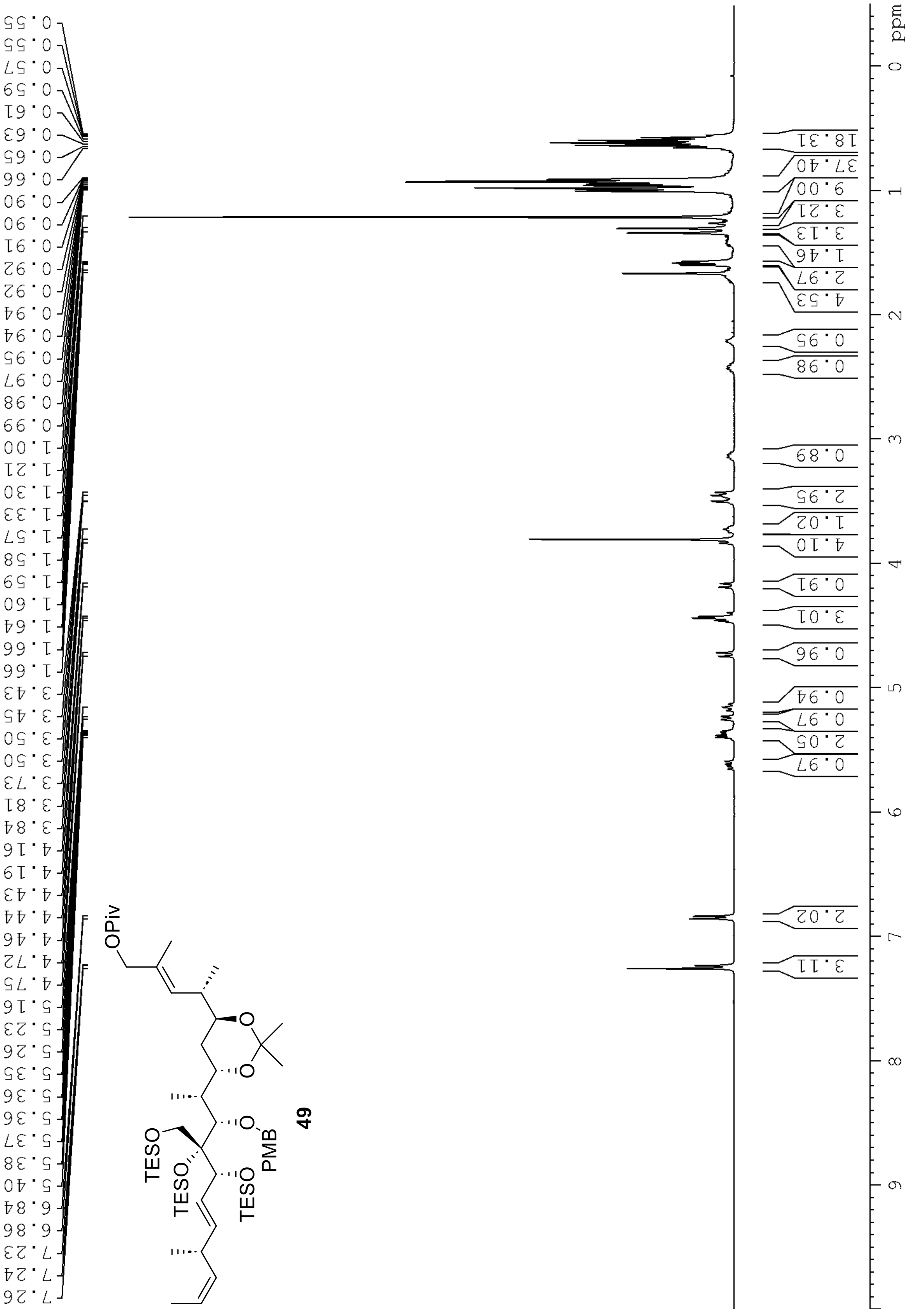

$^{13}C\{1H\}$-NMR (100 MHz, $CDCl_3$)

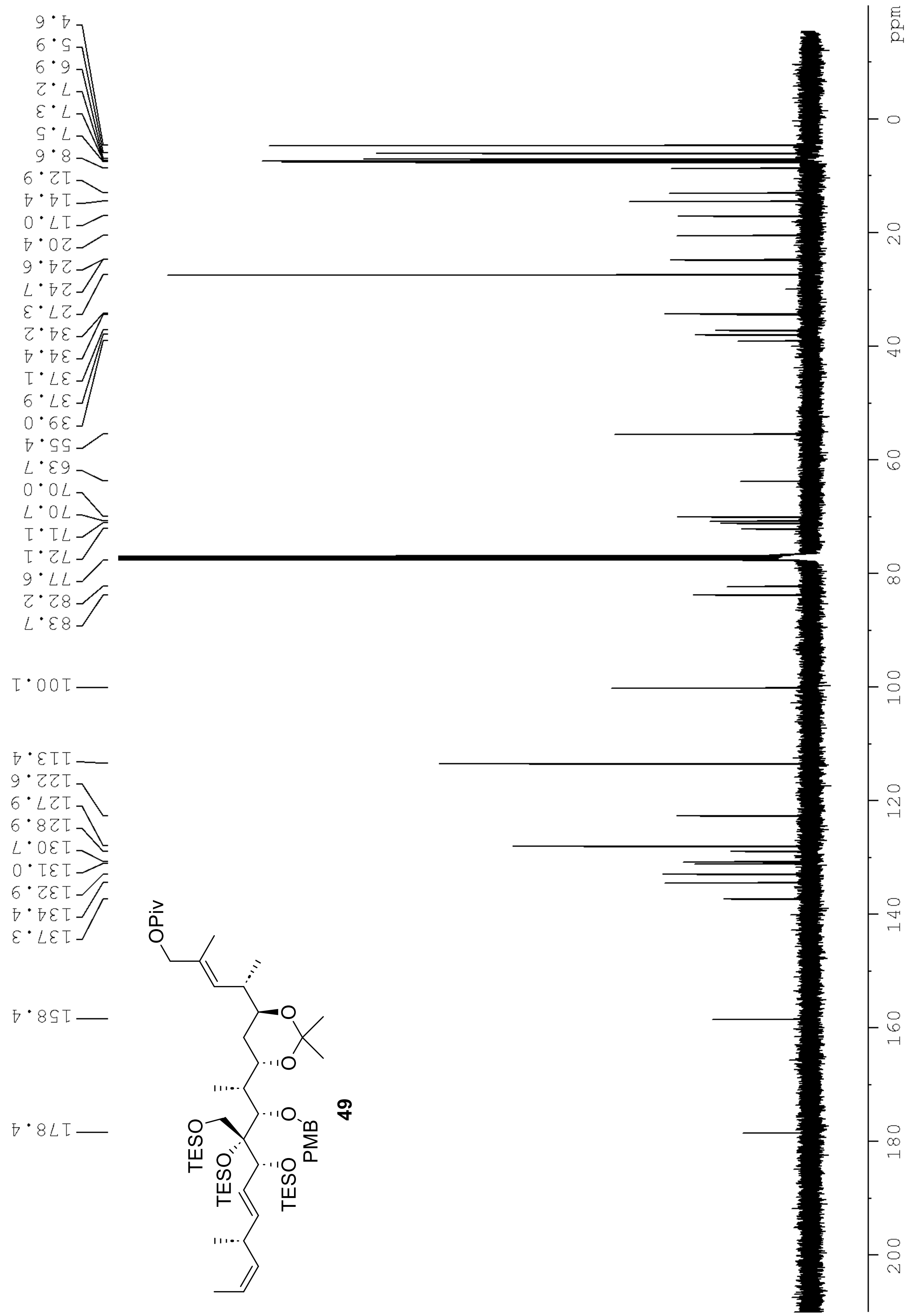

Alcohol **86**

^{1}H-NMR (400 MHz, $CDCl_3$)

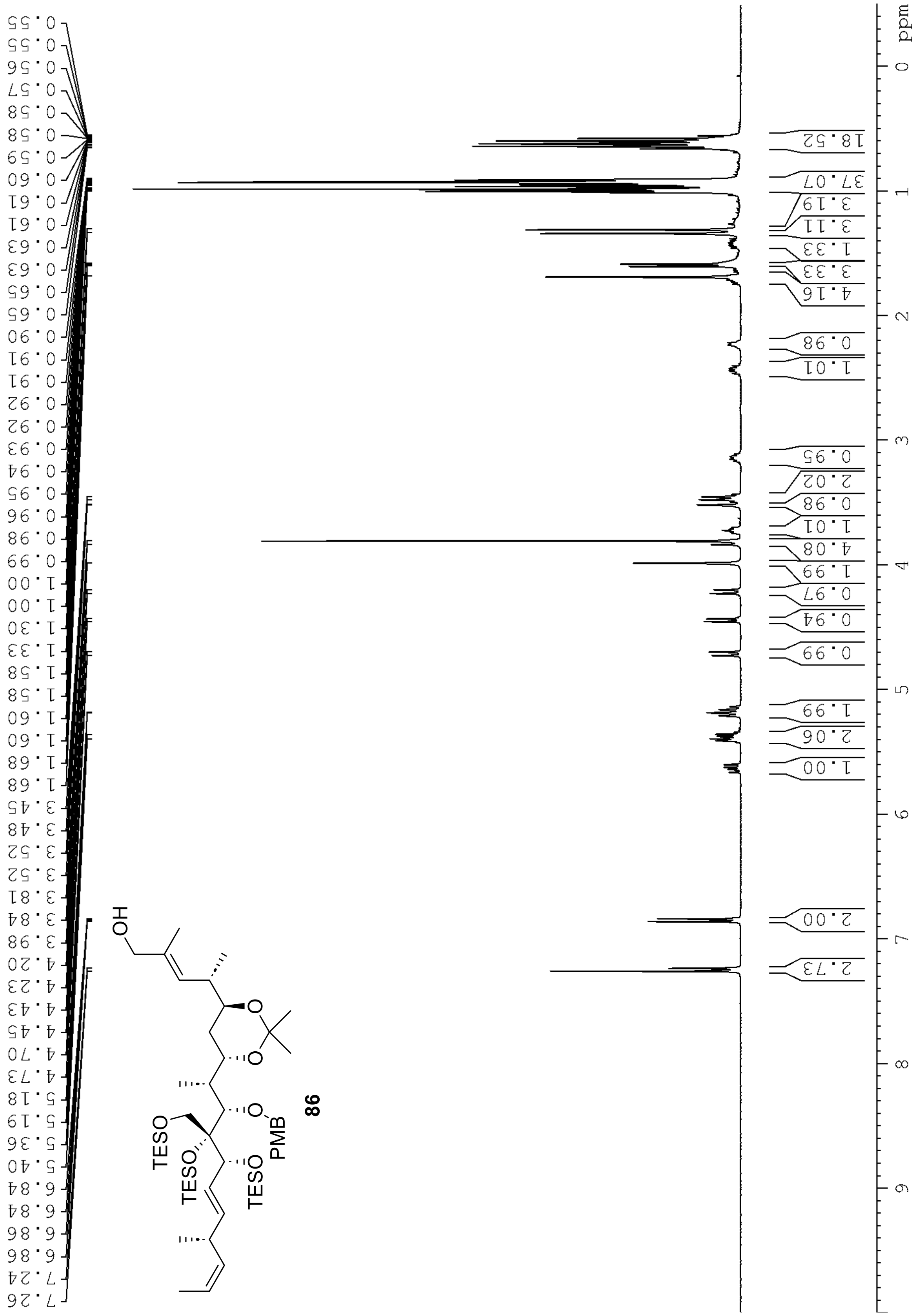

$^{13}C\{1H\}$-NMR (100 MHz, $CDCl_3$)

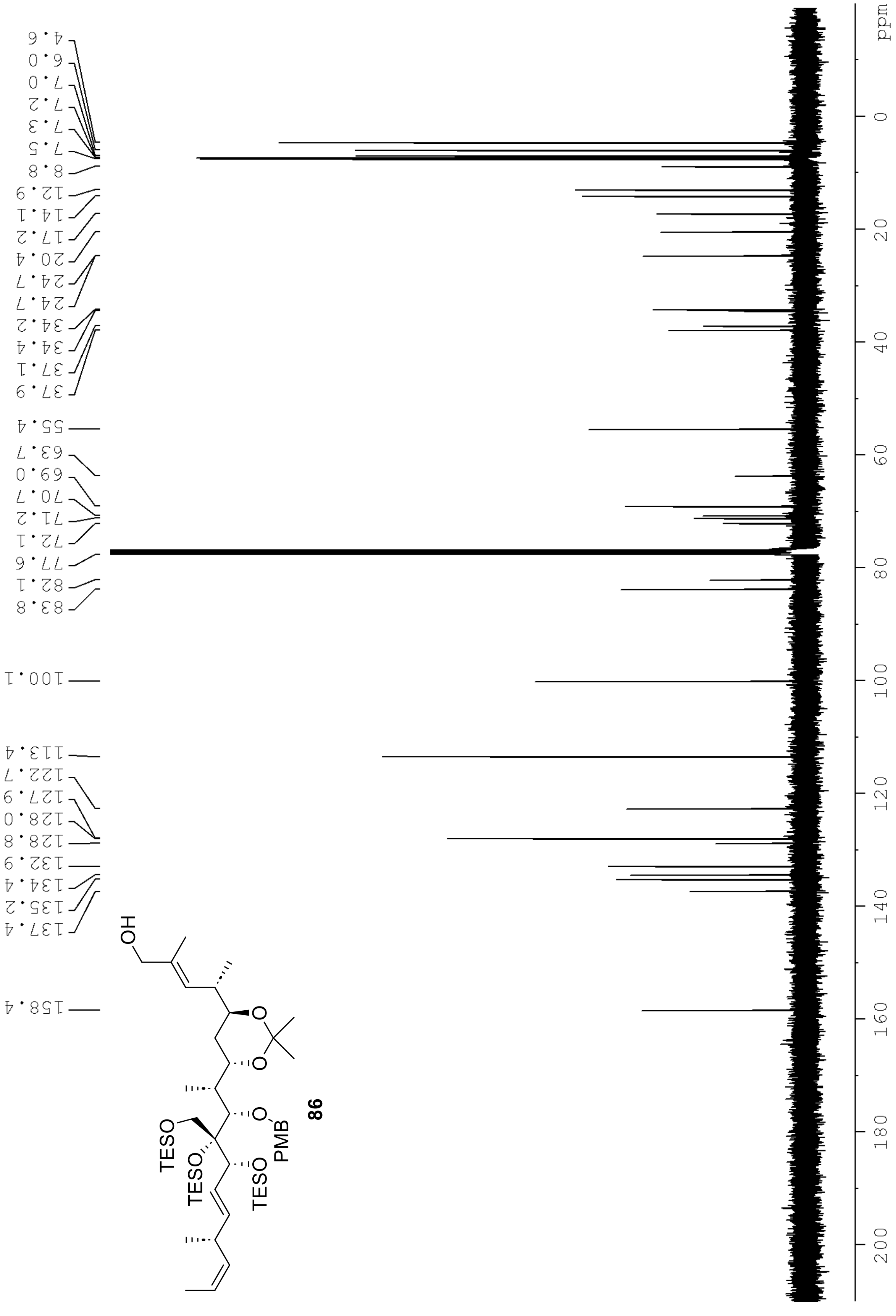

TIPS-ether **51**

^{1}H-NMR (400 MHz, $CDCl_3$)

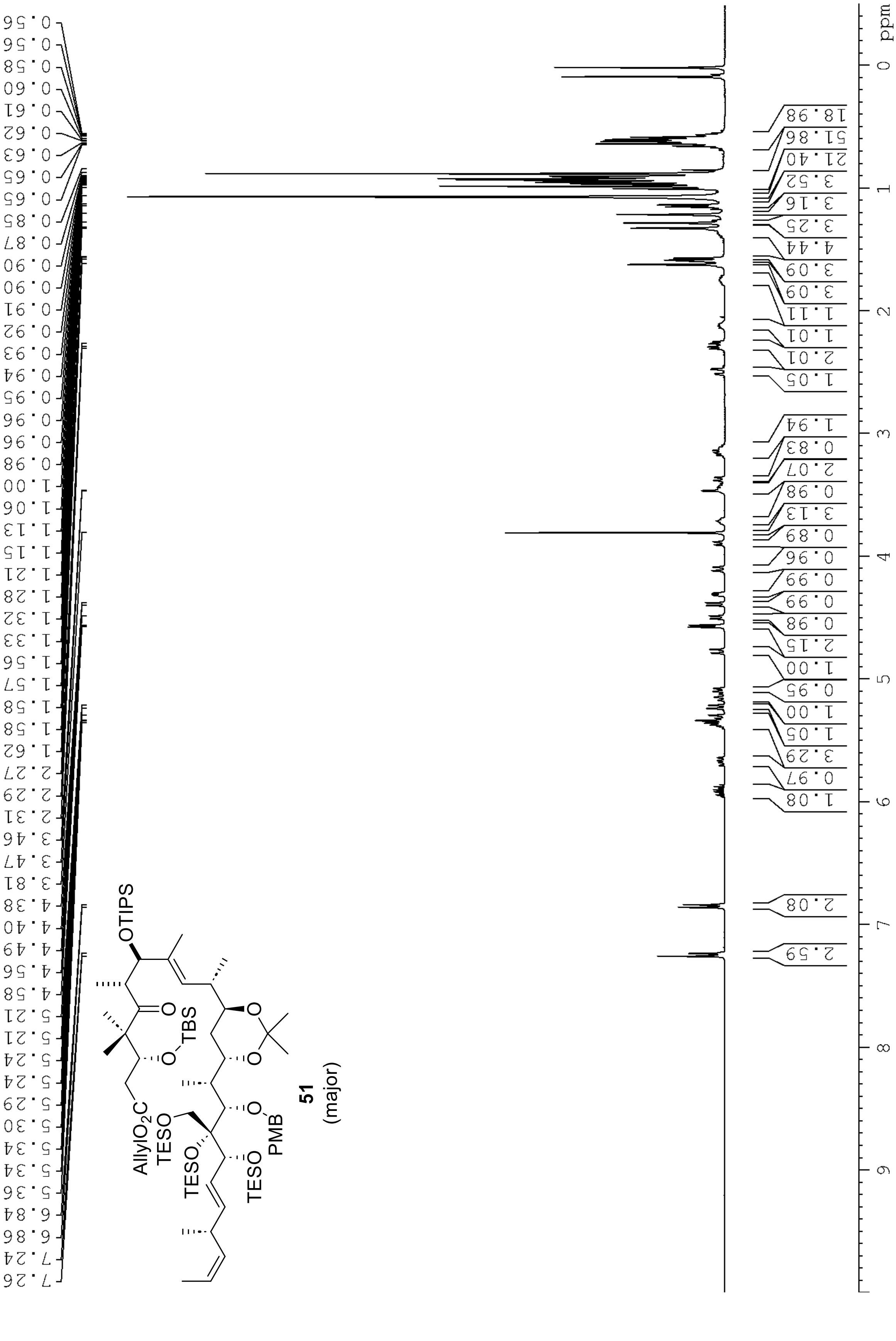

$^{13}C\{1H\}$-NMR (100 MHz, $CDCl_3$)

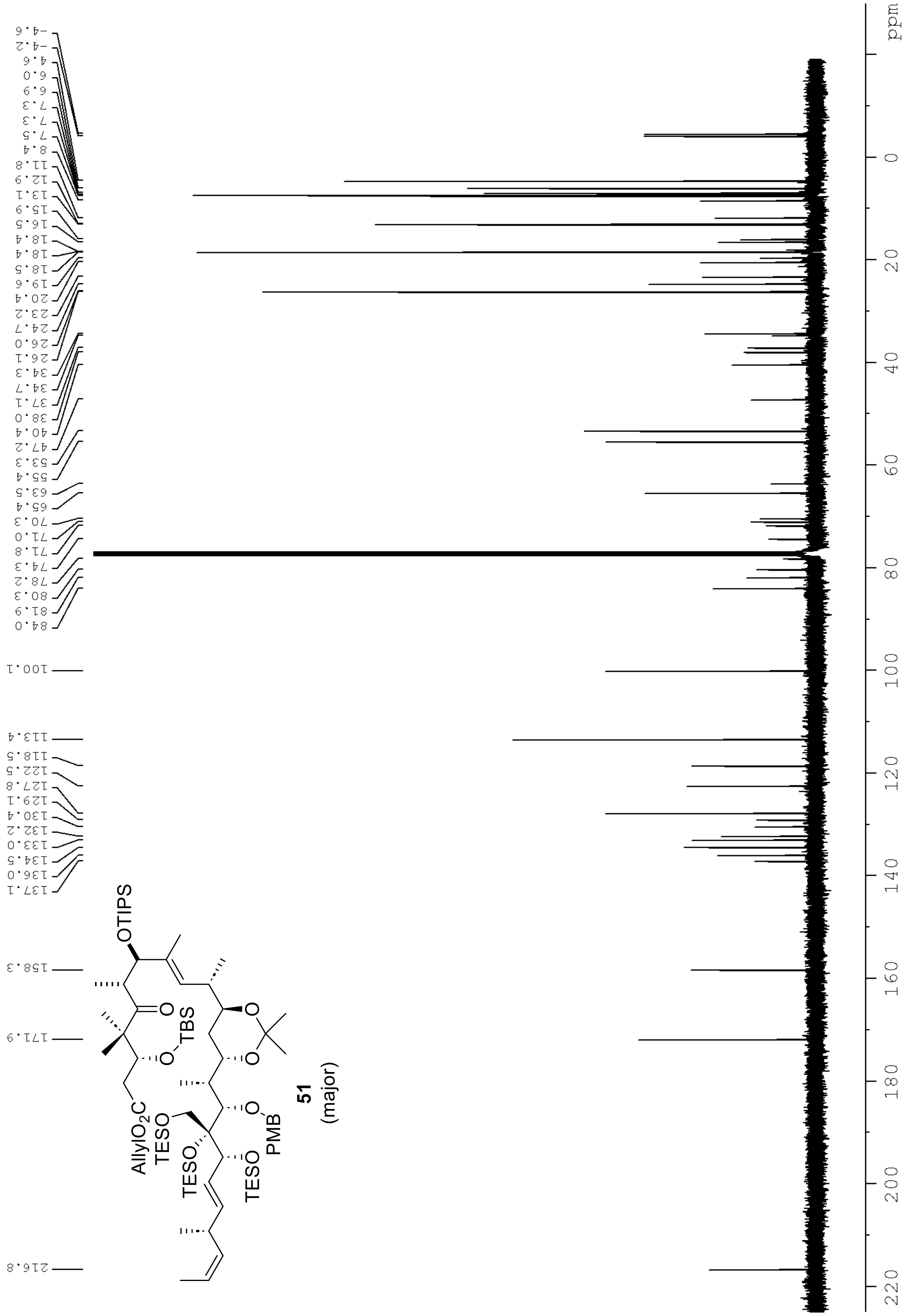

^{1}H-NMR (400 MHz, $CDCl_3$)

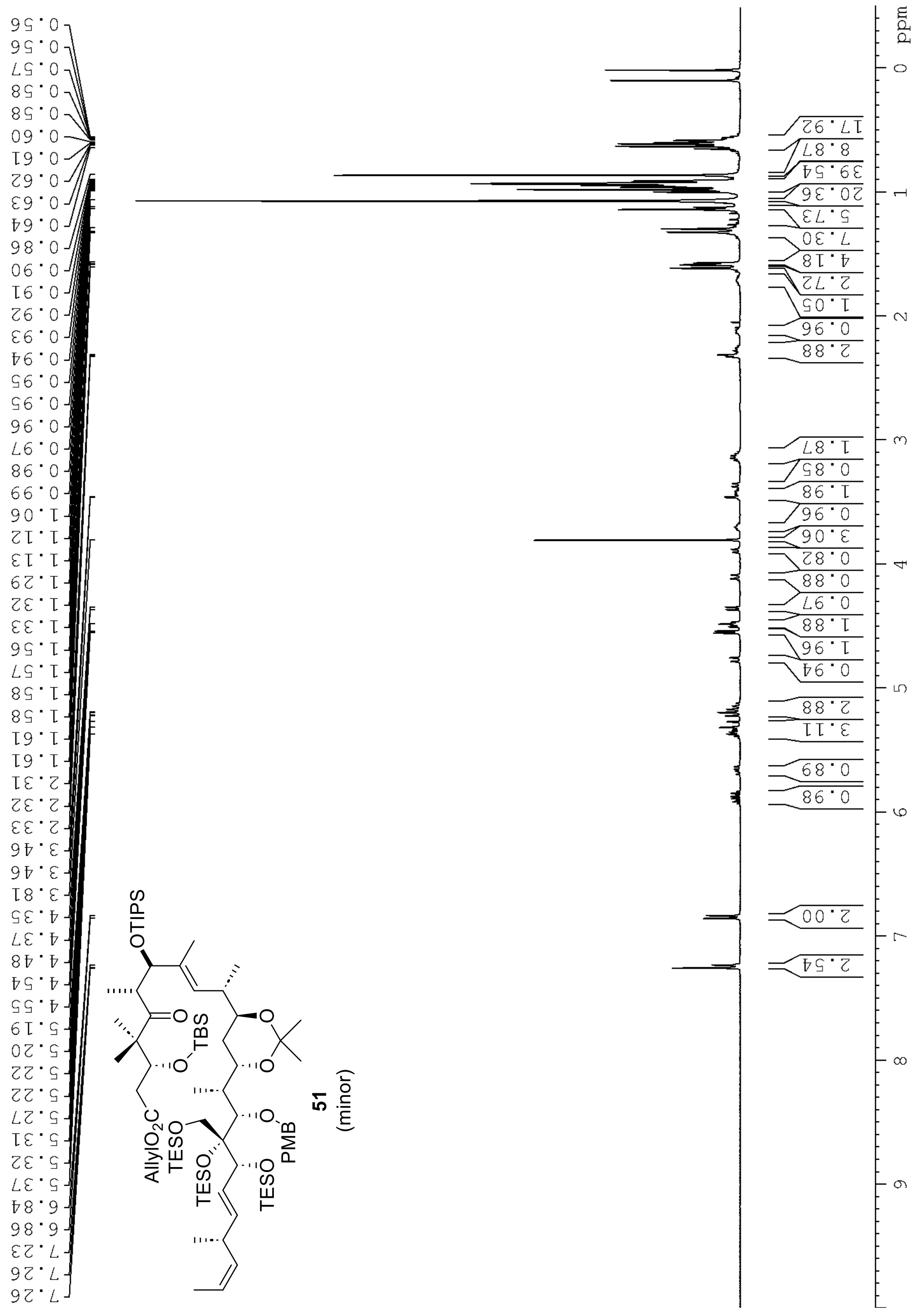

$^{13}C\{1H\}$-NMR (100 MHz, $CDCl_3$)

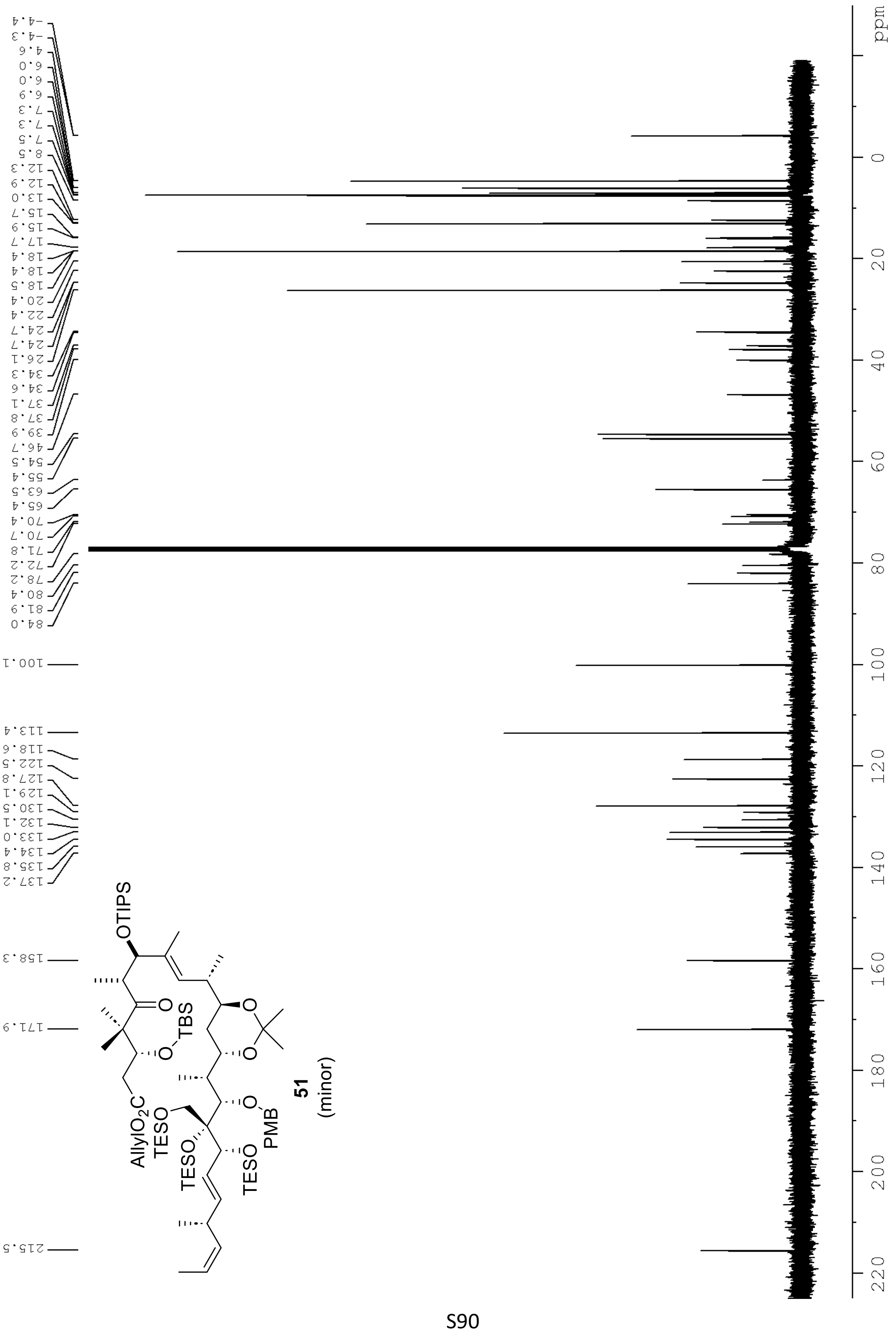

51
(minor)

PMP-acetal **53**

^{1}H-NMR (400 MHz, $CDCl_3$)

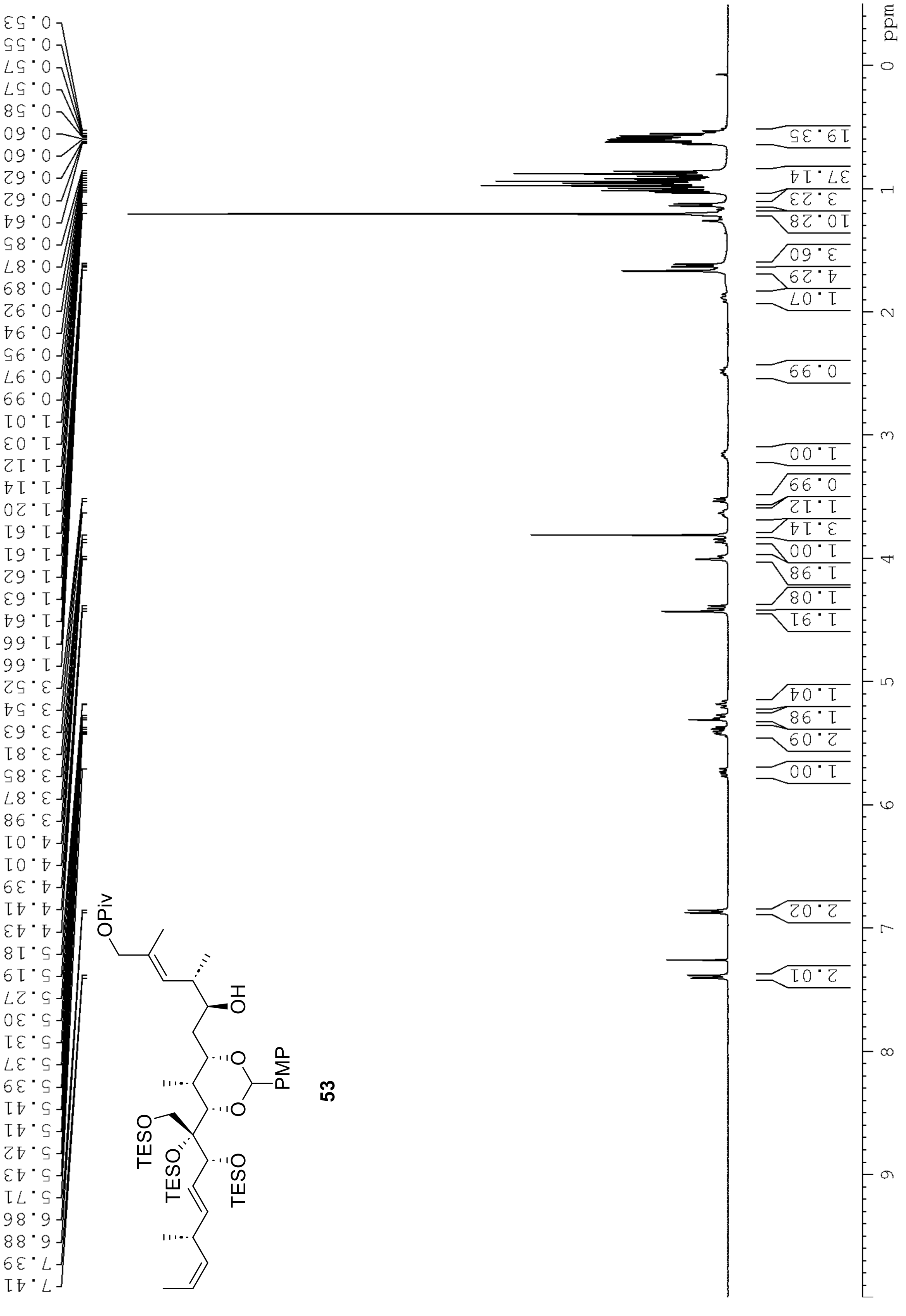

$^{13}C\{1H\}$-NMR (100 MHz, $CDCl_3$)

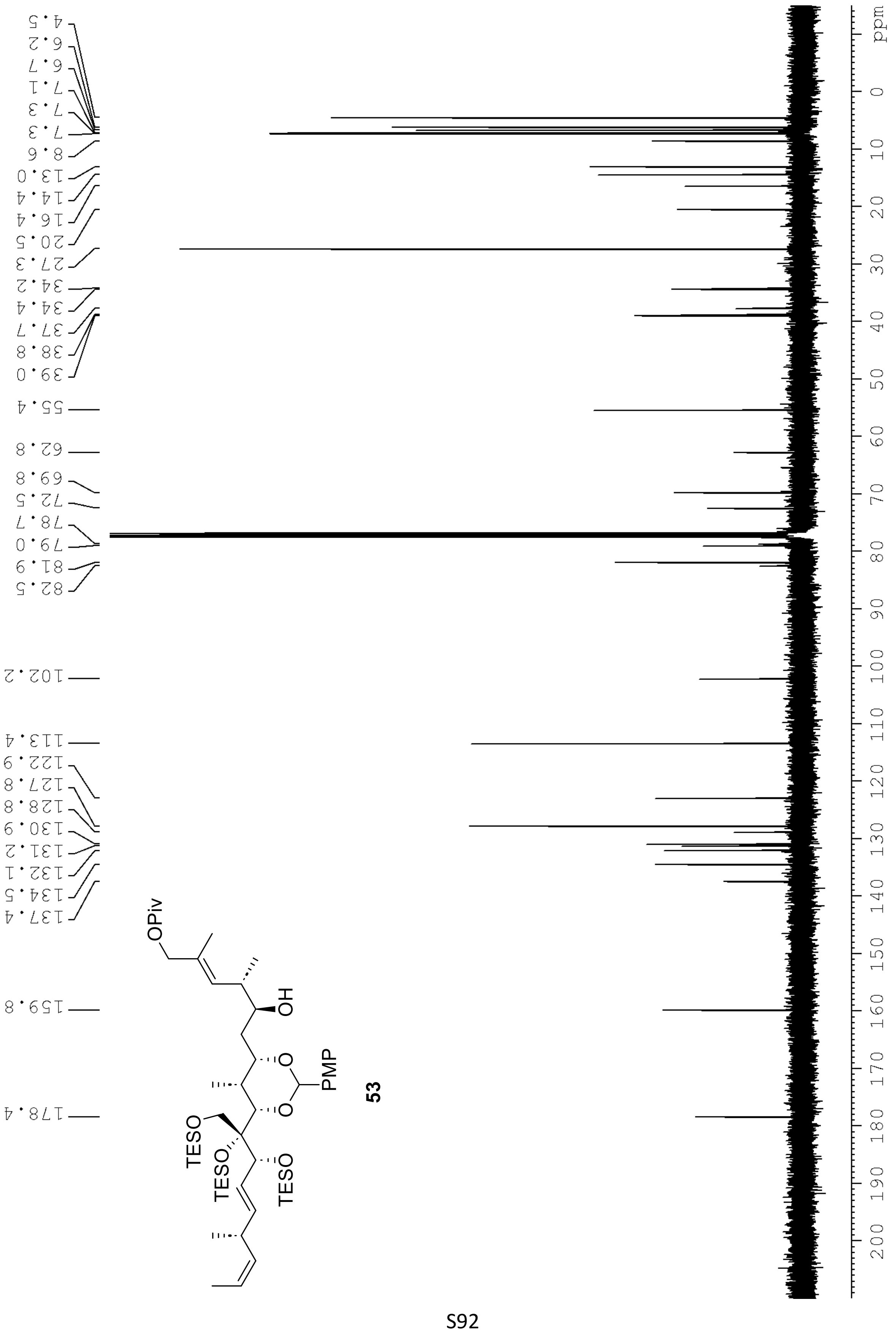

Pivalate **55a**

^{1}H-NMR (400 MHz, $CDCl_3$)

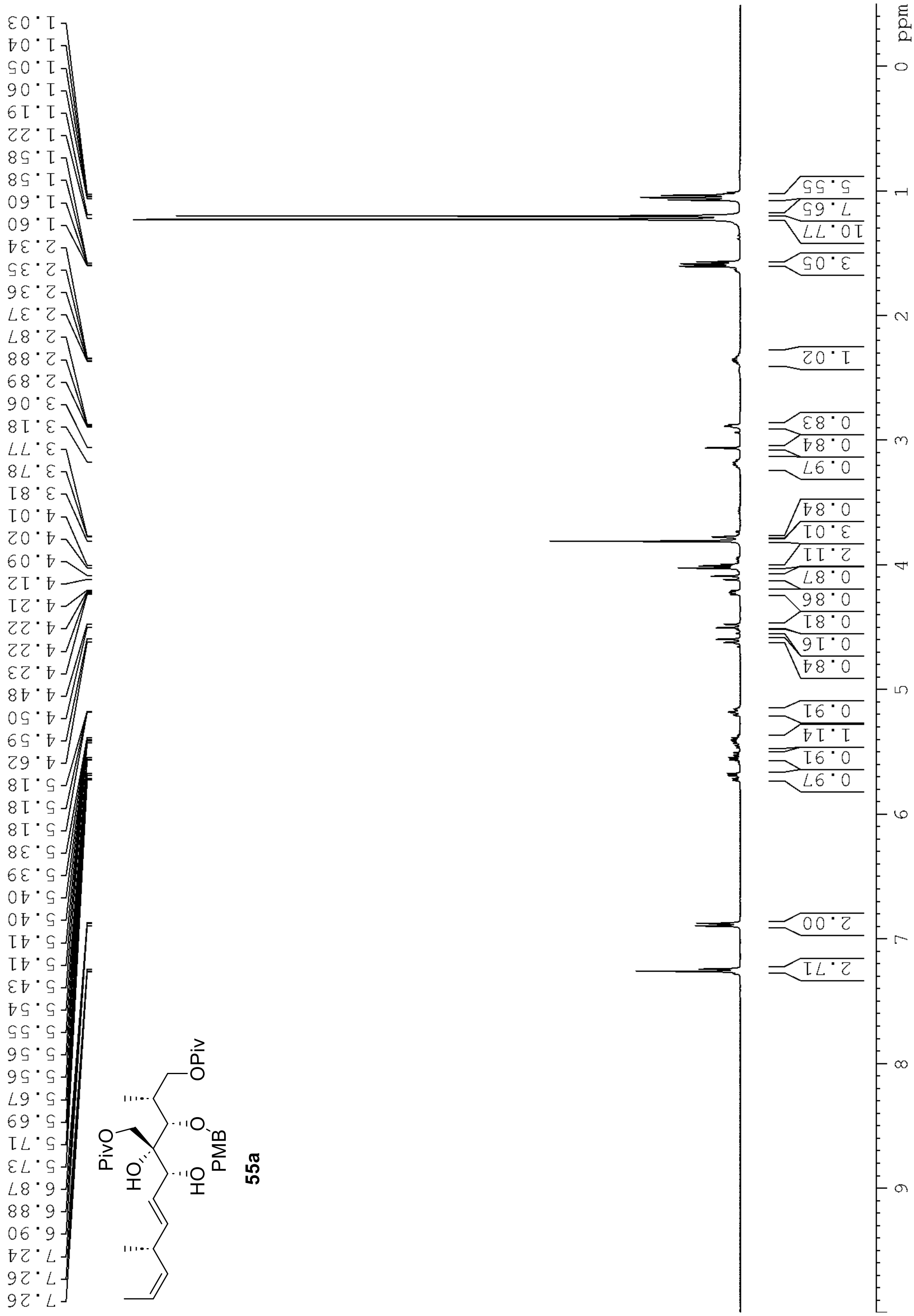

$^{13}C\{1H\}$-NMR (100 MHz, $CDCl_3$)

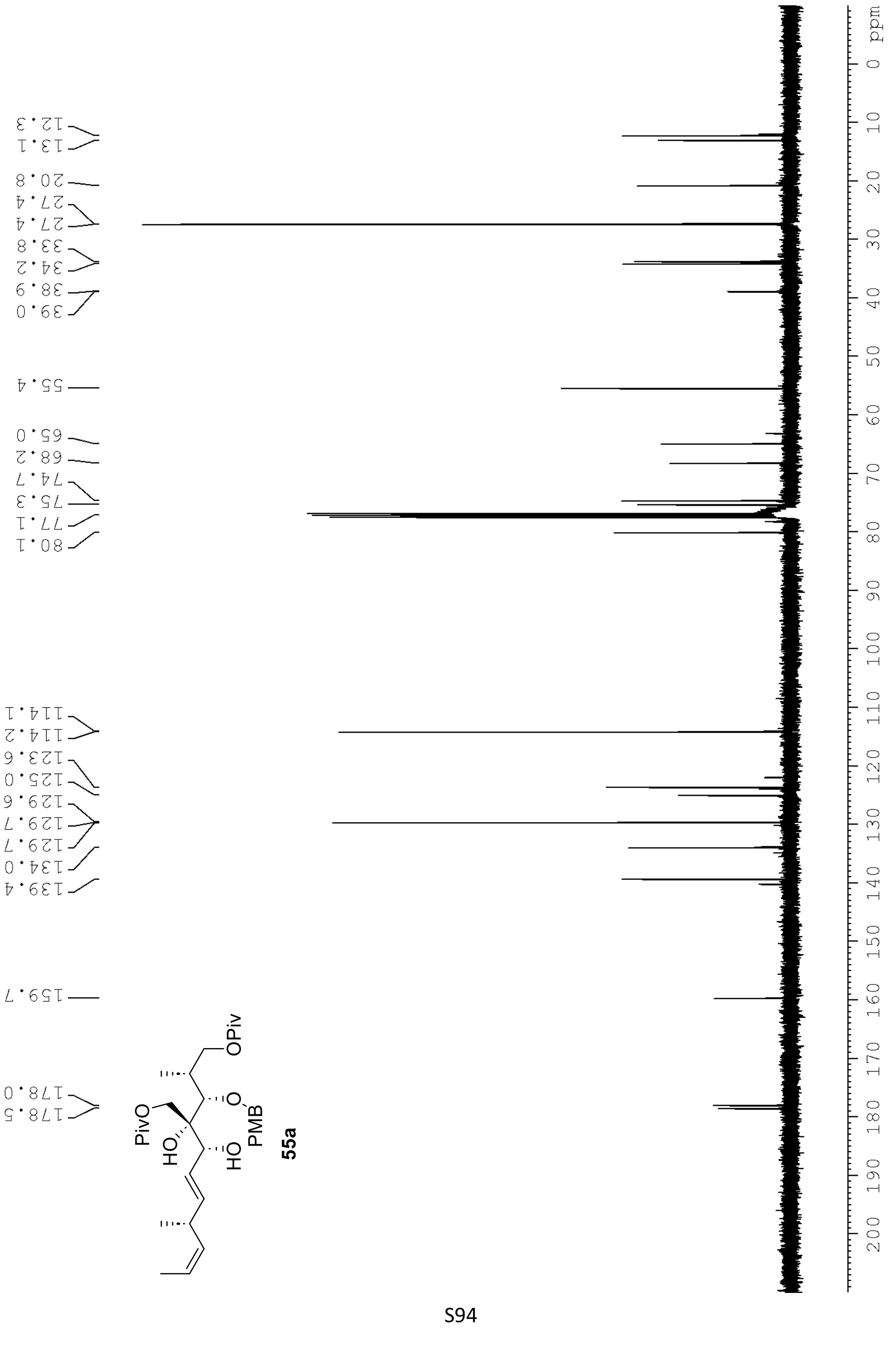

TES-ether **88a**

^{1}H-NMR (400 MHz, $CDCl_3$)

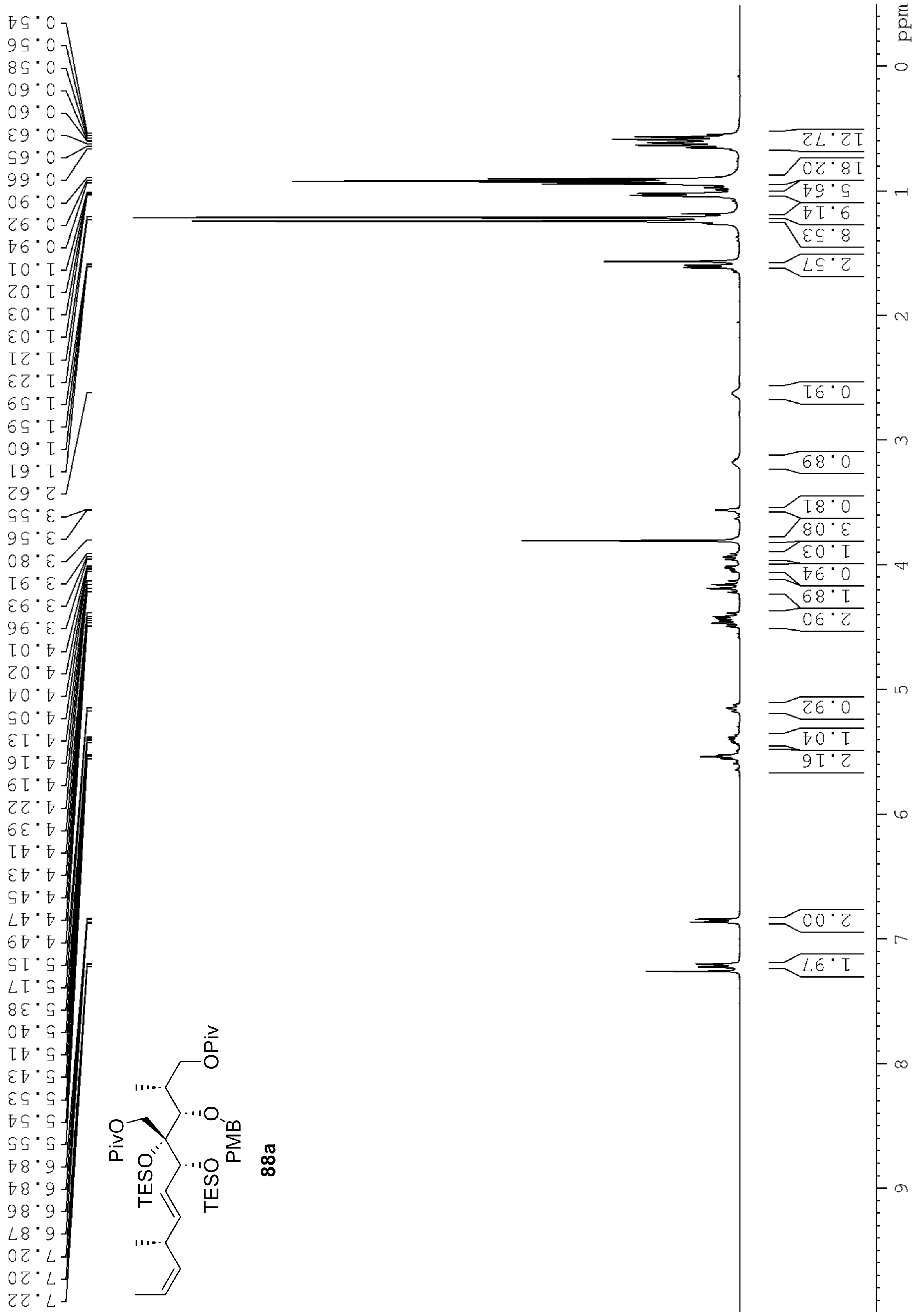

$^{13}C\{1H\}$-NMR (100 MHz, $CDCl_3$)

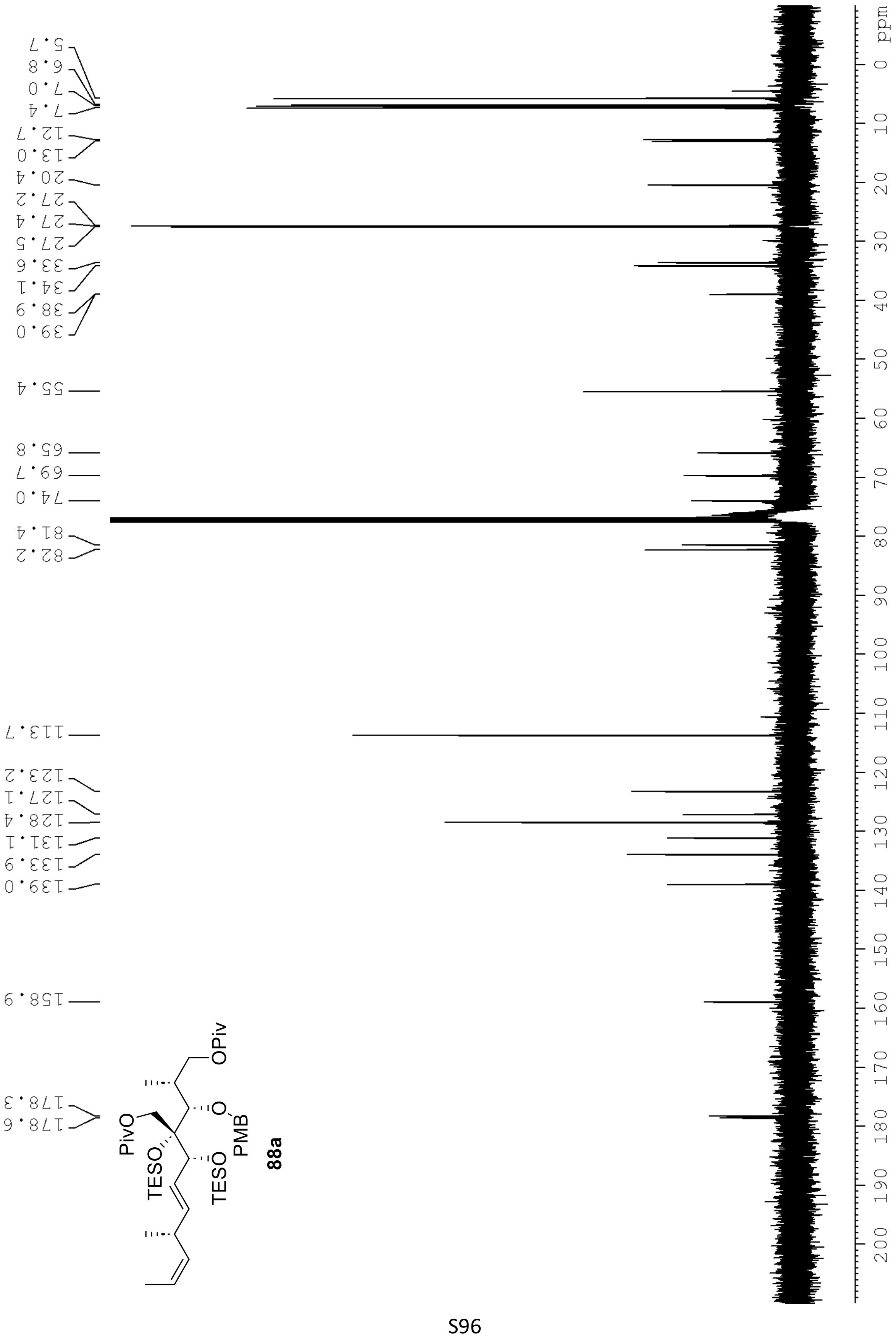

Diol **89a**

^{1}H-NMR (500 MHz, $CDCl_3$)

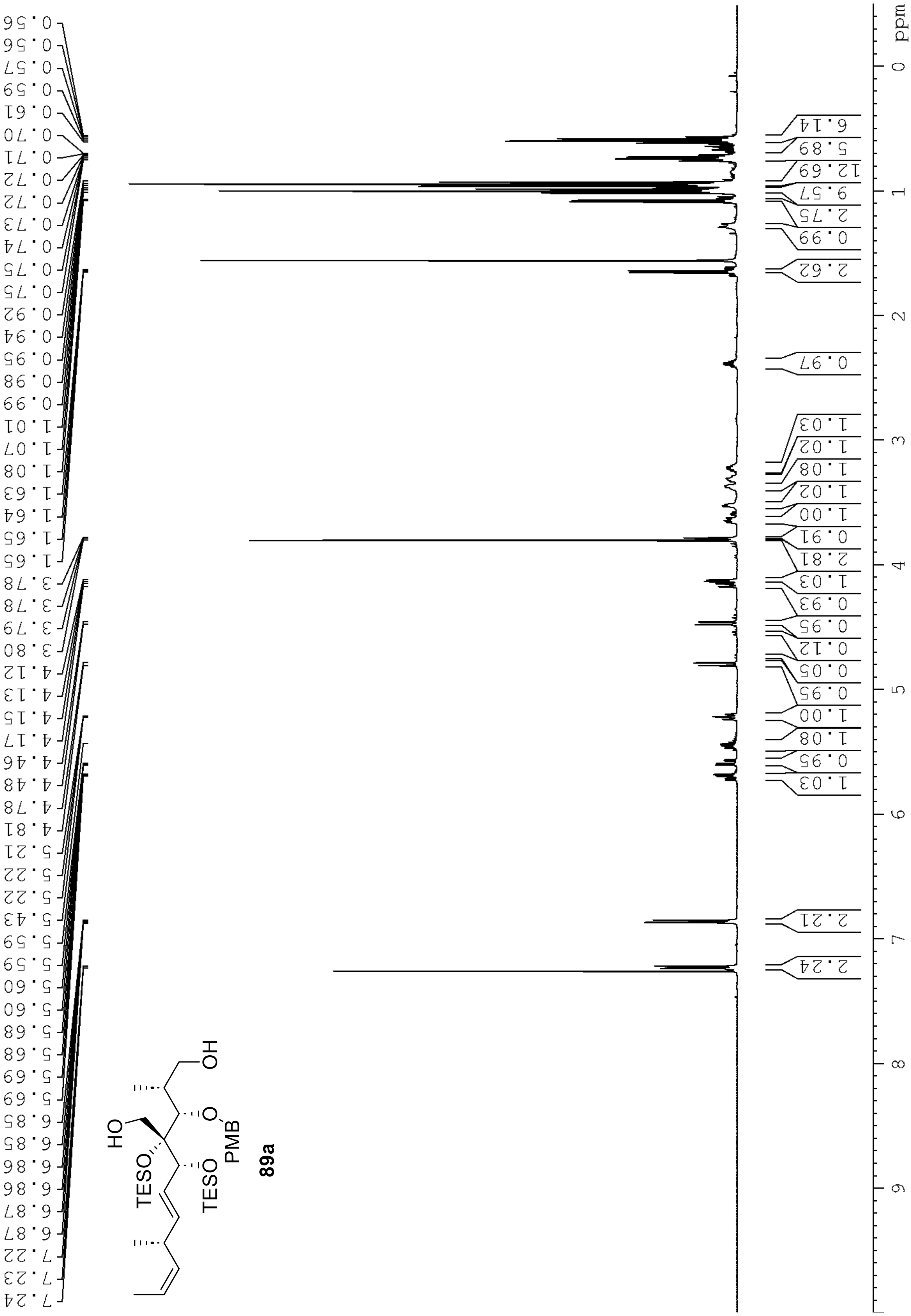

$^{13}C\{1H\}$-NMR (125 MHz, $CDCl_3$)

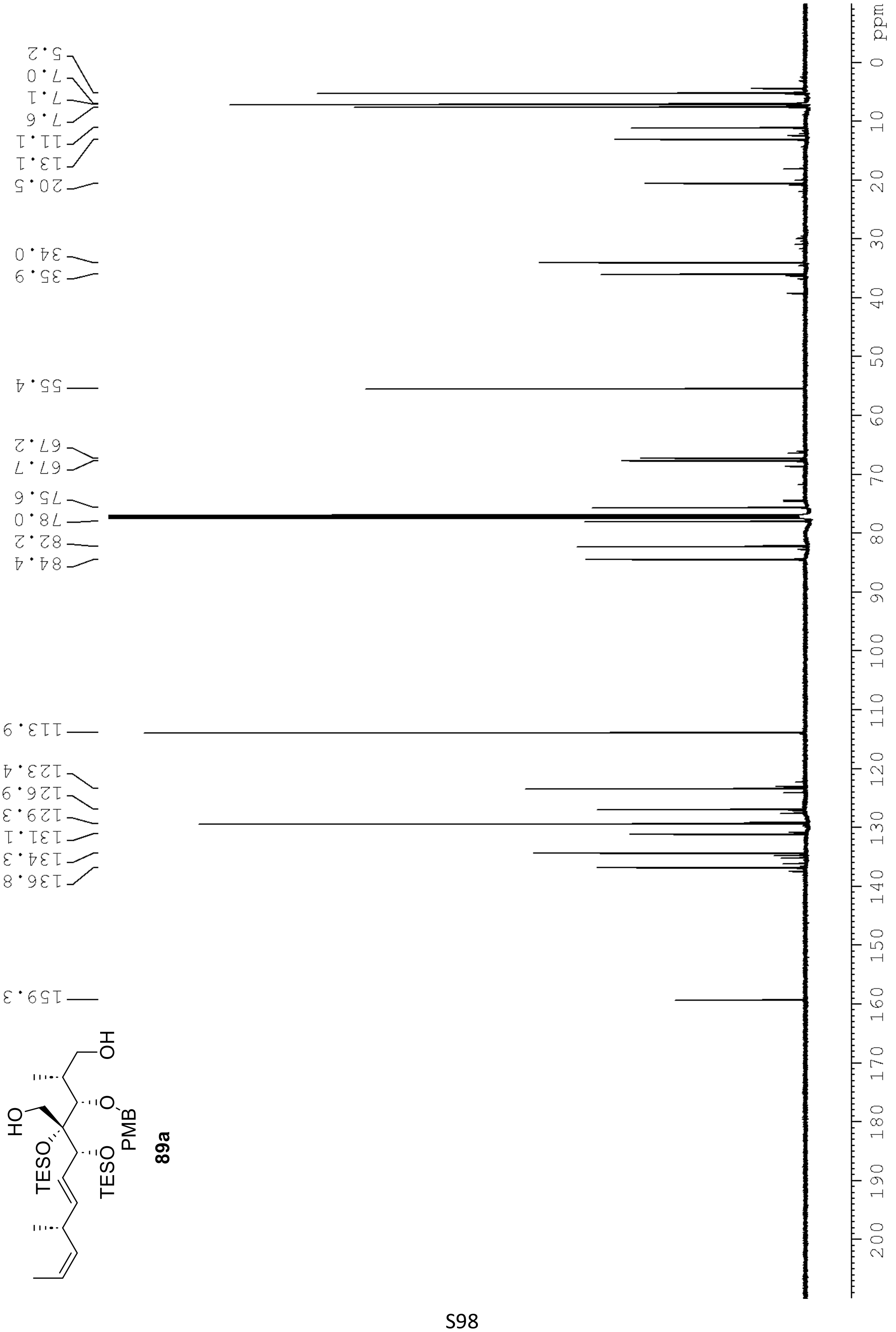

PMP-acetal **90**

^{1}H-NMR (400 MHz, $CDCl_3$)

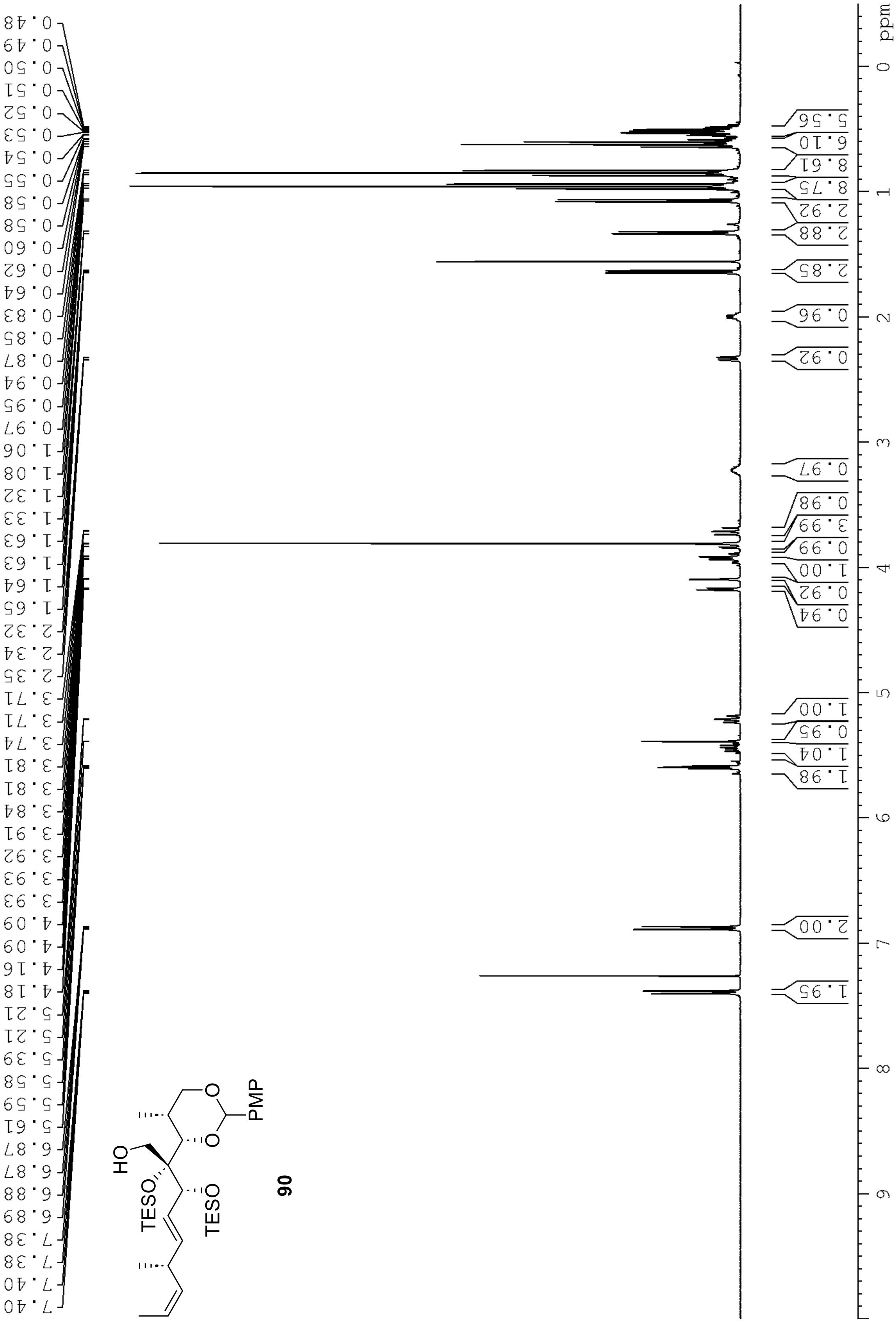

$^{13}C\{1H\}$-NMR (100 MHz, $CDCl_3$)

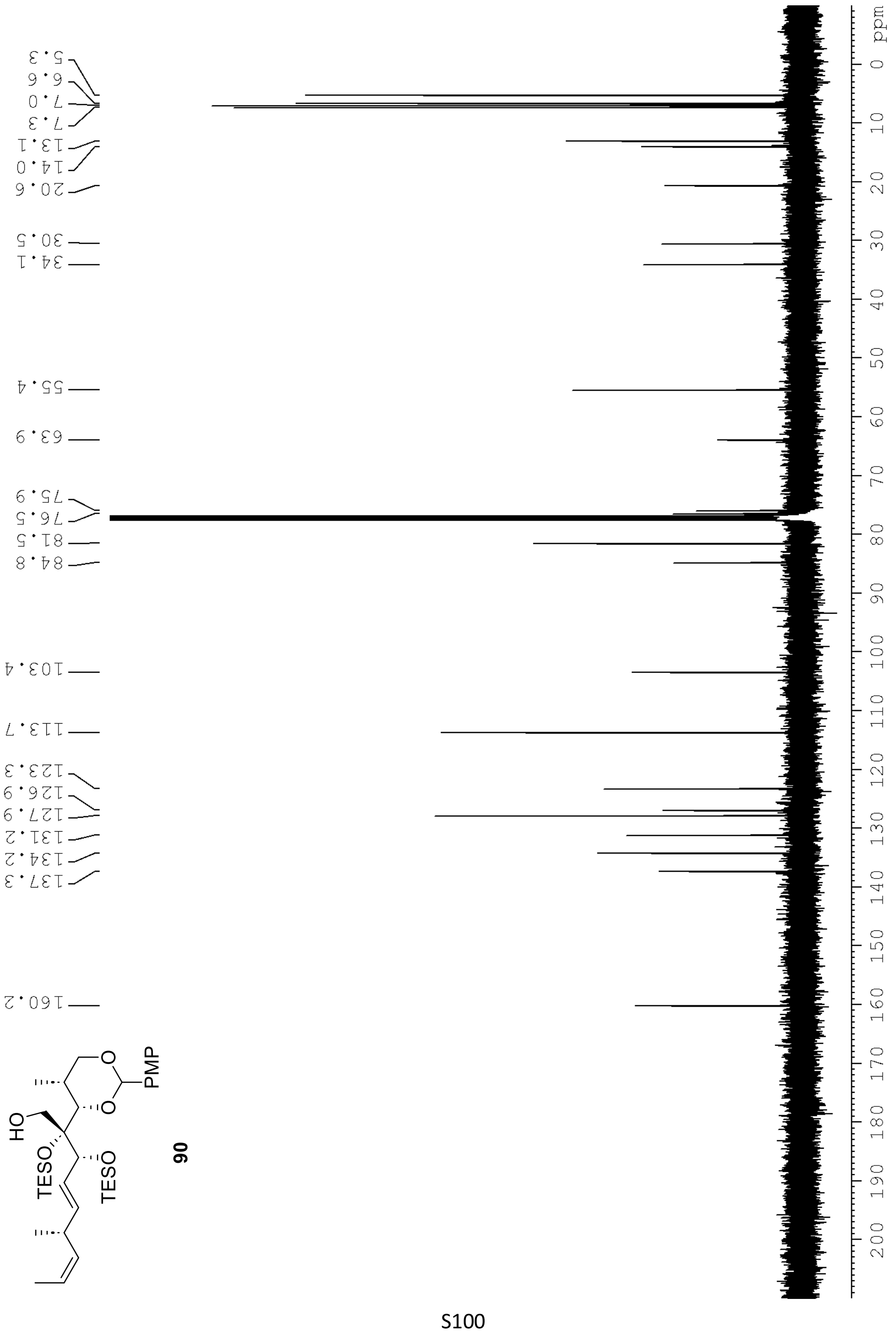

90

Alcohol **91**

^{1}H-NMR (400 MHz, $CDCl_3$)

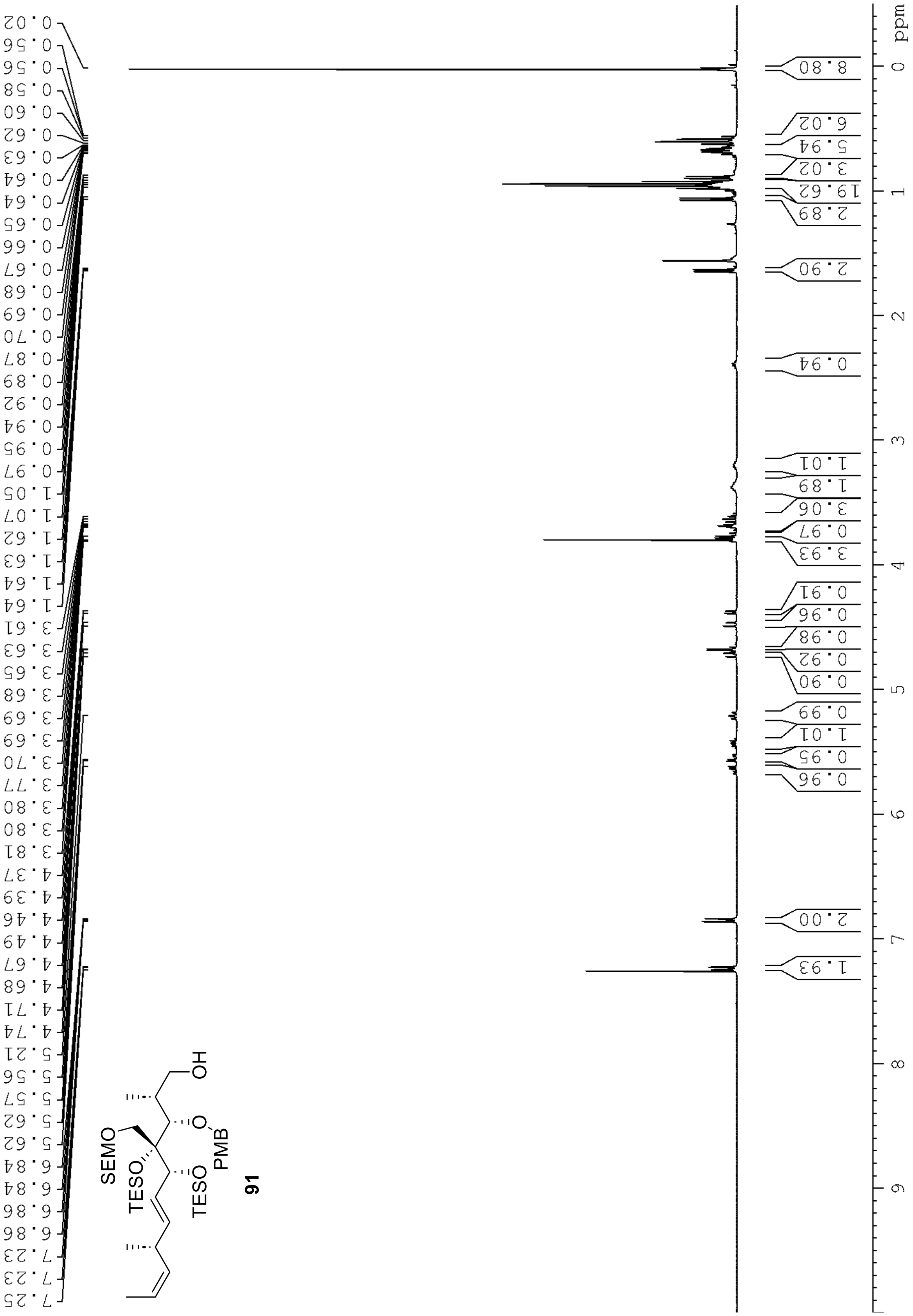

$^{13}C\{^1H\}$-NMR (100 MHz, $CDCl_3$)

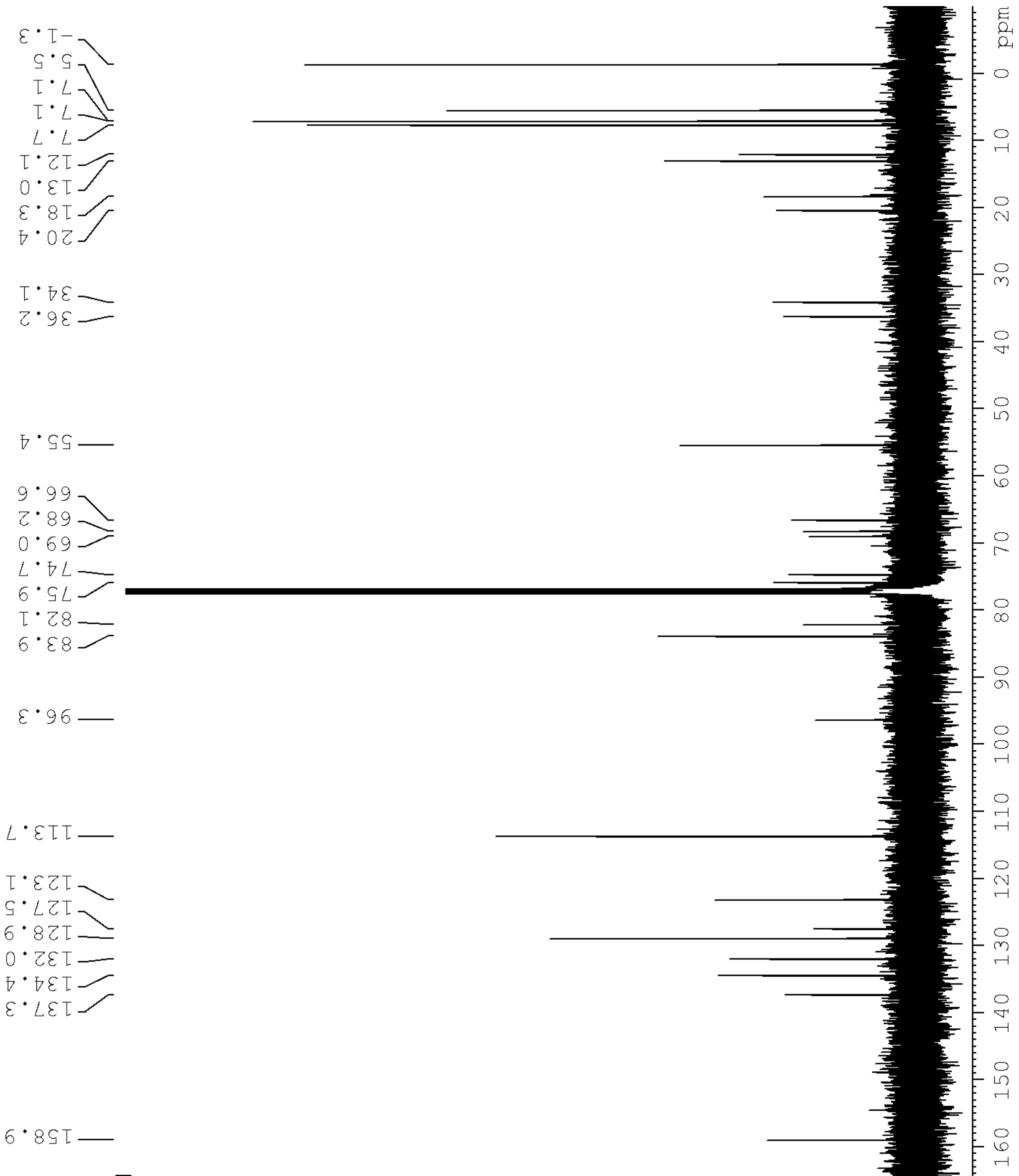

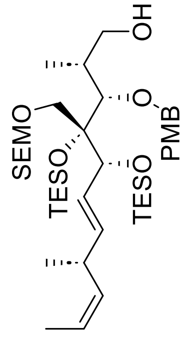

91

PMP-acetal **S8**

^{1}H NMR (500 MHz, C_6D_6)

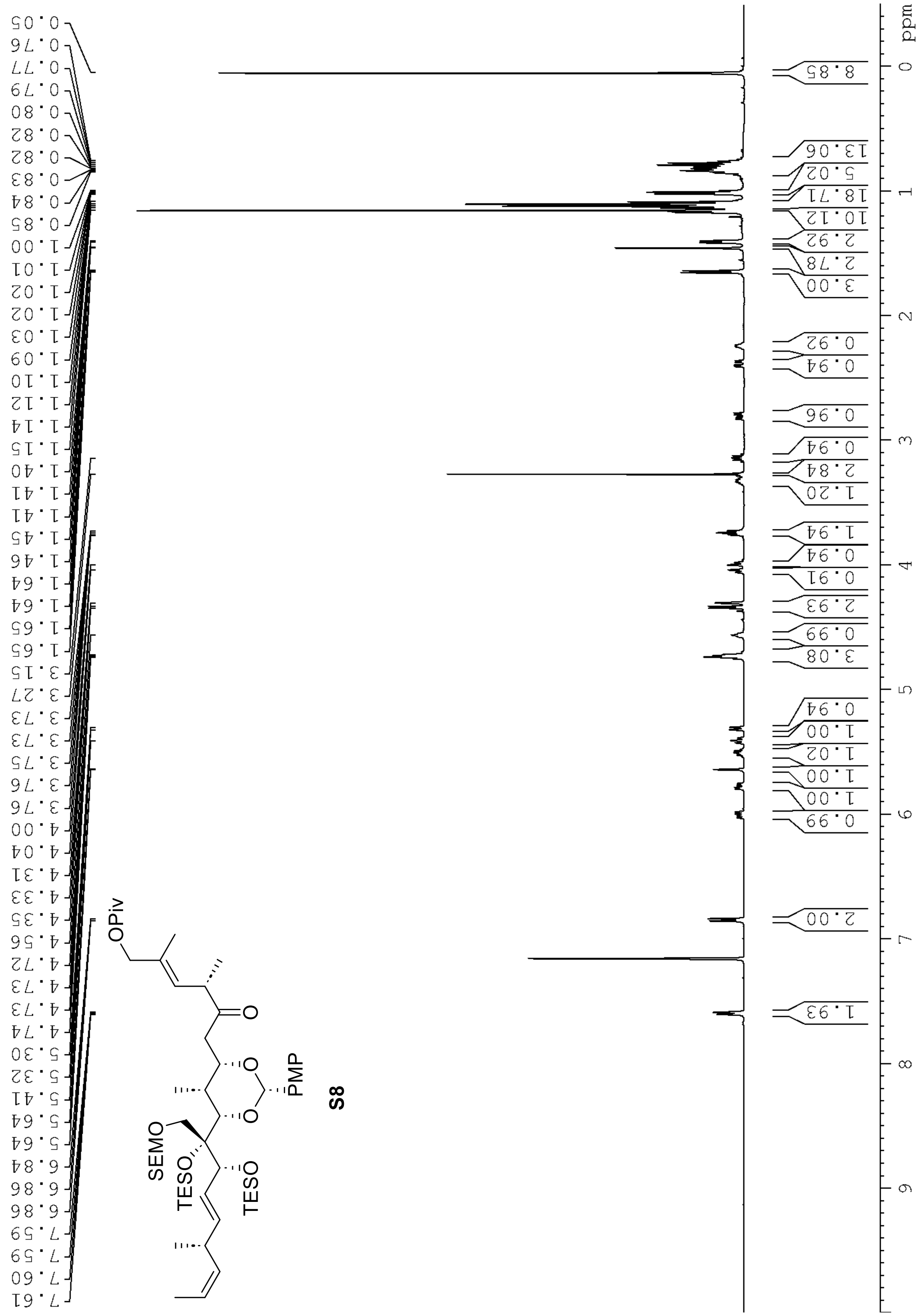

^{13}C{1H}-NMR (125 MHz, C_6D_6)

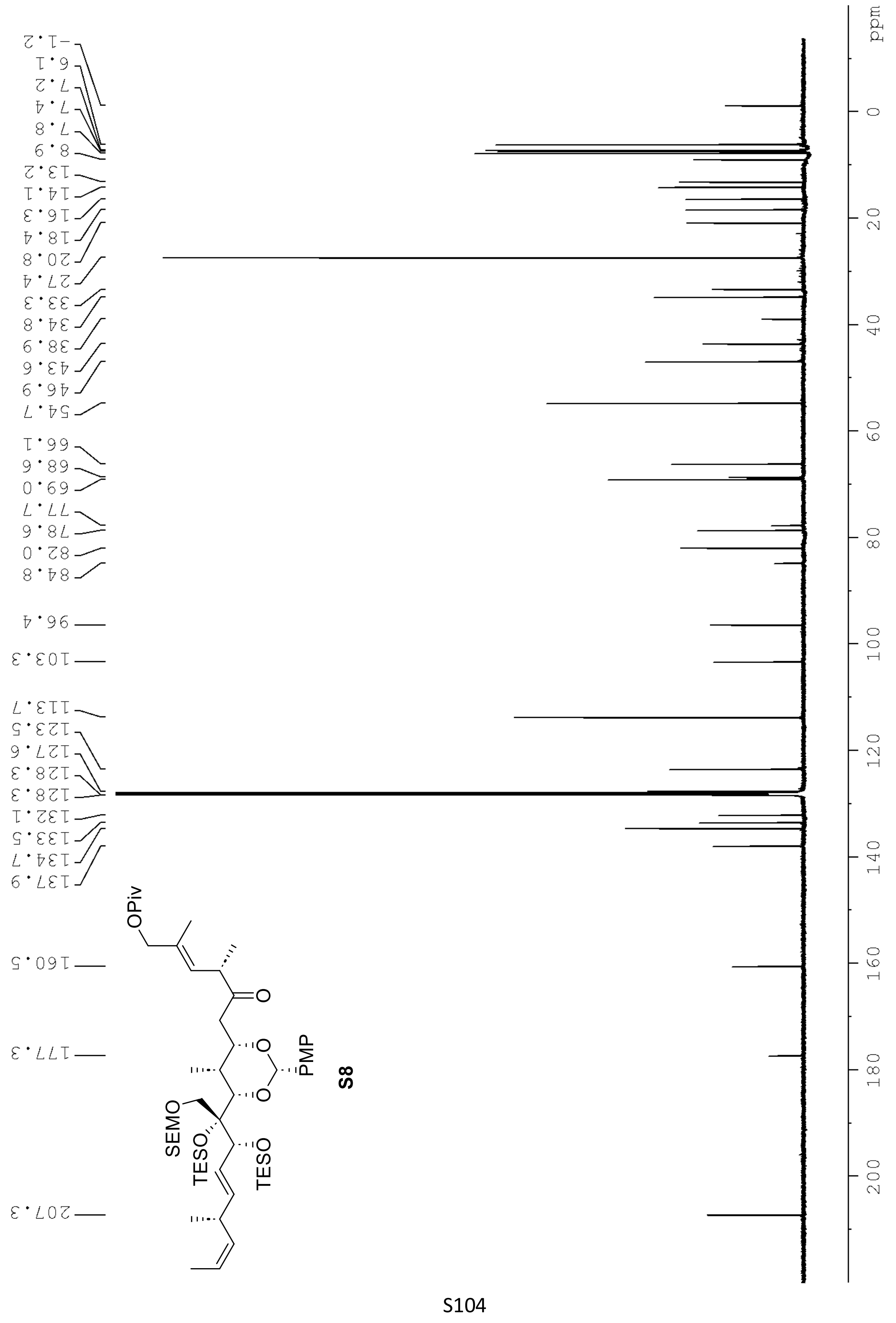

Diol **92**

^{1}H-NMR (600 MHz, $CDCl_3$)

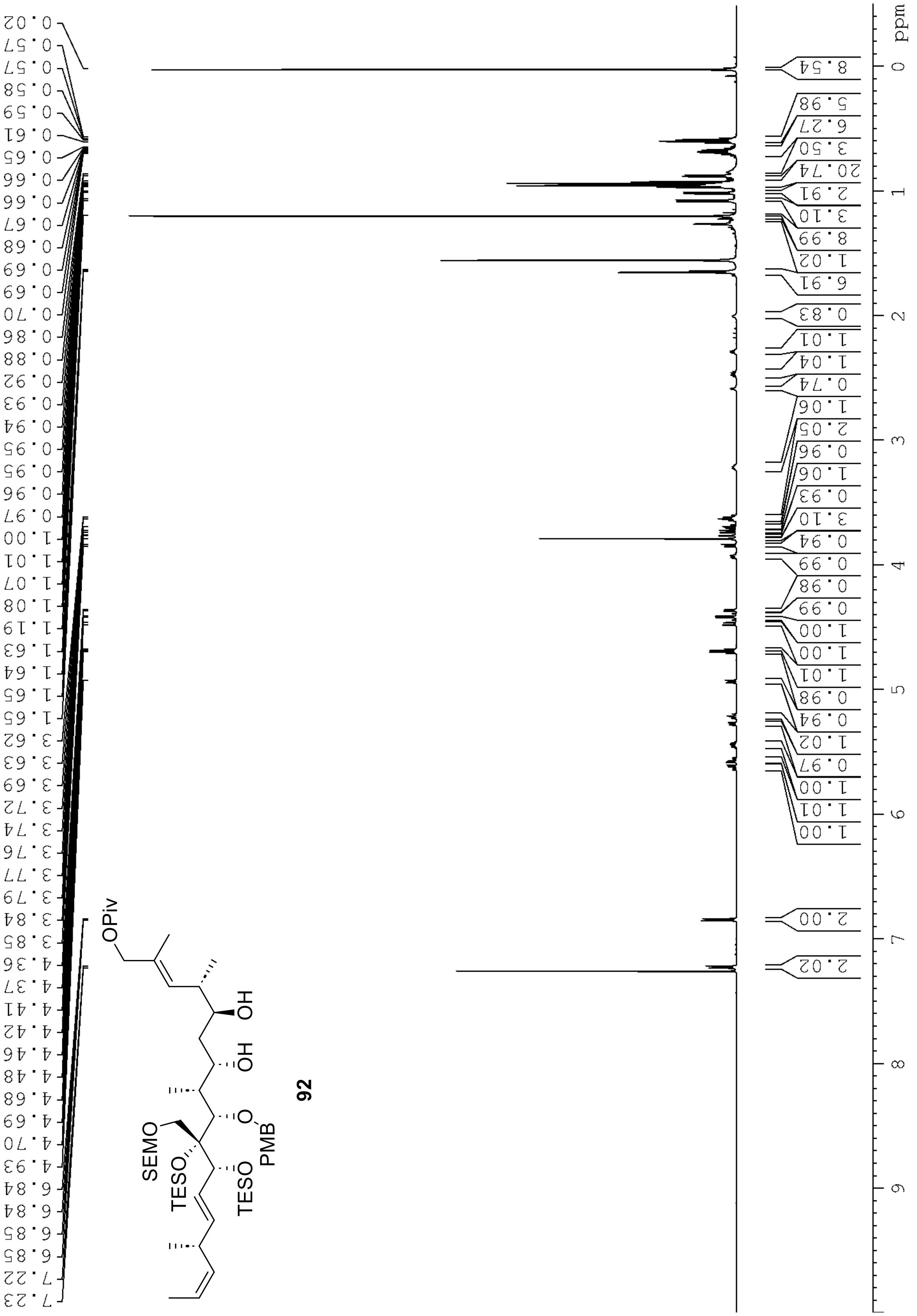

$^{13}C\{1H\}$-NMR (150 MHz, $CDCl_3$)

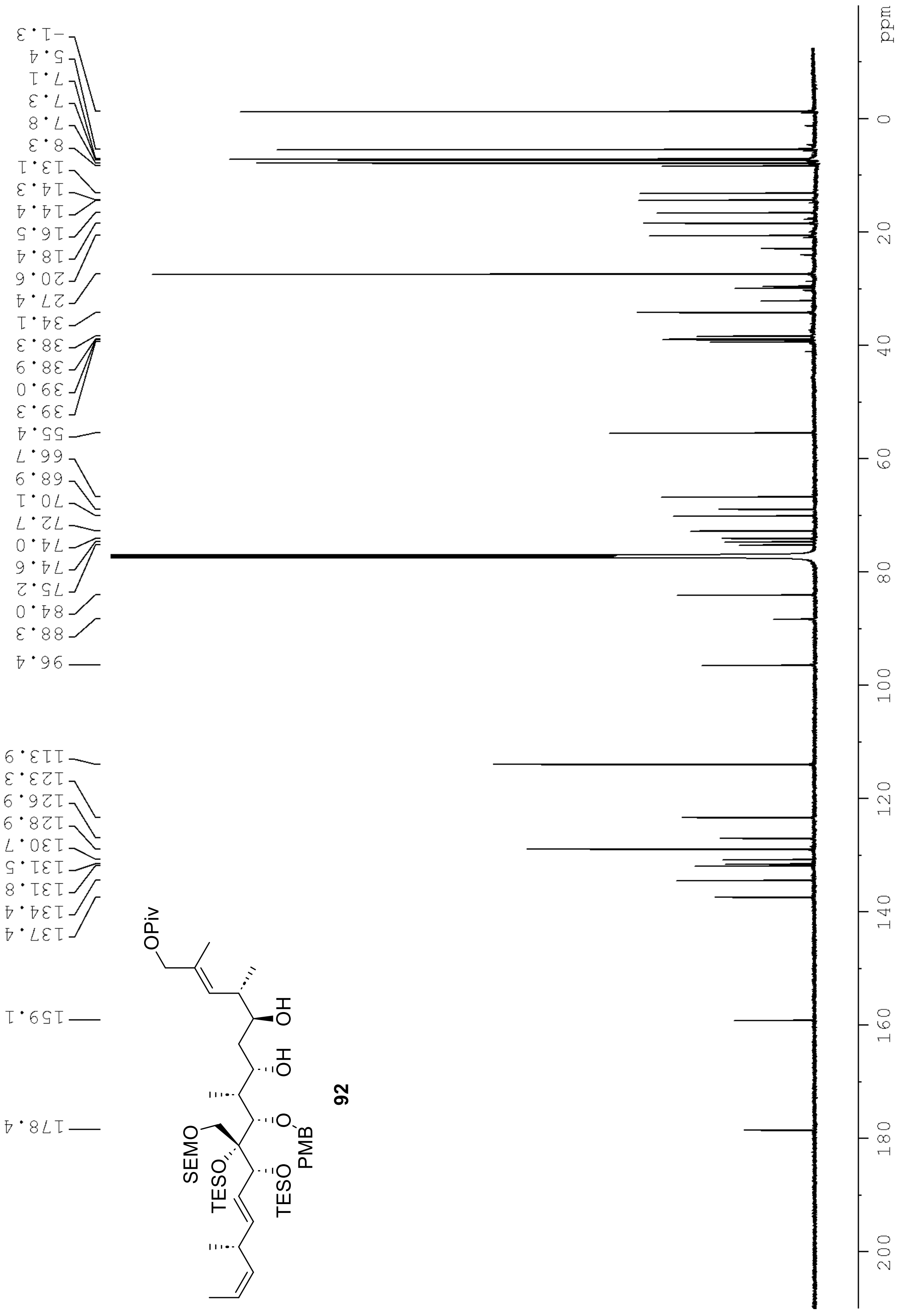

92

Acetonide **S9**

^{1}H-NMR (500 MHz, $CDCl_3$)

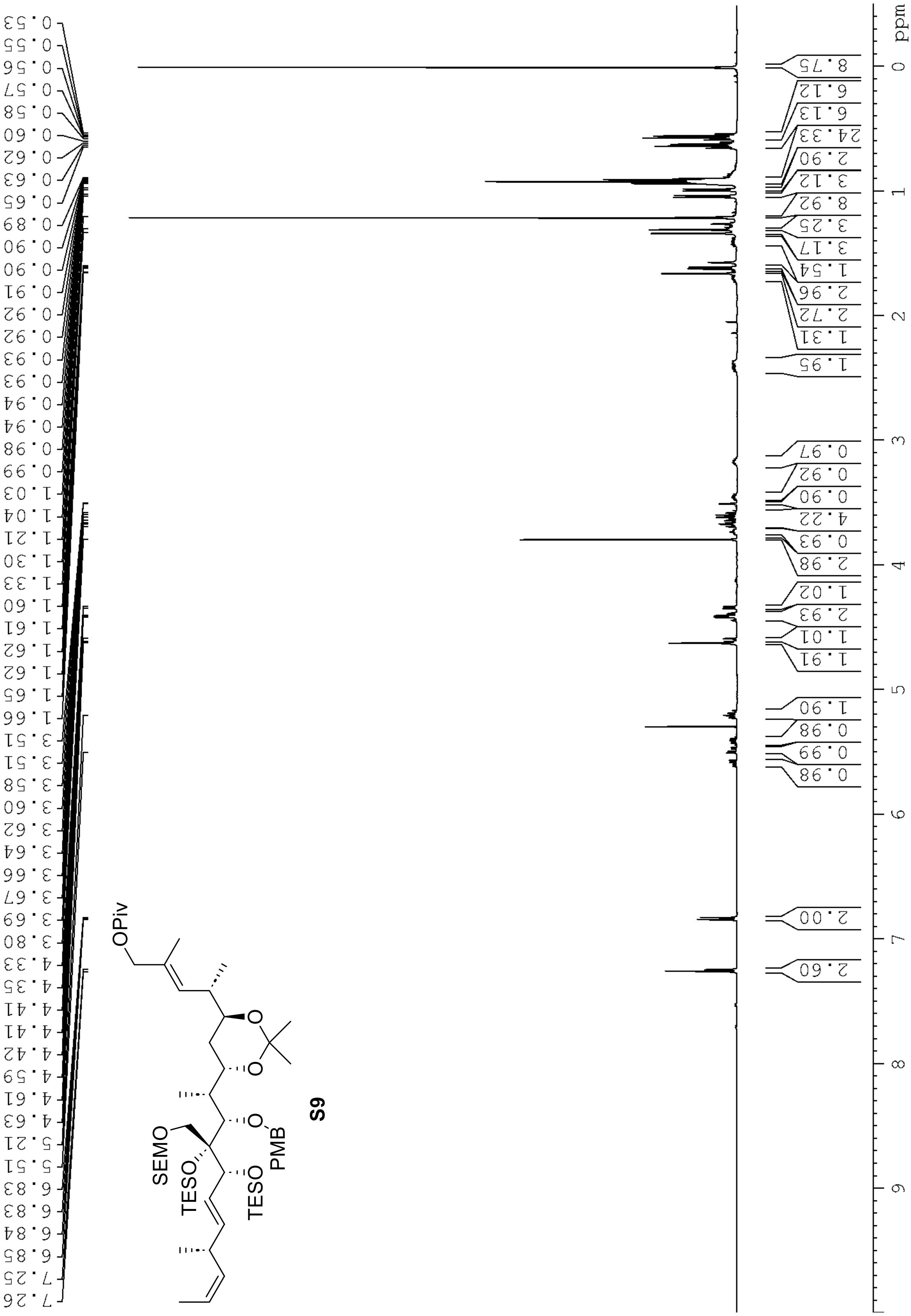

$^{13}C\{^1H\}$-NMR (125 MHz, $CDCl_3$)

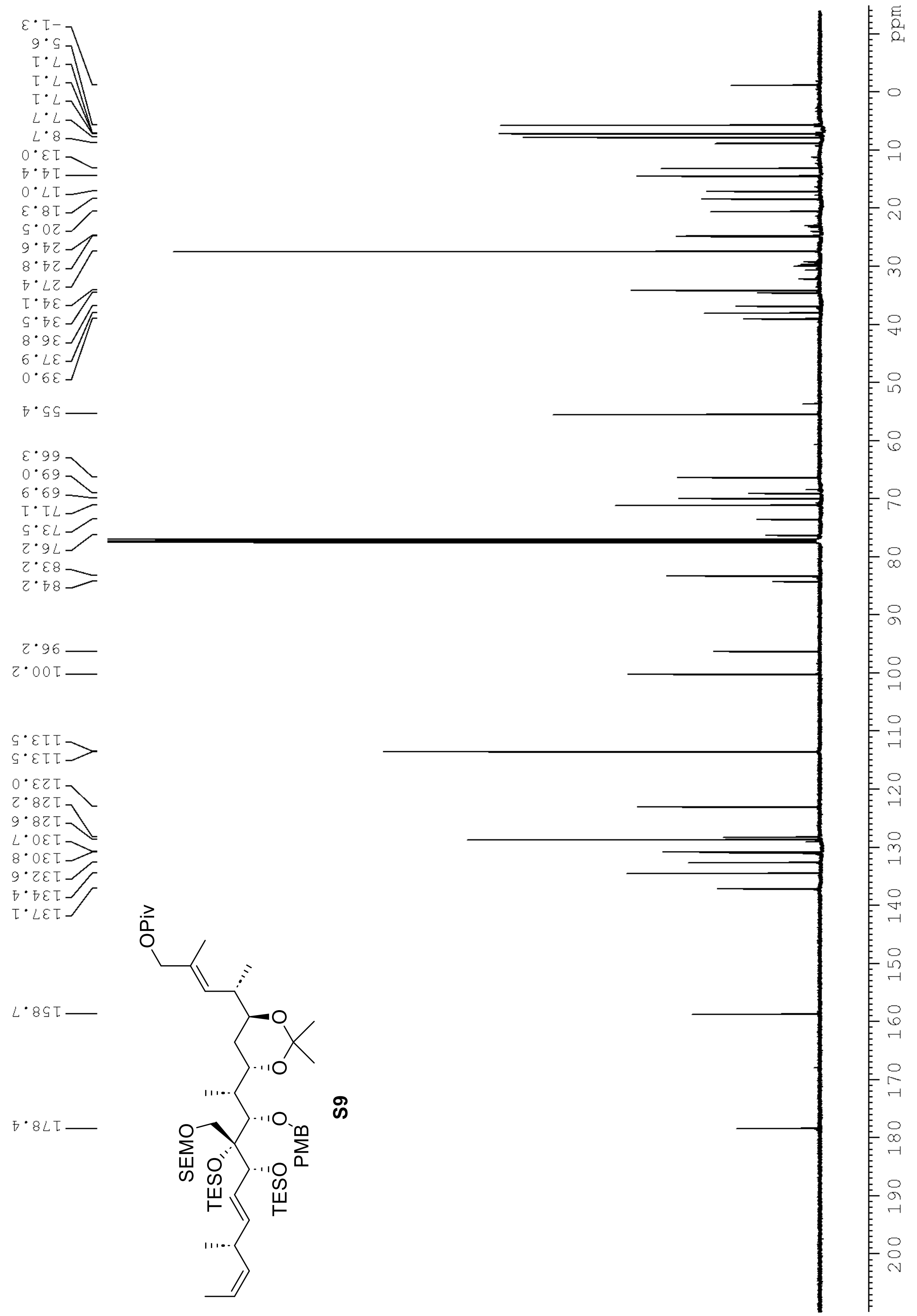

TES-ether **93**

^{1}H-NMR (500 MHz, $CDCl_3$)

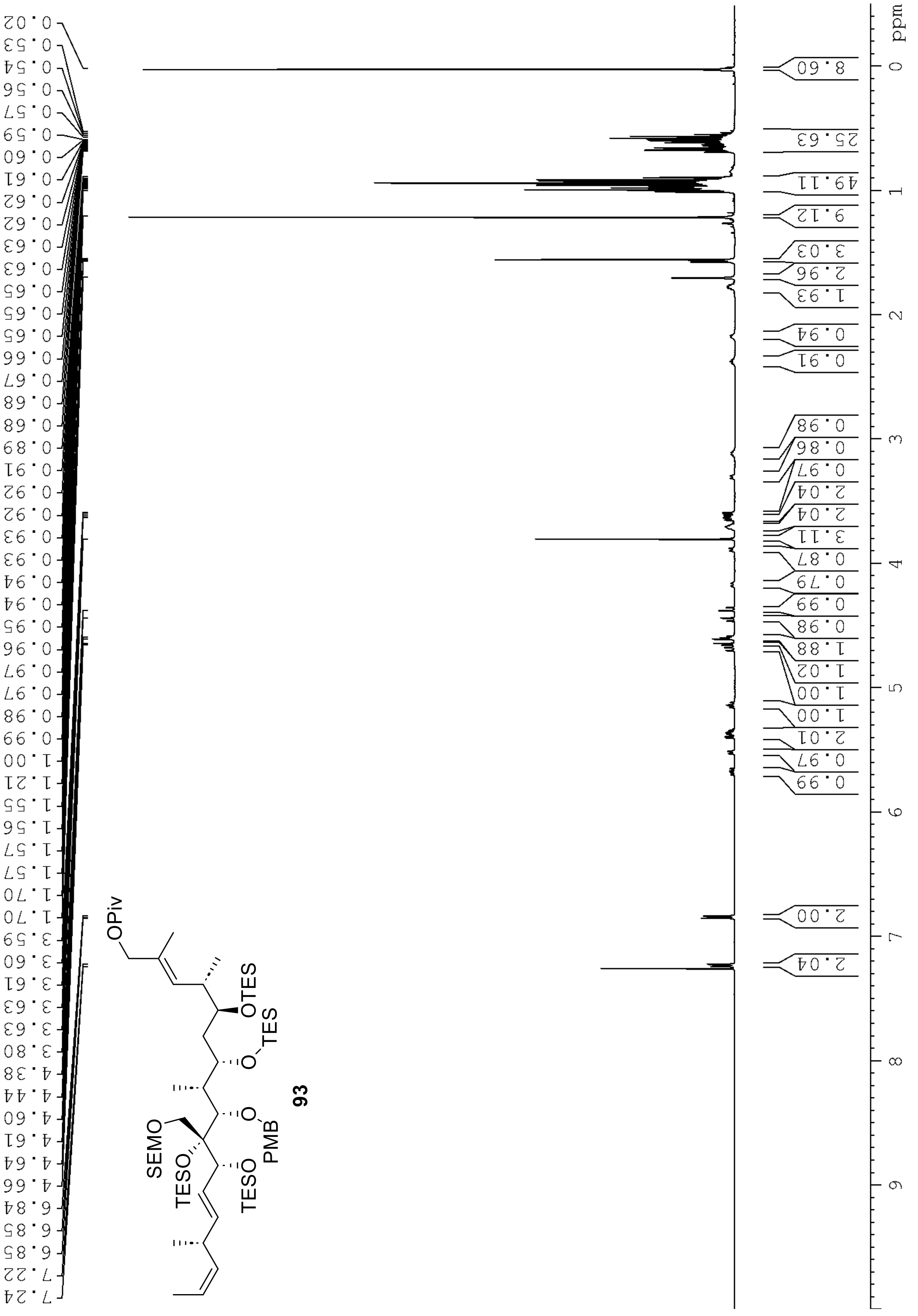

$^{13}C\{1H\}$-NMR (125 MHz, $CDCl_3$)

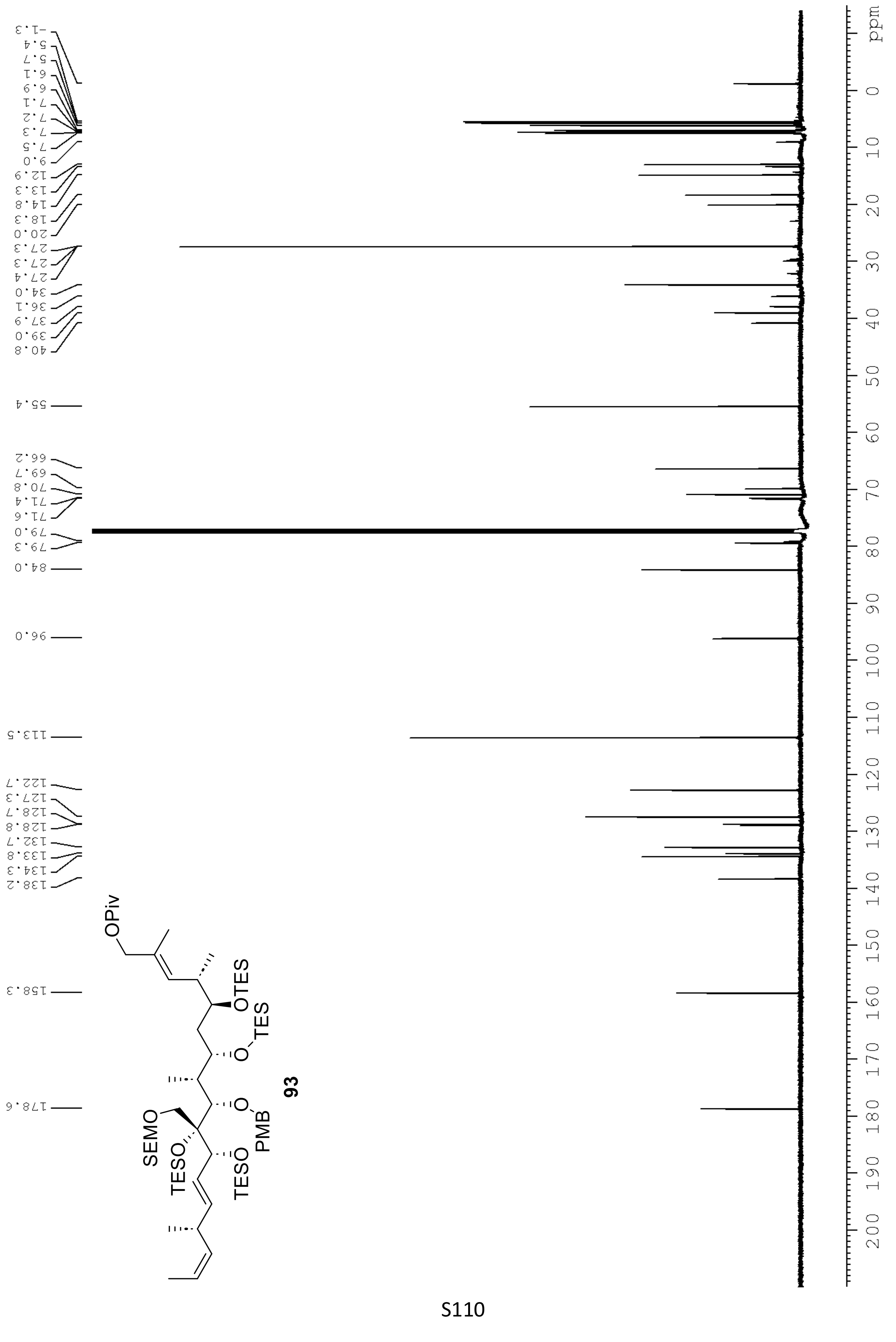

93

Alcohol **94**

^{1}H-NMR (400 MHz, $CDCl_3$)

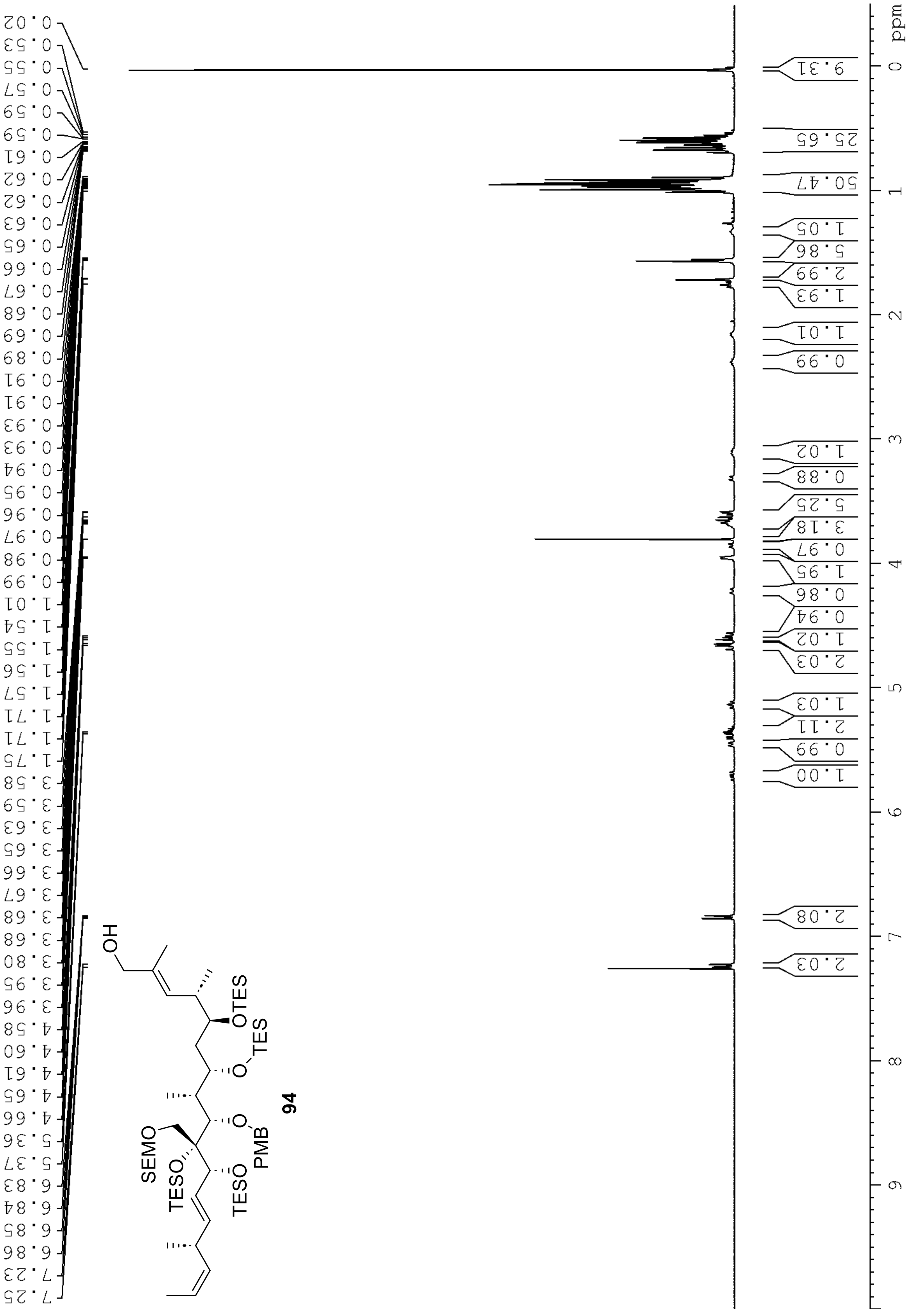

$^{13}C\{1H\}$-NMR (100 MHz, $CDCl_3$)

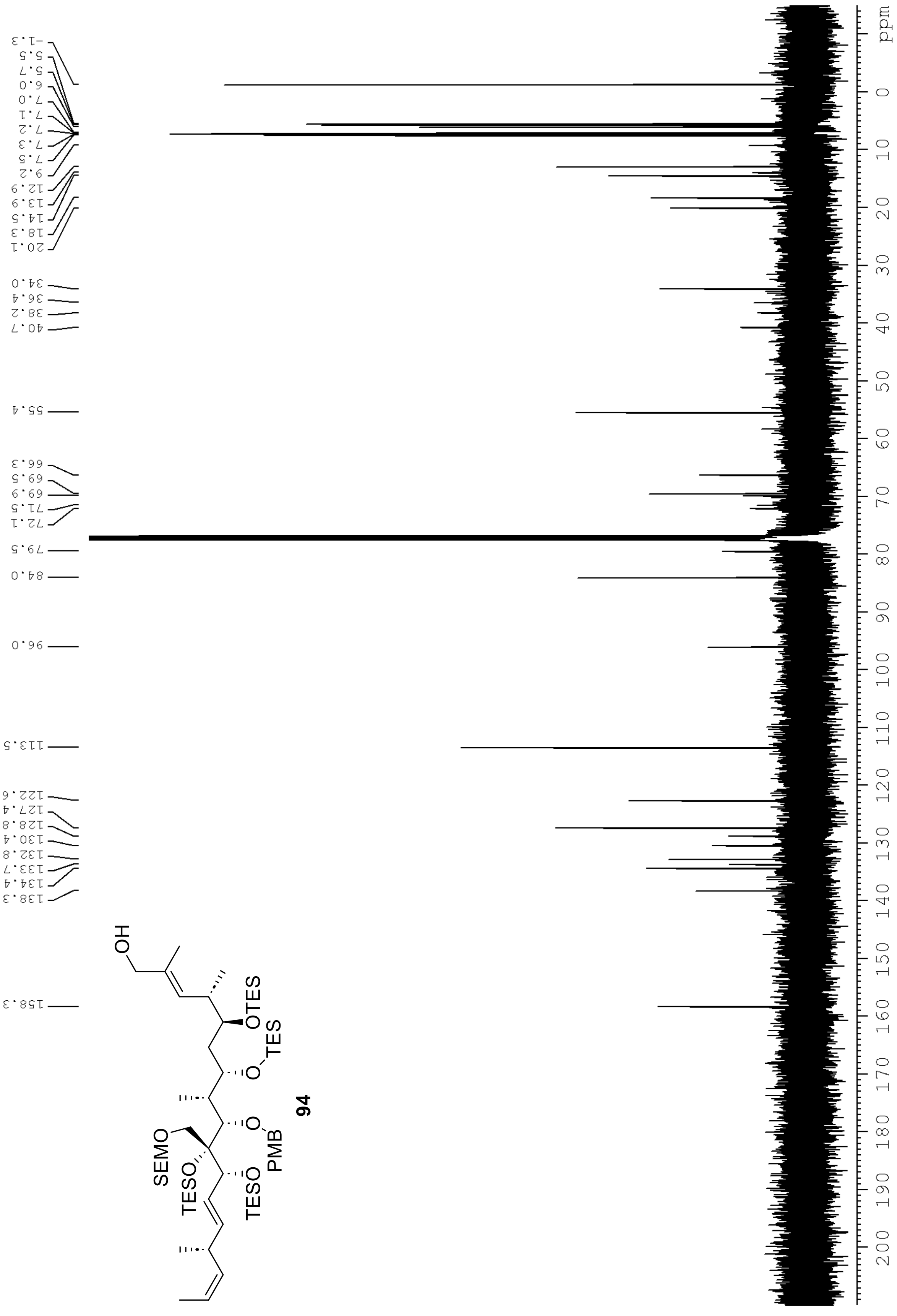

(*S*)-Mosher ester **S10**

^{1}H-NMR (400 MHz, C_6D_6)

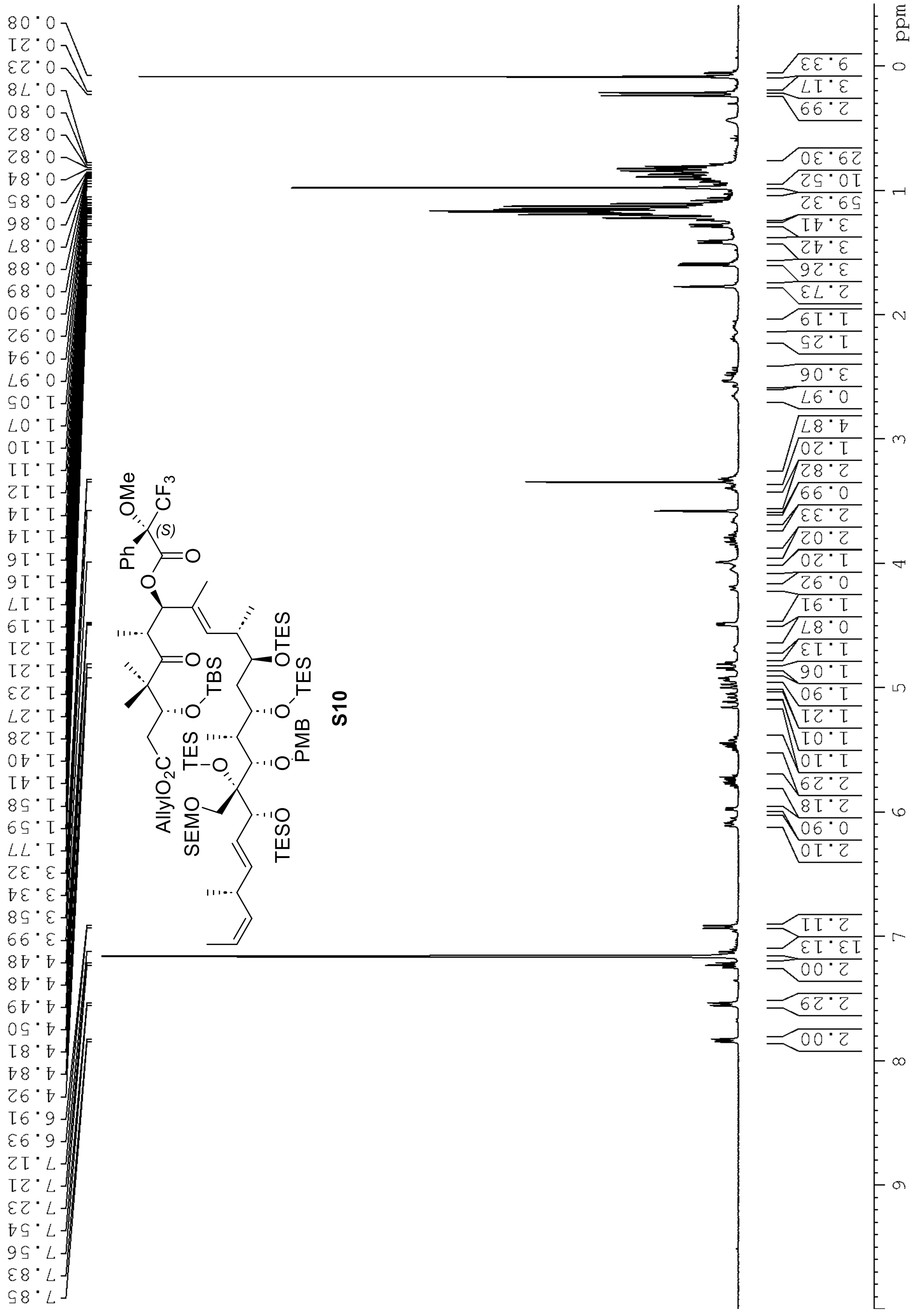

(*R*)-Mosher ester **S11**

^{1}H-NMR (400 MHz, C_6D_6)

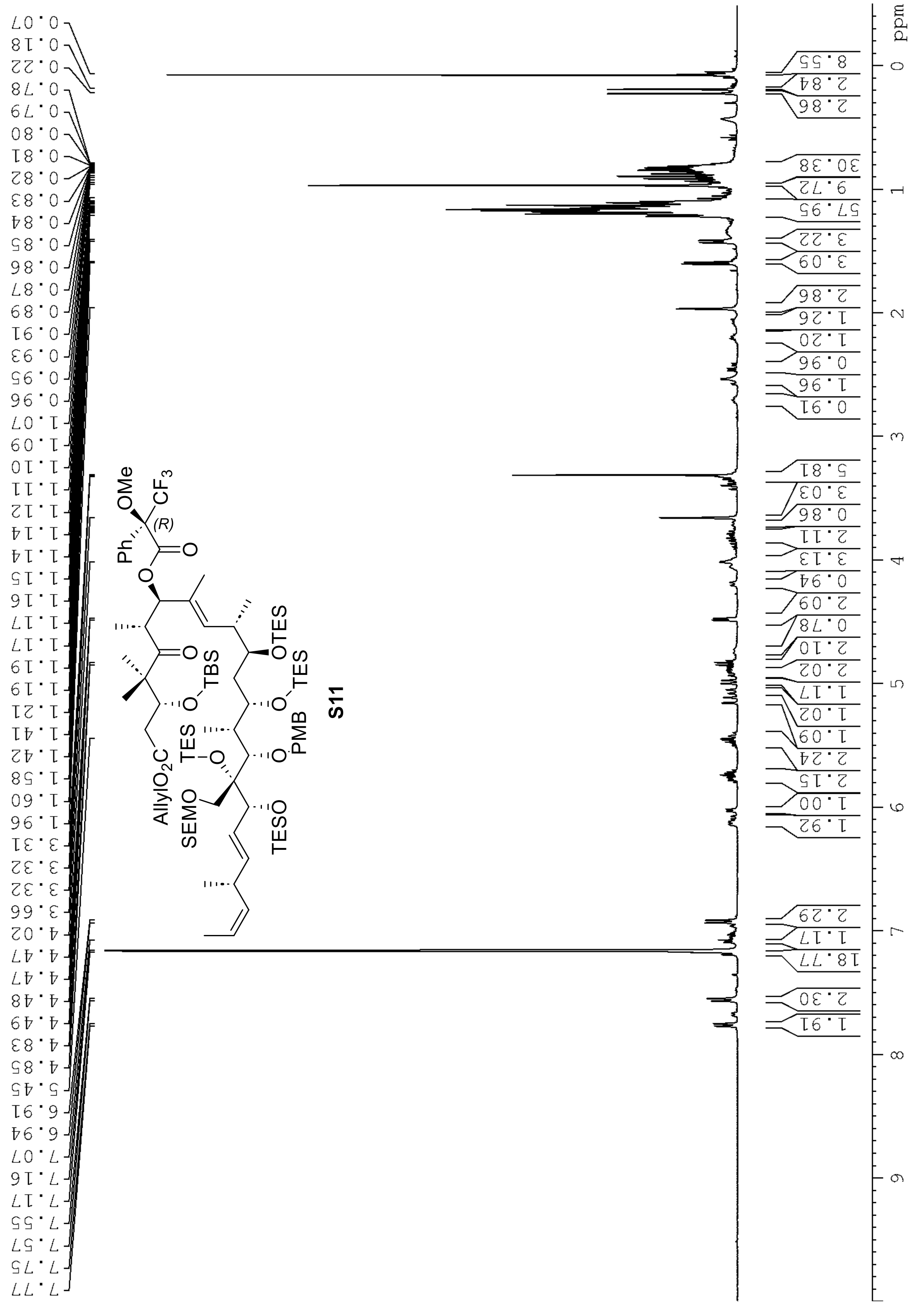

TIPS-ether **60**

^{1}H-NMR (500 MHz, $CDCl_3$)

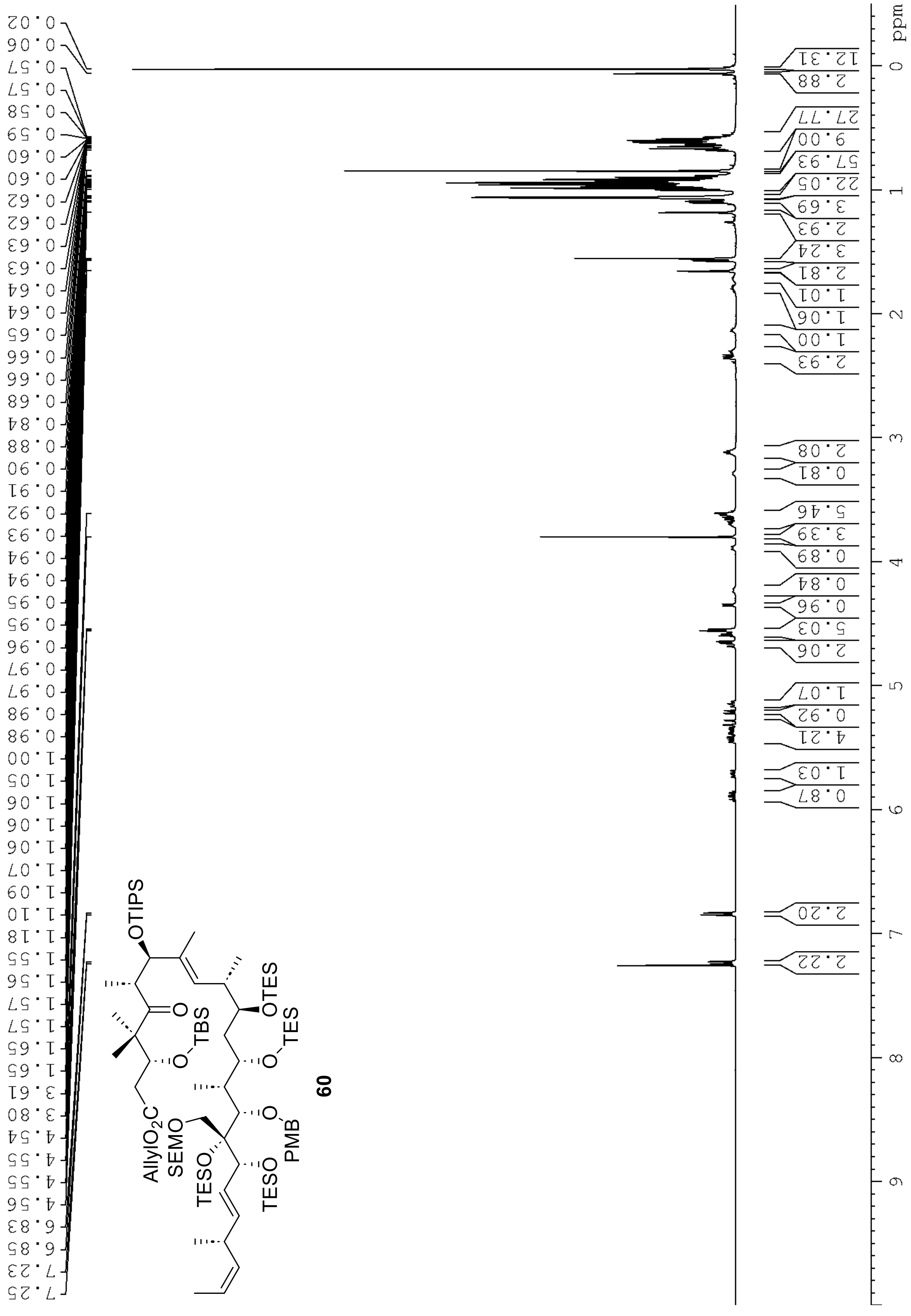

$^{13}C\{1H\}$-NMR (100 MHz, $CDCl_3$)

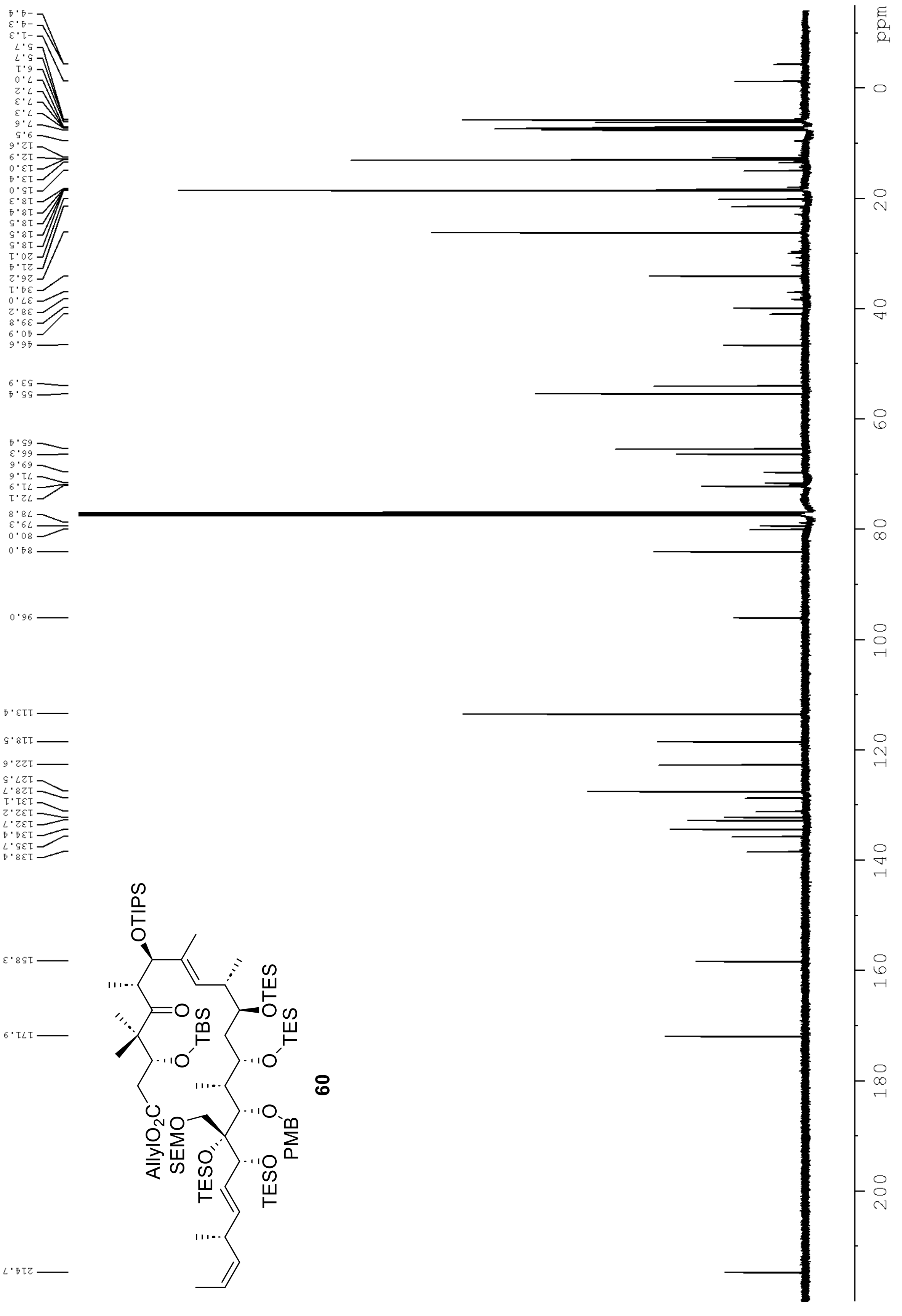

Alcohol **96**

^{1}H-NMR (400 MHz, $CDCl_3$)

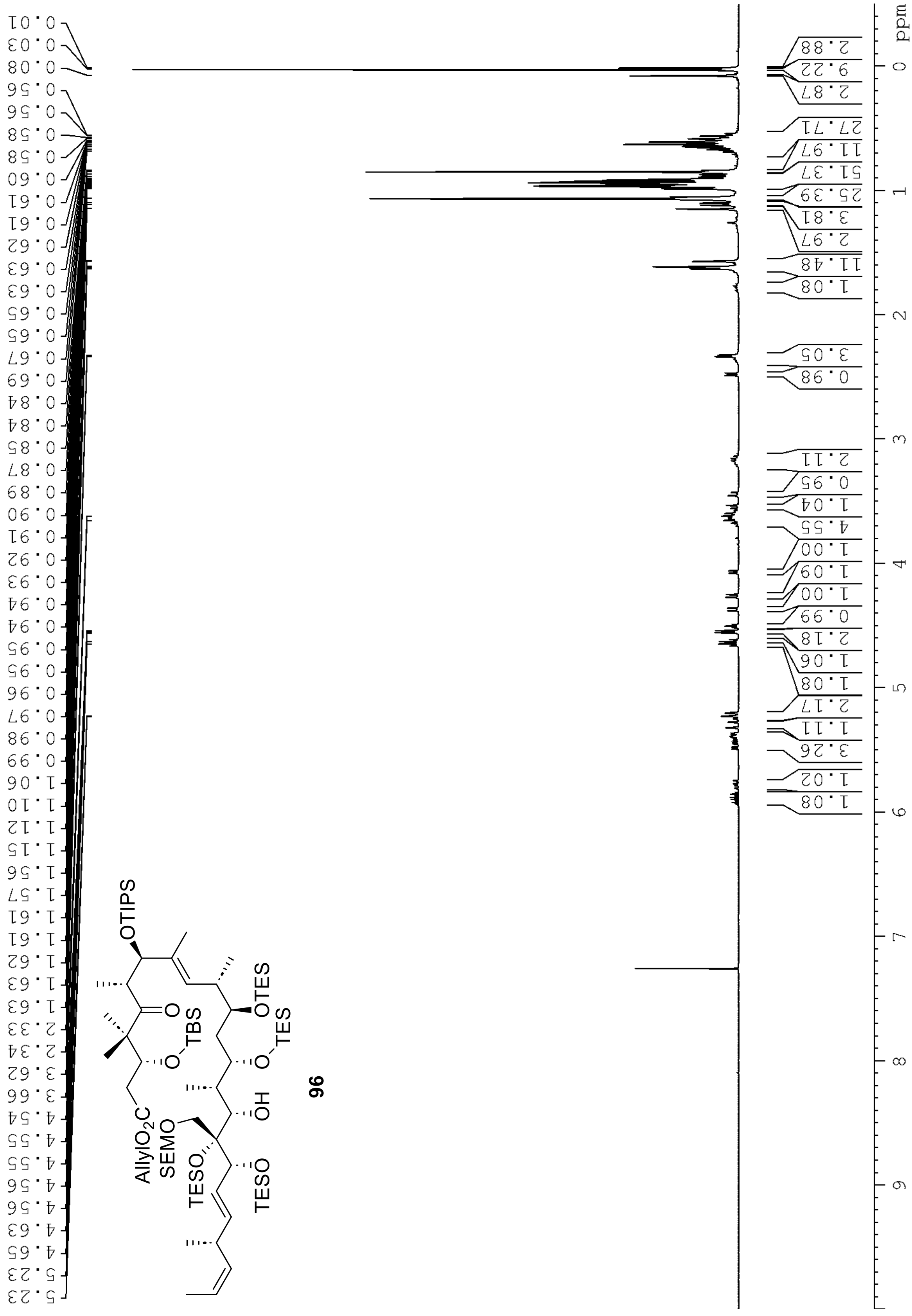

$^{13}C\{1H\}$-NMR (100 MHz, $CDCl_3$)

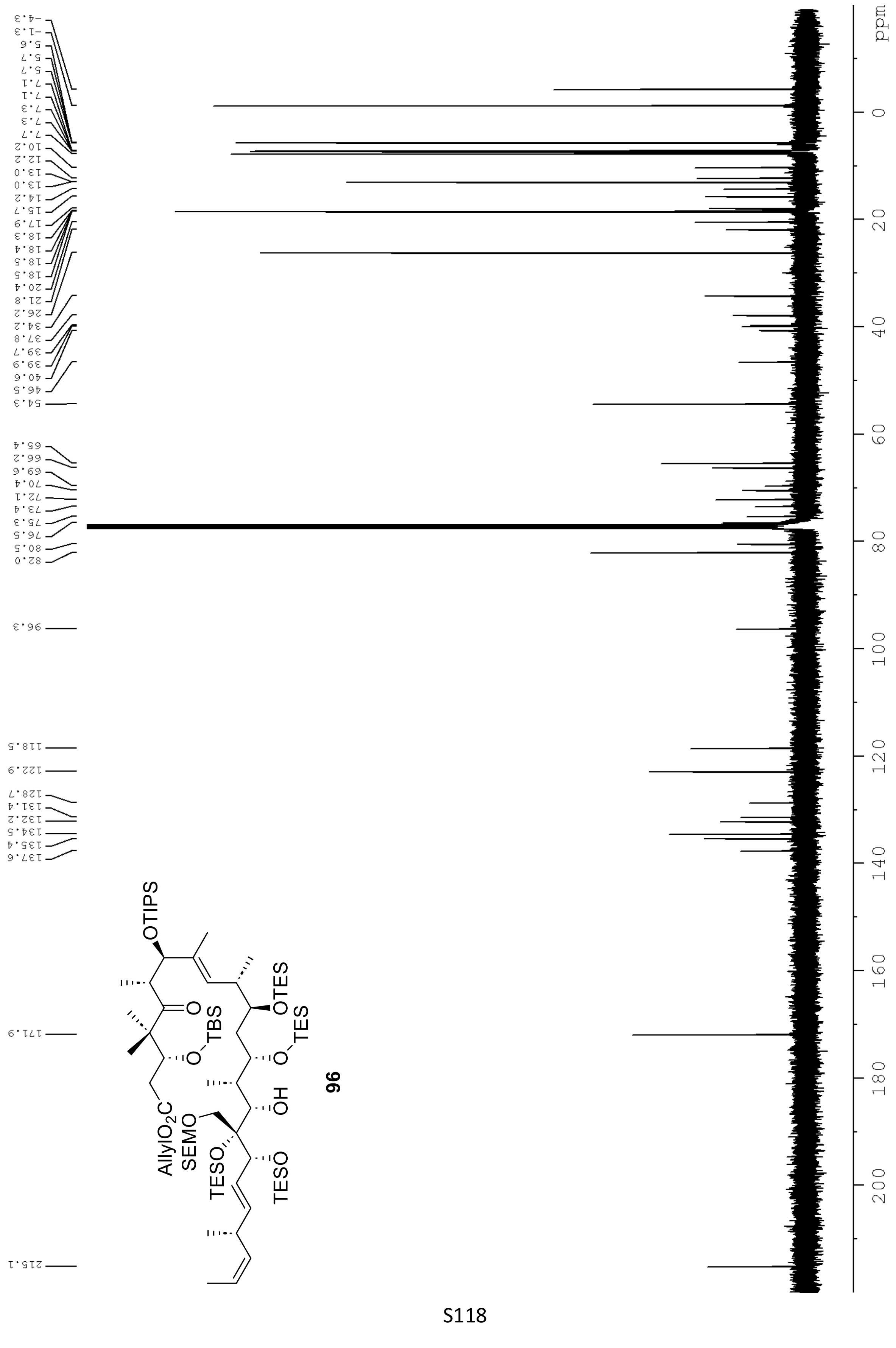

96

Ketone **61**

^{1}H-NMR (400 MHz, $CDCl_3$)

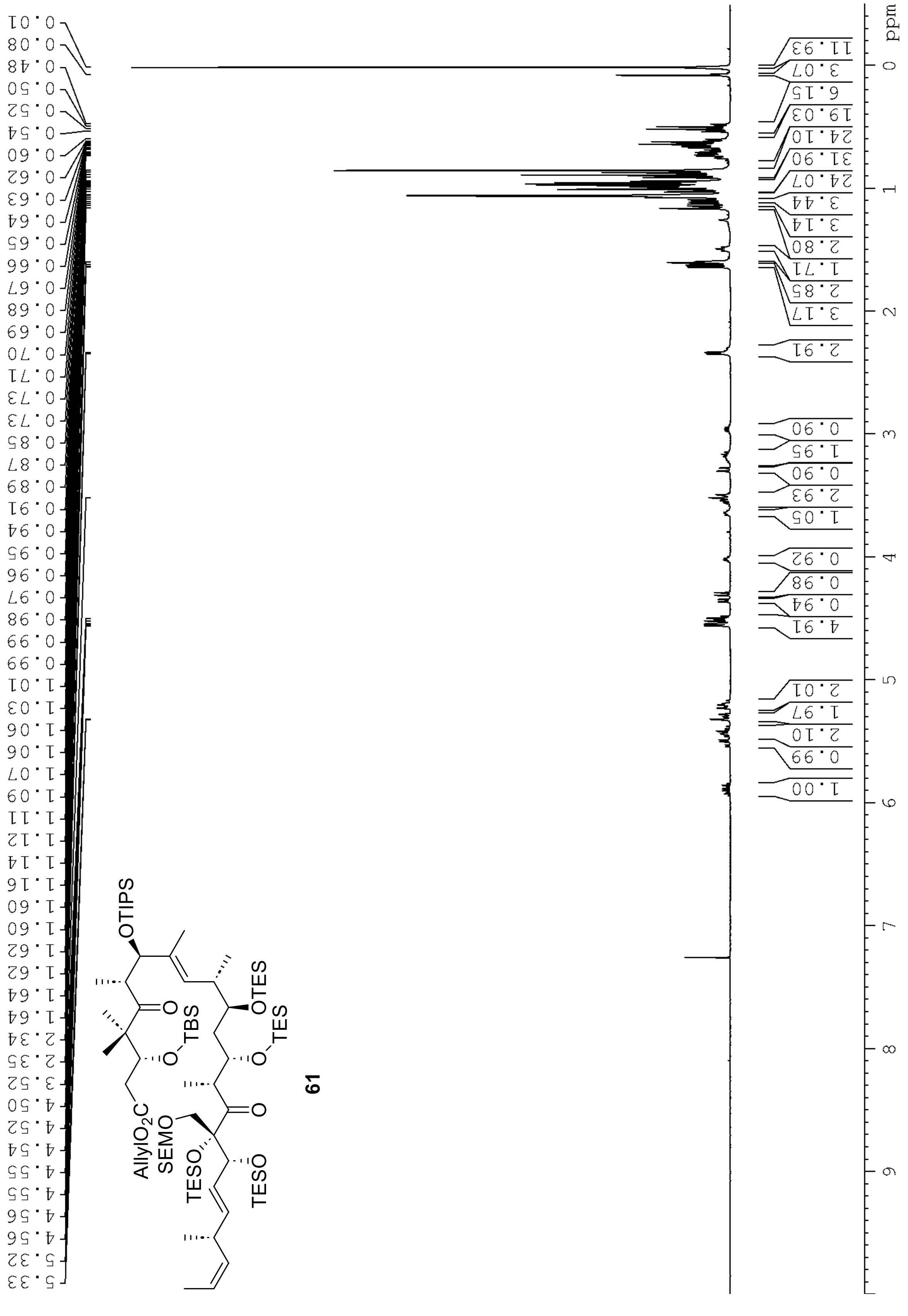

$^{13}C\{1H\}$-NMR (100 MHz, $CDCl_3$)

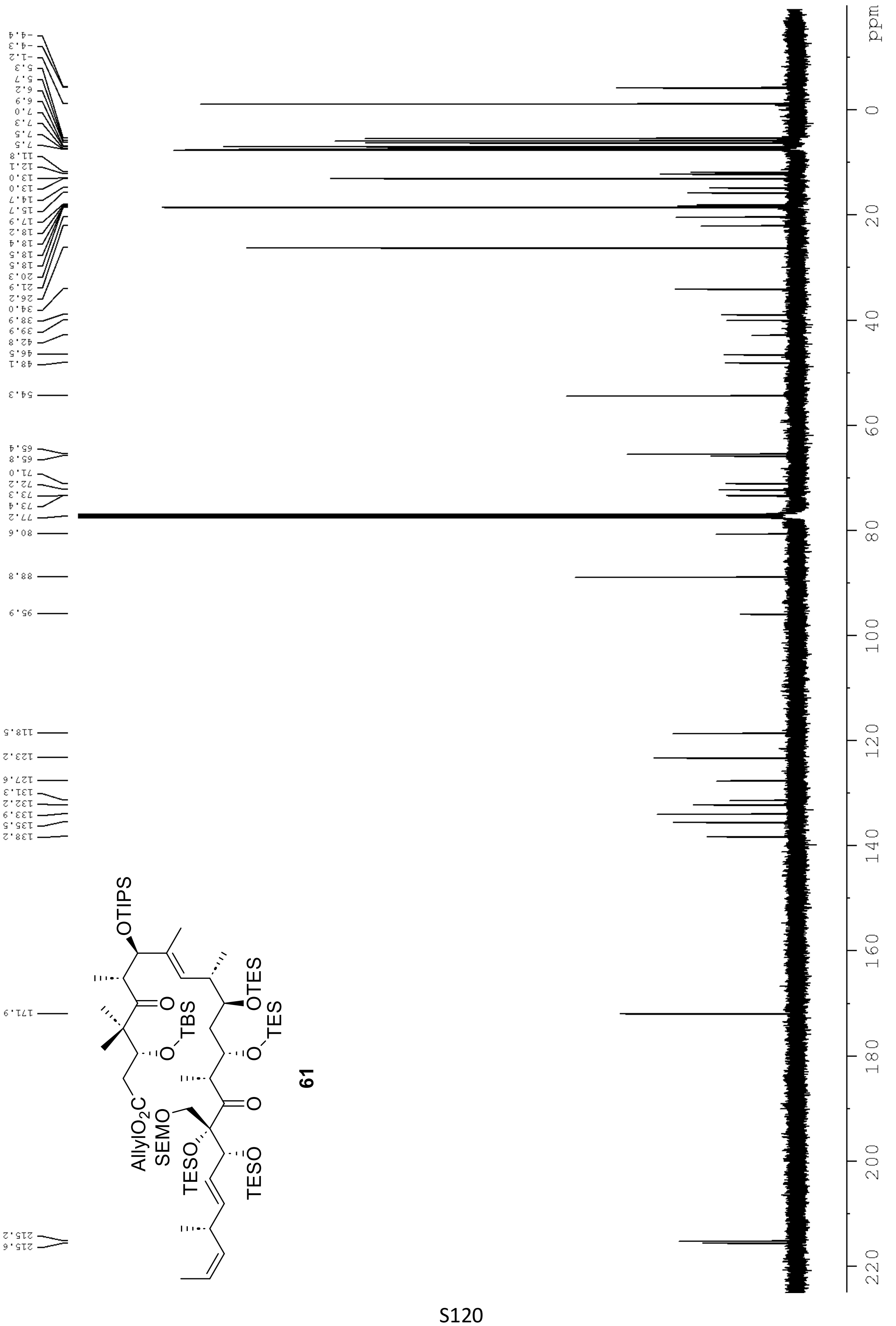

61

Acetonide **64**

^{1}H-NMR (400 MHz, $CDCl_3$)

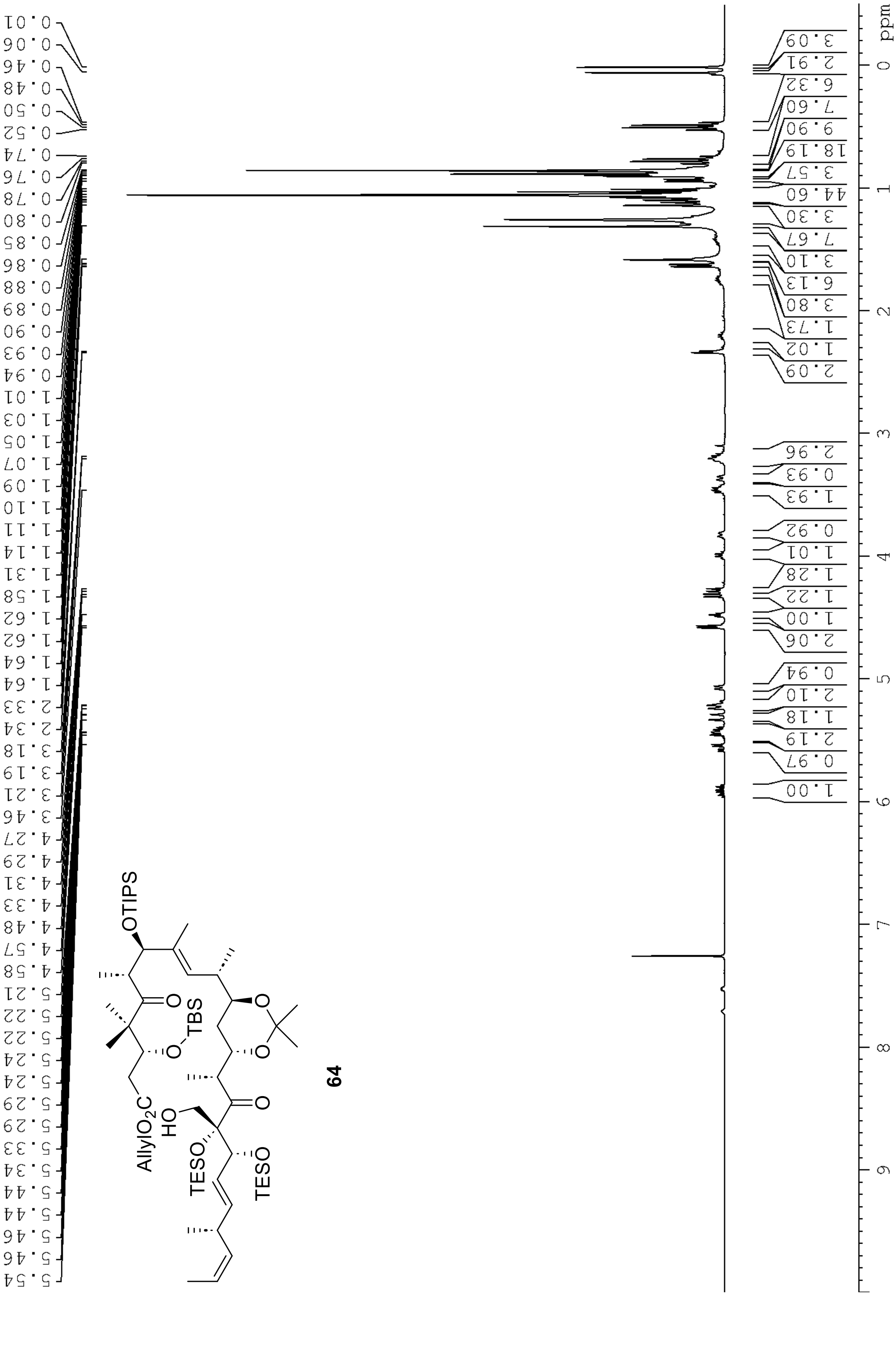

$^{13}C\{1H\}$-NMR (100 MHz, $CDCl_3$)

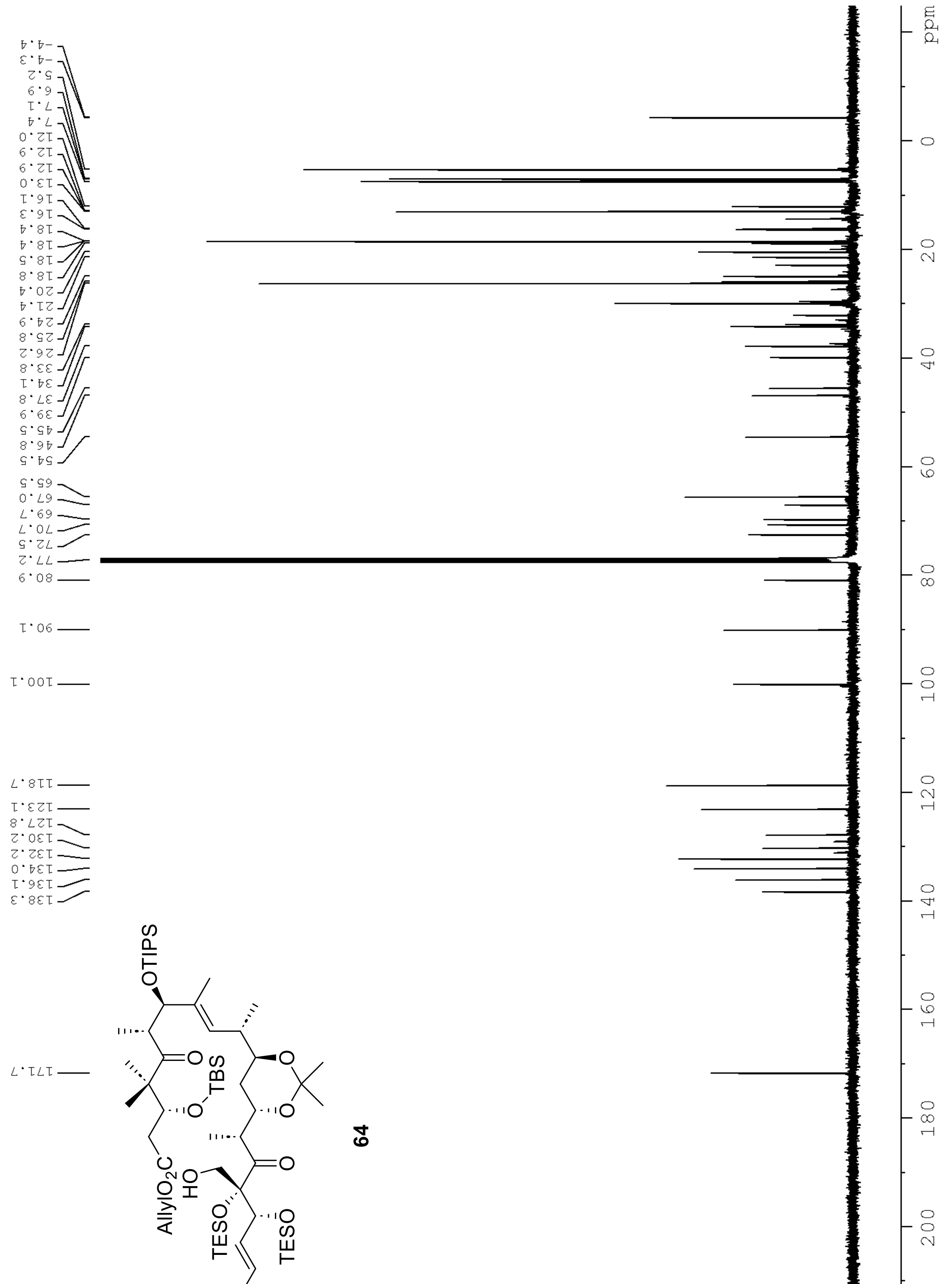

64

Diol **65**

^{1}H-NMR (600 MHz, $CDCl_3$)

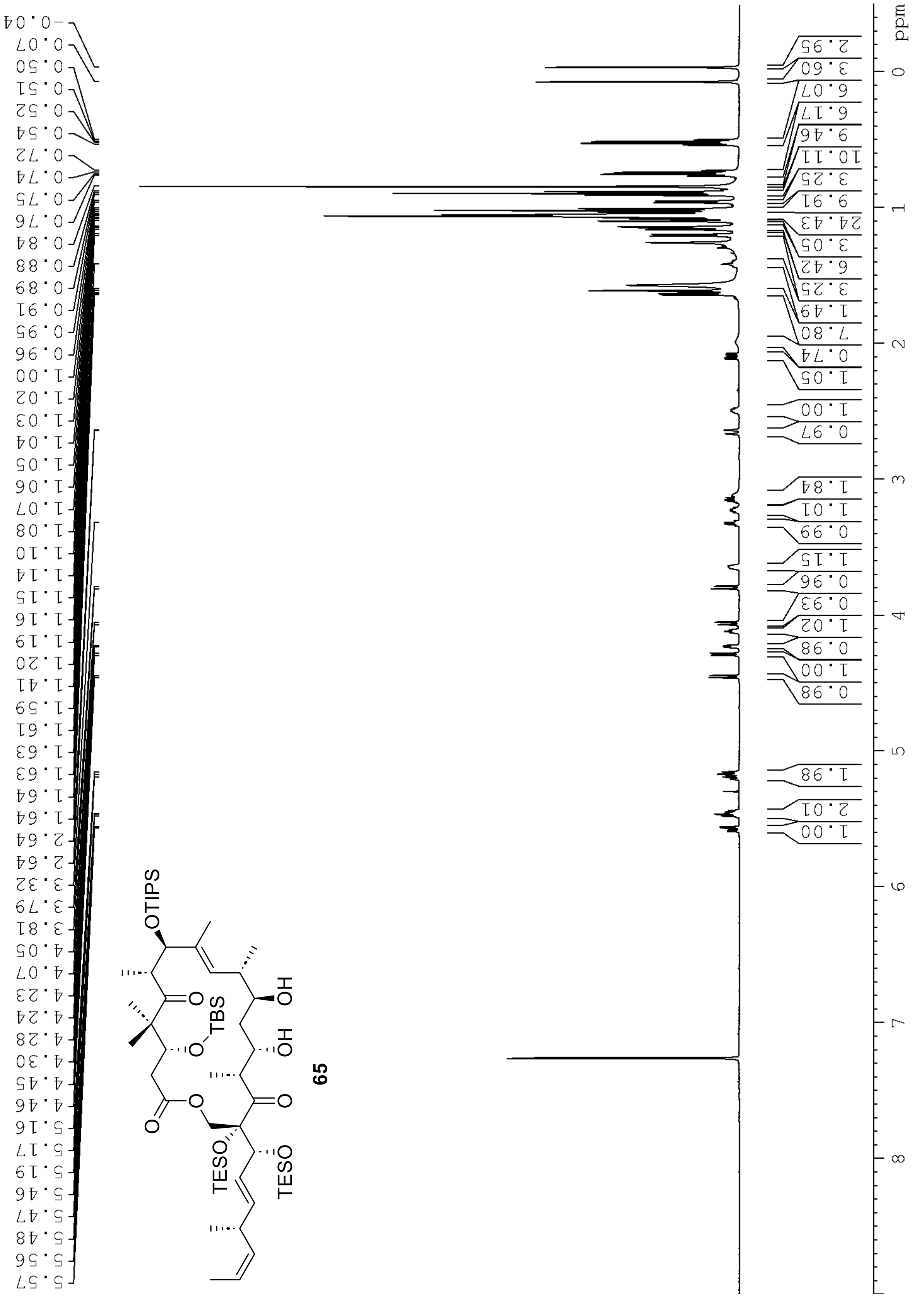

$^{13}C\{^1H\}$-NMR (150 MHz, $CDCl_3$)

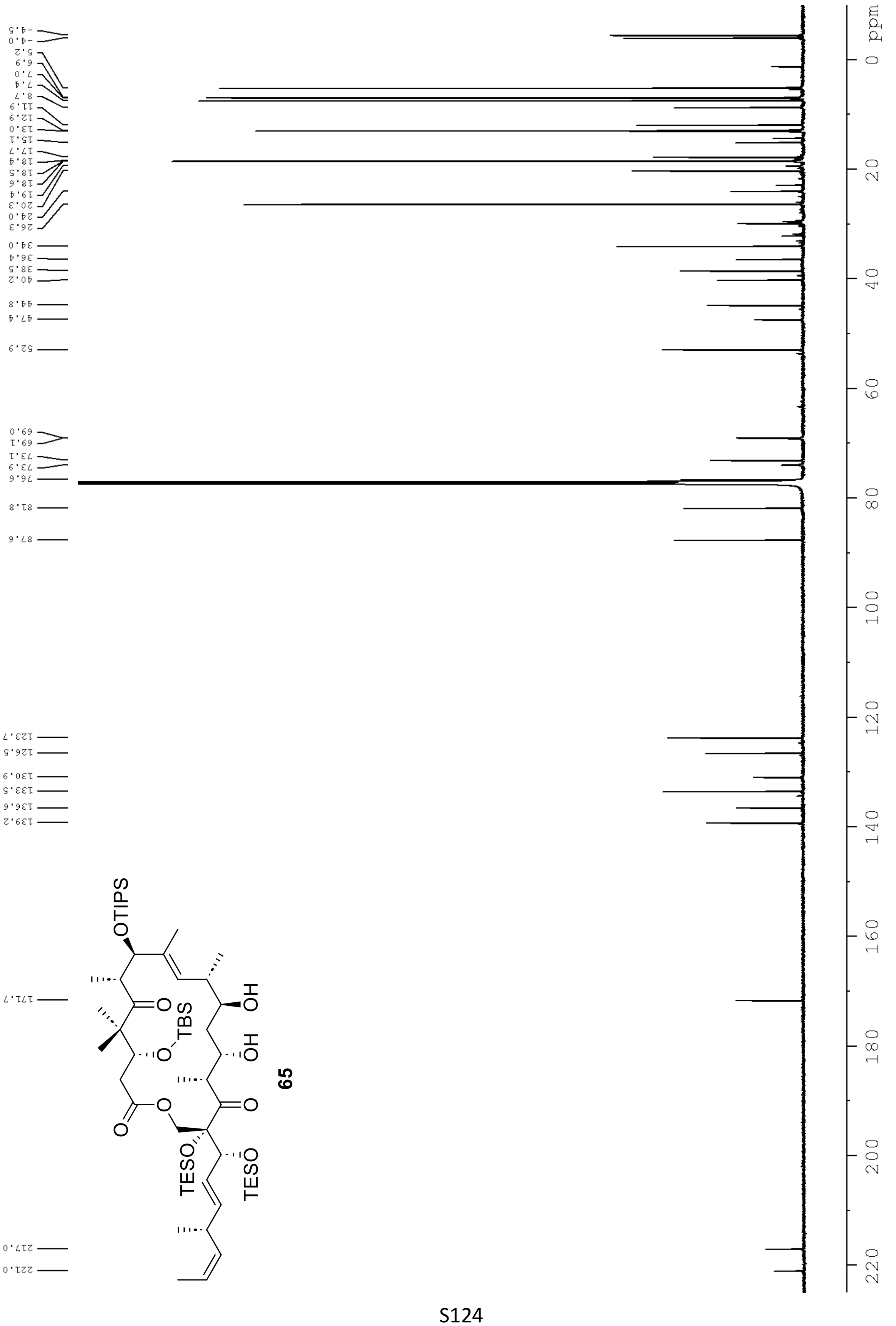

Desepoxy-tedanolide C (**66**)

^{1}H-NMR (600 MHz, CD_3OD)

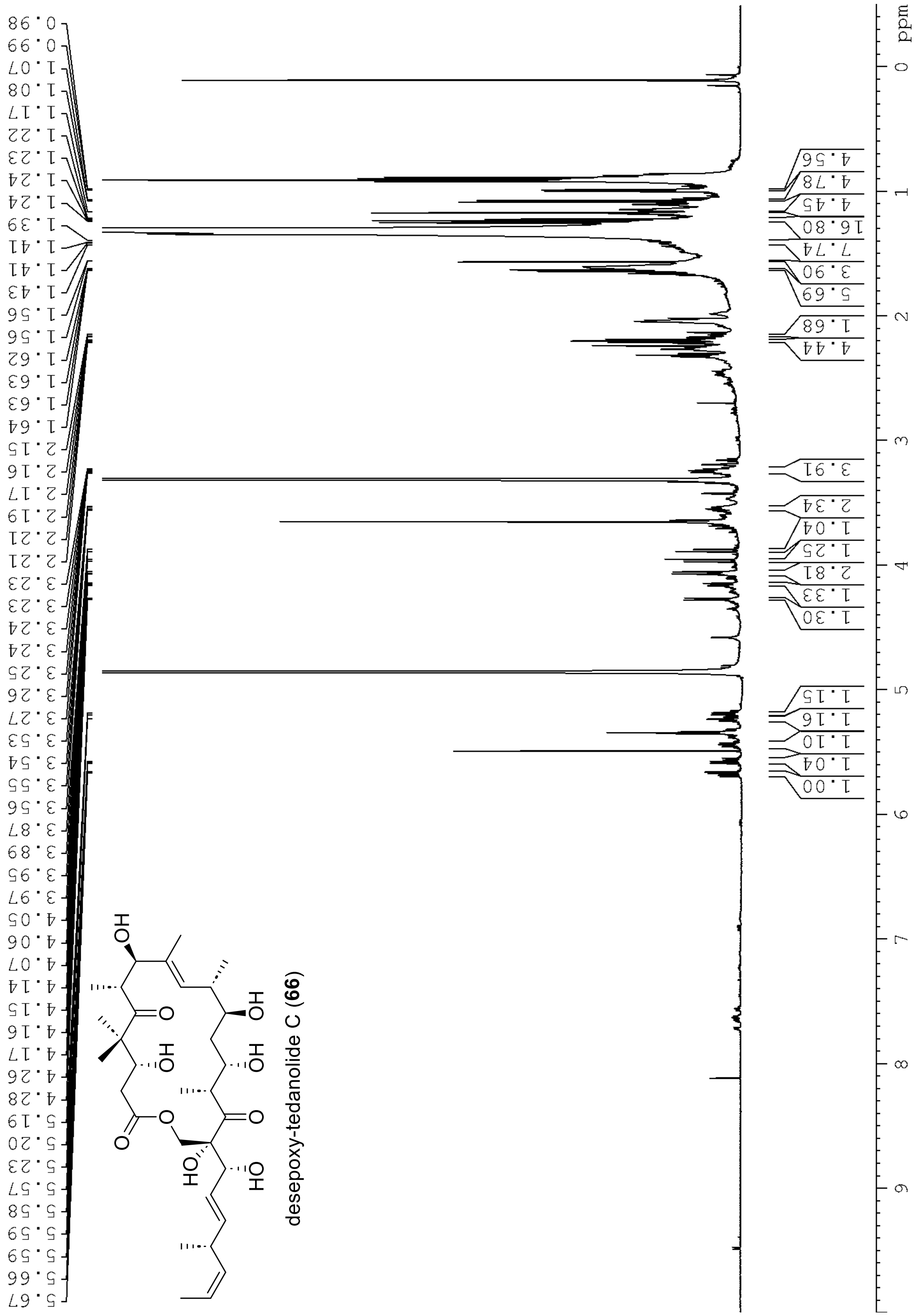

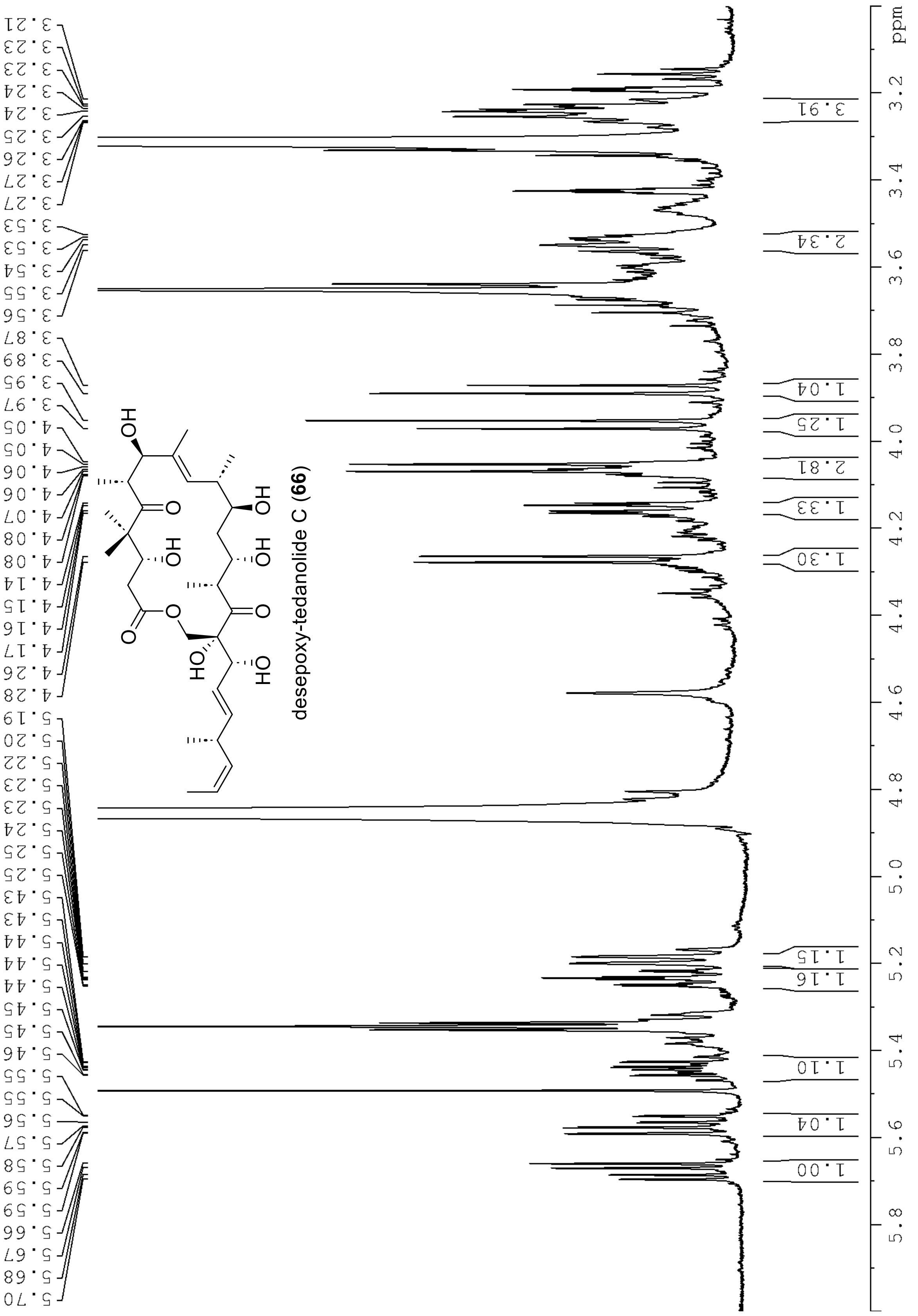
desepoxy-tedanolide C (66)
3.21 3.23 3.23 3.24 3.24 3.25 3.26 3.27 3.27
3.53 3.53 3.54 3.55 3.56
3.87 3.89 3.95 3.97
4.05 4.05 4.06 4.06 4.07 4.08 4.08 4.14 4.15 4.16 4.17 4.26 4.28
5.19 5.20 5.22 5.23 5.23 5.24 5.25 5.25
5.43 5.43 5.44 5.44 5.44 5.45 5.45 5.46
5.55 5.55 5.56 5.57 5.58 5.59 5.59 5.66 5.67 5.68 5.70
3.91 2.34 1.04 1.25 2.81 1.33 1.30 1.15 1.16 1.10 1.04 1.00
ppm 3.2 3.4 3.6 3.8 4.0 4.2 4.4 4.6 4.8 5.0 5.2 5.4 5.6 5.8

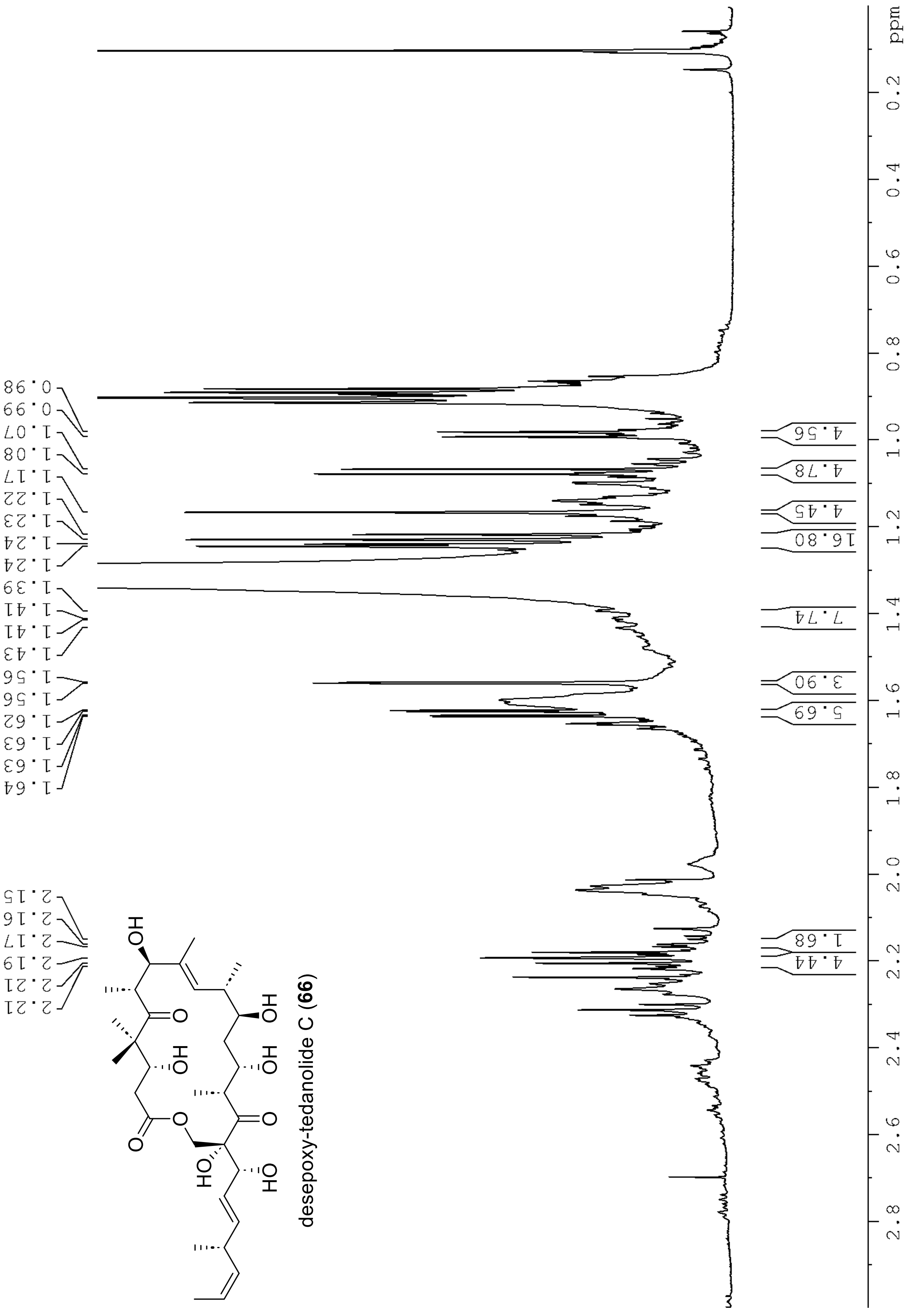
desepoxy-tedanolide C (66)
0.98
0.99
1.07
1.08
1.17
1.22
1.23
1.24
1.24
1.39
1.41
1.41
1.43
1.56
1.56
1.62
1.63
1.63
1.64
2.15
2.16
2.17
2.19
2.21
2.21
4.56
4.78
4.45
16.80
7.74
3.90
5.69
1.68
4.44
ppm
0.2
0.4
0.6
0.8
1.0
1.2
1.4
1.6
1.8
2.0
2.2
2.4
2.6
2.8

^{1}H-^{1}H-COSY (CD_3OD) of desepoxy-tedanolide C (**66**)

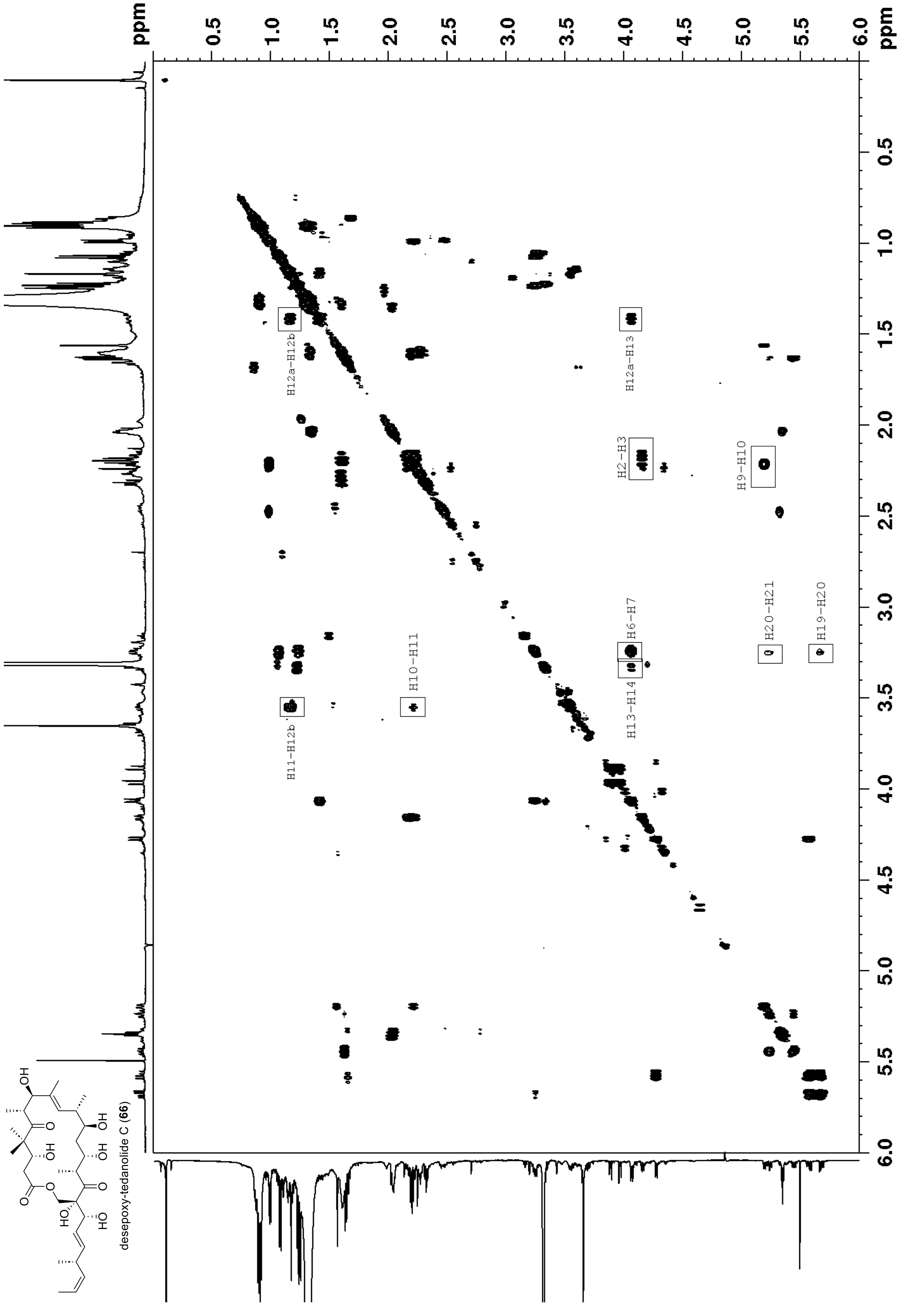

^{1}H-^{13}C-HSQC (CD_3OD) of desepoxy-tedanolide C (**66**)

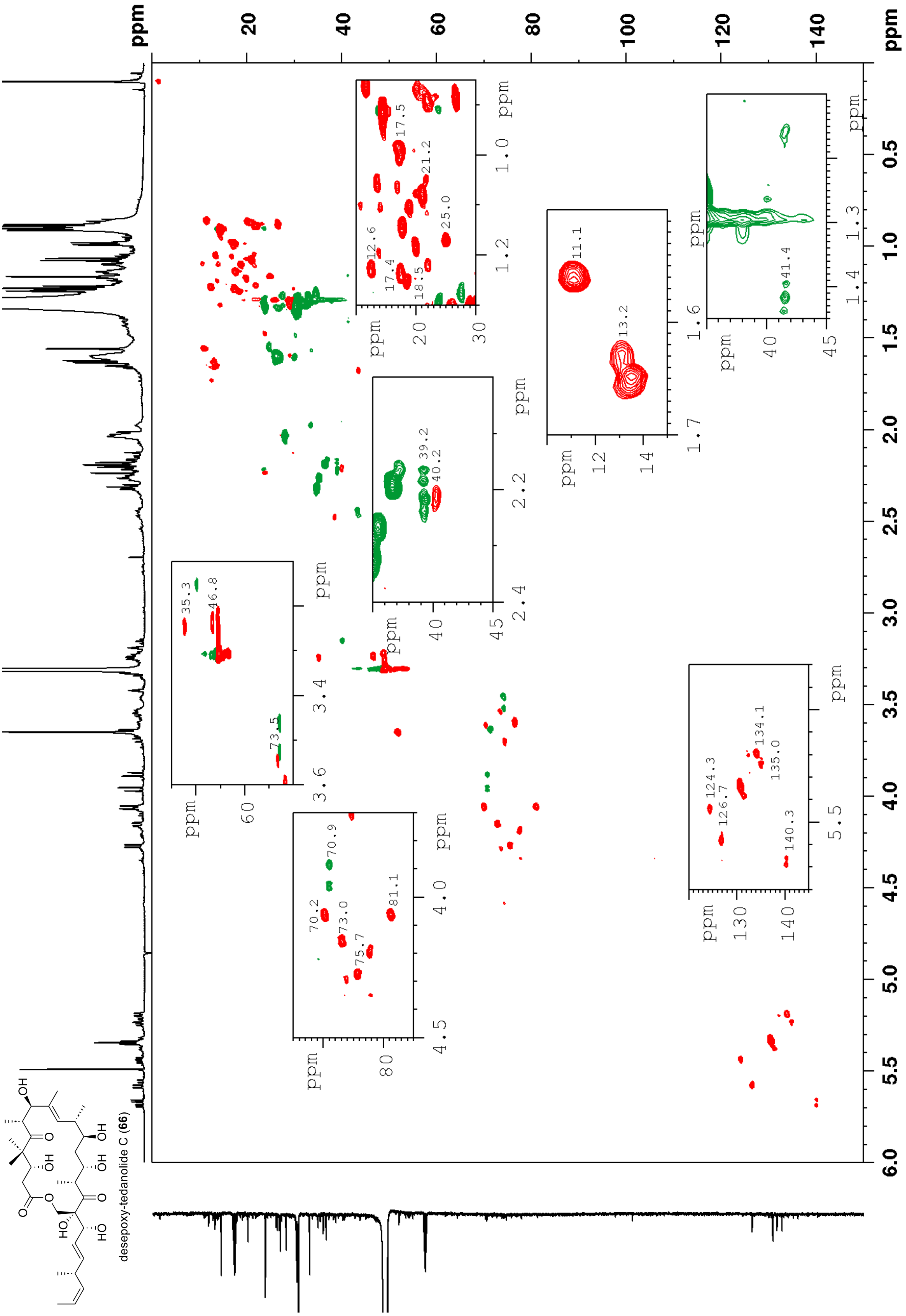

^{1}H-^{13}C-HMBC (CD_3OD) of desepoxy-tedanolide C (**66**)

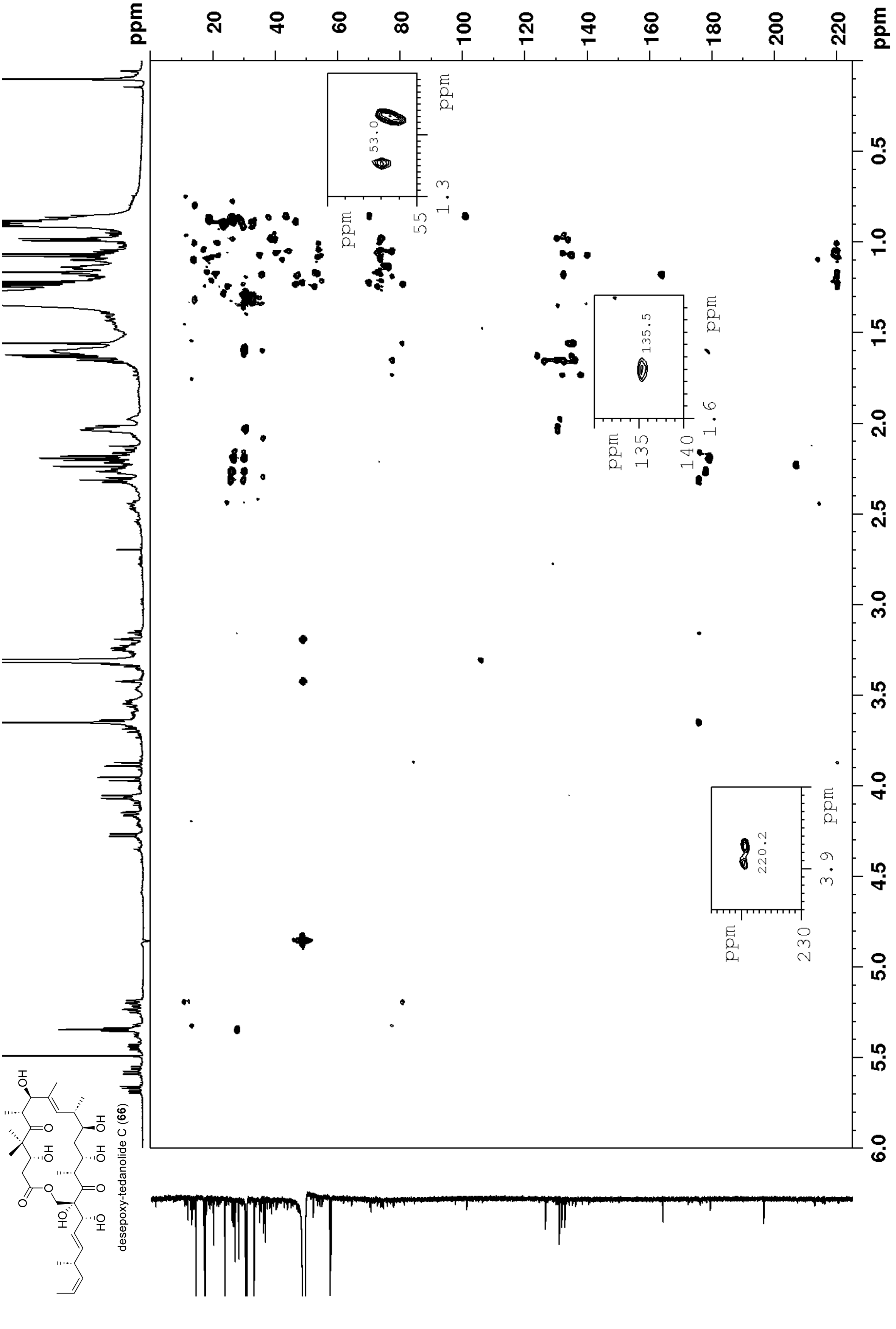

Supplement: Supplementary file 1 — jo3c02437_si_001.pdf [file jo3c02437_si_001.pdf]
